# A Systematic Review of Design of Electrodes and Interfaces for Non-Contact and Capacitive Biomedical Measurements: Terminology, Electrical Model, and System Analysis

**DOI:** 10.3390/s26041374

**Published:** 2026-02-22

**Authors:** Luka Klaić, Dino Cindrić, Antonio Stanešić, Mario Cifrek

**Affiliations:** University of Zagreb Faculty of Electrical Engineering and Computing, 10000 Zagreb, Croatia; dino.cindric@fer.unizg.hr (D.C.); antonio.stanesic@fer.unizg.hr (A.S.); mario.cifrek@fer.unizg.hr (M.C.)

**Keywords:** electric potential sensor, biopotential electrode, wet electrode, dry electrode, insulated electrode, contactless electrode, capacitive electrode, cECG

## Abstract

With the advent of ubiquitous healthcare and advancements in textile industry, non-invasive wearable biomedical solutions are becoming an increasingly attractive alternative to in-hospital monitoring, allowing for timely diagnostics and prediction of severe medical conditions. Non-contact biopotential monitoring is particularly promising because non-contact biopotential electrodes can be applied over clothing or embedded in the material without almost any preparation. However, due to the intricacies of capacitive coupling they rely on, the design of such electrodes and their interface with the body plays a key role in achieving measurement repeatability and their widespread utilization in clinical-grade diagnostics. Based on exhaustive investigation of several decades of the literature on non-contact and capacitive biopotential electrodes and electric potential sensors, this study is intended to serve as a state-of-the-art overview of their historical development and design challenges, a collecting point for important research theories and development milestones, a starting point for anyone seeking for a soft head start into this research area, and a remedy for occasional misnomers and conceptual errors identified in the existing papers. The ultimate goal of this comprehensive analysis is to demystify phenomena of non-contact biopotential monitoring and capacitive coupling, systematically reconciliate terminological inconsistencies, and enhance accessibility to the most important findings for future research. To accomplish this, fundamental concepts are thoroughly revisited—from fundamentals of electrochemistry and working principles of capacitors and operational amplifiers to system stability and frequency-domain analysis. With the use of various mathematical tools (Laplace transform, phasors and Fourier analysis, and time-domain differential calculus), discussions on non-contact and capacitive biopotential electrodes, collected from the 1960s onward, are for the first time compiled into a unified, abstracted, bottom-up analysis. The laid-out inspection provides analytical explanation for various aspects of measurement results available in the referenced literature, but also serves an educative purpose by devising a methodological framework that can be easily applied to other similar research fields. Firstly, the differences and similarities between wet, dry, surface-contact, non-contact, capacitive, insulated, on-body, and off-body biopotential electrodes are clarified. For this purpose, equivalent electrical models of various non-invasive biopotential electrodes are analyzed and compared. As a result, a proposal for a revised classification of biopotential electrodes is given. Secondly, instead of using the concept of a purely capacitive biopotential electrode, a test is proposed for assessing the predominant coupling mechanism achieved with an electrode over an insulating layer. Thirdly, a fundamental model of a buffer active non-contact biopotential electrode and its interface with the body is built and generalized, and the proposed test is applied for analyzing the influence of voltage attenuation and phase shifts on signal morphology. Lastly, guidelines for designing the described electrode–body interfaces are proposed, along with a discussion on practical aspects of their implementation.

## 1. Introduction

### 1.1. Introduction and Motivation

Cardiovascular diseases and respiratory disorders remain among the leading causes of death worldwide [1,2,3], afflicting more and more people and becoming the key public health concern, especially since the recent COVID-19 pandemic. A hectic sedentary lifestyle, intense stress at work, persistent geopolitical conflicts, and exacerbating climate change severely affect not only physical but also mental health, aggravating depression, anxiety, and sleep disorders. The rapid aging of the ever-growing population [4] and global economic issues burden the often slow-adapting healthcare system, demanding cost-effective personal care solutions and urging the extension of health services to out-of-hospital monitoring in domestic environments [5]. Unobtrusive health tracking in subjects’ everyday surroundings [6] allows for timely diagnostics and prompt intervention, which could prevent further worsening of unrecognized health issues and eventually foresee conditions such as stroke and cardiac arrest. Providing continuous hands-on insight into the state of wellness can significantly speed up the process of preliminary diagnostics and lower medical expenses, especially given the fact that some of the main risk factors associated with health issues are a consequence of unhealthy life habits and physical inactivity. This way, out-of-hospital long-term monitoring solutions have a chance to serve as the first line of defense against the development of chronic diseases and prolonged treatments.

For this purpose, biomedical recordings are widely used as indicators of psychophysiological state. Specifically, biopotential signals that arise from the body-area electric field, created as a result of physiological and electrochemical processes within the human body, can serve as a viable diagnostic tool. For instance, electrocardiogram (ECG) could provide information on abnormalities in heart activity, while electromyogram (EMG) could indicate musculoskeletal disorders and assess ergonomic issues. With the advent of ubiquitous availability of versatile smart gadgets, miniaturization of electronic devices, and rapid development of advanced polymers and textiles, these possibilities extend to a wide portfolio of wireless remote applications for quality-of-life enhancement that facilitate the concepts of telemedicine, e-health, and human-centered healthcare. They can be found in consumer-oriented products such as stress monitors, step counters, and fitness trackers, sports physiology and recreation, rehabilitation treatment, ambient assisted living, fall detection and human–machine interface technology for the disabled and elderly, veterinary medicine, and the monitoring of body vital signs for life-threatening professions, such as military and firefighting.

Since such devices must first and foremost be simple-to-use, affordable, portable, and convenient, non-invasive biomedical measurement methods are preferred over more accurate implantable devices, especially for the purpose of long-term monitoring during activity and movement, when minimal invasiveness and obstruction of the user must be achieved. Many unobtrusive and non-contact methods have been developed, including Superconducting Quantum Interference Device (SQUID) magnetometers, thermography, photoplethysmography (PPG), ballistocardiography (BCG), and radar, which are described and compared in [7,8,9,10,11]. Among them, capacitive sensing, as a cost-efficient portable alternative, is proving to be a beneficial technology in a wide range of applications, such as touch screens, pressure, strain, and temperature sensing [12], dimensional metrology [13], and fluid presence [14] and liquid-level detection [15], environmental [16] and plant monitoring [17,18,19], and biochemical sensing [20]. The history of capacitive sensing is summarized in [10].

Electrophysiological capacitive sensing applications in particular are emerging. Compared to piezoresistive, piezoelectric, and iontronic sensing strategies [21], non-contact capacitive biopotential sensing offers one of the most simple-to-use approaches whilst allowing a small form factor, reusability, low power consumption, and low cost, which makes it especially practical for mobile, wearable, and in-vehicle applications [22]. Also, these sensors do not require direct contact with skin because they are based on capacitive coupling between the electrode and the human body, accomplished over one or more insulating layers that separate the sensor from the skin. This means that such non-contact (also called contactless or indirect-contact) and capacitive biopotential electrodes can be applied over clothing or embedded in the material, maximizing user safety and freedom of movement. Instead of being worn, non-contact and capacitive biopotential electrodes could also be placed on furniture (sofa [23], chair [24,25], wheelchair [26], lavatory seat [27], or bathtub [28]), car seats [22,29,30,31] and safety belts [32], bicycle handles [33], airplane seats [34], or incorporated into a mattress [35]. They are particularly suitable in situations when direct contact with skin is difficult to achieve or undesirable (for instance, measurement on animals [36,37,38] or on burned skin [39], or monitoring infants in neonatal intensive care units [40]). They can even be used underwater [41,42]. Principles of capacitive sensing can be employed in various areas of biomedical measurement, such as BCG [43,44] and blood pressure monitoring [45], as well as in electrocardiography (ECG) and heart rate monitoring [46,47], used as alternative methods to PPG and mechanocardiography (MCG)—ballistocardiography (BCG), seismocardiography (SCG) and gyrocardiography (GCG) [48]. Further applications can be found in electrooculography (EOG) [49], electromyography (EMG) [50,51], and electroencephalography (EEG) [52,53], along with steady-state visually evoked potentials (SSVEPs) and auditory steady-state response (ASSR) [54]. Capacitive sensing can also be utilized for electrosurgery [55], bioimpedance measurement [56,57], electrical impedance tomography (EIT) [58], impedance cardiography (ICG) [59], and impedance plethysmography (IPG) [60,61], as well as respiration [62,63], sleep respiration [64] and sleep apnea [65], sleep position classification [66] and sitting posture monitoring [67], sweat rate measurement [12] and stress level measurement during driving [68]. Another use case is human localization [69,70], along with motion and gesture recognition [71] and emotion recognition [72]. Human activity sensing can be further extended to human body communication [10,73,74,75], along with capacitive intrabody communication (IBC) [75]. Also, applications of capacitive power transfer have gained popularity [76,77].

In this study specifically, non-contact and capacitive sensing of biopotential signals (ECG, EMG, and EEG in particular) will be in focus. The contactless capacitive principle, on which this sensing is based, and the fact that the utilization of non-contact and capacitive biopotential electrodes does not require skin preparation, pave the way for plug-and-play brain–computer interfaces (BCIs) [54,78], body area networks (BANs) [10,79,80,81], multimodal long-term home monitoring [6,26,82,83], and various physical rehabilitation and robotic applications [12,84,85], employed without the need for the on-site presence of trained medical professionals.

In spite of offering ease of use and short preparation time, pervasiveness of non-contact and capacitive biopotential electrodes is limited due to their sensitivity to ambient conditions, environmental noise, displacement, and movement of surrounding objects and people in the vicinity. These phenomena impair repeatability of measurements and significantly reduce signal quality achieved in non-contact and capacitive biopotential recording compared to its commonly used counterparts—surface-contact electrodes that are placed directly on the skin, such as the popular wet Ag/AgCl electrodes. Advanced software-based filtering techniques and machine learning can be utilized to enhance robustness. However, this can affect real-time performance and often demands a posteriori processing algorithms, which could hinder the very idea of designing a health monitoring system that would not need additional on-site technical assistance. In this study, it will be shown that most of the issues with using non-contact and capacitive biopotential electrodes originate from the tradeoffs in their design, as well as from unwanted coupling between the system and the environment. To counteract these challenges and allow for the creation of larger datasets that can later be used for reliable healthcare diagnostics, it is necessary to come up with solutions that will circumvent the weaknesses of capacitive coupling early in the recording stage. In this process, the hardware design of electrodes and their interface with the human body will prove to play a role of the utmost importance.

### 1.2. Objectives and Contributions

The main objectives of this study are the following:

1. This study is based on previously published reviews on non-contact and capacitive biopotential electrodes [86,87,88,89,90,91,92,93,94,95]. Yet, it is composed with the aim of consolidating various equivalent electrical models of the electrode–body interface and clarifying the terminology used in the field of non-contact and capacitive biopotential sensing, which is often inconsistently applied, as this complex matter is discussed from the perspective of various scientific disciplines such as electronics, biomedicine, chemistry, and textile technology. The need for such demystification of erroneously used terms and unclear concepts was intelligibly emphasized in the 2010s by several letters and comments on earlier papers and reviews [96,97,98,99]. Relying on the caveats therein expressed, this study is carried out with the hope of preventing such misinterpretations in the future. As a result of a systematic review of differences and similarities between electrical models of various electrode–body interfaces, a revised classification of biopotential electrodes is proposed. Accordingly, practical limitations of capacitive coupling are addressed and extended to the concept of predominantly capacitive coupling. A test for assessing the predominant coupling mechanism is proposed and employed to examine the parameters of the generalized equivalent electrical model of non-contact biopotential electrodes and their interface with the body. The resulting framework analytically corroborates the common practice, leading to the proposal for guidelines for designing non-contact electrode–body interfaces. This first objective results in four contributions, which are listed and visualized later in this section.
2. This study also serves as a review paper, covering the trends that have been followed since some of the most recent general overviews on non-contact and capacitive biopotential electrodes [90,93] have been published. Hence, when possible and convenient, papers dating from 2016 onward are chosen for modern applications and system examples.
3. By surveying discussions provided in the cited papers and summarizing their outcomes through a bottom-up analytical approach, up-to-date feedback to other research groups in the area of non-contact and capacitive biopotential measurement is given. The aim of such a report is to identify major challenges and speed up future research, steering it towards open questions that have to be addressed in order for non-contact and capacitive biopotential electrodes to live up to their commercialization and become standard practice in long-term biomonitoring and wearable biomedical devices. The adopted systematic approach and narrative style have allowed authors to carefully choose the most relevant papers and thoroughly tailor the order of their introduction, creating an “all-in-one-place” digital library that garners the fundamentals of non-contact and capacitive biopotential sensing. The established corpus can later be used to gain quick access to important historical milestones, as well as to instructive theoretical discussions and practical design examples. The build-up of a modern, up-to-date non-contact biopotential electrode, with all of its intricacies, is supported by book chapters, theses, papers, reports, lecture notes, handouts, application notes, and datasheets to accompany the calculations and simulations with practice, thereby verifying the theoretical background scattered across papers published over the last several decades. To fully grasp the context behind common practice and important tradeoffs in the design, no steps in the analysis are skipped, even in the case of applying elementary mathematical derivations and fundamental concepts of electronics or signals and systems analysis, which are elsewhere in the respective literature often implied or considered to be self-explanatory. The ultimate goal of these guiding principles is to make this study as suitable as possible, even for readers without a priori knowledge of biopotential electrodes, providing them with an educative example of using mathematical apparatus and engineering fundamentals in practice and encouraging them to join the deep dive into this sometimes-daunting research niche. Strategies for improving readability will be further described in Section 2 on Materials and Methods.

Based on the first objective of this study, the provided synthesis of the literature yields four main contributions as follows:

1. Contribution 1: Proposal for a test for assessing the predominant mechanism of coupling achieved over an insulating layer (Section 3.1);
2. Contribution 2: Proposal for a generalized equivalent electrical model of the interface between the body and a non-contact biopotential electrode (Contribution 2.1, Section 3.1), that is further developed into an equivalent electrical model of a buffer active non-contact electrode (Contribution 2.2, Section 4.1);
3. Contribution 3: Proposal for revised classification of biopotential electrodes with respect to inaccuracies in the used terminology (Section 3.1);
4. Contribution 4: Proposal for guidelines for designing non-contact electrode–body interfaces (Section 4.2), along with practical aspects of their implementation (Section 4.2 and Section 4.3).

**Figure 1:**
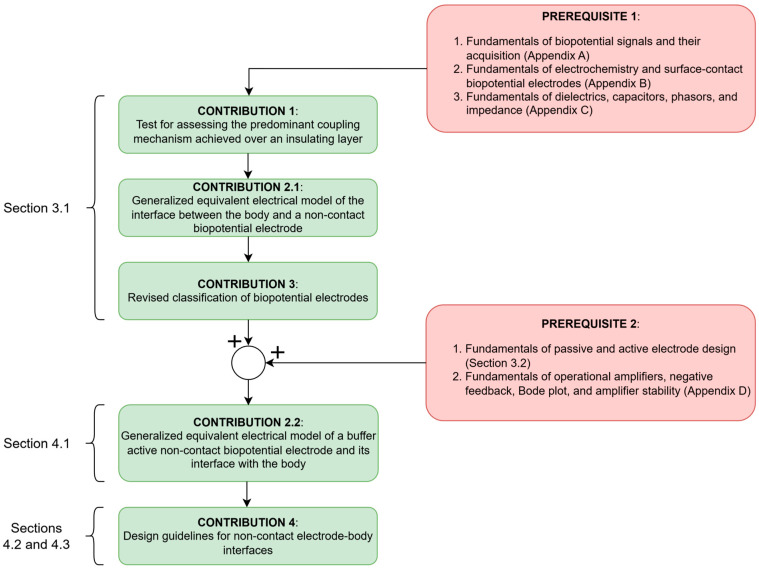
Flow diagram representing the four contributions and two prerequisites in chronological order, along with related sections and appendixes. Prerequisite 1 discusses the content that is a prerequisite for understanding Contributions 1, 2.1 and 3 presented in Section 3.1. Afterward, Section 3.1 and Prerequisite 2 are prerequisites for Contributions 2.2 and 4 presented in Section 4. This is indicated by the summing junction.

These four contributions are ordered chronologically and visualized in the following flow diagram (Figure 1), along with related sections and appendixes. More information on the paper structure will be given in Section 1.3.

### 1.3. Paper Structure

In terms of linear narrative, the paper is structured as follows (Figure 1).

Section 2 expounds on the research methodology and tools used, reporting on the genesis of this study, strategies for improving readability, and the rationale for using four appendixes.

In Section 3.1, non-contact and capacitive biopotential electrodes are introduced. Before a detailed examination of the subject, Appendix A, Appendix B and Appendix C are cross-referenced to conveniently provide the reader with the knowledge that is a prerequisite for understanding non-contact and capacitive biopotential electrodes (Prerequisite 1).

Firstly, Appendix A gives a brief introduction into the origin of biopotential signals and the role of electrodes in their acquisition. After cross-referencing Appendix A, with respect to the observed biopotential signals, Section 3.1 establishes the frequency range of interest for the rest of the paper.

Next, Section 3.1 cross-references Appendix B, where fundamentals of electrochemistry and a comprehensive comparison of commonly used wet and dry surface-contact biopotential electrodes are given, along with a detailed breakdown of their structure design and equivalent electrical models of their interfaces with the body. Through listing advantages and disadvantages of various surface-contact biopotential electrodes, motivation for using non-contact and capacitive biopotential electrodes as an alternative is further elaborated.

Thereafter, Section 3.1 continues by presenting the concept of non-contact and capacitive biopotential electrodes and highlighting the key differences with respect to surface-contact biopotential electrodes, described in Appendix B. After a brief introduction into capacitive coupling, Appendix C is cross-referenced. Therein, fundamentals of dielectrics, capacitors, phasors, and impedance are recalled.

After cross-referencing three appendixes and establishing the frequency range of interest for the rest of the paper, Section 3.1 proceeds with a discussion on the appropriate model that will be used for describing the interface between the body and non-contact and capacitive biopotential electrodes. Based on practical aspects of capacitive coupling and caveats behind electrical properties of real-world dielectrics and insulating layers, a test is proposed for assessing the predominant mechanism of coupling (capacitive or resistive) achieved over an insulating layer (Contribution 1). A model of a single insulating layer is further expanded into a generalized equivalent electrical model of the interface between the body and a non-contact biopotential electrode (Contribution 2.1). As a result of discussions on misterming and methodological inconsistencies present in the existing literature, the initially used term “insulating layer” and group term “non-contact and capacitive electrodes” (or occasionally just “non-contact electrodes” for simplicity’s sake) are refined and corrected. Accordingly, the end of Section 3.1 brings a proposal for the classification of biopotential electrodes based on their distance from the body, type of contact, and invasiveness (Contribution 3).

Afterward, in Section 3.2, the difference between single-ended and differential signals is explained, various amplifier configurations are compared, and benefits of using an active electrode design approach over a passive design are discussed. Based on the comparison between two main types of active electrodes, the buffer active electrode is highlighted, and the rest of the prerequisites and assumptions for further investigation of its interface with the body are established.

Next, in Section 4, the developed model of a non-contact electrode–body interface is expanded into a model of a single buffer active non-contact biopotential electrode (Contribution 2.2). The entire system (interface–electrode–preamplifier) is defined as a cascade of two subsystems: the input voltage divider and the buffer preamplifier.

Section 4.1 begins by cross-referencing Appendix D. Therein, the buffer preamplifier subsystem is analyzed, starting from the fundamentals of operational amplifiers, negative feedback, Bode plot, and amplifier stability (Prerequisite 2).

Thereafter, Section 4.1 continues by identifying equivalent impedance blocks and deriving the transfer function of the entire system, observed as a two-port network. Based on the fundamentals of system analysis, the properties and stability of the entire system are inspected from its poles and zeros. Accordingly, the impulse response of the system and its frequency characteristic (frequency response) are derived as well.

In Section 4.2, the influence of the second subsystem, the input voltage divider, is further examined in depth. Therein, investigation of the influence of equivalent electrical parameters of the electrode–body interface is divided into three steps: influence on the area of predominantly capacitive coupling (step 1), influence on voltage attenuation and the magnitude response (step 2), and influence on the phase response and signal morphology, which is demonstrated on ECG signals (step 3). In each of the three steps, calculations are corroborated by simulation results, and each step ends with a summarizing paragraph. Following the analytical discussion and simulation results within these three steps, guidelines for designing the investigated electrode–body interfaces are given, along with considerations on practical aspects of their implementation (Contribution 4).

Lastly, in Section 4.3, investigation of the frequency response of the input voltage divider subsystem from Section 4.2 is extended to include the buffer preamplifier subsystem from Appendix D. This way, results of the analysis are further confirmed and abstracted by observing the total frequency response in accordance with the total transfer function and impulse response from Section 4.1. The analysis ends with a discussion on intricacies of designing systems for experimental validation of non-contact electrode–body interfaces, and the generalized model is further evaluated in the context of physical measurements that can be found in the literature.

Finally, Section 5 provides conclusions, laid out as another layer of abstraction. This section highlights the most important findings, equations, figures, and proposals, providing further motivation for future research.

## 2. Materials and Methods

In the previous section, the motivation, main objectives, contributions, and structure of this study were elaborated. This section encompasses the means and methods of the performed meta-analysis, carried out in accordance with the updated Preferred Reporting Items for Systematic Reviews and Meta-Analyses (PRISMA) 2020 statement [100,101]. In the Supplementary Materials, the corresponding PRISMA 2020 for Abstracts Checklist is given in Table S1, the PRISMA 2020 Checklist in Table S2, and the PRISMA 2020 Flow Diagram in Figure S1. For details on the availability of this material, readers can refer to the paragraph “Supplementary Materials” at the end of this paper. A detailed and complete report that complies with PRISMA 2020 statement guidance is available in this section. Additionally, sources of financial support for this research are listed in the paragraph “Funding” at the end of this paper.

This study stemmed from state-of-the-art research carried out during the authors’ Master’s studies and ongoing PhD studies. Rather than being a routine update report, it is an unforeseen by-product of a slowly but steadily increasing learning curve, boosted through the authors’ self-practice, as well as experience built during their several-years-long joint research activities. In that sense, the initial search strategy was driven by the need for extending personal knowledge and clarifying the unclear concepts within the research group. In the beginning (year 2022), each of the four authors independently provided the starting syllabus with a list of articles that they considered to be representative and essential for familiarizing them with the research area of non-contact and capacitive biopotential monitoring. The collected papers were stratified into several levels:

1. Biomedical monitoring;
2. Biopotential monitoring;
3. Non-invasive biopotential monitoring;
4. Non-contact and capacitive non-invasive biopotential monitoring.

Additionally, the papers were given various category tags for future use, depending on the topics they cover:

1. Motivation for biomedical and biopotential measurements;
2. Type of biopotential electrodes discussed based on their distance from the body, type of contact, and invasiveness;
3. Motivation for using non-contact and capacitive biopotential electrodes specifically;
4. Type of biomedical and/or biopotential application of non-contact and capacitive electrodes;
5. Original and unique applications of non-contact and capacitive biopotential electrodes;
6. Important milestones in the historical development of non-contact and capacitive biopotential electrodes;
7. Elaborate equivalent electrical models;
8. Elaborate numerical models;
9. Elaborate analytical models;
10. Elaborate calculation examples;
11. Elaborate methods of procedure, protocols, and evaluation metrics;
12. Elaborate procedures for a specific electrical measurement;
13. Illustrative measurement results;
14. Discussions on electrical modeling of electrode–body interfaces;
15. Discussions on insulating materials and their fabrication;
16. Discussions on electrode size, shape, material, and fabrication;
17. Discussions on the number of electrodes used and their configuration;
18. Discussions on feasibility and limitations in activity monitoring;
19. Discussions on feasibility and limitations in long-term monitoring;
20. Discussions on feasibility and limitations in clinical-grade applications;
21. Subcategories on analysis and illustrative examples of remaining challenges (parasitic elements and stability analysis, intrinsic noise, crosstalk, motion artifacts, extrinsic noise);
22. Subcategories on addressing these remaining challenges (electrode–body interface design, discrete and integrated hardware solutions, digital signal processing, machine learning approach and deep learning methods).

The literature was exchanged among the authors, thoroughly examined, and further inspected in the context of fundamental concepts of electronics and system analysis. As the knowledge was expanded, a deeper insight obtained through discussions, thought experiments, and physical measurements provided a clearer understanding of various phenomena behind non-contact biopotential monitoring and intricacies of capacitive coupling. This allowed authors to delve into more complex matter, reexamine the papers from a new perspective, and eventually create a densely connected network of abstract relations between distinct studies. This way, the reference list was iteratively expanded and reshaped.

Soon, the examined literature revealed room for a bottom-up analysis with a scope stretching from early work to the newest advanced solutions. The turning point for the decision to compose a detailed report on the basis of joint research efforts was the discovery of the aforementioned letters and comments on earlier papers and reviews [96,97,98,99], which suggested the presence of erroneous assumptions and methodological issues in common practice that remain to be remedied. Afterward, further investigation was accompanied by a systematic electronic search, performed in the pursuit of works similar to the ones already explored, as well as new findings that had emerged in the meantime.

An electronic search was performed iteratively throughout the years 2023, 2024, and 2025. Eligible studies were obtained by exploring the Scopus database, IEEE Xplore digital library, and the Google Scholar search engine. The coverage was from 1965 to the present date in 2025. Papers were explored in the case of both surface-contact biopotential measurement and non-contact and capacitive biopotential measurement to allow for the drawing of further parallels between various types of biopotential electrodes.

With regard to biopotential electrodes, an individual search of the following terms was undertaken:

1. biomedical/bio-medical;
2. biopotential/bio-potential;
3. unobtrusive/non-obtrusive/nonobtrusive/non-intrusive/nonintrusive;
4. non-invasive/noninvasive;
5. surface;
6. contact;
7. wet;
8. gel;
9. electrolyte;
10. paste;
11. dry/dry-contact/dry contact;
12. gelless/gel-less;
13. pasteless/paste-less;
14. active;
15. passive;
16. on-body;
17. off-body;
18. non-contact/noncontact;
19. indirect;
20. contactless;
21. insulating/insulated;
22. capacitive.

Considering electrode material and fabrication, search queries were as follows:

1. rigid;
2. multi-layer;
3. substrate;
4. soft;
5. flex;
6. stretch;
7. nano;
8. fabric;
9. textile;
10. patch.

Considering biopotential signal measurements and typical applications, search queries were as follows:

1. ECG;
2. cECG;
3. heart;
4. heart rate;
5. EMG;
6. muscle;
7. EEG;
8. brain;
9. evoked;
10. EOG;
11. seat;
12. chair;
13. bed;
14. mattress;
15. cushion;
16. drive/driving;
17. ambulatory;
18. body area/body-area/BAN;
19. long-term;
20. home.

Finally, in terms of analysis of electrode–body interfaces, search queries were as follows:

1. skin;
2. impedance;
3. sweat;
4. perspiration;
5. humid;
6. moist;
7. motion;
8. movement;
9. artifact/artefact;
10. air gap;
11. triboelectric;
12. microphony/microphonic;
13. noise;
14. interference;
15. powerline/power-line/power line;
16. right leg.

To further boost the applicability of discussions, aside from conference and journal papers and several aforementioned letters and comments [96,97,98,99] that allowed us to minimize the risk of bias and limitations in the searching process, book chapters and guides were also included, along with lecture notes and handouts (Massachusetts Institute of Technology and University of Edinburgh), as well as several contributive dissertations and theses [102,103,104]. Also, application notes, reports and bulletins, and technical datasheets from various companies such as Texas Instruments (Dallas, TX, USA), along with Burr-Brown (Tucson, AZ, USA), Analog Devices (Wilmington, MA, USA), Renesas Electronics (Tokyo, Japan), Microchip Technology (Chandler, AZ, USA), Murata Manufacturing (Kyoto, Japan), and AIC tech (Moka, Japan), were added in pursuit of analogous reports at the level of fundamental knowledge. Several topics of the technical literature were explored, with an emphasis on fundamentals of:

1. Action potentials and biopotential signals;
2. ECG biopotential signal and ECG measurement methods;
3. Electrochemistry;
4. Electrical modeling of real-world electric components and electrode–body interfaces;
5. Capacitors and dielectrics;
6. Electromagnetism and Maxwell’s equations, fringing fields, and finite models;
7. Operational amplifiers and negative feedback;
8. Stability analysis;
9. Time-domain and frequency-domain system analysis;
10. Intrinsic noise analysis;
11. Triboelectricity;
12. Microphony;
13. Interference analysis, electric and magnetic coupling, cabling, and shielding;
14. Fabrication of e-textiles and smart fabrics.

An overview of the technical literature will be given on several occasions throughout the rest of the paper.

Aside from the electronic search, further studies were also searched by screening the reference list in every relevant article and report examined. In addition, authors and research groups were identified. For instance, for many prominent authors, the rest of their publications were explored, such as in the case of the [78,83,87,103,105,106,107,108,109] collection. In such cases, both published and unpublished papers were considered. However, to enhance the accessibility of the study, analogous works published in English were preferred. Additionally, in accordance with the second objective (Section 1.2), when possible, papers dating from 2016 onward were chosen for modern applications and system examples.

Finally, amongst the extensive collection of examined papers, the most representative ones were chosen based on the following questionnaire:

1. Is the work subject to the issues addressed in the reference letters and comments [96,97,98,99]?
2. What is the level of complexity and which level of prior knowledge is required for understanding the work?
3. Does the work have an educational value?
4. Is the work significant in the context of historical development?
5. Is there an informative literature review present?
6. Is there an instructive research methodology or measurement protocol provided?
7. Are there any analytical or equivalent electrical models, illustrative calculation examples, or illustrative measurement results provided?
8. Are the phenomena described in the work clearly distinguishable and separately addressed?
9. Are the challenges presented in the work clearly distinguishable and separately addressed?
10. Is the presented solution clearly elaborated and differentiated from the existing solutions?

Based on these inclusion criteria, when possible, studies with a clear and intuitive structure, illustrative examples, and detailed analysis, that could be of use both at a beginner and at an intermediate level of knowledge, were preferred. For instance, paper [90] is included as an important recent review, yet commentary on this paper [98] is additionally referenced when appropriate. The chosen papers are then analyzed once again from the context of each category tag they had been assigned. For every cited source, full citation, along with the Digital Object Identifier (DOI), web address, and date of last access, are provided in the References section. Numbers of identified reports, screened reports, reports assessed for eligibility, and included reports are given in the PRISMA 2020 Flow Diagram in Figure S1 in the Supplementary Materials.

Specifically, the relevant review literature that will be cited throughout this study can be classified into the following categories:

- Dry surface-contact electrodes with a focus on noise analysis [110], as well as on material and structural design [111,112,113,114,115,116,117,118,119];
- Comparison of various types of non-invasive electrodes—in the context of EEG [120,121,122], BCIs [54,78,123], and human body communication [74];
- Non-contact and capacitive electrodes: general overview of capacitive sensing applications [12], overview specifically focused on biochemical applications [20], review from the perspective of electrosurgery [55], integrated unobtrusive biomedical sensing solutions [43], and specifically electric field and biopotential sensing [86,88,89,90]; reviews on biopotential measurement with the addition of noise analysis [87]; reviews focused on ECG [93] and EMG [91] applications, dielectrics [92], and structural design for wearability [21];
- Wearable design: modalities and prospects [124], advancements in devices for arrhythmia detection [125], hydrogel-based devices [126], textile-based electrodes [127,128,129,130], printed wearable electronics [131], biocompatibility [132] and biodegradability [133], wearable antennae [134,135,136], energy harvesting [137], plant monitoring [17], BANs [10,79,80,81], exoskeletons [84], and specifically smart-textile exosuits [85];
- Integrated design [138,139,140,141,142];
- Problem-oriented review papers: interference in ECG recordings [143] and motion artifacts removal techniques for wearable EEG [144,145] and specifically for non-contact and capacitive ECG measurements [94,95].

The resulting study is script-like, written on the basis of an elaborate storyline that was revised multiple times as earlier findings would stumble upon a contradiction or an exception, which allowed for further generalization of the analysis, as well as a finer reassessment of the authors’ perception. Based on the available reviews and discussions, it brings an integration of the gathered findings into a comprehensive step-by-step creation of the most frequently used up-to-date equivalent electrical model of non-contact and capacitive biopotential electrodes and their interface with the body. To corroborate the outcomes of the analysis, mathematical apparatus and engineering fundamentals are extensively used and followed by simulations in the MathWorks^®^ MATLAB R2025b environment [146] and LTspice^®^ 24.1.10 simulation software [147], illustrative examples, and supporting information obtained from the datasheets of off-the-shelf components that have been recently used in the 2010s and 2020s in similar applications. Since ECG is the most popular application commonly chosen by many papers delving into non-contact and capacitive biopotential sensing, most of the examples will be demonstrated on ECG measurement systems. For this purpose, two reference datasets will be used:

1. PhysioNet service from the MIT-BIH Arrhythmia database for standard clinical vital signs measurements [148];
2. UnoViS database for unobtrusive and non-contact medical monitoring in various scenarios ranging from a clinical study to measurements obtained while lying in bed and driving a car [149].

Incidentally, datasets for other methods of contactless sensing are reviewed in [11].

To further improve readability, several additional strategies are employed:

1. Beginnings of discussions inside each section are highlighted with a brief heading indicating the subject of the discussion;
2. Assumptions and simplifications that are used throughout this study are enumerated and highlighted within a dedicated paragraph titled “Assumption X.” This way, each assumption represents a stepping stone in the bottom-up analysis, summarizing the findings built upon the previous assumptions, as well as offering an abridged version of the content for quick access. Throughout the paper, nine assumptions are established in total.
3. Each topic discussed contains a brief literature overview, which serves as a collecting point for all the cited literature on the respective topic. The provided references offer a progressive learning path, as they span a broad spectrum of complexity, ranging from foundational concepts for novices to advanced, expert-level content.
4. Finally, to reduce the main body of the text, elementary mathematical derivations and prior knowledge (Prerequisites 1 and 2 in Figure 1) are strategically relocated to four appendixes, which are appropriately cross-referenced at the beginning of Section 3.1 and Section 4.1. Their content is described in Figure 1 and Section 1.3:
  - Appendix A. Origin and Acquisition of Biopotential Signals;
  - Appendix B. Surface-Contact Electrodes;
  - Appendix C. Fundamentals of Capacitors and Phasor Algebra;
  - Appendix D. First Subsystem: Operational Amplifier.

To preserve the linearity of the narrative, cross-reference of an appendix is treated as the point at which the reader should switch to the cross-referenced appendix and read its content before moving forward with the main body of the text. Appendix A, Appendix B and Appendix C are cross-referenced at the beginning of Section 3.1, whereas Appendix D is cross-referenced at the beginning of Section 4.1. Figures and equations in appendixes are prefixed with “A”.

## 3. Electrodes

### 3.1. Non-Contact Electrodes

**Figure A1:**
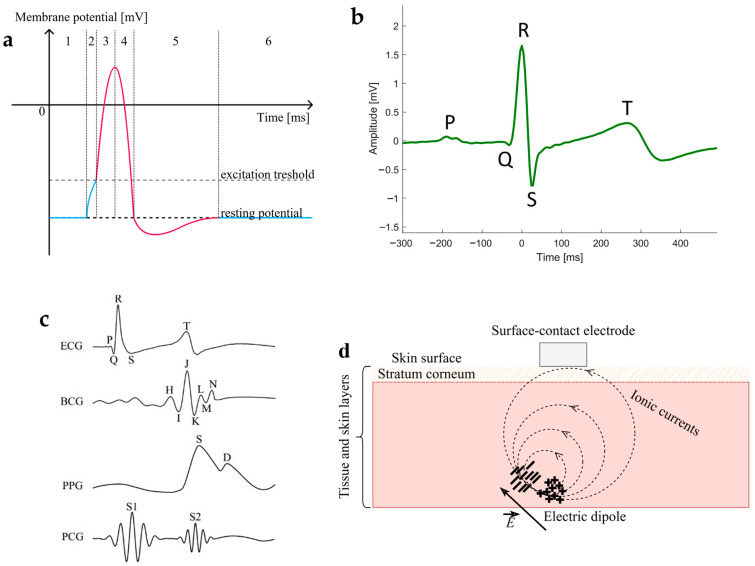
(**a**) Action potential waveform; the following parts of the response to a mechanical, chemical, or electrical stimulus are marked: (1) resting potential, usually −70 mV to −90 mV, (2) charging of membrane capacitance, (3) depolarization, (4) repolarization, (5) hyperpolarization, (6) return to resting potential [127,130,150,151]; (**b**) ECG signal with annotated PQRST complex (reproduced without modifications from [155], which is licensed under a Creative Commons Attribution-NonCommercial-NoDerivatives 4.0 International License (https://creativecommons.org/licenses/by-nc-nd/4.0/)); interesting visual representation of generation of ECG signal waveform as a sum of action potentials, along with basic anatomy of a human heart, can be found in [152]; (**c**) Comparison of ECG, BCG, PPG, and phonocardiography (PCG) waveforms, which are physiologically delayed with respect to ECG due to pulse transit time (reproduced without modifications from [36], which is licensed under a Creative Commons Attribution 4.0 International License (https://creativecommons.org/licenses/by/4.0/)); (**d**) Schematic representation of an electric dipole, ionic currents, and a surface-contact electrode [41,74,120,123].

**Origin and acquisition of biopotential signals.** In Appendix A, the origin of biopotential signals, such as ECG, EMG, and EEG, is briefly explained, as well as the role of electrodes in their acquisition. Also, references [150,151,152,153,154,155,156,157,158] are introduced, along with Figure A1. The comparison between invasive and non-invasive biopotential electrodes given therein further explains why the rest of the paper will be focused on non-invasive and surface-contact electrodes and their interfaces with the body. With respect to the biopotential signals that will be observed in this paper, the first assumption out of nine in total can be expressed as follows.

**Assumption** **1.** ***Biopotential amplitudes and frequencies of interest**. The vast majority of biopotential signals lie in the frequency range from sub-Hz frequencies (0.05 Hz or even 0.02 Hz in the case of clinical-grade ECG [154]) up to several kHz (20 kHz in the case of electrocochleography (EcochG) [104]), with most of the power concentrated at frequencies of up to 500 Hz. Therefore, the frequency range 0–20 kHz will from now on be referred to as the frequencies of interest, frequency range of interest, bandwidth of interest, frequency band of interest, or area of interest. On the other hand, typical amplitudes of biopotential signals reach the order of 1 mV, and the order of 10 mV in the case of EMG signals specifically. Amplitudes and bandwidths for different biopotentials are given in Table 1 in [92] and Table 1 in [95] (ECG), page 11 in [157] (EMG), Table 1 in [144] and Section 2.1 in [145] (EEG), and Table 5.1 in [104] (EOG and EcochG). These properties of biopotential signals will be recalled later and used to draw eight additional assumptions and simplifications. Most of the examples will be demonstrated on ECG measurements and compared with clinical ECG features (Figure A1b). More on noise sources in ECG signals, as well as on ECG signal frequency spectrum and clinical features, can be found in [152,153,154]. A brief history of ECG recording is given in [125].*

**Figure A2:**
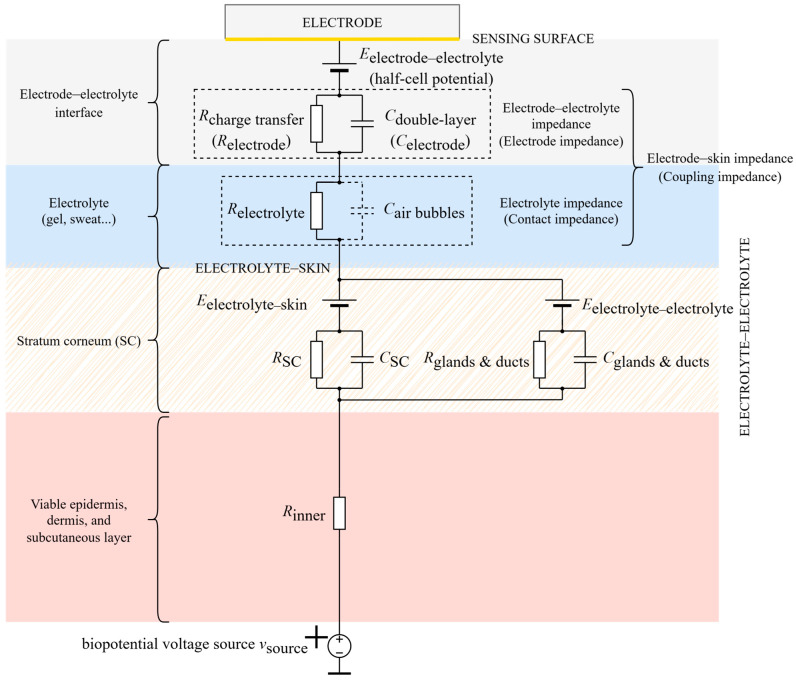
Equivalent lumped-element electrical model of inner body layers, skin, and interface between the electrode and the skin for wet and non-insulated dry surface-contact electrodes, based on [74,111,127,130,158]. Due to safety regulations, the human body is ungrounded, so its floating body potential is denoted by a bar symbol under the *v*_source_ voltage. The model is valid at sub-MHz frequencies. Its detailed description is provided in the following pages.

**Figure A3:**
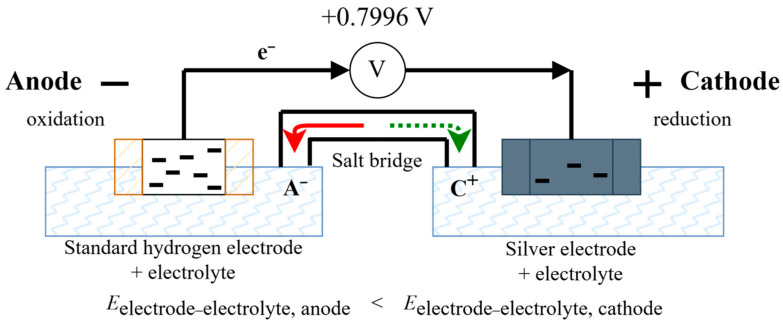
Schematic representation of the electrochemical galvanic cell used to measure the standard reduction potential of a silver cation, Ag^+^. Since the Ag electrode (cathode) exhibits stronger reduction than the standard hydrogen electrode, positive cell voltage is measured. The anode attracts anions, oxidizes, and becomes thinner, whereas the cathode attracts cations, reduces, and becomes thicker. The black clockwise arrow indicates the direction of electrons (e^−^), the red counterclockwise arrow indicates the direction of anions (A^−^), and the green dotted clockwise arrow indicates the direction of cations (C^+^); adapted from [170].

**Surface-contact electrodes.** In Appendix B, surface-contact biopotential electrodes are investigated and described. Appropriately, references [159,160,161,162,163,164,165,166,167,168,169,170,171,172,173,174,175,176,177,178,179,180,181,182,183,184,185,186,187,188,189,190,191,192,193,194,195,196,197,198,199,200,201,202,203,204,205,206,207,208,209,210,211,212,213,214,215,216,217,218,219,220,221,222,223,224,225,226,227,228,229,230,231,232,233,234,235,236,237,238,239,240] and Figure A2 and Figure A3 are introduced. Furthermore, various types of surface-contact electrodes (wet, semi-dry, and dry) are compared, and in addition, their structural design is commented (rigid, flexible, and textile). Although their utilization has long been a standard clinical practice, understanding their theory of operation is a prerequisite for delving into non-contact and capacitive biopotential electrodes. The electrical model of the interface between a surface-contact biopotential electrode and the skin, developed and described therein (Figure A2), is the basis for the model of non-contact electrode–body interfaces, which will be developed and generalized in the following pages of this section. Therefore, the next paragraph, which introduces non-contact and capacitive biopotential electrodes, is written as a continuation of the discussion provided in Appendix B.

**Figure 2:**
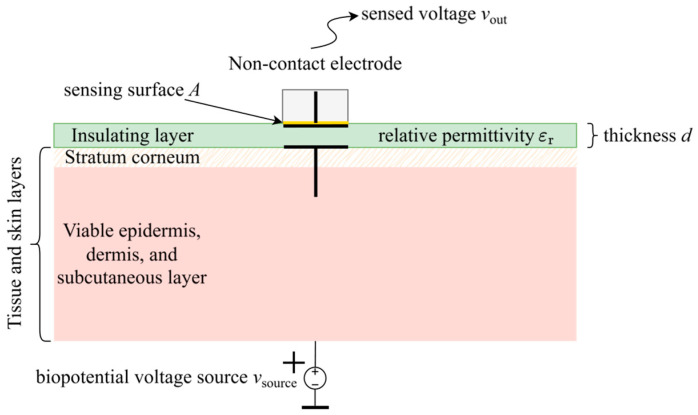
Theoretical principle of an ideal dry non-contact and capacitive electrode created with respect to Figure A2. In the process of dielectric polarization, electric dipoles are displaced under the influence of the applied external electric field. This in turn creates an internal electric field of opposite direction [256,257,258]. Due to safety regulations, the human body is ungrounded, so its floating body potential is denoted by a bar symbol under the *v*_source_ voltage.

**Capacitive coupling, polarization, and displacement currents.** Dry surface-contact electrodes, which concluded Appendix B, are non-insulated and still intended for direct contact with skin, so therein mentioned problems of half-cell and skin potential variation, as well as significantly higher coupling impedance, must be addressed with careful material selection and microstructures, which often implies a sacrifice of comfort and non-invasiveness, impeding their commercialization and rendering the manufacturing process complex and expensive. Following this reasoning, an alternative approach has been drawing more and more attention: the idea of designing non-invasive electrodes that would not require contact with skin at all, and which could instead be applied over clothing or embedded into material, allowing even measurements from a distance and without the subject’s awareness [241]. Dry and non-invasive by definition, such electrodes are called non-contact, contactless, or indirect-contact electrodes. They were first introduced by Richardson in the second half of the 1960s, with papers such as [242], followed by other examples in the early 1970s [243,244,245], later undergoing a renaissance through a series of fundamental quantum physics studies on electric potential sensing (EPS), where non-contact electrodes were used as a more user-friendly alternative to SQUID magnetometers [88,246,247,248,249,250,251,252,253,254]. Contrary to previously described electrodes, which rely on the resistive (ohmic, conductive) method of coupling, the coupling should now be primarily capacitive: the electrode sensing surface and the skin form two plates of a capacitor, whereas the material that separates the electrode from the skin becomes the capacitor dielectric. Dielectric, as a polarizable insulating material [255,256,257], impedes the flow of free charge, and in addition becomes polarized under an externally applied electric field (Figure 2). Hence, the translation of ionic currents in the body into electron currents in the electrical circuit is now based on displacement currents instead [258,259,260,261]. Unlike conduction currents in resistive coupling, which are proportional to the electric field and explained by the physical motion of electrons under electric potential difference, displacement currents are proportional to the rate of change of the electric field in time and represent Maxwell’s correction of Ampère’s law [262,263]. They describe an apparent flow of current through a capacitor, which is in fact a macroscopic manifestation of the displacement of charges stored on the plates: for each electron drawn to one plate of the capacitor, one electron becomes pulled away from the other plate. This way, capacitors resist sudden changes in voltage, counteracting them by creating a flow of charge. As a result, direct current (DC current) flow is blocked, and displacement current *i*_dis_(*t*) exists only as long as either the electric potential difference *v*_coupling_(*t*) across capacitor *C*_coupling_, or *C*_coupling_ itself, changes in time. If a linear time-varying model of a capacitor is assumed [264], this behavior can be expressed mathematically as follows (1):

(1) $$i_{dis}(t)=\frac{dq(t)}{dt}=C_{coupling}\left(t\right)\frac{dv_{coupling}\left(t\right)}{dt}+v_{coupling}\left(t\right)\frac{dC_{coupling}\left(t\right)}{dt}\;,$$

where *dq*(*t*)/*dt* is the rate of apparent charge flow over a given short time interval *dt*. For a *C*_coupling_ assumed to be constant, Assumption 2 can be expressed as follows:

**Assumption** **2.** ***Coupling capacitance.** Unless stated otherwise, C*_coupling_ *is assumed to be linear and constant with time. In addition, a stable environment is assumed without variations in any condition that would change C*_coupling_ *. More on capacitor modeling and its fundamental properties can be found in [264].*

Under Assumption 2, (1) can be simplified to (2):

$$i_{dis}(t)=C_{coupling}\frac{dv_{coupling}\left(t\right)}{dt}\;$$

(2) $$↔\;v_{coupling}\left(t\right)=\frac{1}{C_{coupling}}\int_{-\infty }^{t}i_{dis}\left(\tau \right)d\tau =v_{coupling}\left(t_{0}\right)+\frac{1}{C_{coupling}}\int_{t_{0}}^{t}i_{dis}\left(\tau \right)d\tau \;,t\geq t_{0}\;.$$

In other words, the existence of the displacement current calls for alternating phases of capacitor charging and discharging. On the other hand, integral equation for voltage across the capacitor, *v*_coupling_(*t*), shows that unlike a resistor, a capacitor has memory. This memory property is contained in the initial condition *v*_coupling_(*t*_0_) that represents the voltage present in the chosen initial moment *t*_0_ [264].

**Figure A4:**
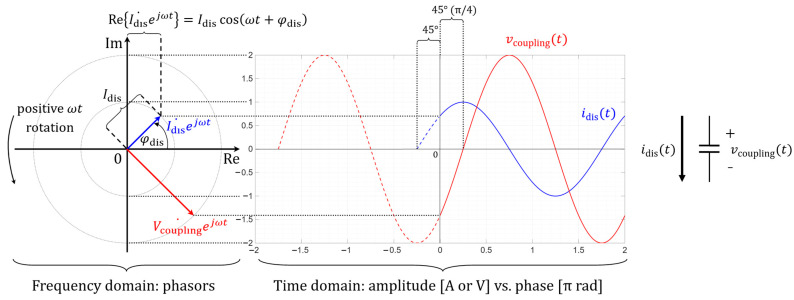
Graphical representation of rotating phasors and their translation into time-domain displacement current *i*_dis_(*t*) and voltage *v*_coupling_(*t*) across a capacitor *C*_coupling_, along with the chosen reference polarity and current direction. The current is leading with the chosen starting phase *φ*_dis_ of +45° (+π/4 rad), whereas the voltage is lagging by 90° (π/2 rad), with the chosen starting phase of −45° (−π/4 rad).

**Fundamentals of capacitors and phasor algebra.** Fundamental considerations on capacitors, as well as fundamentals of phasor algebra and the concept of impedance, are briefly recalled in Appendix C. Also, references [265,266,267,268,269], Equations (A1)–(A5), and Figure A4 are introduced. Therein described properties will be extensively used throughout this study and, in the following paragraphs, employed in the context of coupling with a non-contact biopotential electrode.

**Parallel plate capacitor approximation.** The actual capacitance *C*_coupling_ between two conductive points separated in space is determined by the geometry and electrical properties of the material in between, as well as the material in the surroundings. In the case of non-contact biopotential electrodes, *C*_coupling_ is the equivalent capacitance between the electrode sensing surface and the surface of the skin. The resulting capacitive coupling mechanism is most often approximated with the behavior of a parallel plate capacitor [256,258], as indicated earlier in Figure 2. This approximation assumes two flat plates and negligible fringing fields around their perimeter (3):

(3) $$C_{coupling}=\varepsilon_{0}\varepsilon_{r}\frac{A}{d}\;\;,$$

where *C*_coupling_ is the capacitance of the parallel plate capacitor formed by the body and the electrode sensing surface with an area of *A*. On the other hand, *d* represents the thickness of the material between the plates, i.e., the distance between the electrode and the body. Finally, the extent to which the external electric field is suppressed by the internal electric field, created in the opposite direction by induced charges in the dielectric, is described with permittivity: *ε*_0_ represents the permittivity of free space (vacuum) (8.854 × 10^−12^ F/m), *ε*_r_ represents the relative permittivity of the material that separates the electrode from the skin, and their product *ε*_0_*ε*_r_ represents absolute permittivity. Often, dielectric constant *ϰ* is used (4) as a synonym for *ε*_r_ [270], and the same will be applied throughout this study:

(4) $$\kappa =\frac{{\varepsilon_{0}\varepsilon }_{r}}{\varepsilon_{0}}=\varepsilon_{r}\;.$$

**Other useful approximations.** Other approximations for modeling non-contact and capacitive electrode–body interfaces are also feasible, such as cylindrical capacitor approximation [258,271], but the parallel plate capacitor approximation prevails as by far the most often used method. In fact, approximations for the capacitance of practical geometric configurations and printed circuit board (PCB) structures often stem from the parallel plate capacitor model. Many of these approximations will be used later in this study to model parasitic and stray capacitances. For instance, expressions for capacitance in a coaxial cable, capacitance between two parallel rods, and capacitance of a rod over a conductive plane, can be found in [259]. Further, in [272], capacitances found in several cable configurations are compared. At the PCB design level, additional useful expressions for the capacitance of adjacent PCB copper traces, capacitance between parallel copper planes, and capacitance between a copper trace and an adjacent copper plane can be found in [273]. Finally, calculations for the capacitance of a PCB transmission line in microstrip and stripline configuration can be found in [259,273,274]. The rest of the analysis will use the parallel plate approximation (3), and the employed simplifications will be summarized in the assumption at the end of this section.

**Model accuracy, fringing fields, and finite models.** Equation (3) revealed that the parallel plate capacitor approximation can be used as a tool for qualitatively relating the electrode–body capacitance *C*_coupling_ to electrode sensing area *A*, as well as to thickness *d* and dielectric constant *ε*_r_ of the material inserted between the body and the electrode. In reality, direct proportionality of capacitance to the parallel plate area is an oversimplification because of real-world dielectric material properties and additional layout parasitic capacitance, which will be further discussed in Section 4.1. As a rule of thumb, the greater the ratio $\sqrt{A}$/*d* (i.e., the wider the plates and the smaller the distance between the plates), the more accurate the chosen parallel plate approximation [102,259]. Ideally, the electric field that arises within the equivalent parallel plate capacitor as a result of charge accumulated on the surface of its plates is predominantly uniform and perpendicular to the electrode–body interface. This is in accordance with Gauss’s law for electric fields (first Maxwell’s equation) [258,262]. However, in reality, fringing electric fields that spread into the surroundings from the edges of the electrode–body interface will contribute to additional capacitance that is not accounted for in the parallel plate capacitor model. The contribution of these fringing fields becomes especially evident as *d* is increased [259]. For this reason, when it comes to using electrodes that are physically separated from the body via an air gap (*ε*_r,air_ *≈* 1), validity of the parallel plate approximation is limited to very small *d* up to about the order of 1 mm [9]. Later in this section, this will prove to be an important limiting factor in the employment of off-body biopotential electrodes. More on electric field calculation can be found in [255,258,275,276], and accompanying analysis of finite parallel plate capacitor models that account for edge effects of electric field distribution can be found in [15,277,278,279,280,281]. Likewise, if the space around the parallel plate capacitor were not uniformly filled with the material of same properties, as assumed here, accurate modeling would require accounting for partial dielectric filling and using the effective dielectric constant for describing the dielectric properties of inhomogeneous material (see [259] and subchapter 7.3 in [257]).

**Figure 3:**
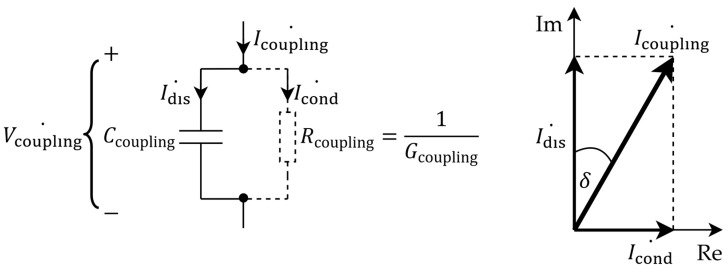
Equivalent electrical circuit of a capacitor *C*_coupling_, along with voltage and current phasors and the chosen reference polarity and current direction. Leakage resistance *R*_coupling_ can also be modeled as conductance *G*_coupling_. Dielectric loss is described by the imaginary part of the permittivity, *ε*″ = *ε*_0_*ε*_r_″, and loss tangent tan*δ*, based on [256,257,260,270,283].

**Dielectric constant and dielectric loss.** The dielectric constant of the dielectric between the plates of the capacitor is an intrinsic macroscopic property of the material. It is roughly related to the size and number of electric dipoles and describes the magnitude of the polarization effect, as well as the extent to which the light is slowed down in the dielectric [259]. Just like electrical properties, such as conductivity *σ* and its reciprocal, resistivity ρ, the dielectric constant also depends on various parameters—from ambient-induced conditions (e.g., temperature and moisture content) to manufacturing process and material structure (surface roughness, material purity and homogeneity). Furthermore, it may also depend on the strength of the electric field and the applied voltage, which is an issue known as the DC bias effect, typical for high-*κ* class 2 and class 3 ceramic capacitors, with a dielectric constant that can reach the order of 1000 (see subchapter 1x.3 in [282]). Additionally, the dielectric constant may also depend on the direction of the electric field, which would require treating it as a tensor [257,276]. Finally, when discussing dielectric properties, there is also the aspect of alternating voltage. Namely, unlike conductors, in which the power dissipation is proportional to the square of the applied voltage, electrical energy transformation into heat in dielectrics displays additional dependence on the frequency. Among all mechanisms of these dielectric losses, the simplest and most often considered in electrical circuits is the dielectric loss conditioned by conductivity (see subchapter 7.8 in [257], and more specifically, [283]). Higher frequencies increase the motion of the dipoles, thereby increasing the conductivity that arises from collisions and scattering of charge carriers, as well as from the heat generated by the friction of increased dipole motion. To model these dielectric losses, complex number formalism is used: imaginary part *ε*_r_″ is added, and the so far observed dielectric constant *ε*_r_ becomes the real part *ε*_r_′. The total permittivity can therefore be observed as a complex number ε* = *ε*′ − *jε*″ = *ε*_0_*ε*_r_′ − *jε*_0_*ε*_r_″ = *ε*_0_*ε*_r_ − *jε*_0_*ε*_r_″. To describe how lossy the dielectric material is, the tangent of the dielectric loss angle tan*δ* is used. It is also called the loss tangent or dissipation factor, and it is roughly related to the number of dipoles and their freedom of movement in the applied alternating current (AC) electric field [283]. Also, its reciprocal, the quality factor *Q* of the dielectric material, can be obtained [256,270]. Specifically, dielectric loss conditioned by conductivity manifests itself in an additional conduction or active current *i*_cond_(*t*) that is in phase with the applied sinusoidal voltage *v*_coupling_(*t*) (Figure 3). In that sense, tan*δ* indicates the extent to which the total current phasor $\dot{I_{coupling}}$ deviates from the desired, purely reactive displacement current phasor, $\dot{I_{dis}}$, and so, lower tan*δ* means lower dielectric loss. $\dot{I_{dis}}$ can be observed through the capacitive reactance *X*_Ccoupling_ (A4), whereas the additional conduction current phasor $\dot{I_{cond}}$ can be perceived through an equivalent resistive leakage component *R*_coupling_. Applying alternating sinusoidal electric field and parallel plate approximation as in (3) yields (5):

$$tan\delta =\frac{\dot{I_{cond}}}{\dot{I_{dis}}}=\frac{\frac{\dot{V_{coupling}}}{R_{coupling}}}{\frac{\dot{V_{coupling}}}{X_{\mathrm{Ccoupling}}(\omega )}}=\frac{X_{\mathrm{Ccoupling}}(\omega )}{R_{coupling}}=\frac{1}{R_{coupling}\cdot \omega C_{coupling}}$$

(5) $$=\frac{1}{\frac{1}{\sigma }\frac{d}{A}\cdot \omega \varepsilon_{0}\varepsilon_{r}\frac{A}{d}}=\frac{\frac{\sigma }{\omega }}{\varepsilon_{0}\varepsilon_{r}}=\frac{\varepsilon^{\prime \prime }}{\varepsilon_{0}\varepsilon_{r}}=\frac{\varepsilon_{0}{\varepsilon_{r}}^{\prime \prime }}{\varepsilon_{0}{\varepsilon_{r}}^{\prime }}=\frac{Im\left\{\varepsilon^{*}\right\}}{Re\left\{\varepsilon^{*}\right\}}\approx \frac{1}{Q}=\frac{1}{2\pi }\frac{energy\;dissipated\;per\;cycle}{energy\;stored\;per\;cycle},$$

where *σ* is the bulk or volume conductivity of the dielectric material. As a rule of thumb for polymers, a tighter confinement of dipoles inside the structure will result in a smaller real part of relative permittivity *ε*_r_′ and lower tan*δ* [283]. The existence of dielectric losses explains why additional precaution must be taken at higher frequencies, e.g., when wearable antennae design is considered [134,135,136,229]. Aside from introducing the dielectric loss, the frequency of the applied alternating electric field will also contribute to characterizing the mechanisms of dielectric polarization, as described in [256,257,260], and specifically in [284], concerning triboelectric effect. The existence of various polarization mechanisms is tightly related to frequency-dependent changes in *ε*_r_* called dielectric dispersion and described with dielectric spectroscopy. Similar observations are applicable to dielectric properties of biological tissues as well [56,75]. Lastly, depending on the physical mechanisms of dielectric loss under given conditions, frequency-dependent variations may also be visible in tan*δ* itself due to, e.g., dipole inertia. For more details on all these phenomena, refer to subchapters 7.1, 7.3, and 7.8 in [257], and subchapter 9.5 in [283]. Assuming that tan*δ* = $\frac{\sigma }{\varepsilon_{0}\varepsilon_{r}\omega }$ is fairly constant means that conductivity *σ* is strongly frequency-dependent. After summarizing the observations in the following Assumption 3, this will be further investigated in the context of the insulating layer between the body and a non-contact electrode. Afterward, the analysis will be extended to the generalized electrical model of the electrode–body interface.

**Assumption** **3.** ***Insulating layers and dielectric properties.** In this paper, the terms “dielectric”, “dielectric layer”, and “insulating layer” are used interchangeably. The real part ε*_r_′ *of the complex relative permittivity, ε*_r_* *= ε*_r_′ − *jε*_r_*″, will be considered equal to the dielectric constant, and denoted simply by ε*_r_′ *= ε*_r_*, whereas the imaginary part will be denoted by ε*_r_*″. For the purpose of accompanying the model with calculations that can allow for a qualitative analysis at frequencies of interest (refer to Assumption 1 for details), linear, isotropic, and homogeneous dielectrics [255,257,285] with uniform cross-sectional areas will be assumed. In other words, it is assumed that insulating layers have a uniform structure with properties equal in all directions and that the superposition principle is applicable in the case of multiple sources of electromagnetic fields. Therefore, ε*_r_′* = ε*_r_* will be treated as a scalar. In accordance with Assumption 2, the influence of ambient conditions on ε*_r_* *and* tan*δ will not be considered. Similarly, practical aspects of manufacturing processes, structural imperfections, and aging effect in the material will be neglected. Finally, assuming that dielectric loss conditioned by conductivity is the predominant mechanism of dielectric losses at frequencies of interest (refer to Assumption 1), any additional possible variations in ε*_r_* *and* tan*δ with frequency are neglected. Thus, at frequencies of interest, ε*_r_′* = ε*_r_ *is approximated with the quasi-static dielectric constant that would be measured in near-DC or constant electric fields. Based on these assumptions, the following analysis regarding the leakage resistance as a function of frequency is performed in accordance with [283].*

**Dielectric leakage resistance and insulator breakdown.** In the case of wet and non-insulated dry surface-contact electrodes described in Appendix B, imperfections of resistive coupling were modeled with parallel capacitances that represented the unwanted dielectric properties in each conductive layer (Figure A2). Now, from the perspective of non-contact and capacitive electrodes, the imperfections of capacitive coupling achieved via an insulating layer are modeled with a leakage or insulation resistance *R*_coupling_ that shunts the desired coupling capacitance *C*_coupling_, giving rise to resistive coupling (Figure 3). In other words, the equivalent impedance of a dielectric can be modeled as *Z*_coupling_ = (−*jX*_Ccoupling_)||*R*_coupling_ (A4). On the one hand, this parallel leakage component *R*_coupling_ accounts for the finite intrinsic conduction of the insulating material due to impurities and defects in its structure, as well as for the current from the motion of mobile charge carriers such as ions if present (see chapter 3 in [260] and subchapter 9.4 in [283]). This leakage usually increases with the applied voltage until the dielectric strength or the breakdown field is reached (see [255,256,275,286] and subchapter 7.10 in [257]). However, aside from this finite DC leakage at 0 Hz, *R*_coupling,DC_, there is also an additional frequency-dependent leakage arising from the dielectric loss and the finite conduction current $\dot{I_{cond}}$. In accordance with the parallel plate capacitor approximation (3), this AC-leakage resistance *R*_coupling,AC_(*ω*) can be expressed as a function of geometric configuration and frequency (6) [283,287,288]:

(6) $$R_{coupling,AC}\left(\omega \right)=\frac{\dot{V_{coupling}}}{\dot{I_{cond}}}=\frac{\dot{V_{coupling}}}{\frac{\dot{V_{coupling}}}{\frac{1}{j\omega \varepsilon_{0}\left(-j{\varepsilon_{r}}^{\prime \prime }\right)\frac{A}{d}}}}=\frac{1}{\omega {{\varepsilon_{0}\varepsilon }_{r}}^{\prime \prime }}\frac{d}{A}=\frac{1}{\omega {\varepsilon_{0}\varepsilon }_{r}tan\delta }\frac{d}{A}=\frac{1}{\sigma_{AC}(\omega )}\frac{d}{A}\;,$$

where *σ*_AC_(*ω*) is the bulk or volume AC conductivity of the dielectric material. In other words, the dielectric constant *ε*_r_ as the real part of *ε*_r_*, along with $\dot{I_{disp}}$, determines the reactive, capacitive portion *X*_Ccoupling_(*ω*) (3,A4) of the total *Z*_coupling_, whereas the imaginary part of *ε*_r_*, along with $\dot{I_{cond}}$, determines the resistive portion *R*_coupling,AC_(*ω*) (6) of the total *Z*_coupling_. Both this capacitive and this resistive component are frequency-dependent. Following Assumption 3 and the analysis from subchapters 9.4–9.6 in [283], the *R*_coupling,AC_(*ω*) component does not emerge abruptly above 0 Hz, but rather appears above a certain *ω*_δ_ angular frequency. Thus, the total shunt leakage resistance *R*_coupling_(*ω*) can be treated as a DC leakage resistance *R*_coupling,DC_ (7):

(7) $$R_{coupling,DC}=\frac{1}{\sigma_{DC}}\frac{d}{A}=\rho_{DC}\frac{d}{A}\;\;\;for\;\omega <\omega_{\delta }\;,$$

until reaching *ω*_δ_, above which it decreases with frequency (8):

$$R_{coupling,AC}\left(\omega \right)=\frac{1}{\sigma_{DC}+\sigma_{AC}(\omega )}\frac{d}{A}=\frac{1}{\frac{1}{\rho_{DC}}+(\omega -\omega_{\delta }){\varepsilon_{0}\varepsilon }_{r}tan\delta }\frac{d}{A}$$

(8) $$=\frac{R_{coupling,DC}}{1+{\varepsilon_{0}\varepsilon }_{r}\rho_{DC}tan\delta (\omega -\omega_{\delta })}\;\;\;for\;\omega \geq \omega_{\delta },$$

where ρ_DC_ is the bulk DC resistivity of the dielectric material. Now, the previous statement on the frequency-dependence of dielectric losses becomes clearer: the increase in frequency of the applied voltage and the generated electric field translates into a faster back-and-forth rotation of electric dipoles, which increases the bulk conductivity and decreases the bulk resistivity. The larger the tan*δ*, the lossier the dielectric and the larger the steepness of that decrease [283]. Substituting further the parallel plate approximations (3) and (7) into (8) finally yields the piecewise first-order equation for the total shunt leakage resistance of the dielectric, *R*_coupling_(*ω*) (9):

(9) $$R_{coupling}(\omega )=\left\{\begin{array}{cc} \;\;\;\;\;R_{coupling,DC}=\rho_{DC}\frac{d}{A}, & \omega <\omega_{\delta }\\ \frac{R_{coupling,DC}}{1+R_{coupling,DC}C_{coupling}tan\delta (\omega -\omega_{\delta })}, & \omega \geq \omega_{\delta } \end{array}\right..$$

The letter *R* for leakage resistance is consistently used, despite its frequency-dependence, to stress the distinction between the leakage resistance *R*_coupling_(*ω*) on the one side and the corresponding *Z*_Ccoupling_ = −*jX*_Ccoupling_, along with the total *Z*_coupling_ = (−*jX*_Ccoupling_)||*R*_coupling_(*ω*), on the other side.

**Assessment of the predominant coupling mechanism.** In the preceding discussion, the dielectric between the capacitor plates (surface of the skin on the one side, and electrode sensing surface on the other side) was modeled as an *R*_coupling_(*ω*)*C*_coupling_-parallel based on parallel plate approximations (3,9). Therein, the existence of the finite leakage component *R*_coupling_(*ω*) revealed that the coupling over a dielectric layer is seldom solely capacitive, and even if it is predominantly capacitive, it is not necessarily so across the entire frequency range. Therefore, Test 1 for evaluating the predominant coupling mechanism over a dielectric can be devised:

**Test** **1.** *For a particular insulating layer, if across a certain frequency range:*

$$R_{\mathrm{coupling}}\left(\omega \right)\gg X_{\mathrm{Ccoupling}}\left(\omega \right)\to \;R_{\mathrm{coupling}}\left(\omega \right)\gg \frac{1}{\omega C_{\mathrm{coupling}}}\;,$$

*i.e.,*

$$\;R_{\mathrm{coupling}}\left(\omega \right)\geq \frac{10}{\omega C_{\mathrm{coupling}}}\;or\;\omega \geq \frac{10}{R_{\mathrm{coupling}}{\left(\omega \right)C}_{\mathrm{coupling}}}\;,$$

*then the predominant coupling mechanism at these frequencies is capacitive.*

*Conversely, if across a certain frequency range:*

$$R_{\mathrm{coupling}}\left(\omega \right)\ll X_{\mathrm{Ccoupling}}\left(\omega \right)\;\to \;R_{\mathrm{coupling}}\left(\omega \right)\ll \frac{1}{\omega C_{\mathrm{coupling}}}\;,$$

*i.e.,*

$$\;R_{\mathrm{coupling}}\left(\omega \right)\leq \frac{1}{10}\frac{1}{\omega C_{\mathrm{coupling}}}\;or\;\omega \leq \frac{1}{10}\frac{1}{R_{\mathrm{coupling}}{\left(\omega \right)C}_{\mathrm{coupling}}}\;,$$

*then the predominant coupling mechanism at these frequencies is resistive (ohmic, conductive).*

*The corresponding frequency f is further calculated from ω with the use of relation (A2).*

**Figure 4:**
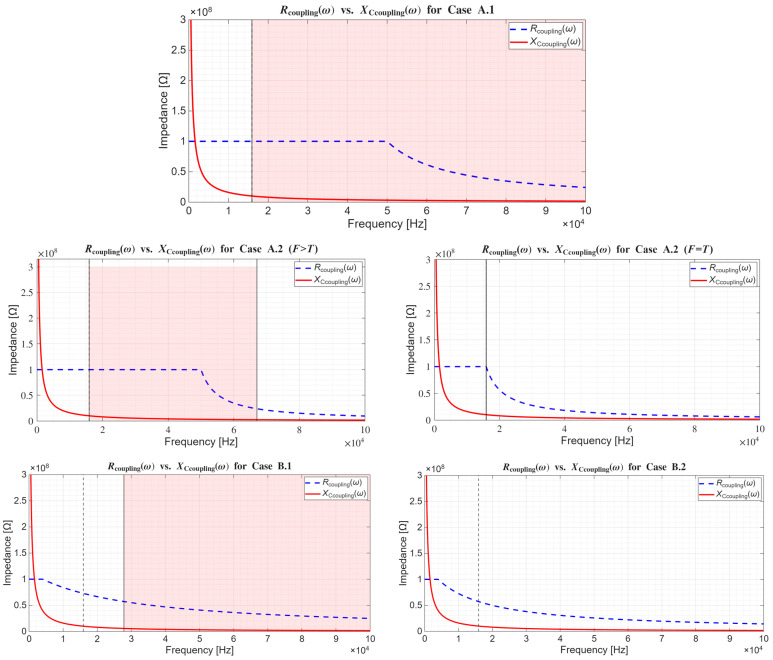
Examples of (dashed blue) *R*_coupling_(*ω*) and (solid red) *X*_Ccoupling_(*ω*) curves with respect to frequency *f* produced in MathWorks^®^ MATLAB R2025b environment [146] and displayed up to 100 kHz for each of the four subcases from Table 1: A.1, A.2 when *F* > *T*, A.2 when *F* = *T*, B.1, and B.2, respectively (*T* = 10). Frequency limits of predominantly capacitive coupling areas are denoted by vertical solid black lines. Whenever only the low-side frequency limit is displayed, that indicates that the high-side limit does not exist under the conditions considered. The predominantly capacitive coupling areas are marked with shaded red areas. In addition, a vertical dashed black line denotes the angular frequency $\frac{T}{R_{coupling,DC}C_{coupling}}$ = $\frac{10}{R_{coupling,DC}C_{coupling}}$. In case A, these dashed lines coincide with the solid-line low-side frequency limits. Specifically, when *F* = *T* in subcase A.2, the predominantly capacitive area is reduced to a single angular frequency equal to $\frac{T}{R_{coupling,DC}C_{coupling}}$ = $\frac{10}{R_{coupling,DC}C_{coupling}}$.

Two important consequences can be noted. Firstly, if a wet surface-contact electrode with its typical analog of *R*_coupling_ up to the order of 10 kΩ and a typical analog of *C*_coupling_ in the order of 10 nF is considered, Test 1 corroborates that the achieved coupling is predominantly resistive, at least up to frequencies in the order of 1 kHz. Interestingly, it can be noted that their *C*_coupling_ in the order of 10 nF is in fact a value higher than most *C*_coupling_ values achieved with predominantly capacitive dry electrodes. In other words, rather than the absolute value of *C*_coupling_, it is the relative value of *X*_Ccoupling_(*ω*) with respect to *R*_coupling_(*ω*) that matters. This can be extended further to the second important consequence: whenever a dielectric exhibits a finite amount of leakage (*R*_coupling_ < ∞), there exists a frequency up to which the resistive coupling predominates. Accordingly, a frequency at which the coupling mechanism transitions from predominantly resistive to predominantly capacitive can be defined. Specifically, since *R*_coupling_(*ω*) = *R*_coupling,DC_ at *ω* ≤ *ω*_δ_, this angular frequency of transition is equal to $\frac{10}{R_{coupling,DC}C_{coupling}}$ whenever *ω*_δ_ ≥ $\frac{10}{R_{coupling,DC}C_{coupling}}$. This indicates that the bandwidth, band, frequency range, or area of predominantly capacitive coupling can be further explored based on the position of *ω*_δ_ with respect to $\frac{10}{R_{coupling,DC}C_{coupling}}$. To generalize the investigation further and explore the influence of the difference between *ω*_δ_ and $\frac{10}{R_{coupling,DC}C_{coupling}}$, *ω*_δ_ can be expressed as a portion (or a multiple) of $\frac{1}{R_{coupling,DC}C_{coupling}}$ with the use of a positive real factor *F*. Accordingly, notation *ω*_δ_ = $\frac{F}{R_{coupling,DC}C_{coupling}}$ can be used. Moreover, the factor of 10 that substitutes the “>>” and “<<” conditions of “at least an order of difference” can also be generalized to a positive real factor of *T* ≥ 10 to investigate the influence of the stringency of Test 1. This way, the area of predominantly capacitive coupling would be defined by the condition $R_{coupling}(\omega )\geq {T\cdot X}_{Ccoupling}(\omega )\geq {10X}_{Ccoupling}(\omega )$. Based on this criterion, the area of predominantly capacitive coupling can be assessed by observing the *R*_coupling_(*ω*) and *X*_Ccoupling_(*ω*) curves. This finally leads to results in Table 1, which are accompanied by visual examples in Figure 4.

The results are as expected: the angular frequency at which the coupling becomes predominantly capacitive is equal to $\frac{T}{R_{coupling,DC}C_{coupling}}$ whenever *ω*_δ_ ≥ $\frac{T}{R_{coupling,DC}C_{coupling}}$, and it can only be higher than *ω*_δ_ whenever *ω*_δ_ < $\frac{T}{R_{coupling,DC}C_{coupling}}$. If the upper angular frequency limit to the area of predominantly capacitive coupling exists, it is situated at *ω* ≥ *ω*_δ_. To increase the upper frequency limit and reduce the lower frequency limit, i.e., broaden the area of predominantly capacitive coupling, several measures can be taken:

- Increasing *C*_coupling_ (reactance reduction (A4));
- Increasing *R*_coupling,DC_ (leakage reduction (9));
- Reducing tan*δ* (slowing down the leakage increase (9));
- Increasing factor *F* and consequently *ω*_δ_ (delaying the leakage increase (9));
- Decreasing *T* (relaxing the criterion for assessing the frequency at which the coupling becomes predominantly capacitive (Table 1)).

Naturally, the presented cases are more of a generalized mathematical construct rather than physically meaningful possibilities. In practice, tan*δ* of wearable fabrics is largely below 0.1 [134,135,229], and the dielectric loss of used material is not of concern before reaching the frequencies in the order of 1 MHz or higher, which are used, e.g., for capacitive power transfer [76,77,279,280,289], intrabody communication [75], and wearable antennae design [134,135,136,229]. These practical aspects narrow the cases from Table 1 down to subcase A.1, with *ω*_δ_ outside of the frequency range of interest (Assumption 1). Therefore, in the rest of this paper, only the *R*_coupling,DC_ component of the leakage resistance *R*_coupling_(*ω*) (9) will be considered. In other words, parallel plate approximations (3,7) will be used. Accordingly, simplified notation *R*_coupling,DC_ = *R*_coupling_ and *ρ*_DC_ = *ρ* will be used. Also, test factor *T* = 10 will continue to be used for “>>” and “<<” conditions. This will be summarized in Assumption 4 at the end of this section.

**Figure 5:**
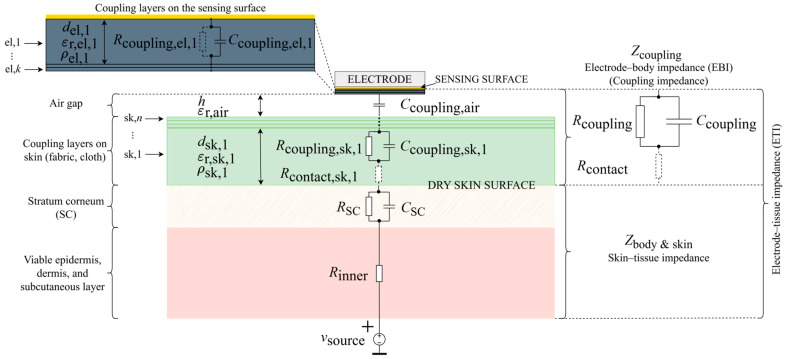
Generalized equivalent lumped-element electrical model of the interface between the body and a non-contact and insulated biopotential electrode, based on Figure 2. Just like the previous model for wet and non-insulated dry surface-contact biopotential electrodes (Figure A2), this model is adequate up to frequencies in the order of 1 MHz [160]. It comprises inner body layers, skin, and interface with a generalized dry biopotential electrode. The electrode is applied over *n* coupling layers that cover the skin, further separated from the skin with a layer of air with thickness *h*, and additionally coated with *k* coupling layers. The total number of coupling layers is therefore *n*+1+*k*. Resistances *R*_coupling,el,index_ and *R*_contact,sk,index_ are marked with dashed lines to indicate that they are sometimes omitted in the literature. In this paper, only *R*_contact,sk,index_ resistances will be neglected. Also, idealized dry skin is assumed, hence, with respect to Figure A2, components that model perspiration, electrodermal response, and electrochemical reaction with electrolytes (*E*_electrolyte–skin_, *R*_glands & ducts_, *C*_glands & ducts_, and *E*_electrolyte–electrolyte_) are neglected [158]. As described later in the text, the equivalent skin–tissue impedance *Z*_body & skin_ and the equivalent coupling (electrode–body) impedance *Z*_coupling_ create the total equivalent electrode–tissue impedance. Finally, as before, the human body is ungrounded due to safety regulations, so its floating body potential is denoted by a bar symbol under the *v*_source_ voltage. The model is based on [74,87,111,130], and its detailed description is provided in the following pages.

**Multiple insulating or coupling layers, equivalent coupling capacitance, and equivalent coupling resistance.** So far, a single insulating layer has been analyzed. In reality, there could be more than just one insulating layer: the non-contact interface between the body and the electrode can be achieved by means of fabric layers that cover the skin, an air gap that separates the electrode from the body, and insulating layers coated on the conductive sensing surface of the electrode. With respect to their equivalent capacitive and resistive components, each of these layers can be more generally referred to as a coupling layer. Such a generalized case of a non-contact and insulated electrode–body interface is given in Figure 5. Therein, “sk,index” is the “layer” notation used for referring to one of the *n* coupling layers placed on the surface of the skin (for each “index” from 1 to *n*), whereas “el,index” is the “layer” notation used for referring to one of the *k* coupling layers coated on the electrode sensing surface (for each “index” from 1 to *k*). Following this naming convention, the bottommost coupling layer placed directly on the surface of the skin is layer sk,1 with a thickness *d*_sk,1_, dielectric constant *ε*_r,sk,1_, and bulk DC resistivity *ρ*_DC,sk,1_ = *ρ*_sk,1_, whereas the topmost coupling layer coated directly on the electrode sensing surface is layer el,1 with a thickness *d*_el,1_, dielectric constant *ε*_r,el,1_, and bulk DC resistivity *ρ*_DC,el,1_ = *ρ*_el,1_. The air gap is then denoted by thickness *h* and dielectric constant *ε*_r,air_. In accordance with the parallel plate approximations for subcase A.1 from Table 1 (3,7), these multiple coupling layers stacked on top of each other can be treated as a series of capacitors *C*_coupling,layer_ and their shunt leakage resistances *R*_coupling,DC,layer_ = *R*_coupling,layer_. Herein, the notation “layer” in subscripts can be replaced by “sk,index”, “air”, and “el,index”. The equivalent *C*_coupling_ of the entire stackup can then be calculated as follows (10) [258]:

$$\frac{1}{C_{coupling}}=\sum \frac{1}{C_{coupling,layer}}$$

$$=\frac{1}{C_{coupling,sk,1}}+\cdots +\frac{1}{C_{coupling,sk,n}}+\frac{1}{C_{coupling,air}}+\frac{1}{C_{coupling,el,k}}+\cdots +\frac{1}{C_{coupling,el,1}}$$

$$=\frac{1}{\varepsilon_{0}\varepsilon_{r,sk,1}\frac{A}{d_{sk,1}}}+\cdots +\frac{1}{\varepsilon_{0}\varepsilon_{r,sk,n}\frac{A}{d_{sk,n}}}+\frac{1}{\varepsilon_{0}\varepsilon_{r,air}\frac{A}{h}}+\frac{1}{\varepsilon_{0}\varepsilon_{r,el,k}\frac{A}{d_{el,k}}}+\cdots +\frac{1}{\varepsilon_{0}\varepsilon_{r,el,1}\frac{A}{d_{el,1}}}$$

(10) $$=\frac{\sum_{i=1}^{n}\frac{d_{sk,i}}{\varepsilon_{r,sk,i}}+\frac{h}{\varepsilon_{r,air}}+\sum_{j=0}^{k-1}\frac{d_{el,k-j}}{\varepsilon_{r,el,k-j}}}{\varepsilon_{0}A}\;.$$

Conveniently, the equivalent dielectric constant *ε*_r_ of the equivalent series capacitance *C*_coupling_ can be calculated; *ε*_r_ (11) follows directly from the equivalence based on (10):

$$\frac{1}{C_{coupling}}=\frac{\sum_{i=1}^{n}\frac{d_{sk,i}}{\varepsilon_{r,sk,i}}+\frac{h}{\varepsilon_{r,air}}+\sum_{j=0}^{k-1}\frac{d_{el,k-j}}{\varepsilon_{r,el,k-j}}}{\varepsilon_{0}A}=\frac{1}{\varepsilon_{0}\varepsilon_{r}\frac{A}{\sum_{i=1}^{n}d_{sk,i}+h+\sum_{j=0}^{k-1}d_{el,k-j}}}$$

(11) $$\to \varepsilon_{r}=\frac{\sum_{i=1}^{n}d_{sk,i}+h+\sum_{j=0}^{k-1}d_{el,k-j}}{\sum_{i=1}^{n}\frac{d_{sk,i}}{\varepsilon_{r,sk,i}}+\frac{h}{\varepsilon_{r,air}}+\sum_{j=0}^{k-1}\frac{d_{el,k-j}}{\varepsilon_{r,el,k-j}}}\;.$$

Other methods for estimating the equivalent dielectric constant *ε*_r_ can be found in the literature, such as the weighted average often used in PCB manufacturing [290].

Similarly, equivalent series resistance *R*_coupling_ (12), along with the equivalent bulk DC resistivity ρ of the entire stackup (13), can be calculated based on (7). In accordance with the results from Table 1, only *R*_coupling,DC,layer_ components are considered (subcase A.1), and, for simplicity’s sake, they are denoted by *R*_coupling,layer_. Similarly, ρ_DC,layer_ components are denoted by ρ_layer_:

$$R_{coupling}=\sum R_{coupling,layer}\left(\omega \right)=\sum R_{coupling,DC,layer}=\sum R_{coupling,layer}$$

$$=R_{coupling,sk,1}+\cdots +R_{coupling,sk,n}+R_{coupling,air}+R_{coupling,el,k}+\cdots +R_{coupling,el,1}$$

$$=\rho_{sk,1}\frac{d_{sk,1}}{A}+\cdots +\rho_{sk,n}\frac{d_{sk,n}}{A}+R_{coupling,air}+\rho_{el,k}\frac{d_{el,k}}{A}+\cdots +\rho_{el,1}\frac{d_{el,1}}{A}$$

(12) $$=\frac{\sum_{i=1}^{n}\rho_{sk,i}d_{sk,i}+R_{coupling,air}+\sum_{j=0}^{k-1}\rho_{el,k-j}d_{el,k-j}}{A}\;,$$

$$\frac{\sum_{i=1}^{n}\rho_{sk,i}d_{sk,i}+R_{coupling,air}+\sum_{j=0}^{k-1}\rho_{el,k-j}d_{el,k-j}}{A}=\rho \frac{\sum_{i=1}^{n}d_{sk,i}+h+\sum_{j=0}^{k-1}d_{el,k-j}}{A}$$

(13) $$\to \rho =\frac{\sum_{i=1}^{n}\rho_{sk,i}d_{sk,i}+R_{coupling,air}+\sum_{j=0}^{k-1}\rho_{el,k-j}d_{el,k-j}}{\sum_{i=1}^{n}d_{sk,i}+h+\sum_{j=0}^{k-1}d_{el,k-j}}\;.$$

Naturally, very high leakage resistance of the air gap layer, *R*_coupling,air_, cannot be accurately described with the parallel plate approximation (7); also, transfer of electrons through the air can occur in the event of electrical discharge (refer to Figure 6 in [275], and to [284,291]). Hence, equations for equivalent *R*_coupling_ (12) and *ρ* (13) can be used whenever *h* = 0.

**Non-contact versus capacitive.** Now that the electrical model of a single insulating (coupling) layer has been defined (Figure 3) and expanded into the stackup model of the electrode–body interface (Figure 5), the abstracted interface can be further adapted for various types of non-contact and capacitive biopotential electrodes. In that process, care must be taken: not all non-contact electrodes are necessarily capacitive, and not all capacitive electrodes are necessarily non-contact. This is often a source of inconsistencies and misunderstandings in the literature, which presented the motivation for this paper in the first place [96,97,98,99]. Three different cases can be observed: insulated, off-body, and on-body electrodes.

**Insulated electrodes.** Especially in the early days of non-contact electrodes [242,243,244,245], high coupling capacitance was achieved by coating the sensing surface of a non-insulated dry surface-contact electrode with a few mils thin layer of high dielectric constant (for instance, metal oxides or barium-titanate ceramics). That way, *C*_coupling,el,1_ in the order of 1 nF and a very weak resistive contact *R*_coupling,el,1_ higher than 1 TΩ was achieved in series with the stratum corneum impedance [98]. With these values of *C*_coupling_ ≈ *C*_coupling,el,1_ and *R*_coupling_ ≈ *R*_coupling,el,1_, predominantly capacitive coupling could have been achieved already at frequencies in the order of 1 mHz ($\frac{10}{{2\pi R}_{coupling}C_{coupling}}$, refer to Test 1). Concomitantly, given the high values of the leakage resistance, the coupling layer indeed served as an electrically insulating layer, and the resistive component *R*_coupling,el,1_ was often omitted in the literature (e.g., Figure 1 in [87]). Since this type of dry electrode exhibits both a high coupling capacitance and a high leakage resistance with the use of an insulating layer coated on the sensing surface, it is more specifically called an insulated or insulating electrode, and often regarded as capacitive even when it is surface-contact, i.e., placed directly on the skin. Moreover, there are even rare examples of immersing insulated electrodes in the electrolytic paste [245]. However, it is safer to treat such cases rather as poor wet surface-contact interfaces. In fact, this is advisable whenever there is a possibility of electrochemical reaction between the electrode sensing surface material and an electrolyte solution such as sweat, as turned out to be the case when anodic aluminum oxide coating was used [242,243,244,245]. To conclude, the case of an insulated electrode, either surface-contact or non-contact, can be considered whenever at least one “el,index” coupling layer is present in Figure 5.

**Off-body electrodes.** Aside from being insulated or non-insulated, electrodes could also be physically separated and isolated from the body, thereby completely removing both galvanic and mechanical contact [292]. Unlike the on-body electrodes described so far, these electrodes are off-body. In that case, naturally, electrodes are non-contact even if they are non-insulated, and even if the skin is exposed and uncovered. Therefore, this case of off-body electrodes is considered whenever an air gap exists in Figure 5. Detaching the electrode from the body and placing it to a height *h* > 0 adds the parallel plate capacitor *C*_coupling,air_, which represents the created air gap. Since *R*_coupling_ in (12) is now theoretically approaching infinity due to the very high *R*_coupling,air_, off-body electrodes draw virtually no current from the body and, therefore, achieve the purest capacitive coupling that truly operates on small displacement currents only. This entirely capacitive sensing mechanism is based on electric potential sensing (EPS) [9], which expands the utilization of non-contact biopotential electrodes to a more generalized concept of electric potential probes, displacement current sensing, and electric field detectors [88,248,253,254,293]. With recently developed off-body electrodes, researchers carried out biopotential measurements even at a distance of several meters [294]. However, although creation of an air gap may ensure pure capacitive coupling, the dielectric constant of the air layer, *ε*_r,air_, is only about 1, and hence, inclusion of a low capacitance *C*_coupling,air_ in series drastically reduces the total equivalent capacitance *C*_coupling_ (10). For instance, using a 1 cm^2^ sensing area electrode at a distance of only 1 mm from the body reduces *C*_coupling_ to the values of about 1 pF at best (3). Therefore, as concluded in [96,98], firm mechanical contact between the electrode and the body, established without an air gap, is preferred—i.e., on-body electrodes. The same was discussed earlier in the paragraph titled “Model accuracy, fringing fields, and finite models”, considering the validity of parallel plate capacitor approximation.

**Non-contact on-body electrodes.** Finally, the third option is a compromise: insulated or not, dry electrodes could be placed on the skin indirectly—most commonly over a layer of fabric or cloth (e.g., over a shirt or under the bed sheets). Therefore, in accordance with Figure 5, this case can be considered whenever there is no air gap (*h* = 0, on-body electrodes) and whenever there is at least one “sk,index” layer present (insulated or non-insulated, non-contact electrodes). However, these conditions do not necessarily make the electrodes capacitive. Unlike the case of off-body electrodes (*h* > 0), in most of practical cases of on-body biopotential measurements, the concept of the capacitive electrode is an oversimplification, especially when the electrode is non-insulated (no “el,index” layers present). Namely, as discussed earlier in this section (Figure 3), there is no perfect insulator; hence, some finite leakage current always flows through the interface, creating a conductive path [103,104,249,260]. This is especially the case when a layer of clothing material is used (wearable non-contact on-body electrodes), since such wearable coupling layers often comprise a fairly low resistive component *R*_coupling,sk,index_ in the order of 100 MΩ or even lower [92,98], allowing a significant faradaic reaction at the interface. Moreover, if the fabric is hygroscopic (moisture absorbent), the corresponding coupling impedance *Z*_coupling,sk,index_ could be further reduced in the presence of moisture content. For instance, the cotton sample provided in [92], as the only layer covering the skin, exhibited a dielectric constant *ε*_r,sk,1_ of about 3 and *R*_coupling,sk,1_ higher than 40 MΩ. The electrode used was non-insulated; hence, *R*_coupling,sk,1_ ≈ *R*_coupling_, *X*_Ccoupling,sk,1_ ≈ *X*_Ccoupling_, *ε*_r,sk,1_ ≈ *ε*_r_, and *d*_sk,1_ ≈ *d*. In accordance with (3) and Test 1, for the used 510 mm^2^ of non-insulated electrode sensing area *A*, even if the dried-up cotton sample provided 10 GΩ of *R*_coupling_, it should still not be thicker than about 4 μm (14) for the condition *R*_coupling_ >> *X*_Ccoupling_ to be satisfied across the entire clinical ECG frequency range (lowest frequency not higher than 0.05 Hz in accordance with Assumption 1):

$$R_{coupling}\gg X_{Ccoupling}\to R_{coupling}\geq 10\frac{1}{\omega C_{coupling}}$$

(14) $$\to \;10\;GΩ\geq \frac{10d}{2\pi \cdot 0.05\;Hz\cdot {\varepsilon_{0}\varepsilon }_{r}A}\;\to \;d\leq 4\;\mu m.$$

Therefore, in practice, the resulting coupling in the case of on-body electrodes is seldom predominantly capacitive across the entire frequency range of the measured biopotential signals. Nevertheless, coupling capacitances *C*_coupling,sk,index_ achieved with wearable fabrics (*ε*_r,sk,index_ usually in the range of 1–4 depending on the material and manufacturing process [92,135]) are still higher than the values of total coupling capacitances *C*_coupling_ achieved with off-body electrodes (order of 10 pF compared to the order of 100 fF or 1 pF at best). For a given fabric material, *C*_coupling,sk,index_ can be further increased if a thinner layer is used (3,14).

**Important conclusions and misterming issues as a motivation for the classification of biopotential electrodes.** In the existing literature, all the discussed cases can be found under the terms non-contact, contactless, indirect-contact, and capacitive electrodes. These electrodes are also referred to as non-intrusive, non-obtrusive, or unobtrusive. However, in the previous paragraphs it has been shown that all these terms are not always interchangeable. For instance, the last three terms regarding intrusiveness are broader and describe any sort of unobtrusively applied electrodes, which might consider not only off-body, non-contact on-body, and insulated dry surface-contact electrodes, but also, e.g., non-insulated dry surface-contact electrodes embedded in clothing. Furthermore, in terms of the coupling mechanism, wet surface-contact electrodes described in Appendix B could in fact achieve a notably higher *C*_double-layer_ (order of 10 nFcm^−2^) than the *C*_coupling_ achievable with common dry electrodes intended to be used capacitively, but that still does not make their coupling capacitive. Conversely, while off-body electrodes achieve the purest capacitive coupling, their *C*_coupling_ is reduced to the limits of usability for biopotential measurements [96,98]. On the other hand, whenever a mechanical contact without an air gap is established with the body (on-body electrodes), some amount of leakage current will always flow through the electrode–body interface—especially in the case of using clothing wearable layers. In other words, coating the sensing surface of a dry on-body electrode or separating it from the skin with an on-skin insulating layer does not guarantee that there will be no resistive coupling across the inspected frequency range. For instance, with the use of silicon dioxide coating in [295], *C*_coupling_ in the order of 1 nF and higher was achieved, whilst *R*_coupling_ might have been in the order of 100 MΩ. Yet, in accordance with Test 1 and (14), even for such a low *R*_coupling_, coupling would become predominantly capacitive already at frequencies in the order of 1 Hz. Moreover, additional effects, such as the aforementioned moisturization and humidification, can easily make an on-skin insulating layer conductive. In that case, a layer would remain insulating only in terms of physically separating the on-body electrode from the skin, rather than providing electrical insulation. Similarly, while insulated surface-contact electrodes could be treated as both electrically insulating and capacitive as long as there is no electrochemical reaction at the interface, their coupling conditions might easily worsen once they are applied over a layer of fabric or separated via an air gap. Namely, while they could still be treated as insulated due to preserved high *R*_coupling_ (12), the total *C*_coupling_ would become dominated by the lowest of all individual layer capacitances, *C*_coupling,layer_ (10). Due to all these considerations, the initially used term “insulating layer” has been broadened and replaced with the term “coupling layer”. Just as a combination of faradaic and non-faradaic processes is always present at the interface with a non-insulated surface-contact electrode (Figure A2), so can the coupling with a non-contact on-body electrode always be described as a combination of capacitive and resistive coupling, rather than as pure capacitive coupling. Accordingly, a more reliable representation of the impedance of each on-skin (“sk,index”) and on-electrode (“el,index”) coupling layer would be the coupling capacitance *C*_coupling,layer_ (e.g., *C*_coupling,sk,1_ or *C*_coupling,el,1_) shunted by the insulation or leakage resistance *R*_coupling,layer_ (e.g., *R*_coupling,sk,1_ or *R*_coupling,el,1_), as in Figure 5. Additionally, and especially in the case of fabric layers, some papers [66,296] add a separate resistance *R*_contact,sk,index_ in series with the *R*_coupling,sk,index_*C*_coupling,sk,index_-parallel as a reminder of non-zero conductivity at the boundaries with adjacent conductive surfaces. To conclude, instead of treating electrodes as pure resistive or pure capacitive, they should rather be treated as predominantly resistive or predominantly capacitive across a certain frequency range. Moreover, contact with the surface of the skin does not guarantee predominantly resistive coupling, nor does placing the electrode over a layer of fabric or coating its sensing surface guarantee predominantly capacitive coupling. In fact, it is almost always more appropriate to treat non-contact on-body electrodes as dry surface-contact electrodes with a higher leakage resistance. Similarly, it is almost never advisable to treat non-contact biopotential measurements as capacitive (e.g., it is more appropriate to use the term non-contact ECG rather than capacitive ECG). In accordance with these issues, it becomes clear that using the group term “non-contact and capacitive electrodes” to describe electrodes alternative to conventional wet and dry surface-contact electrodes is not entirely appropriate. This will be further discussed in the next paragraph.

**Figure 6:**
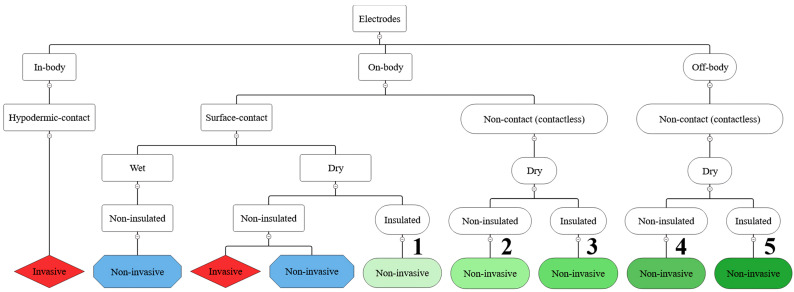
Categorization of electrodes based on their distance from the body, type of contact, and invasiveness, with the assessment of their predominant coupling mechanism (resistive or capacitive). More details are available in the text.

**Proposal for the classification of biopotential electrodes.** To avoid misterming in the future, a categorization of biopotential electrodes is proposed in Figure 6 based on their distance from the body, type of contact, and invasiveness, along with an assessment of their predominant coupling mechanism. In the bottom row of the classification tree, red, sharp diamond blocks represent invasive electrodes, whereas the rest of the electrodes are non-invasive, meaning that they do not require bypassing the stratum corneum or skin protrusion of any kind. Among them, blue octagons represent non-insulated surface-contact electrodes, which are described in Appendix B and which rely mostly on the faradaic process and resistive coupling with the skin. On the other hand, green ellipsoidal blocks represent various types of dry electrodes, in which the influence of resistive and capacitive coupling depends on the electrical and dielectric properties of the electrode–body interface. All five proposed green ellipsoidal cases can be gathered under the term “non-contact and insulated electrodes” as a more appropriate replacement for the previous group term “non-contact and capacitive electrodes”. To emphasize the strength of capacitive coupling in comparison with resistive coupling for each of these cases, stronger capacitive coupling is depicted by a darker shade of green. In that sense, cases 4 and 5 (true non-contact, off-body electrodes) are the ones with the most capacitive coupling and the least resistive coupling. With respect to Figure 5, these cases would correspond to the presence of an air gap (*h* > 0). On the other side, cases 1, 2, and 3 correspond to on-body electrodes (no air gap, *h* = 0). The presence of at least one layer on the surface of the skin (sk,1) is needed to consider the electrode as non-contact, whereas the presence of at least one layer coated on the sensing surface (el,1) is needed to consider the electrode as insulated. However, as mentioned in the paragraph titled “Insulated electrodes”, care must be taken: since wet electrodes are not characterized by the presence of the electrolytic solution itself, but rather by the electrochemical reaction between the electrode material and the solution, an electrochemical reaction with solutions such as sweat could effectively turn any non-insulated or even insulated dry on-body electrode into a poor surface-contact electrode. In any case, based on (3), the total coupling capacitance *C*_coupling_ can be approximated with a series of parallel plate capacitors, as in (10,11). Similarly, based on (7), the total resistance *R*_coupling_ can be approximated with (12,13). The terminology presented in Figure 6 will be used throughout the rest of this paper for identifying and comparing different categories of electrodes: wet surface-contact and non-insulated dry surface-contact electrodes described in Appendix B on the one side, and off-body, non-contact on-body, and insulated dry surface-contact electrodes on the other side. This latter group of electrodes will be referred to as non-contact and insulated electrodes. In the context of non-contact and insulated electrodes, the total coupling impedance *Z*_coupling_ of the coupling path, i.e., the impedance between the electrode sensing surface and the surface of the skin, is often called the electrode–body impedance (EBI). Finally, Assumption 4 can be expressed.

**Assumption** **4.** ***Electrode–body interface, coupling layers, and coupling impedance.** In accordance with the discussion laid out in this section and the proposal for revised classification given in Figure 6, “non-contact and capacitive electrodes” (or occasionally just “non-contact electrodes” for simplicity’s sake) will from now on be more precisely referred to as “non-contact and insulated electrodes”. This term will consider off-body, non-contact on-body, and insulated dry surface-contact electrodes, with a caveat that on-body electrodes have proved to be preferred. The term “coupling impedance” will be used for describing the total equivalent impedance of the coupling path between the electrode sensing surface and the surface of the skin, Z*_coupling_ *(Figure 5). In other words, internal body and tissue impedance, as well as impedance of the stratum corneum, are excluded from the coupling impedance and will be denoted by a separate equivalent skin–tissue impedance Z*_body & skin_*. This will allow the employment of the voltage measured at the surface of the skin, v*_surface_(*t*)*, to be the starting point for circuit analysis. Specifically, in the case of wet and non-insulated dry surface-contact electrodes (Appendix B), the term “coupling impedance” would correspond to the total impedance of the electrode–hair–electrolyte–skin path (electrode–skin path for short), comprising both the electrode or electrode–electrolyte impedance and the contact or electrolyte impedance. To make a distinction between wet and non-insulated dry surface-contact electrodes on the one side (Figure A2), and non-contact and insulated electrodes on the other side (Figure 5), and allow easier comparison with the existing papers, the “electrode–skin” term will be primarily used for addressing the coupling with the former, whereas a more general term “electrode–body” will be primarily used to stress the coupling with the latter. Similarly, terms such as “contact surface” and “contact area” will be primarily used to address the sensing surface of the former, whereas terms such as “coupling surface” and “coupling area” will be primarily used to address the sensing surface of the latter. Therefore, the coupling impedance Z*_coupling_* of wet and non-insulated dry surface-contact electrodes will also be called the electrode–skin impedance (ESI), whereas Z*_coupling_* of non-contact and insulated electrodes will also be called the electrode–body impedance (EBI). Accordingly, the electrode–tissue impedance (ETI) [176] would denote the equivalent impedance that accounts for both the skin–tissue impedance Z*_body & skin_* and the coupling impedance Z*_coupling_*. Further, insulation, leakage, or coupling resistance as the real (resistive) part of Z*_coupling_* shall be denoted by R*_coupling_*, whereas the coupling capacitance and the imaginary (reactive) part of Z*_coupling_* shall be denoted by C*_coupling_ *and X*_Ccoupling_*, respectively. The relation between C*_coupling_ *and X*_Ccoupling_ *is expressed in (A4). In total, Z*_coupling_ = *Z*_Ccoupling_||*R*_coupling_ = (*−jX*_Ccoupling_)||*R*_coupling_*. Accordingly, each layer that builds the electrode–body interface represents one coupling layer. The term “coupling layer” is preferred to the term “insulating layer” to remind that coupling layers are not necessarily insulating from the standpoint of electrical conductivity. For each of the coupling layers, Assumptions 2 and 3 are applicable, and the corresponding coupling layer impedance is Z*_coupling,layer_ = *Z*_Ccoupling,layer_||*R*_coupling,layer_ = (*−jX*_Ccoupling,layer_)||*R*_coupling,layer_*, where the subscript “layer” can stand for “sk,index”, “air”, and “el,index”. In the case of air gap, only the parameter C*_coupling,air_ *is considered. This notation is described in more detail in paragraph “Multiple insulating or coupling layers, equivalent coupling capacitance, and equivalent coupling resistance.” Based on parallel plate approximations for each C*_coupling,layer_*, Equations (3), (10) and (11) will be used to describe the equivalent C*_coupling_ *and ε*_r_ *of the entire stackup. On the other hand, based on the analysis given in Table 1, only the DC component of the leakage resistance is considered in (9) (subcase A.1). Therefore, R*_coupling,DC,layer_ *= R*_coupling,laye_*_r_, ρ*_DC,layer_ *= ρ*_layer_ *, and so Equations (7), (12) and (13) will be used to describe the equivalent R*_coupling_ *and ρ of the stackup. Finally, the predominant coupling mechanism will be determined in accordance with Test 1. Test factor T = 10 will continue to be used, as well as “>>” and “<<” conditions that indicate “at least an order of difference”. Practical examples are given in (14) and the paragraph titled “Important conclusions and misterming issues as a motivation for the classification of biopotential electrodes.”*

**Figure 7:**
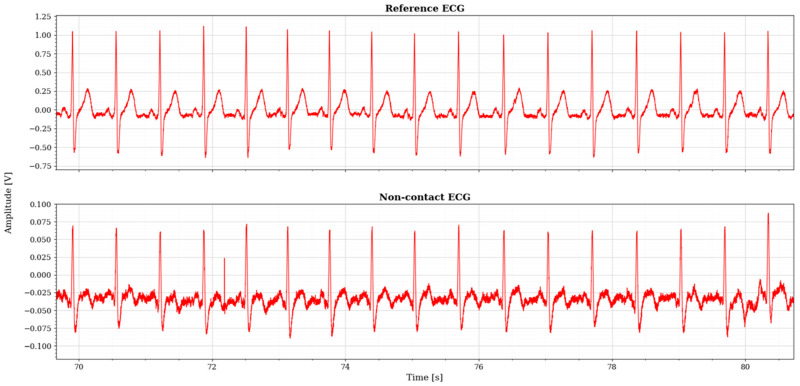
Comparison of simultaneous ECG recordings obtained (**top**) with reference gel surface-contact electrodes and (**bottom**) non-contact electrodes attached to a driver seat; excerpt adapted from UnoViS auto2012 dataset [149].

**Examples of measurements and motivation for further research on non-contact and insulated biopotential electrodes.** Through contactless and remote sensing properties independent of the skin, non-contact and insulated electrodes offer maximization of user safety, at the same time minimizing preparation, intrusiveness, and subjects’ anxiety, as well as the possibility of irritation and allergic reaction. All these advantages show that with the use of a non-contact and insulated measurement approach, we are one step closer to out-of-hospital unobtrusive biomonitoring in a day-to-day environment. Nevertheless, the challenges of low coupling capacitances and absence of direct contact with skin remain to be tackled. The resulting differences between signals measured with non-contact and insulated electrodes on the one side, and surface-contact electrodes on the other side, can be significant (Figure 7) [32,297]. Other interesting examples of comparison in performance and recorded biopotential signals are available in the literature—e.g., comparison between wet, dry, and insulated surface-contact electrodes [197], comparison between all types of non-invasive electrodes [121,122,123], comparison between non-contact on-body electrodes placed directly on the skin and over fabric (Figure 3.5 in [103]), comparison between wet surface-contact electrodes and non-contact on-body electrodes placed over various fabric material [32,297,298], and comparison between surface-contact electrodes and off-body electrodes at various distances [9]. In this study, the phenomena behind these results will be addressed through a thorough system analysis of non-contact and insulated electrode–body interfaces, as well as by demystifying the key requirements of their design. As a common application example, surface-contact ECG and non-contact ECG (sometimes ill-advisedly referred to as capacitive ECG or cECG) measurements will be used. More on noise sources in ECG signals, as well as on the properties and frequency spectrum of ECG signals, can be found in [152,153,154].

**Fundamental literature overview.** Throughout the following sections, fundamentals of passive components and capacitor working principle will be extensively used. The literature on this fundamental knowledge can be categorized as follows:

- Fundamentals of dielectrics, polarization, and capacitors: insulators and dielectrics on a quantum level in [256], and polarization effect in [255,256,257,258,260,284]; extensive discussion on electrical, mechanical, and thermal properties of dielectrics is given in [257]; physical basis of capacitance is given in [256,258,259,263,285] and further extended to linear and non-linear capacitor models and their properties in [264], and applications of capacitor circuits in [261,264]; more on properties and manufacturing of real-world capacitors is given in [260,261] and subchapter 1x.3 in [282]; an overview of historical development of capacitive sensing is given in [10].
- Considerations for higher frequencies, such as transmission line effects, controlled impedance, and dielectric loss are given in [257,283,289,299,300,301]; more on dielectric loss specifically can be found in [256,260,270,283,289] and subchapters 7.1 and 7.8 in [257].
- Considerations for higher voltages, electrical discharge, electrical breakdown, and dielectric failure mechanisms can be found in [255,256,275,286] and subchapter 7.10 in [257].
- Physical basis of resistance [287] and inductance [302]; therein presented fundamentals, along with references on physical basis of capacitance listed in the previous bullets, can be extended to discussions on modeling PCB parasitic elements ([274] and subchapter 1x.1 in [282]), package lead parasitics [303], and on-chip interconnects parasitics [304], as well as equivalent circuits of capacitors, resistors, inductors, and diodes [260,305,306,307,308]. More on manufacturing of real-world passive components and diodes along with their non-idealities can be found in [282,305,306,307], with a focus on capacitors in [260] and subchapter 1x.3 in [282], and with a focus on resistors in [288] and subchapter 1x.2 in [282].
- Useful approximations for capacitance, resistance, and inductance of various practical geometric configurations can be found in [258,259,272,274,287,302,306]. Incidentally, an example of theoretical modeling of wire inductance and capacitance between the wire and the ground can be found in [280];
- Comparison between working principles of capacitors and inductors [264,265,309] that will provide a further insight into analogies between capacitive and inductive coupling mechanisms.
- Basics of phasor analysis [260,265,266,267,268].
- Guide to understanding Maxwell’s equations [262,263,285,289,291]; more on electric field calculation and numerical simulations can be found in [255,258,275,276,289,291].
- Finite parallel plate capacitor models [15,277,278,279,280,281].
- Various important rules and useful formulae for board-level and system-level design are highlighted in [273] and will be occasionally referenced. Various PCB design issues are covered in [274,306], chapter 3 in [104], and subchapter 1x.1 in [282]; specifically, more on power supply noise and decoupling is given in [310].
- With the addition of the literature on operational amplifiers and systems theory that will be listed throughout Section 3.2 and Section 4.1, all these references will serve as the basis for the rest of the assumptions.

### 3.2. Passive and Active Design

**Single-ended and differential measurement.** Throughout the following analysis of a single non-contact and/or insulated biopotential electrode, signals will be considered as single-ended and referenced to the circuit common. However, in reality, one must be aware that at least two electrodes are required for an ECG measurement [98]. The obtained ECG signal can be either single-ended (unipolar, monopolar), meaning that one electrode measures the ECG signal, whereas the other serves as a reference node with assumed zero electric potential (e.g., circuit common), or it can be differential (also known as single-differential or bipolar), meaning that both electrodes measure the ECG signal. Also, hybrid configurations are possible, such as the pseudo-differential configuration [102]. In any case, the obtained electric potential difference between the two electrodes represents one lead or channel. More on unipolar and bipolar leads can be found in [152,311].

**Figure 8:**
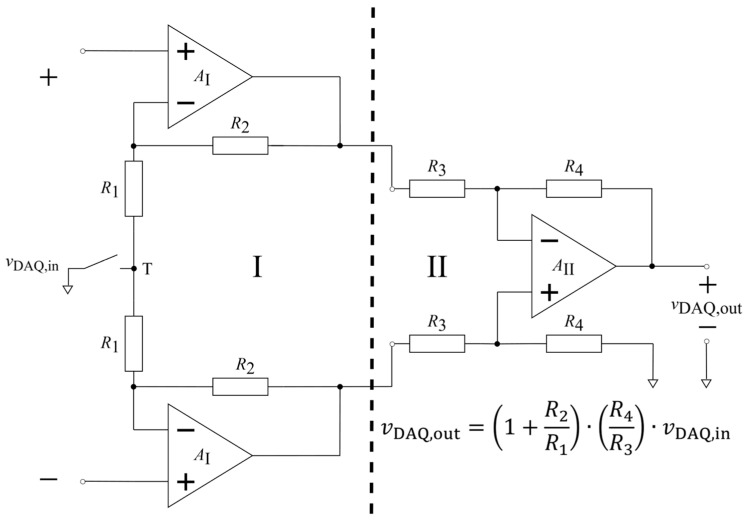
Basic main amplifier topology used for two-electrode biopotential recordings: instrumentation amplifier with first-stage amplifiers (I) in coupled variant (node T disconnected from the circuit common) or non-coupled variant (node T connected to the circuit common) and a single-ended output differential amplifier in the second stage (II), adapted from [102]. The written equation explains the general conversion of an input differential voltage signal, *v*_DAQ,in_, into a single-ended output voltage signal, *v*_DAQ,out_, where DAQ stands for data acquisition. Triangle symbol represents the circuit common (system reference, i.e., amplifier common).

**Passive design and the main amplifier.** In a classic two-electrode biopotential measurement (one-channel ECG), the input signal is differential, and the most convenient approach to achieve signal acquisition and convert that differential signal into a single-ended output signal is to use either a differential-input amplifier or an instrumentation amplifier (Figure 8) that offers improvements in terms of higher input impedance, lower sensitivity to component tolerances, and easier gain adjustment [102]. The input non-inverting amplifiers in the first stage of the instrumentation amplifier are usually coupled or, less frequently, non-coupled. Alternatively, if a differential output is desired instead of a single-ended output, fully differential amplifiers with a differential input and a differential output can be used [312,313,314]. More on various voltage amplifier configurations is given in [315]. Because there are no active electronics mounted on the electrode and applied to the signal path between the electrode sensing surface and the input of the main amplifier, such a design is referred to as passive.

**Figure 9:**
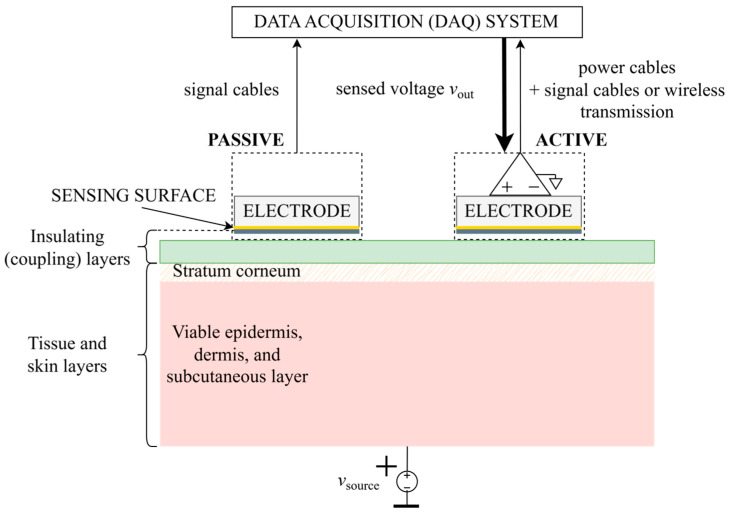
Concept of (left) a passive biopotential electrode and (right) an active biopotential electrode with a preamplifier, based on [139,218]. Depicted electrodes are non-contact and insulated in accordance with Figure 2 and Figure 5. Aside from coupling layers placed on the skin, which are usually fabric or cloth (green), coupling layers can also be coated on the electrode sensing surface (gray) or realized by means of an air gap between the electrode and the body (white space in between). In both passive and active case, the sensed biopotential signal at the electrode output, *v*_out_, is transmitted to the DAQ system or unit for further processing and analog-to-digital conversion. The DAQ input is commonly based on an instrumentation amplifier (Figure 8). Just like before, due to safety regulations, the human body is ungrounded, so its floating body potential is denoted by a bar symbol under the *v*_source_ voltage. This electric potential differs from the reference of the analog front-end mounted on the top of the active electrode (circuit common represented with the triangle symbol).

**Buffer and amplifying active electrodes.** Passive design is prevalent in the case of wet surface-contact electrodes, but rather rare in the realm of non-contact and insulated electrodes (some examples of passive non-contact design are given in [316,317]). Namely, input of a high-coupling impedance electrode, just like any high-impedance node, is susceptible to electric coupling with the environment, and thus behaves like a charge-sensitive electrometer due to a lack of low-impedance path to a reference potential (refer to Figures 2–9 in [318], and to [110,274,314]). Therefore, the high coupling impedance of dry electrodes leaves the signal path more vulnerable to environmental noise. This results in attenuation of the signal and in a significantly lower signal-to-noise ratio (SNR). To counteract this issue, the first amplifying stage can be implemented directly on the electrode—most commonly as an additional voltage buffer. While a single-transistor source follower was typical in the early days [242,243,244,245,319], such buffer electrodes are today mostly realized with an operational amplifier in a voltage follower configuration, comprising a negative voltage feedback and a unity closed-loop gain. Since this amplifier precedes the main amplifier (differential or instrumentation, as described in the last paragraph), it is also called a preamplifier. One of the first notable papers describing the importance of such a buffer preamplifier implementation was [319]. Serving as impedance converters, these preamplifiers convert the high input impedance into low output impedance, reducing the noise pickup and signal loss. This allows for a longer wired signal transmission path toward the data acquisition (DAQ) system and its main amplifier (Figure 8). Alternatively, it also enables the option of wireless transmission in the case of a more complex design with a dedicated microprocessor that enables wireless connectivity. However, this comes at the cost of additional wires required for powering the preamplifiers, which is why this design is called active (Figure 9). Usually, the sensing surface is implemented on the bottom layer of the active electrode PCB and connected through vias with the top layer of the PCB, where the analog front-end with the preamplifier is implemented. Such an active approach to combating the high coupling impedance is not limited to non-contact and insulated electrodes—on the contrary, there are many examples of using it for non-insulated dry surface-contact electrodes [218,228,320], and even examples of using it for wet surface-contact electrodes [321]. Aside from the described buffer active electrodes, which use unity voltage gain preamplifiers, active electrodes can also be amplifying or preamplified. In this case, the electrode preamplifiers employ voltage gains higher than unity and, therefore, entirely replace the first stage of the instrumentation amplifier, allowing for a reduction in power consumption and the number of necessary operational amplifiers [102,203,210,322,323]. However, this increases sensitivity to component tolerances, making the possible gain mismatch between the electrodes more problematic. In addition, accounting for the possibility of a DC electrode offset at the input may demand considerable gain limitation. In spite of gain adaptation and DC offset compensation solutions that address these challenges of amplifying active electrodes [322,323], buffer active electrodes with unity-gain voltage preamplifiers still prevail as the most widely used method of signal acquisition in non-contact and insulated biopotential measurements. Further developed from papers such as [319] through fundamental electric potential sensing (EPS) research of the Sussex research group [88,246,247,248,249,250,251,252,253,254], such an approach has remained a standard to this day. Therefore, buffer active electrodes will be further investigated in the rest of the paper. More on the performance and architecture of various active electrodes can be found in [139]. These solutions will be revisited at the end of Section 4.2.

**Transimpedance and charge amplifiers as an alternative.** Specifically, for cases of low-capacitance coupling (*C*_coupling_ in the order of 1 pF or lower) or underwater environments, where impedance of the electrode–body interface *Z*_coupling_ is shunted by the low impedance of the water medium [41,230,289], trans-impedance (current-to-voltage) and charge (charge-to-voltage) amplifiers are a promising alternative [9,293,294,324,325,326,327,328].

**Active versus passive with respect to the proposed categorization.** In conclusion, non-contact and insulated biopotential electrodes are commonly active and, more specifically, designed in a buffer configuration, whereas non-insulated surface-contact biopotential electrodes (especially the wet ones) are commonly passive. However, previous paragraphs have shown that active design does not imply a contactless biopotential measurement method, nor does passive design imply a surface-contact biopotential measurement method. Examples of measurement and comparison between active and passive approaches can be found in [110,329]; also, refer to Figure 1 in [197] and Figures 6 and 7 in [228].

**Assumption** **5.** ***Buffer active non-contact and/or insulated electrode.** In Section 3.1, the equivalent electrical model of the interface between the body and a non-contact and insulated biopotential electrode was developed (Figure 5 and Assumption 4). In Section 4, it will be combined with the model of a buffer preamplifier from the appendix, cross-referenced at the beginning of Section 4.1, to analyze a single buffer active non-contact and/or insulated electrode and its interface with the body. Throughout the paper, the terms “voltage follower” and “buffer” will be used interchangeably. The entire system (interface–electrode–preamplifier) will be denoted by the subscript “el” and separated into two subsystems: the input voltage divider (subscript “div”) and the buffer preamplifier (subscript “preamp”), with individual equivalent electrical parameters defined in the first pages of Section 4.1. Details on variable naming is given in Assumption 6. The analysis is applicable both to non-contact and to insulated active electrodes. For this reason, the “and/or” conjunction is used to emphasize that the generalized non-contact and insulated electrode–body interface can be non-contact only, insulated only, and both non-contact and insulated. In addition, depending on the validity of assumptions on the operational amplifier, which will be summarized in Section 4.1, and depending on the interpretation of the term “coupling impedance” with respect to the type of the electrode (Assumption 4), the analysis is further applicable to any given non-invasive active biopotential electrode. The electrode will be employed for ECG measurements, with its input denoted by v*_in_ *and output denoted by v*_out_*. In accordance with Assumption 4, voltage v*_surface_*, that would have been measured with an ideal equivalent passive non-insulated dry surface-contact electrode at the surface of the skin, will be the starting point for circuit analysis. For the purpose of achieving a gradually complexified analysis throughout the study, electric potential difference between the floating body potential (bar symbol) and the circuit common (triangle symbol) will be neglected for the rest of this paper. Therefore, voltages v*_surface_*, v*_in_*, and v*_out_ *will be referenced to the circuit common. Similarly, zero initial conditions will be considered.*

**Assumption** **6.** ***Variable naming.** In general, the following convention will be used for naming currents and voltages: lowercase letters will indicate time-domain variables with the argument (t). On the other hand, uppercase letters will be used to denote frequency-domain variables with either argument (s) as the complex frequency s = σ + jω that will indicate the Laplace transform, or argument (jω) or (jf) in accordance with (A2). Likewise, unless used in the Laplace s-domain, impedances Z are assumed to be functions of (jω) or (jf). Accordingly, reactances X are functions of (ω) or (f) (A4). Specifically, $\dot{V}e^{j\omega t}$ and $\dot{I}e^{j\omega t}$ represent rotating phasors as analytic representations of voltage and current with their static parts or phasors $\dot{V}$ and $\dot{I}$, respectively (A1). In addition, uppercase letters will also be used to denote any type of quantity that is assumed to be constant or fixed within the observed time frame, such as power supply voltages and mean average values.*

## 4. System Analysis

### 4.1. System Transfer Function and Impulse Response

**Figure A5:**
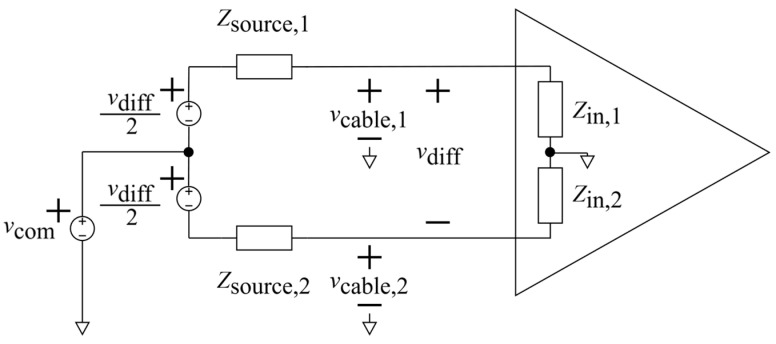
General definition of the common-mode input voltage component, *v*_com_, and the normal-mode or differential-mode input voltage component, *v*_diff_, adapted from [339]. Since the voltage amplification is of interest, and not the power transfer, the source impedance *Z*_source_ should be as low as possible compared to the input impedance *Z*_in_. In the case of the analyzed electrode–body interface, *Z*_source_ will be defined by *Z*_coupling_, while Z_in_ will be defined by the voltage buffer preamplifier and, together with the contribution of external input networks and parasitic capacitances, observed in the context of the input voltage divider effect, as described in Section 4.1. Finally, *v*_cable,1_ and *v*_cable,2_ represent voltages from the respective conductor (wire or PCB trace) to the circuit common, denoted by the triangle symbol.

**Figure A6:**
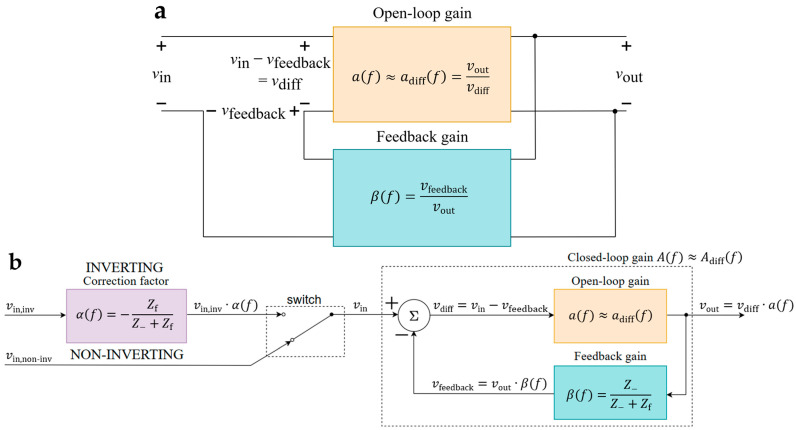
(**a**) Control system representation of the series–parallel or series–shunt negative feedback topology [334,340,342]. Voltage *v*_diff_ is also known as *v*_error_. (**b**) Block diagram for derivation of the ideal closed-loop differential-mode voltage gain *A*_diff_ of a non-inverting and inverting operational amplifier [315]. Open-loop differential-mode voltage gain *a*_diff_ represents the gain of the forward path. It is assumed that the amplifier ignores common-mode input signals, i.e., *a ≈ a*_diff_. Similarly, *A ≈ A*_diff_, as will be discussed in the following pages. *Z*_−_ is the external impedance present at the inverting input terminal, whereas *Z*_f_ is the impedance of the feedback path. Definitions of *Z*_−_ and *Z*_f_ impedances, as well as *v*_in,inv_ and *v*_in,non-inv_ voltages, can be found in the next figure. The summing junction represents the inverting input terminal. In the case of the inverting amplifier configuration, the additional correction factor *α* is present at the input to account for the fact that the position of the input voltage is changed with respect to the non-inverting configuration. This standard control system representation, based on feedback theory [315,341,342], assumes that the open-loop voltage gain *a* remains unchanged after connecting the source, load, and *β*-network (feedback network). Also, it is assumed that the signal flows from the input to the output exclusively through the forward path, and returns from the output to the input exclusively through the feedback path. In other words, *β*-network is unidirectional or unilateral—it does not couple any signal from the input to the output. For a more complexified analysis that considers feedback networks as bilateral, accounting for the feedthrough gain and resulting in Blackman’s full-blown impedance formula, refer to [334].

**Figure A7:**
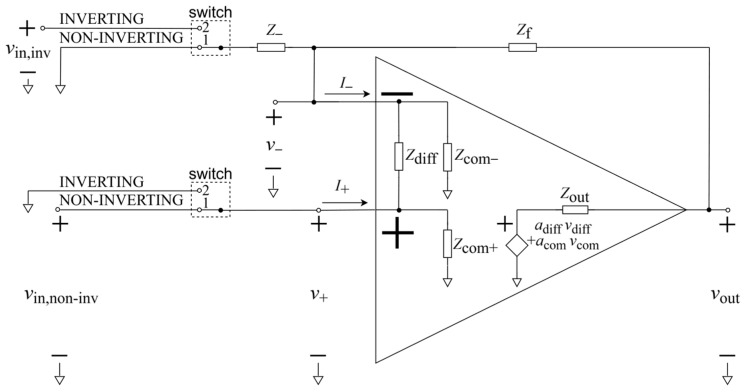
Basic configuration of a non-inverting and inverting operational amplifier, along with input and output impedances, external impedances, and the total open-loop voltage gain *a*, based on [315,330,331,332,333,334]. Therein, *I*_+_ and *I*_−_ represent DC bias currents with their reference directions. The difference between the two configurations is symbolized by two switches: in the case of a basic non-inverting configuration (switches in position 1), input is applied to the non-inverting (“plus”) input terminal and denoted by *v*_in,non-inv_, whereas in the case of a basic inverting configuration (switches in position 2), the input is applied to the inverting (“minus”) input terminal and denoted by *v*_in,inv_. In either case, one switch is connected to the circuit common, and the other to the input signal. In accordance with the idealized control system representation from Figure A6, *a ≈ a*_diff_.

**Figure A8:**
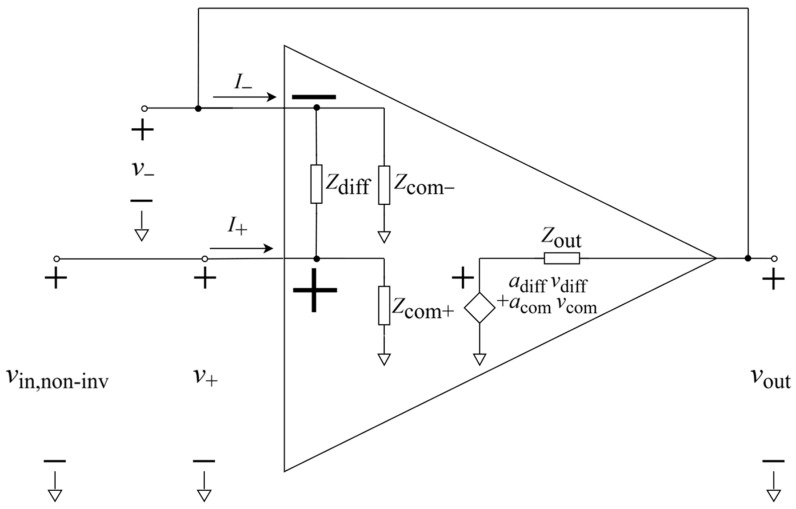
Non-inverting voltage-feedback operational amplifier configured as a voltage follower (see subchapter 2.6 in [331]). In accordance with the analysis, *a ≈ a*_diff_. In this paper, the terms “voltage follower” and “buffer” will be used interchangeably.

**Figure A9:**
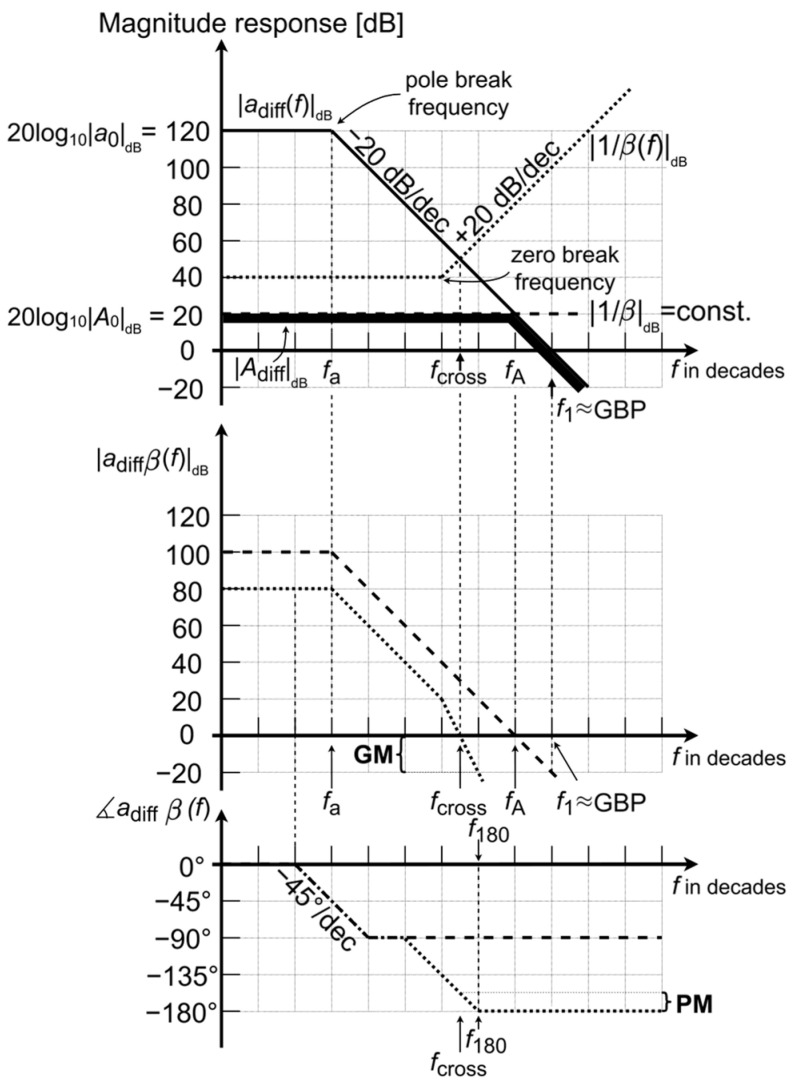
Example of using Bode plot to observe the frequency characteristic (frequency response) of a first-order (single-pole) internally compensated model of a non-inverting voltage-feedback operational amplifier and assess its stability. The fundamentals of Bode plots are given in [273,341,348,349]. Therein, Bode approximation of two basic first-order, single-pole systems is constructed: first, approximation of the system $a_{0}/\left(1+j\frac{\omega }{\omega_{\mathrm{break}}}\right)$—a lag network with a trivial zero (at infinity), depicting the influence of a non-trivial pole, and second, approximation of the system $a_{0}\cdot \left(1+j\frac{\omega }{\omega_{\mathrm{break}}}\right)$ with a trivial pole (at infinity), depicting the influence of a non-trivial zero. These two models will be extensively used in Section 4.2. More on poles and zeros, and on stability at the level of the entire system, can be found in Section 4.1. Specifically, it can be observed that the first-order system with a non-trivial pole corresponds to the first-order model of the open-loop gain *a*_diff_(*f*) (A14), and in this figure, Bode plot is used to assess the stability of the corresponding first-order operational amplifier model. For simplicity’s sake, chosen frequencies are set to be multiples of 10, as adapted from [344]. Thin solid line on the topmost graph depicts the open-loop magnitude characteristic (magnitude response) |*a*_diff_(*f*)|_dB_ = 20log_10_|*a*_diff_(*f*)|, whereas the dashed line on the bottommost graph depicts the open-loop phase characteristic (phase response) ∡*a*_diff_(*f*). Frequency *f*_a_ denotes the open-loop bandwidth, whereas *f*_1_ denotes the open-loop unity-gain crossover frequency, transition frequency, or unity-gain closed-loop bandwidth, which corresponds to the gain-bandwidth product (GBP). Dashed line on the topmost graph depicts the first example—stability assessment for a frequency-independent, purely resistive feedback network with the corresponding 1/*β* = const. Combined with |*a*_diff_(*f*)|_dB_ (thin solid line), it yields the closed-loop gain |*A*_diff_(*f*)|_dB_ (thick solid line). Appropriately, the Bode plot of the corresponding loop gain *a*_diff_*β*(*f*) is given in dashed lines in the middle graph (magnitude |*a*_diff_*β*(*f*)|_dB_) and the bottommost graph (phase ∡*a*_diff_*β*(*f*)). In this case, ∡*a*_diff_*β*(*f*) = ∡*a*_diff_(*f*), and also, the crossover frequency *f*_cross_ corresponds to the closed-loop bandwidth *f*_A_. Next, dotted lines depict the second example—stability assessment for a frequency-dependent feedback network 1/*β*(*f*), described in the discussion titled “Generalized analysis: frequency-dependent feedback network and higher-order models.” In this case, the crossover frequency differs from *f*_A_, hence it is denoted by *f*_cross_. The Bode plot of the corresponding loop gain *a*_diff_*β*(*f*) is given in dotted lines in the middle graph (magnitude |*a*_diff_*β*(*f*)|_dB_) and the bottommost graph (phase ∡*a*_diff_*β*(*f*)). Additionally, phase margin (PM) and gain margin (GM) are indicated for this case, as well as frequency *f*_180_, at which ∡*a*_diff_*β*(*f*_180_) = −180°.

**Figure A10:**
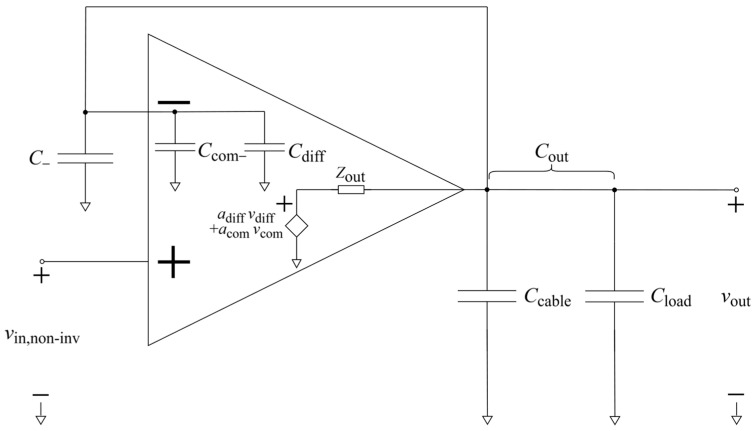
Non-inverting amplifier configured as a voltage follower (buffer) with inverting input and output capacitances that could affect its performance and stability; based on [360]. For a conservative estimate and the measured signal looking into the non-inverting input, all five capacitances are referenced to the circuit common and effectively seen in parallel, creating the total equivalent worst-case capacitance *C*_total_ [345]. Specifically, parallel *C*_cable_||*C*_load_ is referred to as the total effective capacitive load *C*_out_. This voltage follower model, along with the model in Figure A8, will be employed in Section 4 as a buffer preamplifier mounted on a buffer active electrode and investigated as the buffer preamplifier subsystem. Capacitances at the inverting input and the output of the buffer preamplifier will be treated in accordance with conclusions of the analysis (A21). On the other hand, capacitance present at the non-inverting input terminal will be introduced and observed separately in the paragraph “Parasitic input capacitance” in Section 4.1.

**Introduction to Section 4 and the first subsystem: operational amplifier.** In accordance with Assumption 5, in Section 4, a buffer active non-contact and/or insulated biopotential electrode and its interface with the body will be modeled and analyzed. The model is based on two subsystems: the generalized model of non-contact and insulated electrode–body interfaces, presented in Section 3.1, and a buffer preamplifier, presented in Appendix D. Therein, fundamentals of operational amplifiers, negative feedback, Bode plot, and amplifier stability are recalled, with a focus on the voltage follower (buffer) configuration. By beginning this section with Appendix D, analysis of the system conveniently starts from the voltage-feedback operational amplifier, which is then configured as a voltage follower and mounted as a buffer preamplifier on a passive non-contact and insulated biopotential electrode presented in Figure 5. This way, a buffer active non-contact and/or insulated electrode is created. Analysis in Appendix D is based on several book chapters and application reports, discussing fundamentals of operational amplifiers and their specifications [330,331,332,333,334], as well as on the previously listed fundamental literature (paragraph “Fundamental literature overview”). Useful calculations are summarized in [273]. In addition, detailed gain analysis of various practical voltage amplifiers is given in [315]. Also, steps will occasionally be supported by a compendium of interesting blog topics on operational amplifier design given in [335]. The analysis will also be corroborated by several examples of amplifiers commonly used as preamplifiers for buffer active electrodes: LMP7721 [336] and OPA129 [337] (Texas Instruments, Inc., Dallas, TX, USA), and AD8641 [338] (Analog Devices, Inc., Wilmington, MA, USA). After introducing references [339,340,341,342,343,344,345,346,347,348,349,350,351,352,353,354,355,356,357,358,359,360,361,362,363,364,365,366,367,368,369,370,371,372,373], Equations (A6)–(A21), and Figure A5, Figure A6, Figure A7, Figure A8, Figure A9 and Figure A10, the provided recollection of the working principles of operational amplifiers and, specifically, buffer amplifiers, leads from Appendix D to Assumptions 7 and 8. Therein, the key assumptions on the operational amplifier, used as a buffer preamplifier, and the preamplifier analog front-end are highlighted, along with PCB design considerations. Afterward, the derived first-order (single-pole) model of a buffer preamplifier *A*_diff_(*f*) (A17)–(A19) from Appendix D will be combined with the generalized model of non-contact and insulated electrode–body interfaces from Section 3.1. The resulting system (interface–electrode–preamplifier) will represent the model of a buffer active non-contact and/or insulated electrode and its interface with the body. Thereafter, the last assumption, Assumption 9, will be expressed, and the rest of this section will analyze the transfer function and impulse response of the entire system. Along the way, additional useful literature on theory of systems and their stability will be provided. Specifically, stability of operational amplifiers, discussed in Appendix D, will be revisited later in Section 4.3. The next paragraph is written as a continuation of Appendix D.

In summary, in Appendix D, the departure from the ideal operational amplifier model was achieved in several aspects: finite input impedance, non-zero output impedance, and finite bandwidth (for more on the ideal operational amplifier model, refer to [330,331,332]). These small-signal dynamic limitations of operational amplifiers, along with large-signal dynamic limitations, such as a finite slew rate [331,332,335,344], will be surveyed in the following Assumption 7. Additionally, static limitations, representing various input-referred DC errors, rejection capabilities, and operating limits, will also be addressed, along with the departure from the rest of the ideal characteristics—zero bias currents, zero input offset voltage and zero input offset current, and absence of noise. More on static limitations and error modeling can be found in [315,330,332,333,335,374,375]. The following assumption on the buffer preamplifier, along with assumptions on parasitic elements expressed in Assumption 8, will be used in the rest of this paper.

**Assumption** **7.** ***Operational amplifier employed as a buffer preamplifier.***

***(I)*** *For the given amplitudes of interest (Assumption 1), the operational amplifier is considered to operate in the linear, small-signal regime. In other words, analysis considers a small-amplitude, time-varying AC biopotential signal superimposed to any DC voltage present at the interface.*
***(II)*** *Open-loop common-mode voltage gain a*_com_* is neglected in comparison with the open-loop differential-mode voltage gain a*_diff_*,* i.e.*, a ≈ a*_diff_ *and v*_out,open-loop_ *≈ a*_diff_*v*_diff_ *(A8). Also, a*_diff_ *is sufficiently high at frequencies of interest (at least 90 dB [336,337,338]) that the virtual short concept can be adopted and that the resulting closed-loop differential-mode voltage gain A*_diff_ *at frequencies of interest depends only on the gain of the external feedback path β. In other words, Equations (A17) and (A18) can be used. The operational amplifier is assumed to be internally compensated and modeled with a first-order system: open-loop gain a*_diff_(*f*) *(A14) and closed-loop gain A*_diff_(*f*) *(A19) with a unity closed-loop gain A*_0_* = 1 V/V = 1 (0 dB) for a voltage follower (buffer) configuration. Given the typical gain-bandwidth product (GBP) of 1 MHz [336,337,338], the influence of capacitance at the inverting input and at the output will be neglected (A21) and revisited in Section 4.3. On the other hand, the capacitance at the non-inverting input will be introduced in the subsequent pages.*
***(III)*** *Given that the implementation of a series–parallel negative feedback increases Z*_diff_*, the relation Z*_diff_* >> Z*_com−_*,Z*_com+_* will be assumed. Similarly, Z*_out_* is decreased by the series–parallel negative feedback and will be neglected [331,333,334,340,342]. Further, Z*_com−_* and Z*_com+_ *are considered to be approximately equal (matched, balanced). Since the negative feedback path is short-circuited (R*_f_* = 0 Ω) and since the signals source is present at the non-inverting input, for practical considerations of the equivalent electrical circuit at the buffer preamplifier input, only Z*_com+_* will be of interest and, from now on, named Z*_in_ *[332].*
***(IV)*** *Furthermore, internal errors of the operational amplifier and effects of its native non-linearities, significantly reduced by the presence of negative feedback, will not be considered.*
***(V)*** *Similarly, the influence of power supply fluctuations on output voltage and their finite rejection, expressed by the power supply rejection ratio (PSRR) [315,330,332,375], are neglected. In general, the implementation and intricacies of amplifier power supply are not considered [333,376], and in addition, proper power supply decoupling, along with proper biasing of internal circuitry, is assumed (refer to Section 27 in [335], and to [310]).*
***(VI)*** *Also, manufacturer tolerances and mismatch in components and input stages, as well as asymmetry in the signal path, are neglected. Therefore, output offset voltage or zero error, as the output voltage present when input terminals are short-circuited and grounded, and input offset current, as the difference between bias currents I*_+_* and I*_−_* (Figure A8) [315,330,332,333,335,374,375], are neglected. Just like DC and slow-fluctuating errors arising from the electrode–body interface, such as the half-cell potential, these DC limitations and offset errors are in practice mitigated through various procedures of correction, filtering, cancelation, and calibration, more on which can be found in [332,333,335,374,375]. For instance, instead of an input series blocking capacitor, DC rejection can be achieved with the use of a DC servo loop that allows for automatic control of amplifier output offset [314,377,378]. Specifically, the influence of non-zero bias currents I*_+_* and I*_−_* will be addressed in the subsequent pages in the context of DC biasing circuitry.*
***(VII)*** *Next, constant ambient conditions and constant ambient temperature are assumed (Assumption 2). Hence, output voltage drift due to the temperature dependence of reverse-biased leakage of p-n junctions, as well as due to the temperature dependence of amplifier input offset voltage and input offset current, is not considered. Similarly, drift with time due to aging is not considered.*
***(VIII)*** *Finally, AC and large-signal limitations can also be considered—more specifically, slew rate and swing limitations. Although the addition of a capacitance for internal compensation severely impacted the slew rate, and although lower amplifier consumption usually draws lower slew rates, commonly used amplifiers achieve slew rates of at least 2 V/μs [336,337,338]. With these values of slew rate, even a 10 V amplitude sine wave would yield a full power bandwidth of at least 31.8 kHz ($\frac{2\;V/\mu s}{2\pi \cdot 10\;V}\;$in accordance with the magnitude of the time derivative of a sinusoid; refer to subchapter 2.2 in [331], and [273,332,335,344,379]). Therefore, slew rates of off-the-shelf amplifiers commonly employed for biopotential monitoring are more than sufficient for most biopotential signals. In other words, rate of change in output voltage is fast enough to track the input voltage. Hence, the slew rate-induced distortion is not considered, and the small-signal bandwidth, described in Appendix D, is rather limited by the input and output swing limitations [330,332,375]. Given the amplitudes of biopotential signals (Assumption 1), clipping distortion (*i.e.*, truncation of signal peaks) as a result of exceeding the swing specifications will not be considered for the system analysis.*
***(IX)*** *In conclusion, the established assumptions are in accordance with similar analyses, such as [21,32,38,95,380].*

**Assumption** **8.** ***Parasitic elements.***

***(I)*** *Given the frequencies of interest (Assumption 1), transmission line effects will be neglected.*
***(II)*** *Furthermore, PCB parasitics can be considered. Firstly, for a typical FR-4 PCB at room temperature, given that a 1 oz (35 microns) thick copper trace exhibits about 0.5 mOhm per square area, unit-length resistance of a PCB copper trace achieves orders of 10–100 mOhms/cm [273,287]. Secondly, PCB trace inductance per unit length is in the order of 10 nH/cm, whereas* via *inductance achieves values of about 1–2 nH [273,302], which is negligible at frequencies of interest (Assumption 1). Thirdly, unit-length stray capacitance between adjacent copper traces on the same layer can reach the order of 100 fF/cm for very small distances of 0.1 mm, which is negligible even for fairly long coupling lengths [259,273]. Therefore, PCB trace resistance and inductance, as well as stray capacitance between adjacent traces on the same layer, will be neglected and considered minimized with proper PCB stack-up and PCB layout (refer to paragraph “Fundamental literature overview” at the end of Section 3.1 for details). Likewise, in the case of using e-textile devices, the parasitic capacitance of conductive yarns that is usually in the order of 1 fF/cm would be neglected at frequencies of interest [381].*
***(III)*** *Aside from PCB and e-textile parasitics, parasitic elements of the operational amplifier (buffer preamplifier) can also be considered. Firstly, in accordance with estimation (A21), capacitance present at the inverting input terminal and output capacitance will not be considered. Their influence, elaborated in Appendix D, will be appropriately revisited in Section 4.3. On the other hand, the paragraph “Parasitic input capacitance” in Section 4.1. will introduce the parasitic capacitance present at the non-inverting input terminal. Secondly, considering the frequencies of interest (Assumption 1), amplifier input inductance [352], as well as parasitic resistance and inductance of package leads [303] and on-chip interconnects [304], will be neglected.*
***(IV)*** *Lastly, non-idealities and parasitic elements of any additional discrete component are neglected at frequencies of interest (Assumption 1); hence, equivalent electrical models for components such as resistors, inductors, ceramic capacitors, electrolytic capacitors, and diodes are not considered. More on equivalent electrical circuits and non-idealities of various components can be found in the paragraph “Fundamental literature overview” in Section 3.1.*

**Figure 10:**
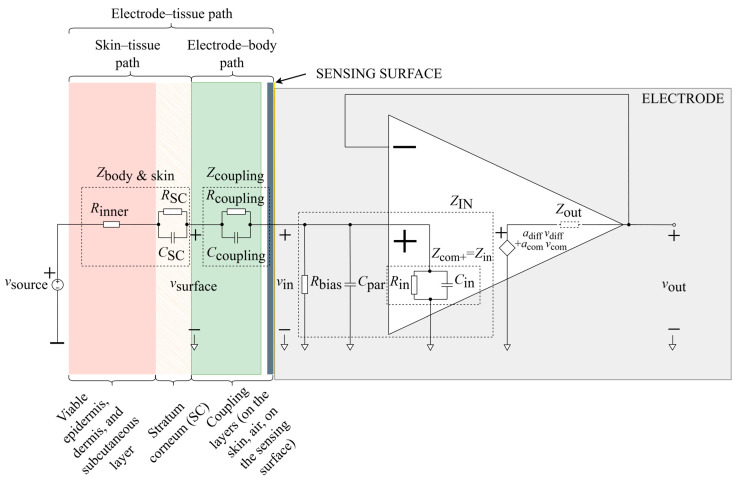
Generalized equivalent lumped-element electrical model of a buffer active non-contact and insulated electrode, implemented with a voltage-feedback operational amplifier in a non-inverting voltage follower (buffer) configuration, and its interface with the body, based on [21,32,38,95,380] and Figure 5, Figure 9 and Figure A8. The model includes all possible coupling layers; hence, the conjunction “and” in “non-contact and insulated electrode” is used instead of “and/or”. As before, due to safety regulations, the human body is ungrounded, so its floating body potential is denoted by a bar symbol under the *v*_source_ voltage, whereas the triangle symbol represents the circuit common. For the purpose of the following system analysis, voltages *v*_surface_, *v*_in_, and *v*_out_ will be referenced to the circuit common (Assumption 5).

**Model of a buffer active non-contact and/or insulated electrode and its interface with the body with three equivalent impedances.** In Section 3.1, an equivalent electrical model of the interface between the body and a non-contact and insulated biopotential electrode was built and generalized (Figure 5) in accordance with Assumption 4. In Section 3.2, a preamplifier was added to the passive electrode, and the electrode became active (Figure 9). In Appendix D, the buffer preamplifier was modeled (Figure A8 and Figure A10), which led to Assumption 7. Now, the model of the electrode–body interface can be extended toward the input of the buffer preamplifier in accordance with Assumptions 5 and 8. This yields the model of a buffer active non-contact and/or insulated electrode and its interface with the body that will be analyzed in the rest of this paper. The generalized model, presented in Figure 10, reveals the existence of three equivalent impedance blocks. The first one, *Z*_body & skin_, represents the equivalent skin–tissue impedance of the layered *RC*-parallels describing electrical properties of the subcutaneous layer, dermis, viable epidermis, and stratum corneum. The second one, *Z*_coupling_, represents the impedance of the electrode–body path or the coupling impedance, i.e., the equivalent impedance of all the coupling layers between the electrode and the surface of the skin, described with the equivalent *R*_coupling_*C*_coupling_-parallel (Assumption 4). Altogether, these two equivalent impedances, *Z*_body & skin_ and *Z*_coupling_, would correspond to the electrode–tissue impedance (ETI) [176]. Finally, the third equivalent impedance block, *Z*_in_, represents the preamplifier common-mode input impedance *Z*_com+_ (Assumption 7). In accordance with the general definition [330], just like *Z*_coupling_ and *Z*_diff_, *Z*_in_ can be modeled with an *RC*-parallel as well: the common-mode input resistance *R*_com+_ = *R*_in_ representing *Z*_in_ at 0 Hz, and the common-mode input capacitance *C*_com+_ = *C*_in_, which models the decrease in the *R*_in_ with increasing frequency. This third impedance block will be further investigated in the next two paragraphs, leading to Assumption 9.

**Parasitic input capacitance.** While the capacitances present at the preamplifier inverting input pin and output were discussed in Appendix D, now, capacitance present at the non-inverting input pin will be introduced. This parasitic capacitance can be modeled with two parallel capacitances referenced to the preamplifier circuit common: the aforementioned common-mode input capacitance *C*_com+_ = *C*_in_ and the additional capacitance *C*_par_. Unlike C_in_, which is an internal capacitance inherent to the semiconductor junctions of the preamplifier input stage transistors and electrostatic discharge (ESD) input protection structures, *C*_par_ is similar to *C*_−_ described in Appendix D. It represents the external pin, pad, and layout capacitance: pin and pad capacitance, via capacitance, and PCB trace-to-reference-plane capacitance. Each of these components are far from negligible, as they could easily add a few pF of capacitance. In fact, for a thin multi-layer stackup, a capacitance of a copper surface to a copper surface on an adjacent layer (in this case, the circuit common plane) can reach the order of 10 pF/cm^2^ (per unit length and width) [99,273,274]. Since the copper PCB path at the non-inverting input pin often includes not only the PCB trace, but also the sensing surface itself (as in the herein analyzed model), it is not surprising that *C*_par_ could total over 120 pF, as simulated in [380]. In addition, if a cable exists at the input of the preamplifier, such as in the case when the preamplifier analog front-end is not implemented directly on the electrode but rather connected via wires, *C*_par_ also comprises cable capacitance similar to *C*_cable_ in Appendix D, indicating the capacitance between the inner conductor and cable shield. Even for low-capacitance cables, this additional capacitance exhibits about 1 pF/cm [259,272,356].

**Bias currents and DC biasing.** As explained in Appendix D with the concept of negative feedback, an operational amplifier will ideally force *v*_−_ to track *v*_+_ by outputting the required voltage and current, but without the need for drawing any current at input terminals. However, in reality, since the common-mode input impedance is finite, DC bias currents *I*_+_ and *I*_−_ exist in the input stage and ESD input protection structures (Figure A8). In fact, their presence at the electrode–body interface is one of the reasons why proper modeling of the electrode–body coupling is essential for fully grasping the phenomena of non-contact and insulated biopotential measurements. Similarly to *v*_diff_ (A6) and *v*_com_ (A7), specifications of operational amplifiers usually define the input bias current as the average $\frac{I_{+}-I_{-}}{2}$ and the input offset current as the difference between the bias currents, *I*_+_ − *I*_−_ [330]. Specifically, the contribution of *I*_−_ can be neglected in comparison with the contribution of *I*_+_, since the inverting input terminal is connected to the preamplifier low output impedance. On the other hand, as described in [382] for an AC-coupled amplifier, if the bias current *I*_+_ is not shunted to circuit common, it charges up the *C*_coupling_ and becomes integrated into voltage (2), which is further amplified by the non-inverting DC closed-loop gain of the preamplifier (A18). Depending on the direction of *I*_+_, this process drives the non-inverting input toward one of the supply rails until eventually the common-mode input voltage range or the output voltage swing is exceeded and the preamplifier is driven out of its range of linear operation and into saturation. The speed of the charging process will be defined by the capacitance and the resistance of the charging path, the product of which describes the time constant. The smaller bias current will take longer to achieve the same effect. Therefore, one way to mitigate this effect is to reduce the bias currents with the choice of the preamplifier. Aside from using amplifiers with lower bandwidth, amplifiers with bipolar junction transistor (BJT) input stages, commonly exhibiting bias currents of up to the order of 1 μA, could be replaced with amplifiers with field-effect transistor (FET) input stages. This way, bias currents are reduced to the leakage currents in the order of 1 pA or even 1 fA, as in the case of the chosen preamplifiers [336,337,338]. However, this comes with an increase in the common-mode input resistance *R*_in_—from typical values up to the order of 1 MΩ in the case of BJT-input stages to the order of 1 TΩ and higher in the case of FET-input stages. Furthermore, the choice of input stage technology comes with additional tradeoffs. For example, although bias currents of the FET-input stages are several orders smaller, they exhibit a severe increase with temperature because they arise from the leakage currents of reverse-biased p-n junctions. Also, while using BJT-input stages typically results in larger bias currents and higher current noise, using FET-input stages may result in larger input offset voltage and higher voltage noise. More on these tradeoffs between input stage technologies can be found in [332,333,383], and subchapters 4x.5 and 4x.10 in [355]. Finally, even if bias currents are minimized, biasing will still remain an issue to be resolved for long-term measurements. To define the DC operating point and provide an alternative pathway for discharging *C*_coupling_, thereby maximizing the output signal range and preventing the input voltage from drifting outside of the common-mode input voltage range, an external DC biasing net is added to the preamplifier input. Most simply, it can be realized with an external input resistor, *R*_bias_ (later, at the end of Section 4.2, more complex biasing circuits will be mentioned). However, additional bias pathways could be inadvertently created in the system as well. Namely, the previously described devastating effect of *I*_+_ considered the electrode–body interface as an AC coupler (infinite *R*_coupling_). In reality, the effect will depend not only on the DC biasing circuit, but also on *R*_coupling_. For instance, as mentioned in paragraph “Non-contact on-body electrodes” in Section 3.1, using hygroscopic fabric as a coupling layer would allow the bias currents to flow through the coupling material toward the skin. With increased perspiration, this would increase the faradaic reaction at the interface and eventually render the DC biasing path ineffective. While this would be an effective way to resolve the issue of bias currents, it would also effectively turn a non-contact electrode into a surface-contact electrode. Therefore, from the methodological point of view, the hygroscopic fabric would begin to serve as merely a spacer that physically separates the electrode from the skin, rather than as an electrically insulating layer with predominantly capacitive coupling at the frequencies of interest [98]. This example stresses the importance of preserving the dedicated DC biasing path and taking into account the finite conductivity of the used coupling layers.

Now, the assumption on the total equivalent input impedance can be expressed.

**Assumption** **9.** ***The third impedance block—the total equivalent input impedance.*** *The first equivalent impedance block in Figure 10, Z*_body & skin_*, was investigated in Appendix B. The second equivalent impedance block, Z*_coupling_*, was investigated in Section 3.1 and defined in Assumption 4. Now, the third equivalent impedance block, describing the total equivalent input impedance Z*_IN_*, can be defined. To summarize, similarly to Z*_coupling_*, the total input impedance Z*_IN_* can be expressed as the R*_IN_*C*_IN_*-parallel. Therein, R*_IN_ *represents the parallel combination of the equivalent DC biasing resistor R*_bias_ *and the preamplifier common-mode input resistance R*_com+_ *= R*_in_ *(*i.e.*, R*_IN_ *= R*_bias_*||R*_in_*). On the other hand, C*_IN_ *represents the total capacitance seen between the preamplifier non-inverting input pin (sensing surface) and the circuit common, expressed as a parallel combination of the pin, pad, layout, and cable parasitic input capacitance C*_par_* and the preamplifier common-mode input capacitance C*_com+_ *= C*_in_ *(*i.e.*, C*_IN_* = C*_par_*||C*_in_*). In conclusion, subscripts “in” with lowercase letters represent equivalent elements inherent to the preamplifier (R*_in_*, C*_in_*, Z*_in_* = *(−*jX*_Cin_)*||R*_in_*), whereas subscripts “IN” with uppercase letters represent equivalent elements of the total input impedance (R*_IN_*, C*_IN_*, Z*_IN_* = *(−*jX*_CIN_)*||R*_IN_*). Following the idea behind modeling the layers and stages with equivalent RC-parallels, some papers [384] add a parasitic resistance component, expressed as R*_par_*, in parallel with C*_par_ *to formally create a distinction with respect to the R*_bias_ *component, especially for the case of more complex models that consider shielding enclosures and additional methods of input capacitance compensation, which will be mentioned later at the end of Section 4.2. However, for the most basic conceptual model generalized in this paper, this separate R*_par_ *component will not be considered.*

**Figure 11:**
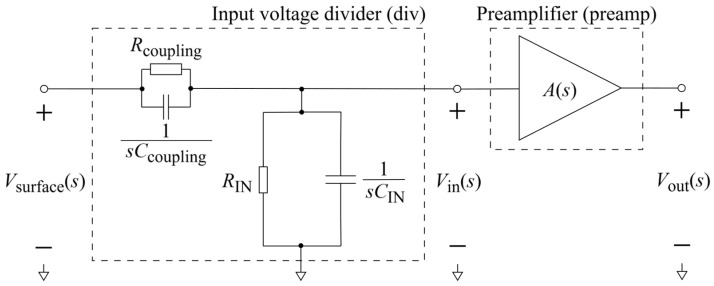
Two-port network with a common ground (circuit common denoted by the triangle symbol) comprising the input voltage divider subsystem and the preamplifier subsystem in accordance with Figure 10 and Assumption 5. Four equivalent passive electrical elements are presented: *R*_coupling_ and *C*_coupling_ build the total equivalent coupling impedance *Z*_coupling_ in accordance with Assumption 4, whereas *R*_IN_ and *C*_IN_ build the total equivalent input impedance *Z*_IN_ in accordance with Assumption 9. Circuit variables, named in accordance with Assumption 6, are functions of the complex frequency *s = σ* + *jω*, which will be used in the process of Laplace transform in the following pages. As explained in Assumptions 4 and 5, by using the voltage *V*_surface_(*s*) as the starting point for circuit analysis, the equivalent skin–tissue impedance *Z*_body & skin_ is excluded from the analyzed system, and the voltages *V*_surface_(*s*), *V*_in_(*s*), and *V*_out_(*s*) are referenced to the circuit common.

**Unique solvability, linearity, and time invariance.** In accordance with Assumption 5, the system interface–electrode–preamplifier comprises two subsystems: the electrode–body interface, modeled as an input voltage divider, and the buffer preamplifier, modeled as a first-order system (A14,A19). It can be analyzed as a two-port network with a common ground (Figure 11). Since the purpose of this system is to allow electric potential sensing without the loading effect and at the same time preserve the shape of the measured signal without introducing any distortion, and since only lumped-element passive electrical components and an operational amplifier serving as a voltage follower for a small AC signal (Assumption 7) are used in the model, the properties of homogeneity and additivity are satisfied. In addition, the network contains only independent sources with the assumption of constant behavior over the time of analysis (i.e., response changes only when the input changes). Hence, the system can be treated as a continuous-time, single-input single-output (SISO), uniquely solvable [385], linear time-invariant (LTI) system. In other words, across the entire frequency range of interest (Assumption 1), the output contains only the frequency components that are already present at the input. The caveat behind these simplifications will be discussed later in Section 4.2, when phase response and group delay of the input voltage divider subsystem will be investigated. Treating the system as LTI also means that the principle of superposition is applicable: every voltage and every current in the network can be expressed as the sum of two terms—zero-state response (response to external inputs as independent sources of perturbation, with initial conditions set to zero) and zero-input response (response to initial conditions with external inputs set to zero). Whereas zero-state response is a result of externally applied independent voltage and current sources, zero-input response is a result of energy previously stored in inductors and capacitors. More on LTI systems and their fundamental properties that will be extensively used in the following pages can be found in [386,387].

**Laplace transform and transfer function.** Under assumed zero initial conditions (Assumption 5), the Laplace transform [387,388] of the impulse response *h*_el_(*t*) (zero-state response to a unit impulse) gives the transfer function or network function *H*_el_(*s*) of this uniquely solvable LTI system. The letter *s*, *s* = *σ* + *jω*, denotes the complex frequency, which comprises the real part *σ*, and the imaginary part, angular frequency *ω*. In accordance with Assumption 5, the voltage sensed with the analyzed active non-contact and/or insulated electrode can be calculated with respect to the voltage *V*_surface_(s) as follows (15):

(15) $$V_{out}\left(s\right)=V_{in}\cdot A\left(s\right)\approx V_{in}\cdot A_{diff}\left(s\right)=V_{surface}\left(s\right)\cdot \frac{Z_{IN}\left(s\right)}{Z_{coupling}\left(s\right)+Z_{IN}\left(s\right)}\cdot \;\frac{A_{0}}{1+\frac{s}{\omega_{A}}}\;\;,$$

where *ω*_A_ is the angular frequency corresponding to the preamplifier closed-loop bandwidth *f*_A_ (A2), which is, in the case of the first-order model of a buffer preamplifier (*A*_0_ = 1 V/V = 1), equal to its unity-gain closed-loop bandwidth *f*_1_ (A17)–(A20). It can be observed that the presence of finite *Z*_IN_ reduces the open-circuit voltage, resulting in circuit loading and voltage divider effect. As mentioned in Assumption 9, *Z*_IN_ can be expressed as the *R*_IN_*C*_IN_-parallel, where *R*_IN_ is equal to *R*_bias_||*R*_in_, and *C*_IN_ is equal to *C*_par_||*C*_in_; hence (16)

(16) $$V_{out}\left(s\right)=V_{surface}\left(s\right)\cdot \frac{\frac{R_{IN}}{1+sR_{IN}C_{IN}}}{\frac{R_{coupling}}{1+sR_{coupling}C_{coupling}}+\frac{R_{IN}}{1+sR_{IN}C_{IN}}}\cdot \frac{A_{0}}{1+\frac{s}{\omega_{A}}}\;\;.$$

Using Laplace transform directly on the input–output form as a more efficient alternative to the state-space approach for SISO systems [388,389] gives the transfer function *H*_el_(*s*) of the analyzed system interface–electrode–preamplifier. As announced in Assumption 5, *H*_el_(*s*) can be simplified into a cascade of the input voltage divider and preamplifier transfer functions, *H*_div_(*s*) and *H*_preamp_(*s*), respectively (17):

$$H_{el}\left(s\right)=\frac{V_{out}\left(s\right)}{V_{surface}\left(s\right)}=\frac{V_{in}\left(s\right)}{V_{surface}\left(s\right)}\cdot \frac{V_{out}\left(s\right)}{V_{in}\left(s\right)}=H_{div}\left(s\right)\cdot \;H_{preamp}\left(s\right)$$

$$=\frac{R_{IN}}{R_{coupling}+R_{IN}}\cdot \frac{1+\frac{s}{\frac{1}{R_{coupling}C_{coupling}}}}{1+\frac{s}{\frac{1}{{{(R}_{coupling}||R}_{IN})(C_{coupling}||C_{IN})}}}\cdot \frac{A_{0}}{1+\frac{s}{\omega_{A}}}$$

(17) $$=\frac{C_{coupling}}{C_{coupling}+C_{IN}}\cdot \frac{s+\frac{1}{R_{coupling}C_{coupling}}}{s+\frac{1}{{{(R}_{coupling}||R}_{IN})(C_{coupling}||C_{IN})}}\cdot \frac{A_{0}\omega_{A}}{s+\omega_{A}}\;.$$

**Figure 12:**
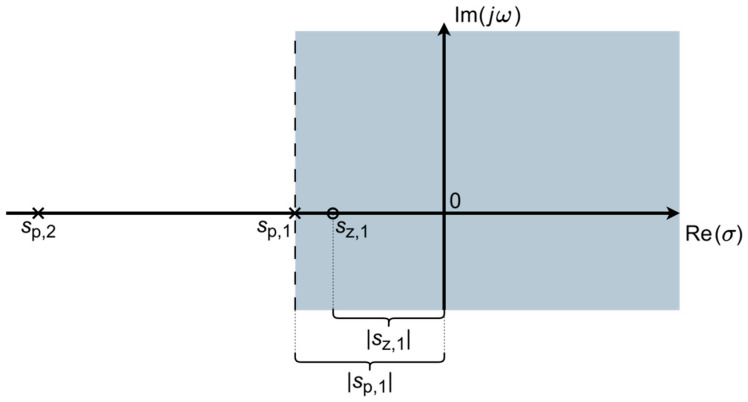
The pole–zero plot in the complex *s*-plane for one arbitrary combination of *R*_coupling_, *C*_coupling_, *R*_IN_, *C*_IN_, and *ω*_A_ values. “Re” stands for real axis (*σ*), whereas “Im” stands for imaginary axis (*jω*). The non-trivial single real zero *s*_z,1_ is marked with a circle, and two non-trivial single real poles *s*_p,1_ and *s*_p,2_ are marked with a cross. Later, in Section 4.2, |*s*_z,1_| and |*s*_p,1_| will be used as absolute values of *s*_z,1_ and *s*_p,1_, respectively, representing their distances from the origin of the complex *s*-plane. The shaded area represents the region of convergence (ROC) of Laplace transform [392].

**Poles and zeros.** Since the analyzed system is a uniquely solvable LTI network, the resulting transfer function *H*_el_(*s*) (17) is a ratio of two polynomials with real coefficients. Also, the null points (roots) of these polynomials are generally either real numbers or they occur in complex conjugate pairs. The null points of the denominator polynomial are called poles and represent complex frequencies at which the transfer function approaches infinity, whereas null points of the numerator polynomial are called zeros and represent complex frequencies at which the transfer function approaches zero. More on the transfer function and geometrical interpretation of poles and zeros can be found at the beginning of [387], and in [390,391]. The degree of the denominator polynomial is 2. Since there are no additional poles canceled by pole–zero cancelation (see [387], subchapter 4.9 in [333], and [346]), both *H*_el_(*s*) and the uniquely solvable LTI system itself are second-order, with two single real poles *s*_p,1_ and *s*_p,2_ (18) (Figure 12):

$$\left[s_{p,1}+\frac{1}{{(R_{coupling}||R}_{IN})(C_{coupling}||C_{IN})}\right]\cdot \left(s_{p,2}+\omega_{A}\right)=0$$

(18) $$\to s_{p,1}=-\frac{1}{\left(R_{coupling}||R_{IN})(C_{coupling}||C_{IN}\right)}=-\left|s_{p,1}\right|\;,\;\;s_{p,2}=-\omega_{A}=-\left|s_{p,2}\right|\;.$$

It can be noticed that |*s*_p,2_| = *ω*_A_ = 2π*f*_A_ is the angular frequency that corresponds to the preamplifier closed-loop bandwidth *f*_A_ (A19,A20). This explains why the first-order model of the preamplifier subsystem (A14,A19), established in Appendix D, is also referred to as single-pole. In Bode approximation of a first-order (single-pole) system (Figure A9) [273,341,348,349], *f*_A_ is also the break frequency of the closed-loop frequency response—|*A*_diff_(*f*)|_dB_ and ∡*A*_diff_(*f*).

Similarly, the degree of the numerator polynomial is 1; hence, the analyzed system contains a single non-trivial zero *s*_z,1_ and a single trivial zero *s*_z,2_ at infinity (19), corroborating that the transfer function approaches zero as the frequency increases:

(19) $$s_{z}+\frac{1}{R_{coupling}C_{coupling}}=0\;\to \;s_{z,1}=-\frac{1}{R_{coupling}C_{coupling}}=-\left|s_{z,1}\right|\;,\;\;s_{z,2}=\infty \;.$$

**Finite bandwidth and causality.** As a result of an excess of non-trivial poles over the number of non-trivial zeros, the numerator polynomial is of smaller degree than the denominator polynomial. Consequently, *H*_el_(*s*) is strictly proper, and the system, just like the preamplifier itself, has a finite bandwidth. Also, the system is strictly causal, meaning that the output depends only on the previous values of the input—it can neither instantaneously nor anticipatorily respond to changes. Given that a finite amount of propagation delay always exists in physical systems, causality is a commonly satisfied property in practical applications. As a result of causality, the region of convergence (ROC) of Laplace transform *H*_el_(*s*) extends to the right of the rightmost pole [392] (Figure 12).

**Stability.** Furthermore, stability analysis can be revisited. In Appendix D, stability analysis has already been carried out for the single-pole model of the preamplifier, *A*_diff_(*f*) (A19). For this purpose, the simplified Nyquist criterion on the basis of the Bode plot was used [350,351]. Now, system stability analysis [392,393,394] can be carried out at the level of the entire system *H*_el_(*s*) described with its poles and zeros. In accordance with the superposition principle, stability can be observed on the zero-input response from the perspective of internal stability, and on the zero-state response from the perspective of input–output stability. From the perspective of internal stability, since the system is causal uniquely solvable LTI, since both poles have negative real parts (18), and since there are no additional canceled poles, the ROC of Laplace transform *H*_el_(*s*) contains the imaginary axis (Figure 12) and the system is asymptotically stable. In other words, the system zero-input response is bounded in magnitude and decays to equilibrium over time for every finite initial condition. Also, asymptotic stability automatically draws bounded-input bounded-output (BIBO) stability as input–output stability: the system zero-state response to a bounded input remains bounded.

**Impulse response.** For the described causal, continuous-time, uniquely solvable LTI system, having all poles in the open left-half complex *s*-plane guarantees its stability. However, it does not guarantee a response without oscillatory decay. Namely, in addition to stability itself, the phase margin must be observed to assess how close the stable system is to becoming unstable. This issue was mentioned in Appendix D, where it was explained that the loop gain determines not only the case of marginal stability and whether the system is stable, but also the amount of gain peaking in the closed-loop frequency response of a stable system, along with the amount of overshoot, ringing, and settling time in its time-domain transient response. Now, that discussion can be related to the location of poles in the complex *s*-plane and their impact on the impulse response *h*_el_. Often described as a fingerprint of an LTI system, the impulse response represents its reaction to a sudden, brief external input—specifically, the zero-state response to a unit impulse—offering further insight into the natural dynamics, transient behavior, and stability of the system “at rest” (i.e., for zero initial conditions). For the analyzed uniquely solvable LTI system, impulse response *h*_el_ can be obtained as the time derivative of the zero-state step response [387], or from the inverse Laplace transform (20,21) after partial fraction expansion of *H*_el_(*s*) (17) [387,388]:

$$H_{el}\left(s\right)=\frac{C_{coupling}}{C_{coupling}+C_{IN}}A_{0}\omega_{A}\;\frac{1}{\omega_{A}-\frac{1}{\left(R_{coupling}||R_{IN})(C_{coupling}||C_{IN}\right)}}$$

(20) $$\cdot \left(\frac{\frac{1}{R_{coupling}C_{coupling}}-\frac{1}{\left(R_{coupling}||R_{IN})(C_{coupling}||C_{IN}\right)}}{s+\frac{1}{{(R_{coupling}||R}_{IN})(C_{coupling}||C_{IN})}}+\frac{\omega_{A}-\frac{1}{R_{coupling}C_{coupling}}}{s+\omega_{A}}\right)\;,$$

(21) $$h_{el}\left(t\right)=\frac{C_{coupling}}{C_{coupling}+C_{IN}}A_{0}\omega_{A}\;\cdot \left(\frac{\left|s_{z,1}\right|-\left|s_{p,1}\right|}{\omega_{A}-\left|s_{p,1}\right|}e^{-\left|s_{p,1}\right|t}+\frac{\omega_{A}-\left|s_{z,1}\right|}{\omega_{A}-\left|s_{p,1}\right|}e^{-\omega_{A}t}\right)\cdot step\left(t\right).$$

**Figure 13:**
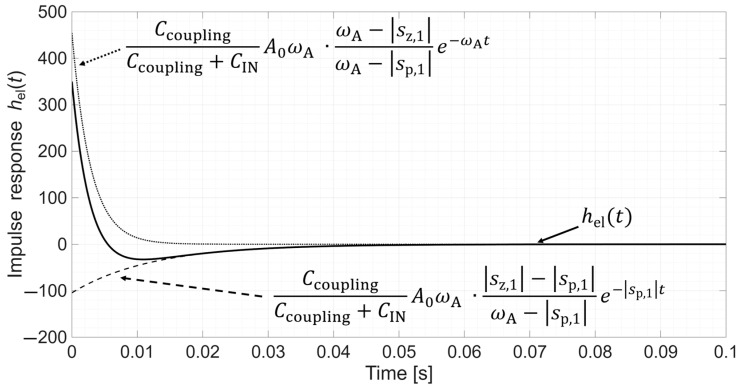
Impulse response *h*_el_(*t*) of the analyzed system interface–electrode–preamplifier based on (21). The plot is obtained in MathWorks^®^ MATLAB R2025b environment [146]. Parameter values are not chosen by the criterion of common use, but rather to clearly illustrate their influence: *R*_coupling_ = 10 GΩ, *C*_coupling_ = 50 pF, *R*_IN_ = 250 MΩ, *C*_IN_ = 0 pF, *ω*_A_ = 350 rad/s. These parameters and their values typically used in practice will be thoroughly explored in Section 4.2 and Section 4.3.

Firstly, since the system is real and comprises zeros and poles that are either real-valued or they occur in complex conjugate pairs, the resulting impulse response *h*_el_(*t*) is a real function. Secondly, as a consequence of causality, *h*_el_(*t*) is zero before the unit impulse is applied at *t* = 0 s, hence *h*_el_(*t*) is right-sided (Figure 13). This is indicated in (21) with multiplication by the Heaviside unit step function step(*t*). Thirdly, it has already been noted that the analyzed uniquely solvable LTI system is asymptotically stable and BIBO stable as a result of being causal and having all poles in the open left-half complex *s*-plane (Figure 12). In addition, *h*_el_(*t*) is absolutely integrable, i.e., the area under the curve |*h*_el_(*t*)| in time (*t*) is finite. Namely, requirement *ω*_A_ > |*s*_p,1_| yields (22)

$$\int_{0}^{\infty }\left|h_{el}\left(t\right)\right|dt=$$

(22) $$\frac{C_{coupling}}{C_{coupling}+C_{IN}}\frac{A_{0}\omega_{A}}{\omega_{A}-\left|s_{p,1}\right|}\;\cdot \int_{0}^{\infty }\left|\left(\left|s_{z,1}\right|-\left|s_{p,1}\right|\right)e^{-\left|s_{p,1}\right|t}+\left(\omega_{A}-\left|s_{z,1}\right|\right)e^{-\omega_{A}t}\right|dt<\infty \;.$$

**Exponential internal stability and the generalized contribution of poles.** Finally, asymptotic internal stability of an LTI system also draws the more stringent property of exponential internal stability: for all initial conditions and zero input, all the circuit variables return to their equilibrium at an exponentially decaying rate. In fact, the prerequisite for using the simplified Nyquist stability criterion with the use of Bode plots in Appendix D [350,351] was to assume that the open-loop gain *a*_diff_(*f*) is exponentially stable, and the stability of the closed-loop response, assessed in Figure A9, was in fact the exponential stability. The same is indicated by the location of poles in the complex *s*-plane (Figure 12): each pole *s*_p_ = *σ* + *jω* in the Laplace *s*-domain (transfer function) contributes to a summation term in the time domain (impulse response). Let the analyzed real system be generalized into a rational transfer function *H*(*s*), expressed as a ratio of polynomials with real coefficients. Then, as explained in subchapter 3 in [387], after partial fraction expansion and inverse Laplace transform, the time-domain modes from each of *n* poles with multiplicity *m* can be generalized as follows (23):

$$h\left(t\right)=L^{-1}\left\{s_{p,1}\;term\;in\;H(s)\right\}+\cdots +L^{-1}\left\{s_{p,n}\;term\;in\;H(s)\right\}$$

(23) $$=\left\{\sum_{i=0}^{m_{1}-1}{const}_{1,i}{\cdot t}^{i}e^{(\sigma_{1}+j\omega_{1})t}+\cdots +\sum_{i=0}^{m_{n}-1}{const}_{n,i}{\cdot t}^{i}e^{\left(\sigma_{n}+j\omega_{n}\right)t}\right\}\cdot \mathrm{step}(t)\;.$$

**Figure 14:**
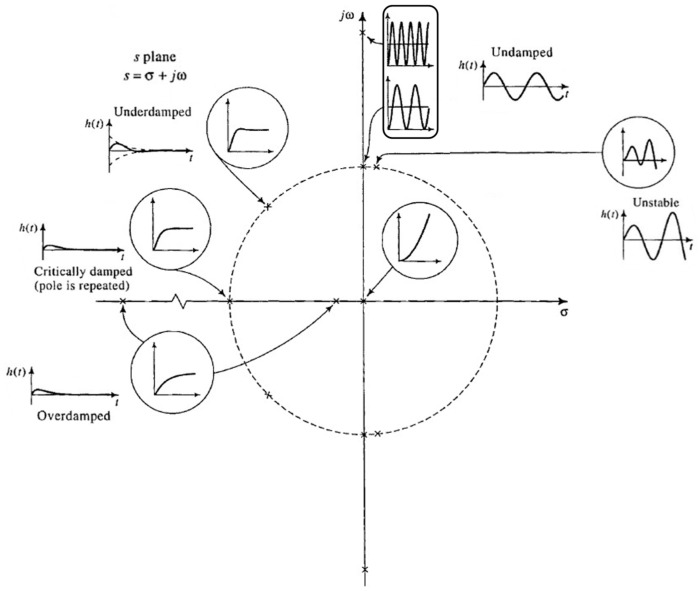
Qualitatively depicted time-domain transient step response (enclosed curves) and impulse response *h*(*t*) with respect to location of real-valued poles or complex conjugate pairs of poles in the complex *s*-plane for the example of a second-order system; adapted courtesy of [395]. More details are available in the text. Similar examples of gain peaking and oscillatory response with respect to the feedback factor *β* and pole location can be found in Figures 8.5 and 8.6 in [345].

Accordingly, as described in [390,391], the imaginary parts of poles in *e^jωt^* terms determine the frequencies of oscillations in the impulse response *h*(*t*), whereas the real parts of poles in *e^σt^* terms control the rates of their exponential change (decay or growth) (Figure 14). In the case of *H*_el_(*s*), both poles (18) are real-valued (*ω*_1_ = 0 and *ω*_2_ = 0), negative (*σ*_1_ < 0 and *σ*_2_ < 0), and single (*m*_1_ = 1 and *m*_2_ = 1); thus, the resulting behavior of *h*_el_(*t*) is overdamped, as obtained in (21) and depicted in Figure 13. To give a physical interpretation from an electrical engineering perspective, this behavior can be explained via an *RLC* circuit: first, there is no inductor to counteract the discharging and charging of the capacitor by allowing for the back-and-forth energy transfer, and second, the resistive component of the circuit dissipates the energy available for storage in the electric field of the capacitor. This translates into an exponential decay without oscillation.

**Convolution and multiplication.** Given how tangled the solution for *h*_el_(*t*) might be in comparison with *H*_el_(*s*), the power of the transfer function as a tool for evaluating LTI systems “at rest” (i.e., for zero initial conditions) now becomes evident. Namely, instead of using the convolution operation with the impulse response to obtain a zero-state response in the time domain, Laplace transform can be used to transform this operation into multiplication with the transfer function in the *s*-domain [387] (24):

$$V_{out}\left(s\right)=L\left\{v_{out}(t)\right\}=L\left\{\left(v_{surface}*h_{el}\right)(t)\right\}=L\left\{\int_{0}^{t}h\left(t-\tau \right)v_{surface}\left(\tau \right)d\tau \right\}=$$

(24) $$=V_{surface}(s)\cdot H_{el}(s)\;.$$

**Figure 15:**
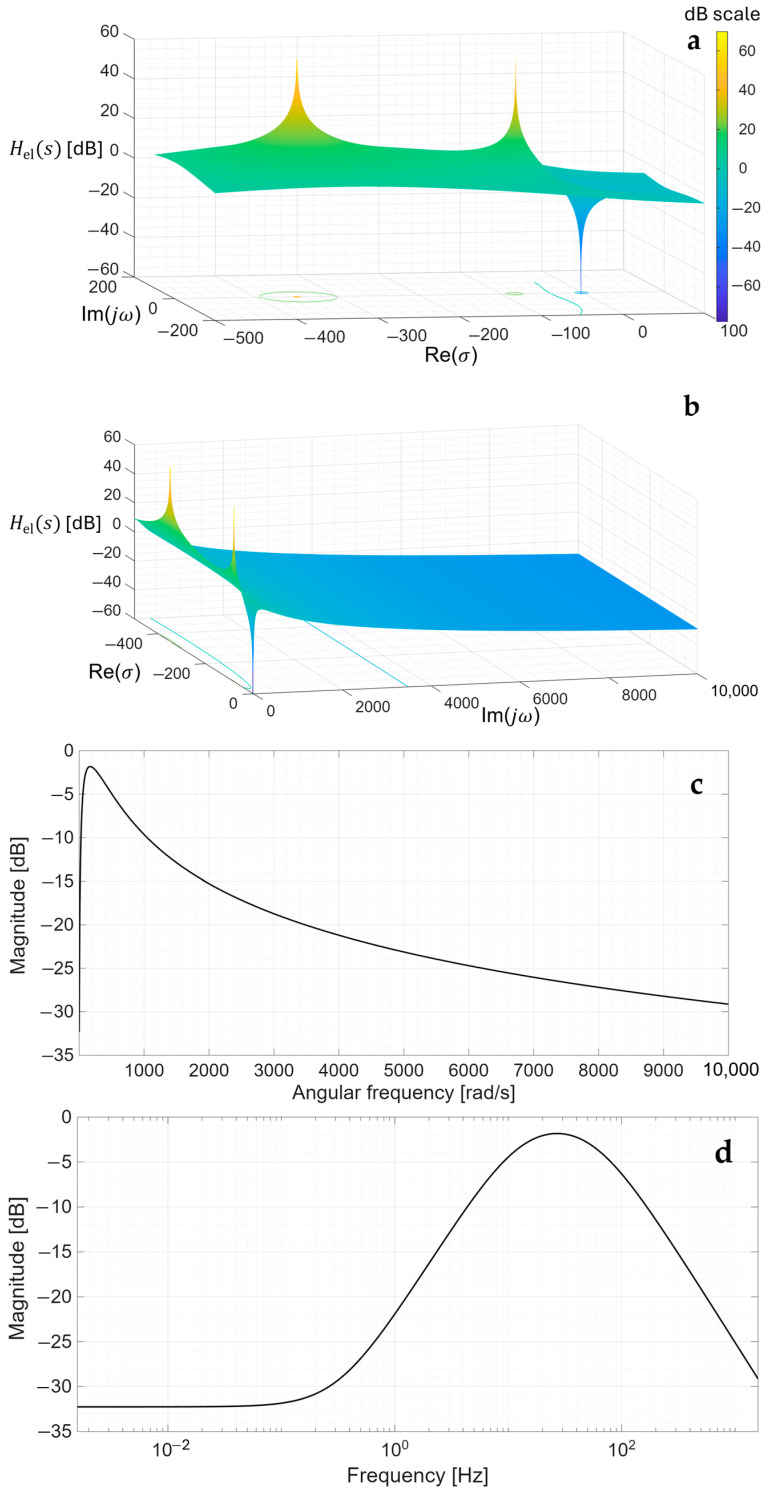
(**a**) 3D transfer function *H*_el_(*s*) in decibels (dB); locations of poles and zeros in the complex *s*-plane correspond to the pole–zero plot depicted in Figure 12; (**b**) The same *H*_el_(*s*) in dB with cross-sections along the planes *σ* = 0 and *ω* = 0; (**c**) translation of *H*_el_(*s*) into 2D magnitude response in dB, |*H*_el_(*jω*)|_dB_; (**d**) once again, |*H*_el_(*jω*)|_dB_ is plotted, but now, angular frequency *ω* = 2π*f* on abscissa axis is replaced with frequency *f* in logarithmic scale. This representation will be used in the rest of the paper and it can be further approximated with the Bode linear piecewise approximation as presented in Figure A9. Parameter values correspond to the ones chosen for impulse response *h*_el_(*t*) in Figure 13. Plots are obtained in MathWorks^®^ MATLAB R2025b environment [146].

**Fourier transform and frequency characteristic.** If the excitation applied to this uniquely solvable LTI system were a complex exponential *Ae^jωt^* with a constant magnitude *A* instead of an exponentially modulated complex exponential *Ae*^(*σ*+*jω*)*t*^, the steady-state response of all voltages and currents would be obtained [268,386,387], in which the contribution of the transient response vanishes. In that case, *s* is substituted with *jω*, and Laplace transform is evaluated along the imaginary axis and reduced to Continuous-Time Fourier Transform (CTFT), which exists since *h*_el_(*t*) satisfies Dirichlet conditions [392]. Lastly, as depicted in Figure 15, the transfer function *H*_el_(*s*) (17) turns into frequency characteristic or frequency response *H*_el_(*jω*) (25), and consequently, circuit variables can be described with phasors (A1), whereas the relation between the voltage and current phasors can be expressed with impedances (A3,A4) [268]:

$$H_{el}\left(j\omega \right)=\frac{\dot{V_{out}\left(j\omega \right)}}{\dot{V_{surface}\left(j\omega \right)}}=\frac{\dot{V_{in}\left(j\omega \right)}}{\dot{V_{surface}\left(j\omega \right)}}\cdot \frac{\dot{V_{out}\left(j\omega \right)}}{\dot{V_{in}\left(j\omega \right)}}=H_{div}\left(j\omega \right)\cdot \;H_{preamp}\left(j\omega \right)$$

$$=\frac{R_{IN}}{R_{coupling}+R_{IN}}\cdot \frac{1+\frac{j\omega }{\frac{1}{R_{coupling}C_{coupling}}}}{1+\frac{j\omega }{\frac{1}{{(R_{coupling}||R}_{IN})(C_{coupling}||C_{IN})}}}\cdot \frac{A_{0}}{1+j\frac{\omega }{\omega_{A}}}$$

(25) $$=\frac{C_{coupling}}{C_{coupling}+C_{IN}}\cdot \frac{j\omega +\frac{1}{R_{coupling}C_{coupling}}}{j\omega +\frac{1}{{(R_{coupling}||R}_{IN})(C_{coupling}||C_{IN})}}\cdot \frac{A_{0}\omega_{A}}{j\omega +\omega_{A}}\;.$$

The frequency response *H*_el_(*jω*) can be visualized by depicting its frequency-dependent magnitude characteristic (magnitude response) |*H*_el_(*jω*)| and phase characteristic (phase response) ∡*H*_el_(*jω*). In accordance with convolution (24) and the superposition principle, the magnitude response of this LTI system acts as amplitude scaling, whereas the phase response acts as a phase shift. In other words, in steady state, each frequency component *ω*_k_ = 2π*f*_k_ of the input signal *v*_surface_ will be scaled by |*H*_el_(*jω*_k_)| and shifted in phase by ∡*H*_el_(*jω*_k_) [268]. This will be further explored in the next two sections. Since the observed system is real, with real-valued inputs and outputs, the frequency response is conjugate symmetric: *H*_el_(*jω*) = *H*_el_^*^(−*jω*). As a result, magnitude response |*H*_el_(*jω*)| is even (symmetric about the ordinate) and phase response ∡*H*_el_(*jω*) is odd (antisymmetric about the ordinate), so it is sufficient to observe the response only at positive frequencies [268,386]. The same is valid for both subsystems, *H*_div_(*jω*) and *H*_preamp_(*jω*). By plotting the frequency response curves (or their linear piecewise approximation—Bode plot [273,341,348,349]), the plot of the three-dimensional (3D) transfer function is replaced with its two-dimensional (2D) cross-section along the plane *σ* = 0 (Figure 15). This way, poles, as complex frequencies in the *s*-plane at which the transfer function goes to infinity, are mapped to break frequencies in the Bode plot. Interesting graphical relations on this subject can be found in [396]. In that sense, just like the transfer function and impulse response, the frequency response is also shaped by the location of poles and zeros, as described in [390,391]. With regard to the frequency domain, in the vicinity of poles close to the imaginary *jω*-axis, magnitude response will be raised toward a local maximum (peak), whereas in the vicinity of zeros close to the imaginary *jω*-axis, magnitude response will be lowered toward a local minimum (dip or notch), with a rapidly varying phase in both cases. Conversely, in terms of the time-domain transient response, the distance of the poles from the real axis will define the frequencies of oscillations, whereas the distance from the imaginary axis will define the rate of their exponential change. Specifically, a complex conjugate pair of non-repeated poles, that lies directly on the imaginary axis, contains only imaginary parts. Therefore, these poles are entirely imprinted in the 2D frequency response, rendering the system marginally stable with sustained, undamped oscillations at the angular frequency that corresponds to the absolute value of their imaginary parts.

The concept of frequency characteristic has already been introduced in Appendix D with the single-pole model of an operational amplifier (preamplifier), *a*_diff_(*f*) (A14) and *A*_diff_(*f*) (A19). The only difference is that now, a more formal expression, with the use of *jω* or *jf* argument rather than *f*, is employed in accordance with Assumption 6. In other words, the model *A*_diff_(*f*) analyzed in Appendix D corresponds to the preamplifier subsystem *H*_preamp_(*jω*) and, specifically, to the buffer preamplifier subsystem for *A*_0_ = 1 and *ω*_A_ = *ω*_1_ = 2π*f*_1_ in (25). Therefore, the rest of the system analysis will focus on the second subsystem, input voltage divider *H*_div_(*jω*) (Section 4.2). Subsequently, the findings will be employed at the level of the entire system, *H*_el_(*jω*) (Section 4.3). More on the frequency characteristic and its construction can be found in [268,347,348,386,390,391,396].

### 4.2. Second Subsystem: Input Voltage Divider

**Input voltage divider effect.** The existence of a voltage divider at the preamplifier input has already revealed the number one reason for the difference between the biopotential that is being sensed by the system, *v*_surface_, and the signal that is acquired at the preamplifier non-inverting input, *v*_in_. This voltage divider effect depends not only on the parameters of the electrode–body interface, but also on frequency. Hence, the frequency characteristic (frequency response) of the input voltage divider subsystem, *H*_div_(*jω*), can be observed in more detail (26):

$$H_{div}\left(j\omega \right)=\frac{\dot{V_{in}\left(j\omega \right)}}{\dot{V_{surface}\left(j\omega \right)}}=\frac{R_{IN}}{R_{coupling}+R_{IN}}\cdot \frac{1+\frac{j\omega }{\frac{1}{R_{coupling}C_{coupling}}}}{1+\frac{j\omega }{\frac{1}{{(R_{coupling}||R}_{IN})(C_{coupling}||C_{IN})}}}$$

(26) $$=\frac{C_{coupling}}{C_{coupling}+C_{IN}}\cdot \frac{j\omega +\frac{1}{R_{coupling}C_{coupling}}}{j\omega +\frac{1}{{(R_{coupling}||R}_{IN})(C_{coupling}||C_{IN})}}=K\cdot \frac{j\omega -s_{z,1}}{j\omega -s_{p,1}}\;.$$

As seen from (26), *H*_div_(*jω*) is first-order and bilinear, with one real pole *s*_p,1_ (18) and one real zero *s*_z,1_ (19) [397]. If the polar form [268,269,390,397] is used (27), magnitude response in dB, |*H*_div_(*jω*)|_dB_ (28), and phase response in degrees, ∡*H*_div_(*jω*) (29), can be further computed from (26)

(27) $$H_{div}\left(j\omega \right)=\left|H_{div}\left(j\omega \right)\right|e^{j∡H_{div}\left(j\omega \right)}\;,$$

$${\left|H_{div}\left(j\omega \right)\right|}_{dB}=20{log}_{10}\left[\frac{R_{IN}}{R_{coupling}+R_{IN}}\cdot \frac{\sqrt{1+({\omega R_{coupling}C_{coupling})}^{2}}}{\sqrt{1+{\left[\omega {(R_{coupling}||R}_{IN})(C_{coupling}||C_{IN})\right]}^{2}}}\right]$$

(28) $$=\;20{log}_{10}\left[\frac{C_{coupling}}{C_{coupling}+C_{IN}}\cdot \frac{\sqrt{\omega^{2}+{\left(\frac{1}{R_{coupling}C_{coupling}}\right)}^{2}}}{\sqrt{\omega^{2}+{\left(\frac{1}{{(R_{coupling}||R}_{IN})(C_{coupling}||C_{IN})}\right)}^{2}}}\right]\;,$$

(29) $$∡H_{div}\left(j\omega \right)=atan2\left(\omega R_{coupling}C_{coupling}\right)-atan2\left[\omega (R_{coupling}||R_{IN})(C_{coupling}||C_{IN})\right]\;,$$

where atan2 is the two-argument arctangent function that accounts for the quadrant of the result based on the signs of both arguments, wrapping the results in a [−180°, 180°] or [−π, π] rad interval in accordance with (30) [398]:

(30) $$atan2\left(\frac{y}{x}\right)=\left\{\begin{array}{cc} \arctan\left(\frac{y}{x}\right), & x>0\\ \arctan\left(\frac{y}{x}\right)+180{}^{\circ}, & x<0\;and\;y\geq 0\\ \arctan\left(\frac{y}{x}\right)-180{}^{\circ}, & x<0\;and\;y<0\\ sign(y)\cdot 90{}^{\circ}, & x=0\;and\;y\neq 0\\ undefined, & x=0\;\;and\;y=0 \end{array}\right.\;.$$

In accordance with convolution (24) and the superposition principle, in steady state, *H*_div_(*jω*) will affect each frequency component *V*_k_cos(*ω*_k_*t* + *φ*_k_) of the input signal *v*_surface_ in such a way that it will scale its amplitude into *V*_k_·|*H*_div_(*jω*_k_)| and shift its phase by ∡*H*_div_(*jω*_k_) [268]. In this section, |*H*_div_(*jω*)|_dB_ will be preferred for denoting the magnitude response.

**Figure 16:**
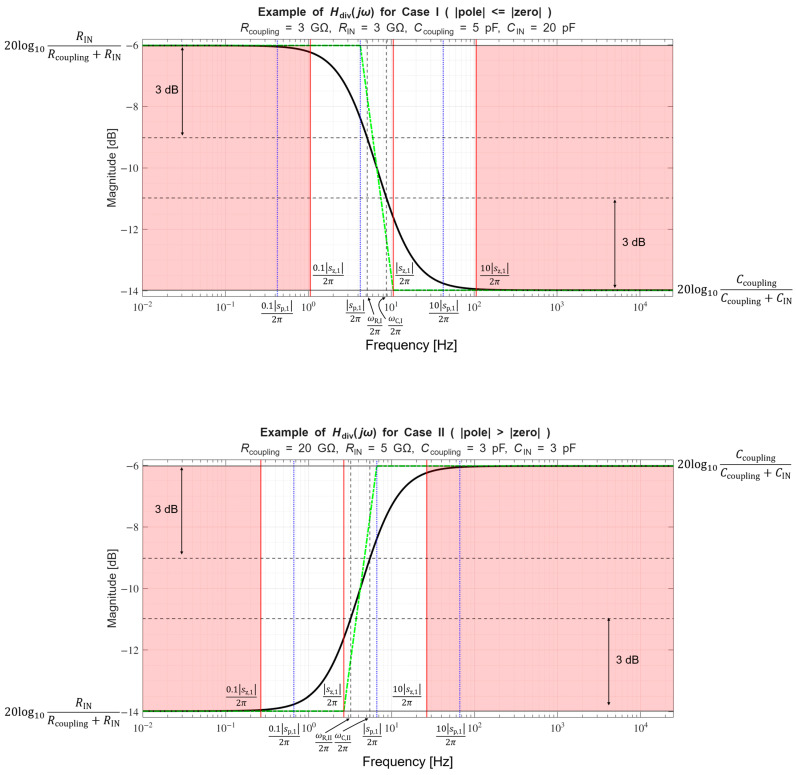
Magnitude response |*H*_div_(*jω*)|_dB_ when |*s*_p,1_| ≤ |*s*_z,1_| (case I) and |*s*_p,1_| > |*s*_z,1_| (case II). Magnitude response is denoted by solid black curves. Vertical thinner solid red lines denote the frequencies arising from the zero: 0.1$\frac{\left|s_{z,1}\right|}{2\pi }$, $\frac{\left|s_{z,1}\right|}{2\pi }$, and 10$\frac{\left|s_{z,1}\right|}{2\pi }$ from the left to the right, respectively. Vertical dotted blue lines denote the frequencies arising from the pole: 0.1$\frac{\left|s_{p,1}\right|}{2\pi }$, $\frac{\left|s_{p,1}\right|}{2\pi }$, and 10$\frac{\left|s_{p,1}\right|}{2\pi }$ from the left to the right, respectively. Vertical dashed black lines denote the pairs of cutoff frequencies: low-end $\frac{\omega_{R,I}}{2\pi }$ and high-end $\frac{\omega_{C,I}}{2\pi }$ in case I, and low-end $\frac{\omega_{R,II}}{2\pi }$ and high-end $\frac{\omega_{C,II}}{2\pi }$ in case II. Horizontal dashed black lines denote the magnitudes of the resistive and capacitive voltage dividers in decibels (dB). Dash-dotted green lines represent the Bode magnitude plot of the respective magnitude response, with break frequencies $\frac{\left|s_{p,1}\right|}{2\pi }$ and $\frac{\left|s_{z,1}\right|}{2\pi }$ from the left to the right in case I, and $\frac{\left|s_{z,1}\right|}{2\pi }$ and $\frac{\left|s_{p,1}\right|}{2\pi }$ from the left to the right in case II. Finally, shaded red areas stretching to the left represent the areas of predominantly resistive coupling, whereas shaded red areas stretching to the right represent the areas of predominantly capacitive coupling. All these parameters will be discussed in detail in the following pages.

**Four subcases.** In Section 3.1, it was discussed that the term capacitive electrode is an oversimplification for most cases of practical biopotential measurement. As an alternative, Test 1 was established as a tool for evaluating the predominant coupling mechanism in each of the coupling layers. Now, the predominant coupling mechanism can be investigated in the context of the generalized buffer active non-contact and/or insulated electrode and its interface with the body, presented in Figure 10. In that sense, Test 1 can be applied to the total equivalent coupling impedance *Z*_coupling_, which comprises the equivalent *R*_coupling_ and *C*_coupling_ in accordance with Assumption 4. This equivalent *Z*_coupling_ is loaded by the equivalent input impedance *Z*_IN_, which comprises *R*_IN_ and *C*_IN_ in accordance with Assumption 9. The resulting *H*_div_(*jω*) (26) has one pole, *s*_p,1_ = $-\frac{1}{\left(R_{coupling}||R_{IN})(C_{coupling}||C_{IN}\right)}$ (18), and one zero, *s*_z,1_ = $-\frac{1}{R_{coupling}C_{coupling}}$ (19). Hence, two cases can be discerned based on the position of the pole *s*_p,1_ with respect to the position of the zero *s*_z,1_ in the pole–zero plot (Figure 12). For this purpose, absolute value of the pole, |*s*_p,1_| = $\frac{1}{\left(R_{coupling}||R_{IN})(C_{coupling}||C_{IN}\right)}$, and absolute value of the zero, |*s*_z,1_| = $\frac{1}{R_{coupling}C_{coupling}}$, can be compared. Condition |*s*_p,1_| ≤ |*s*_z,1_| yields the first case (case I), whereas |*s*_p,1_| > |*s*_z,1_| yields the second case (case II). The resulting two cases are depicted in Figure 16.

As recalled from (18) and (19), the zero *s*_z,1_ = $-\frac{1}{R_{coupling}C_{coupling}}$ is defined with the combination of values *R*_coupling_ and *C*_coupling_, whereas the pole *s*_p,1_ = $-\frac{1}{\left(R_{coupling}||R_{IN})(C_{coupling}||C_{IN}\right)}$ is defined with the combination of values *R*_coupling_, *R*_IN_, *C*_coupling_, and *C*_IN_. This means that the position of the pole with respect to the position of the zero is determined by the value of the *R*_coupling_/*R*_IN_ ratio with respect to the *C*_IN_/*C*_coupling_ ratio, as shown in (31):

$$\left|s_{p,1}\right|=\frac{1}{\left(R_{coupling}||R_{IN})(C_{coupling}||C_{IN}\right)}=\frac{1}{\frac{R_{coupling}R_{IN}}{R_{coupling}{+R}_{IN}}\left(C_{coupling}+C_{IN}\right)}=$$

(31) $$=\frac{1}{\frac{R_{coupling}C_{coupling}}{\frac{R_{coupling}}{R_{IN}}+1}\left(1+\frac{C_{IN}}{C_{coupling}}\right)}=\left|s_{z,1}\right|\frac{1+\frac{R_{coupling}}{R_{IN}}}{1+\frac{C_{IN}}{C_{coupling}}}\;.$$

The ratio *R*_coupling_/*R*_IN_ can be treated as the attenuation of the resistive voltage divider: the higher the ratio *R*_coupling_/*R*_IN_, the more attenuation is caused by the resistive voltage divider. Similarly, the ratio *C*_IN_/*C*_coupling_ can be treated as the attenuation of the capacitive voltage divider: the higher the ratio *C*_IN_/*C*_coupling_, the more attenuation is caused by the capacitive voltage divider. If |*s*_p,1_| ≤ |*s*_z,1_|, then *C*_IN_/*C*_coupling_ ≥ *R*_coupling_/*R*_IN_ (case I in Figure 16). Otherwise, if |*s*_p,1_| > |*s*_z,1_|, then *C*_IN_/*C*_coupling_ < *R*_coupling_/*R*_IN_ (case II in Figure 16).

Furthermore, two additional conditions are possible: R_IN_ < *R*_coupling_ (subcases 1) and R_IN_ ≥ *R*_coupling_ (subcases 2). These two conditions will determine the extent to which the attenuations of the resistive and capacitive voltage dividers can be manipulated.

In total, combination of the aforementioned conditions gives four subcases (I.1, I.2, II.1, and II.2), which are listed in Table 2. The four parameters of the interface, *R*_coupling_, *R*_IN_, *C*_coupling_, and *C*_IN_, will define the attenuation of the input voltage divider, as well as the position of the pole and its |*s*_p,1_| absolute value. In addition, *R*_coupling_ and *C*_coupling_ will define the position of the zero and its |*s*_z,1_| value. As explained earlier in Figure 12, these absolute values |*s*_z,1_| and |*s*_p,1_| correspond to the distance of the negative, real-valued zero *s*_z,1_ and the negative, real-valued pole *s*_p,1_ from the origin of the complex *s*-plane, respectively. Additionally, |*s*_z,1_| and |*s*_p,1_| also correspond to the zero and the pole angular break frequency in the Bode plot of *H*_div_(*jω*), respectively. Similarly, $\frac{\left|s_{z,1}\right|}{2\pi }$ and $\frac{\left|s_{p,1}\right|}{2\pi }$ denote the corresponding break frequencies in accordance with the relation *ω* = 2π*f* (A2). Shifting the angular break frequencies |*s*_z,1_| and |*s*_p,1_| toward lower frequencies would be translated into moving the zero *s*_z,1_ and the pole *s*_p,1_ toward the origin of the complex *s*-plane. In accordance with Bode approximations [273,341,348,349,397] and the example provided of analysis with Bode plots (Figure A9), |*s*_z,1_| in the term $\left(1+\frac{s}{\left|s_{z,1}\right|}\right)$ is the zero angular break frequency above which the slope of the magnitude response |*H*_div_(*jω*)|_dB_ increases for 20 dB/decade. Accordingly, |*s*_z,1_| introduces leading (positive) phase shifts into ∡*H*_div_(*jω*), with a positive slope of +45°/decade that begins approximately at angular frequency 0.1|*s*_z,1_| and ends approximately at angular frequency 10|*s*_z,1_|, achieving a total of +90° across the two decades. Similarly, |*s*_p,1_| in the term $1/(1+\frac{s}{\left|s_{p,1}\right|})$ is the pole angular break frequency above which the slope of the magnitude response |*H*_div_(*jω*)|_dB_ decreases for 20 dB/decade. Accordingly, |*s*_p,1_| introduces lagging (negative, delaying) phase shifts into ∡*H*_div_(*jω*), with a negative slope of −45°/decade that begins approximately at angular frequency 0.1|*s*_p,1_| and ends approximately at angular frequency 10|*s*_p,1_|, achieving a total of −90° across the two decades. For these reasons, case I (|*s*_p,1_| ≤ |*s*_z,1_|) yields a magnitude response |*H*_div_(*jω*)|_dB_ that can contain a portion descending with increasing frequency (roll-off), whereas case II (|*s*_p,1_| > |*s*_z,1_|) yields a magnitude response |*H*_div_(*jω*)|_dB_ that will always contain a portion ascending with increasing frequency (roll-on). The concept of lagging and leading is illustrated in [397], along with frequency responses of various bilinear transfer functions. In the following pages, all these effects will be inspected in the context of the input voltage divider subsystem of the generalized buffer active non-contact and/or insulated electrode and its interface with the body. Analysis will be further divided into three steps. Analytical discussions will be accompanied by simulation results in MathWorks^®^ MATLAB R2025b environment [146]. To visually separate the discussions in each of the three steps, each step ends with a summarizing paragraph, and the respective figures are given afterward. Lastly, summaries from each of the three steps will be assembled to define guidelines for designing non-contact and insulated electrode–body interfaces.

**STEP 1: Extending the area (frequency band) of predominantly capacitive coupling.** The first interesting observation for each of the four subcases (Figure 16 and Table 2) is the following: since |*s*_z,1_| is equal to $\frac{1}{R_{coupling}C_{coupling}}$ (19), |*s*_z,1_| could serve as a rough transition point from predominantly resistive to predominantly capacitive coupling. Namely, in accordance with Test 1, the coupling mechanism is predominantly resistive up to the angular frequency of 0.1|*s*_z,1_|. Concomitantly, the magnitude at *ω* << min{|*s*_z,1_|, |*s*_p,1_|} (*ω* at least a decade lower than the lowest value in the braces) flattens and the frequency response is predominantly defined by the resistive divider, $\frac{R_{IN}}{R_{coupling}+R_{IN}}\;$or 20log_10_$\frac{R_{IN}}{R_{coupling}+R_{IN}}$ in decibels (32), which provides a conductive path at DC:

$$\underset{\omega \to 0}{\lim}H_{div}\left(j\omega \right)=\underset{\omega \to 0}{lim}\frac{R_{IN}}{R_{coupling}+R_{IN}}\cdot \frac{1+\frac{j\omega }{\frac{1}{R_{coupling}C_{coupling}}}}{1+\frac{j\omega }{\frac{1}{{(R_{coupling}||R}_{IN})(C_{coupling}||C_{IN})}}}$$

(32) $$=\frac{R_{IN}}{R_{coupling}+R_{IN}}\;,$$

Conversely, above the angular frequency of 10|*s*_z,1_|, the coupling mechanism is predominantly capacitive. Concomitantly, the magnitude at *ω* >> max{|*s*_z,1_|, |*s*_p,1_|} (*ω* at least a decade higher than the highest value in the braces) flattens once again and the frequency response is predominantly defined by the capacitive divider, $\frac{C_{coupling}}{C_{coupling}+C_{IN}}\;$or 20log_10_$\frac{C_{coupling}}{C_{coupling}+C_{IN}}$ in decibels (33):

$$\underset{\omega \to \infty }{\lim}H_{div}\left(j\omega \right)=\underset{\omega \to \infty }{lim}\frac{C_{coupling}}{C_{coupling}+C_{IN}}\cdot \frac{j\omega +\frac{1}{R_{coupling}C_{coupling}}}{j\omega +\frac{1}{{(R_{coupling}||R}_{IN})(C_{coupling}||C_{IN})}}\;\;$$

(33) $$=\frac{C_{coupling}}{C_{coupling}+C_{IN}}\;,$$

**Figure 17:**
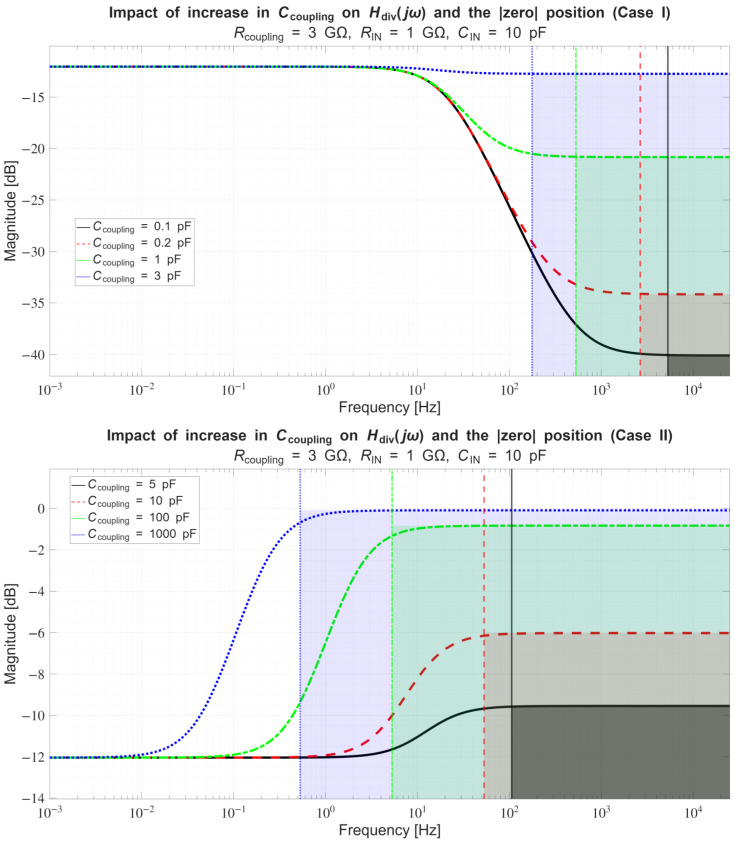
Impact of increased *C*_coupling_ on the magnitude response |*H*_div_(*jω*)|_dB_ for case I (|*s*_p,1_| ≤ |*s*_z,1_|, *C*_IN_/*C*_coupling_ ≥ *R*_coupling_/*R*_IN_) and case II (|*s*_p,1_| > |*s*_z,1_|, *C*_IN_/*C*_coupling_ < *R*_coupling_/*R*_IN_). Vertical lines denote the $\frac{10|s_{z,1}|}{2\pi }\;$frequencies, along with the shaded areas of predominantly capacitive coupling. It can be noted that the magnitude response can contain a roll-off in case I due to |*s*_p,1_| ≤ |*s*_z,1_| and that it contains a roll-on in case II due to |*s*_p,1_| > |*s*_z,1_|.

**Figure 18:**
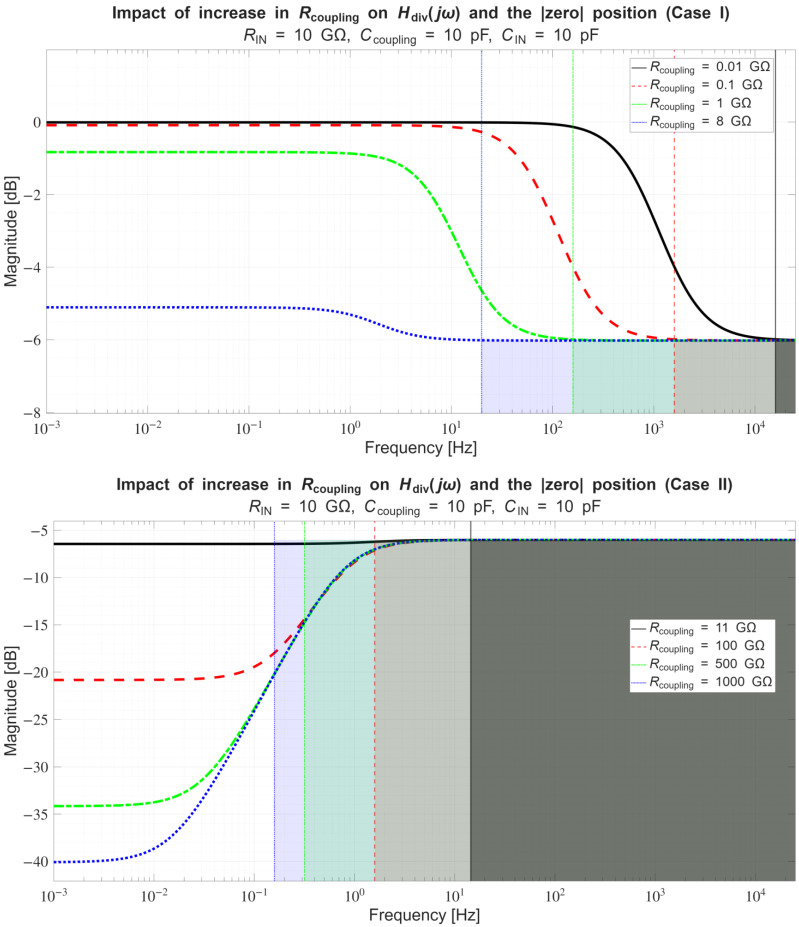
Impact of increased *R*_coupling_ on the magnitude response |*H*_div_(*jω*)|_dB_ for case I (|*s*_p,1_| ≤ |*s*_z,1_|, *C*_IN_/*C*_coupling_ ≥ *R*_coupling_/*R*_IN_) and case II (|*s*_p,1_| > |*s*_z,1_|, *C*_IN_/*C*_coupling_ < *R*_coupling_/*R*_IN_). Vertical lines denote the $\frac{10|s_{z,1}|}{2\pi }\;$frequencies along with the shaded areas of predominantly capacitive coupling. It can be noted that the magnitude response can contain a roll-off in case I due to |*s*_p,1_| ≤ |*s*_z,1_| and that it contains a roll-on in case II due to |*s*_p,1_| > |*s*_z,1_|.

The influence of *C*_coupling_ and *R*_coupling_ on the position of the zero can be observed in Figure 17 and Figure 18, respectively.

Aside from the low-side frequency limit, the high-side frequency limit to predominantly capacitive coupling can also be noted. Since only subcase A.1 from Table 1 is considered for any coupling layer given (Assumption 4), once the angular frequency 10|*s*_z,1_| is reached, coupling stays predominantly capacitive up to the highest frequency of interest (Assumption 1). Thus, for the purpose of biopotential measurement, no high-side frequency limit to the predominantly capacitive coupling area will be considered.

**Figure 19:**
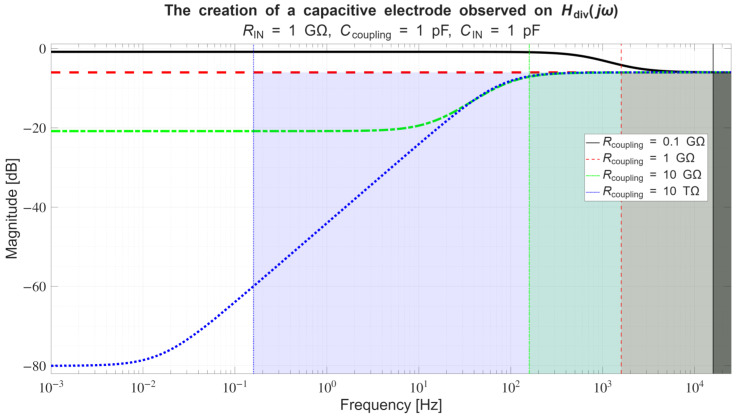
Impact on the magnitude response |*H*_div_(*jω*)|_dB_ when *R*_coupling_ goes to infinity. Vertical lines denote the $\frac{10|s_{z,1}|}{2\pi }\;$frequencies, along with the shaded areas of predominantly capacitive coupling. By increasing *R*_coupling_ for all other parameters fixed, the electrode approaches to the concept of a true pure capacitive electrode with a pronounced high-pass frequency response—the special case of subcase II.1 (|*s*_p,1_| > |*s*_z,1_|, i.e., *C*_IN_/*C*_coupling_ < *R*_coupling_/*R*_IN_, and *R*_IN_ << *R*_coupling_). This will be further investigated in step 2.

**Capacitive electrode.** The existence of a frequency below which the coupling is not predominantly capacitive is the reason why it is often stated that non-contact and insulated electrodes possess an inherent high-pass frequency characteristic, which would lead to the conclusion they could not be used down to DC (0 Hz). This would be true for pure capacitive electrodes (*R*_coupling_ virtually infinite). Indeed, their input stage would be AC-coupled by nature due to the pronounced capacitive character of the coupling impedance and an extremely attenuating resistive voltage divider. Therefore, their *H*_div_(*jω*) (26) would come down to (34) as a special case of subcase II.1 (|*s*_p,1_| > |*s*_z,1_| and *R*_IN_ << *R*_coupling_, Figure 19):

(34) $$H_{div}(j\omega ){|}_{R_{\mathrm{coupling}}\to \infty }=\frac{C_{coupling}}{C_{coupling}+C_{IN}}\cdot \frac{j\omega }{j\omega +\frac{1}{R_{IN}(C_{coupling}||C_{IN})}}\;.$$

However, in most practical cases of on-body measurements, which are preferred for biopotential monitoring, *R*_coupling_ is finite. Therefore, the influence of finite leakage through the electrode–body interface should be taken into account, as discussed in Section 3.1. Also, an increase in *R*_coupling_ is in practice limited by the interface material and ambient conditions. In that sense, the existence of a frequency below which the coupling mechanism is not predominantly capacitive does not necessarily impose a pronounced high-pass behavior, nor does it preclude the usage of non-contact and insulated electrodes below that frequency. Rather, it stresses the methodological issue that has already been commented on in paragraphs “Non-contact on-body electrodes” and “Bias currents and DC biasing” in previous sections: if, e.g., a non-insulated non-contact on-body electrode were used over a moisture-absorbent fabric, *R*_coupling_ could decrease to the order of 100 MΩ or even lower with time, which could cause |*s*_z,1_| = $\frac{1}{R_{coupling}C_{coupling}}$ to increase. This would in turn expand the predominantly resistive coupling area toward higher frequencies. Thus, it would no longer be correct to treat the electrode as predominantly capacitive across the initial frequency range.

**STEP 1: SUMMARY.** To sum up, in accordance with Test 1 and Assumption 4, the area of predominantly capacitive coupling spreads above the angular frequency 10|*s*_z,1_|. Thus, the first step in using active electrodes with a predominantly capacitive coupling is to make *R*_coupling_ and *C*_coupling_ interface parameters sufficiently high so that 10|*s*_z,1_| = $\frac{10}{R_{coupling}C_{coupling}}$ is decreased below the lowest angular frequency of interest (ω_min_). This way, the bandwidth of the measured signal will be entirely situated in the area of predominantly capacitive coupling. On the one hand, a higher *R*_coupling_ will reduce the leakage through the interface. On the other hand, the higher the *C*_coupling_, the closer to short it will appear at frequencies of the measured biopotential signal (A4), which again enhances the capacitive coupling mechanism. In conclusion, in step 1, parameters *R*_coupling_, *C*_coupling_, and the zero *s*_z,1_ are chosen and fixed.

**STEP 2: Manipulating the attenuation of the input voltage divider.** In the previous step, the equivalent coupling impedance *Z*_coupling_, along with its parameters, *R*_coupling_ and *C*_coupling_, was chosen and fixed. Consequently, the zero *s*_z,1_ = $-\frac{1}{R_{coupling}C_{coupling}}$ (19) was chosen and fixed, as well as its angular break frequency |*s*_z,1_| and the area of predominantly capacitive coupling, with its low-side limit at an angular frequency of 10|*s*_z,1_|. Aside from the area of predominantly capacitive coupling, the second important aspect is the magnitude response |*H*_div_(*jω*)|_dB_ and its attenuation, which arises from the resistive and capacitive voltage dividers. To observe their influence, the other two parameters of the interface, *C*_IN_ and *R*_IN_, should be accounted for, along with their effect on the pole *s*_p,1_ = $-\frac{1}{\left(R_{coupling}||R_{IN})(C_{coupling}||C_{IN}\right)}$ (18) and its angular break frequency |*s*_p,1_|. First, the influence of *C*_IN_ and *R*_IN_ on the voltage divider attenuation and the pole position will be inspected. Next, cutoff frequencies will be defined to emphasize the possible distinction between the area of predominantly capacitive coupling and the area of predominant capacitive divider. Lastly, the preferred position of |*s*_p,1_| with respect to the fixed position of |*s*_z,1_| will be observed based on possible configurations of the electrode–body interface: high-pass, all-pass, and low-pass. The summary of the analysis is available at the end of step 2.

**Figure 20:**
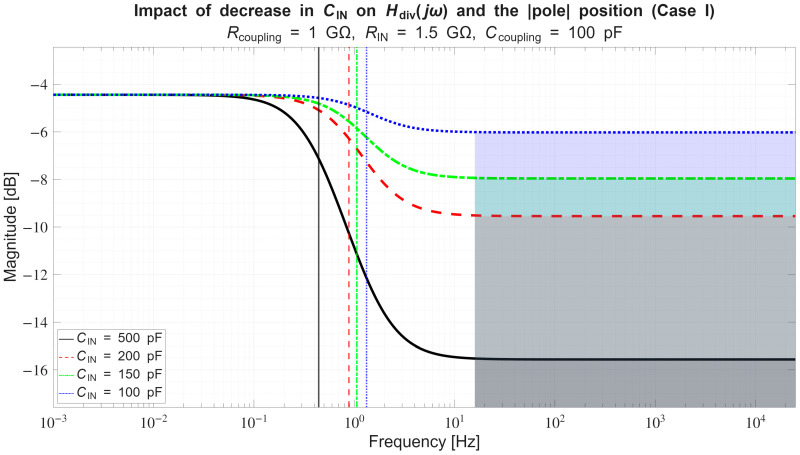

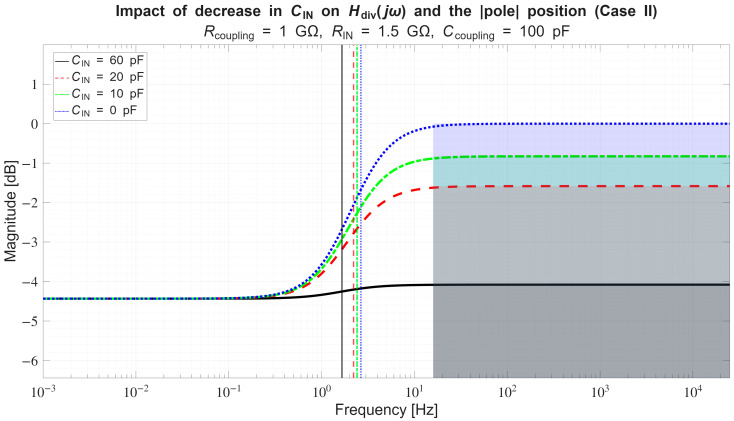
Impact of decreased *C*_IN_ on the magnitude response |*H*_div_(*jω*)|_dB_ for case I (|*s*_p,1_| ≤ |*s*_z,1_|, *C*_IN_/*C*_coupling_ ≥ *R*_coupling_/*R*_IN_) and case II (|*s*_p,1_| > |*s*_z,1_|, *C*_IN_/*C*_coupling_ < *R*_coupling_/*R*_IN_). Vertical lines denote the $\frac{\left|s_{p,1}\right|}{2\pi }\;$pole break frequencies, along with the shaded areas of predominantly capacitive coupling.

**Influence of *C*_IN_.** Firstly, the capacitive voltage divider effect can be tackled. It dominates the magnitude response at *ω* >> max{|*s*_z,1_|, |*s*_p,1_|}, simplifying it to $\frac{C_{coupling}}{C_{coupling}+C_{IN}}$ (33). As seen from the shape of the capacitive divider, since *C*_coupling_ is fixed in step 1, this magnitude level comes down to dependence on *C*_IN_. To minimize the capacitive divider attenuation and raise its magnitude toward the maximum value of 1 (or 0 dB), *C*_IN_ should be 0, i.e., as low as possible. To achieve this, requirement *C*_coupling_ >> *C*_IN_ (i.e., *C*_coupling_ ≥ 10*C*_IN_) should be satisfied. This will reduce the attenuation to 1/11 in the case when *C*_coupling_ = 10*C*_IN_, or to a lower value when *C*_coupling_ > 10*C*_IN_. Therefore, the rest of the analysis in the paper will be carried out with the aim of fulfilling the condition *C*_coupling_ >> *C*_IN_. However, as seen from |*s*_p,1_| = $\frac{1}{\left(R_{coupling}||R_{IN})(C_{coupling}||C_{IN}\right)}$, reducing *C*_IN_ increases |*s*_p,1_| (Figure 20). Ultimately, minimizing *C*_IN_ to the ideal value of 0 would maximize the |*s*_p,1_| value for all other parameters fixed. This and similar interdependencies between the pole and the interface parameters will prove to be of use later in this step.

**Figure 21:**
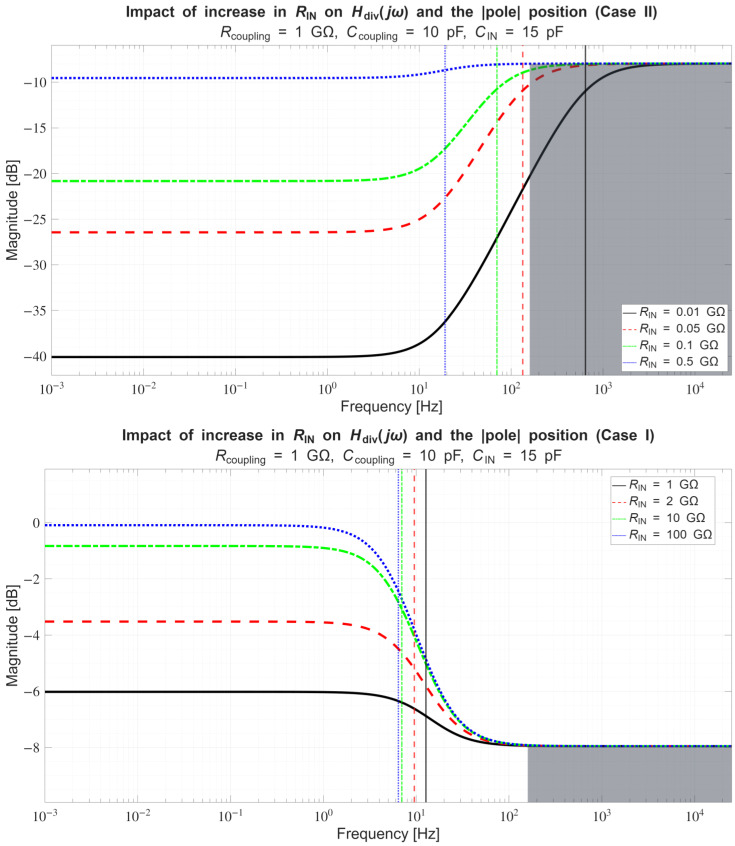
Impact of increased *R*_IN_ on the magnitude response |*H*_div_(*jω*)|_dB_ for case I (|*s*_p,1_| ≤ |*s*_z,1_|, *C*_IN_/*C*_coupling_ ≥ *R*_coupling_/*R*_IN_) and case II (|*s*_p,1_| > |*s*_z,1_|, *C*_IN_/*C*_coupling_ < *R*_coupling_/*R*_IN_). Vertical lines denote the $\frac{\left|s_{p,1}\right|}{2\pi }$ pole break frequencies, along with the shaded areas of predominantly capacitive coupling. Note that the upper figure represents case II, whereas the lower figure represents case I.

**Influence of *R*_IN_.** On the other side of the frequency response, at the lowest frequencies, the resistive voltage divider effect can be investigated. It dominates the magnitude response at *ω* << min{|*s*_z,1_|, |*s*_p,1_|}, simplifying it to $\frac{R_{IN}}{R_{coupling}+R_{IN}}$ (32). As seen from the shape of the resistive divider, since *R*_coupling_ is fixed in step 1, this magnitude level comes down to dependence on the fourth variable of the interface, *R*_IN_. The importance of *R*_IN_ has already been noticeable in Figure 19, where the creation of a pure capacitive electrode was depicted through an increase in *R*_coupling_ (for all other parameters fixed). Therein, once *R*_coupling_ reached the value of 10 GΩ, which was chosen to be 10*R*_IN_, further increase in *R*_coupling_ allowed for a further increase in attenuation of the resistive divider and further extension of the predominantly capacitive coupling area toward lower frequencies, but the angular frequency |*s*_p,1_| = $\frac{1}{\left(R_{coupling}||R_{IN})(C_{coupling}||C_{IN}\right)}$ remained unchanged because it became dominated by *R*_IN_ << *R*_coupling_. This influence of *R*_IN_ on the pole *s*_p,1_ can be generalized: for a given *R*_coupling_ and *C*_coupling_ (step 1) and for the chosen *C*_IN_, lower *R*_IN_ would decrease the *R*_coupling_||*R*_IN_ parallel, increasing the value of |*s*_p,1_|. This means that the highest possible |*s*_p,1_| for a given *R*_coupling_ and *C*_coupling_ would be achieved when both *C*_IN_ and *R*_IN_ are minimized. In other words, the by-product of achieving the least attenuating capacitive voltage divider and the most attenuating resistive voltage divider would be maximization of |*s*_p,1_|. Conversely, a higher *R*_IN_ increases the *R*_coupling_||*R*_IN_ parallel, lowering the value of |*s*_p,1_| = $\frac{1}{\left(R_{coupling}||R_{IN})(C_{coupling}||C_{IN}\right)}$ for the chosen *C*_IN_ (Figure 21).

**Figure 22:**
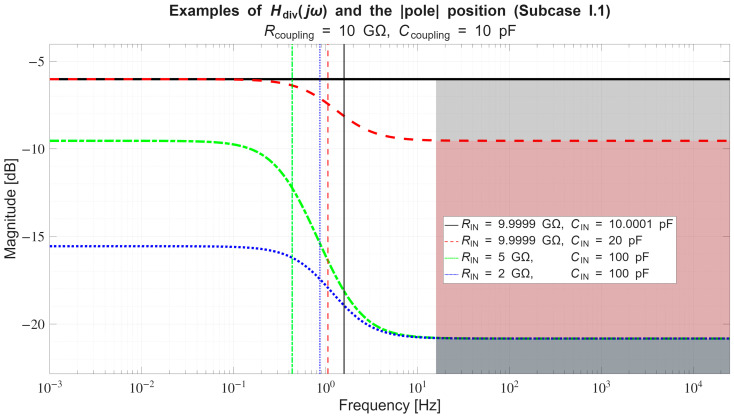

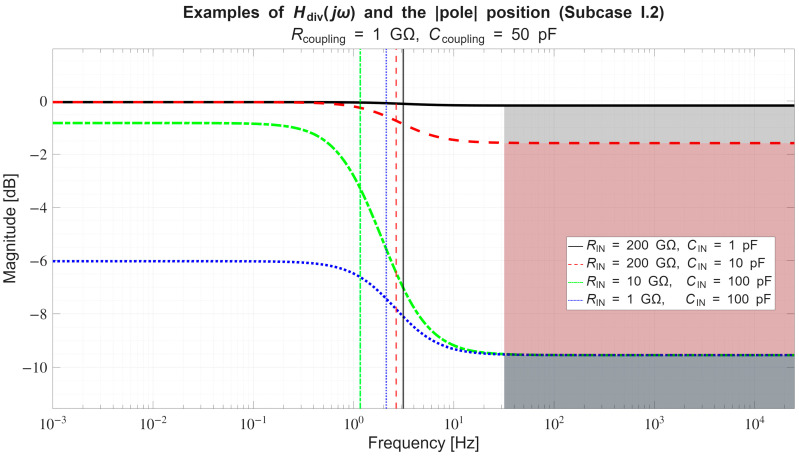
Mutual impact of *R*_IN_ and *C*_IN_ on the magnitude response |*H*_div_(*jω*)|_dB_ for case I (|*s*_p,1_| ≤ |*s*_z,1_|, *C*_IN_/*C*_coupling_ ≥ *R*_coupling_/*R*_IN_): subcase I.1 (*R*_IN_ < *R*_coupling_) and subcase I.2 (*R*_IN_ ≥ *R*_coupling_). Vertical lines denote the $\frac{\left|s_{p,1}\right|}{2\pi }\;$pole break frequencies, along with the shaded areas of predominantly capacitive coupling. It can be noted that the magnitude response can contain a roll-off due to |*s*_p,1_| ≤ |*s*_z,1_|. Also, the achieved magnitude levels of the resistive voltage divider, 20log_10_$\frac{R_{IN}}{R_{coupling}+R_{IN}}$, are below 20log_10_0.5 ≈ −6 dB (50% or more attenuation) in subcase I.1 (*R*_IN_ < *R*_coupling_), and above −6 dB (50% or less attenuation) in subcase I.2 (*R*_IN_ ≥ *R*_coupling_).

**Figure 23:**
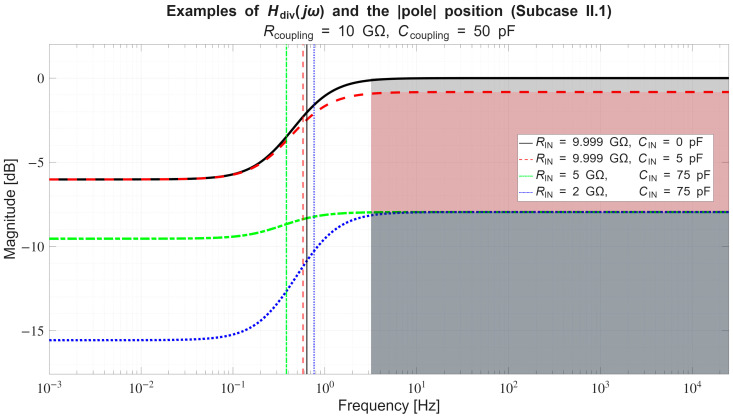

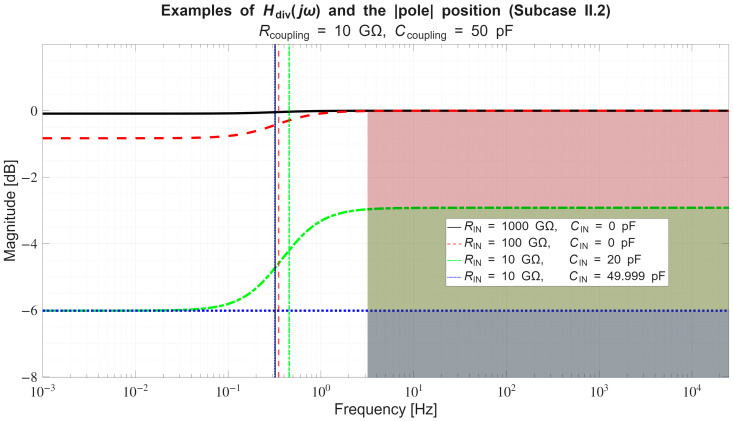
Mutual impact of *R*_IN_ and *C*_IN_ on the magnitude response |*H*_div_(*jω*)|_dB_ for case II (|*s*_p,1_| > |*s*_z,1_|, *C*_IN_/*C*_coupling_ < *R*_coupling_/*R*_IN_): subcase II.1 (*R*_IN_ < *R*_coupling_) and subcase II.2 (*R*_IN_ ≥ *R*_coupling_). Vertical lines denote the $\frac{\left|s_{p,1}\right|}{2\pi }\;$pole break frequencies, along with the shaded areas of predominantly capacitive coupling. It can be noted that the magnitude response contains a roll-on due to |*s*_p,1_| > |*s*_z,1_|. Also, the achieved magnitude levels of the resistive voltage divider, 20log_10_$\frac{R_{IN}}{R_{coupling}+R_{IN}}$, are below 20log_10_0.5 ≈ −6 dB (50% or more attenuation) in subcase II.1 (*R*_IN_ < *R*_coupling_), and above −6 dB (50% or less attenuation) in subcase II.2 (*R*_IN_ ≥ *R*_coupling_).

To further analyze the influence of *R*_IN_, subcases 1 and 2 determined by the *R*_coupling_/*R*_IN_ ratio can be included in the analysis. As shown earlier in Figure 16, case I (|*s*_p,1_| ≤ |*s*_z,1_|) results in a magnitude response that can contain a roll-off portion, whereas case II (|*s*_p,1_| > |*s*_z,1_|) results in a magnitude response that certainly contains a roll-on portion. Now going a step further, as shown in Figure 22 for subcase I.1 and I.2, and in Figure 23 for subcase II.1 and II.2, the achieved magnitude levels 20log_10_$\frac{R_{IN}}{R_{coupling}+R_{IN}}$ in subcases 1 (*R*_IN_ < *R*_coupling_) are of values below 20log_10_0.5 ≈ −6 dB (50% attenuation). On the other hand, in subcases 2 (*R*_IN_ ≥ *R*_coupling_), the achieved magnitude levels 20log_10_$\frac{R_{IN}}{R_{coupling}+R_{IN}}$ are greater than or equal to about −6 dB. In that sense, subcases 1 (*R*_IN_ < *R*_coupling_) represent the cases of aggressive resistive voltage divider (50% or more attenuation) and allow a maximization of resistive divider attenuation. On the contrary, subcases 2 (*R*_IN_ ≥ *R*_coupling_) allow a minimization of the resistive voltage divider attenuation (50% or less attenuation). The rough boundary between subcases 1 and subcases 2 is the −6 dB magnitude level (50% attenuation) at the low-frequency end.

**Limitations on minimizing *C*_IN_.** In previous paragraphs, influence of *C*_IN_ and *R*_IN_ on the voltage attenuation and the pole position were addressed separately. Now, the combined effect of the capacitive and the resistive voltage divider can be examined to inspect how the achievable values of the *C*_IN_/*C*_coupling_ ratio are affected by the *R*_coupling_/*R*_IN_ ratio. For each of the four subcases and for a fixed *R*_coupling_, *C*_coupling_, and |*s*_z,1_|, a change in *R*_IN_ and *C*_IN_ corresponds to two effects: first, a vertical shift in the magnitude of the resistive and capacitive voltage dividers, 20log_10_$\frac{R_{IN}}{R_{coupling}+R_{IN}}$ and 20log_10_$\frac{C_{\mathrm{coupling}}}{C_{\mathrm{coupling}}+C_{\mathrm{IN}}}$ respectively, and second, a horizontal shift in the |*s*_p,1_| angular break frequency. However, the range of achievable values for *C*_IN_ and *R*_IN_ might not be unconstrained: only in subcase II.1 (*C*_IN_/*C*_coupling_ < *R*_coupling_/*R*_IN_ and *R*_IN_ < *R*_coupling_) can *C*_IN_ be chosen to be as low as possible for any given value of *R*_coupling_, *R*_IN_, and *C*_coupling_ for which the subcase is applicable (subcase II.1 in Figure 23). In the rest of the subcases, a decrease in the *C*_IN_/*C*_coupling_ ratio is limited by the *R*_coupling_/*R*_IN_ ratio. Specifically, prerequisites for subcase I.1, *C*_IN_/*C*_coupling_ ≥ *R*_coupling_/*R*_IN_ and *R*_IN_ < *R*_coupling_, lead to the requirement *C*_IN_ > *C*_coupling_. This means that in subcase I.1, both the capacitive voltage divider 20log_10_$\frac{C_{coupling}}{C_{coupling}+C_{IN}}$ and the resistive voltage divider 20log_10_$\frac{R_{IN}}{R_{coupling}+R_{IN}}$ attenuate at least 50% of the input signal. As a result, the magnitude response |*H*_div_(*jω*)|_dB_ in subcase I.1 is entirely situated below the −6 dB magnitude level. Therefore, subcase I.1 is not a practical solution for biopotential measurement (subcase I.1 in Figure 22). On the other hand, in subcase I.2 (*C*_IN_/*C*_coupling_ ≥ *R*_coupling_/*R*_IN_ and *R*_IN_ ≥ *R*_coupling_), condition *C*_coupling_ = 10*C*_IN_ requires *R*_IN_ ≥ 10*R*_coupling_. To generalize this with the use of a positive real factor *T* ≥ 10, condition *C*_coupling_ = *T*·*C*_IN_ requires *R*_IN_ ≥ *T*·*R*_coupling_. In other words, in subcase I.2, a less attenuating resistive divider (lower *R*_coupling_/*R*_IN_ ratio) allows for the realization of a less attenuating capacitive divider (lower *C*_IN_/*C*_coupling_ ratio). Specifically, since *R*_coupling_ and *C*_coupling_ are fixed in step 1, this means that a higher *R*_IN_ allows us to achieve a lower *C*_IN_ (subcase I.2 in Figure 22). Lastly, in subcase II.2 (*C*_IN_/*C*_coupling_ < *R*_coupling_/*R*_IN_ and *R*_IN_ ≥ *R*_coupling_), condition *C*_coupling_ = 10*C*_IN_ is feasible when *R*_IN_ < 10*R*_coupling_. To generalize this once again with the use of a positive real factor *T* ≥ 10, condition *C*_coupling_ = *T*·*C*_IN_ is feasible whenever *R*_IN_ < *T*·*R*_coupling_. In other words, minimization of the capacitive voltage divider attenuation imposes a limit on the minimization of the resistive voltage divider attenuation (subcase II.2 in Figure 23).

**Cutoff frequencies and the area of predominant divider.** Aside from observing the magnitude levels of the resistive and capacitive voltage dividers, it is also interesting to observe the frequencies at which these magnitude levels are reached with respect to the areas of predominant coupling mechanisms. In particular, the area in which the magnitude response |*H*_div_(*jω*)|_dB_ simplifies to the capacitive divider 20log_10_$\frac{C_{coupling}}{C_{coupling}+C_{IN}}$ (the area of predominant capacitive divider) (33) can be observed with respect to the area of predominantly capacitive coupling. Similarly, the area in which the magnitude response |*H*_div_(*jω*)|_dB_ simplifies to the resistive divider 20log_10_$\frac{R_{IN}}{R_{coupling}+R_{IN}}$ (the area of predominant resistive divider) (32) can be observed with respect to the area of predominantly resistive coupling. So far, it has been stated that the area of predominant resistive divider corresponds to angular frequencies *ω* << min{|*s*_z,1_|, |*s*_p,1_|}, and that the area of predominant capacitive divider corresponds to angular frequencies *ω* >> max{|*s*_z,1_|, |*s*_p,1_|}. Instead of using vague mathematical definitions, these frequency limits can be estimated with the use of conventional cutoff frequencies, as introduced in Appendix D and [347]. For the case when |*s*_p,1_| ≤ |*s*_z,1_| (case I, Figure 16), the magnitude response |*H*_div_(*jω*)|_dB_ can contain a roll-off; hence, *ω*_R,I_ at low-end frequencies can be defined as the angular frequency at which the descending |*H*_div_(*jω*)|_dB_ falls to a value that is 3 dB below 20log_10_$\frac{R_{IN}}{R_{coupling}+R_{IN}}$ (35,36):

$$CASE\;I:\frac{R_{IN}}{R_{coupling}+R_{IN}}\cdot \frac{\sqrt{1+{\left(\omega_{R,I}R_{coupling}C_{coupling}\right)}^{2}}}{\sqrt{1+{\left[\omega_{R,I}{(R_{coupling}||R}_{IN})(C_{coupling}||C_{IN})\right]}^{2}}}$$

(35) $$\approx \frac{1}{\sqrt{2}}\cdot \frac{R_{IN}}{R_{coupling}+R_{IN}},$$

$$\omega_{R,I}=\frac{1}{\sqrt{{\left[{(R_{coupling}||R}_{IN})(C_{coupling}||C_{IN})\right]}^{2}-2{\left(R_{coupling}\cdot C_{coupling}\right)}^{2}}}$$

(36) $$=\frac{1}{\sqrt{{\left(\frac{1}{s_{p,1}}\right)}^{2}-2{\left(\frac{1}{s_{z,1}}\right)}^{2}}}.$$

At the high-end side, *ω*_C,I_ in case I can be defined as the angular frequency at which the descending |*H*_div_(*jω*)|_dB_ falls to a value that is 3 dB above 20log_10_$\frac{C_{coupling}}{C_{coupling}+C_{IN}}$ (37,38):

$$CASE\;I:\frac{C_{coupling}}{C_{coupling}+C_{IN}}\cdot \frac{\sqrt{{\omega^{2}}_{C,I}+{\left(\frac{1}{R_{coupling}C_{coupling}}\right)}^{2}}}{\sqrt{{\omega^{2}}_{C,I}+{\left(\frac{1}{{(R_{coupling}||R}_{IN})(C_{coupling}||C_{IN})}\right)}^{2}}}$$

(37) $$\approx \sqrt{2}\cdot \frac{C_{coupling}}{C_{coupling}+C_{IN}}\;\;,$$

$$\omega_{C,I}=\sqrt{{\left[\frac{1}{{(R_{coupling}||R}_{IN})(C_{coupling}||C_{IN})}\right]}^{2}-2{\left(\frac{1}{R_{coupling}C_{coupling}}\right)}^{2}\;\;}$$

(38) $$=\sqrt{{s_{z,1}}^{2}-2{s_{p,1}}^{2}}\;\;.$$

This pair of cutoff frequencies *ω*_R,I_ and *ω*_C,I_ for case I exists whenever (39) is satisfied:

(39) $$CASE\;I:{{|s}_{p,1}|\leq \omega }_{R,I},\omega_{C,I}\leq \left|s_{z,1}\right|\to \frac{\left|s_{z,1}\right|}{\left|s_{p,1}\right|}=\frac{1+\frac{C_{IN}}{C_{coupling}}}{1+\frac{R_{coupling}}{R_{IN}}}\geq \sqrt{3}\;.$$

**Figure 24:**
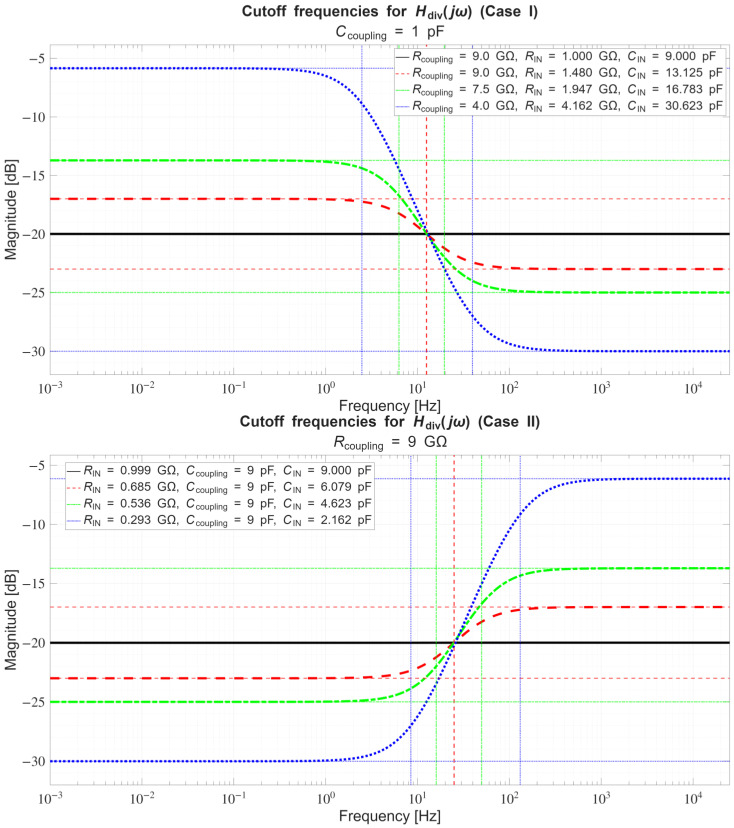
Magnitude response |*H*_div_(*jω*)|_dB_ along with the pairs of cutoff frequencies (vertical lines): *ω*_R,I_ and *ω*_C,I_ in case I (|*s*_p,1_| ≤ |*s*_z,1_|, *C*_IN_/*C*_coupling_ ≥ *R*_coupling_/*R*_IN_), and *ω*_R,II_ and *ω*_C,II_ in case II (|*s*_p,1_| > |*s*_z,1_|, *C*_IN_/*C*_coupling_ < *R*_coupling_/*R*_IN_). In these graphs, the existence of cutoff frequencies and their relative position with respect to *C*_IN_/*C*_coupling_ and *R*_coupling_/*R*_IN_ ratios is inspected. The first example in each of the two cases (solid black line) depicts a scenario in which there are no cutoff frequencies. Accordingly, the difference between magnitude levels of the resistive and capacitive voltage dividers is less than 3 dB. Also, the ratio |*s*_z,1_|/|*s*_p,1_| in case I or, equivalently, the ratio |*s*_p,1_|/|*s*_z,1_| in case II, is lower than $\sqrt{3}$. The other three examples in each of the two cases are arbitrarily chosen to illustrate the interdependence between the relative position of voltage divider magnitude levels, 20log_10_$\frac{R_{IN}}{R_{coupling}+R_{IN}}$ and 20log_10_$\frac{C_{coupling}}{C_{coupling}+C_{IN}}$, and the relative position of angular break frequencies |*s*_z,1_| and |*s*_p,1_| and cutoff frequencies in a pair. Starting from the scenario in which the difference between magnitude levels of the resistive and capacitive voltage dividers is 6 dB and the cutoff frequencies are equal (dashed red line examples), greater difference between the cutoff frequencies sets the magnitude levels of the voltage dividers, as well as the angular break frequencies, further apart.

The resulting calculations are summarized in Table 2 and visualized in Figure 24 (case I).

Similarly, in the case when |*s*_p,1_| > |*s*_z,1_| (case II, Figure 16), the magnitude response |*H*_div_(*jω*)|_dB_ contains a roll-on; hence, *ω*_R,II_ at low-end frequencies can be defined as the angular frequency at which the ascending |*H*_div_(*jω*)|_dB_ increases to a value that is 3 dB above 20log_10_$\frac{R_{IN}}{R_{coupling}+R_{IN}}$ (40,41):

$$CASE\;II:\frac{R_{IN}}{R_{coupling}+R_{IN}}\cdot \frac{\sqrt{1+{\left(\omega_{R,II}R_{coupling}C_{coupling}\right)}^{2}}}{\sqrt{1+{\left[\omega_{R,II}{(R_{coupling}||R}_{IN})(C_{coupling}||C_{IN})\right]}^{2}}}$$

(40) $$\approx \sqrt{2}\cdot \frac{R_{IN}}{R_{coupling}+R_{IN}},$$

$$\omega_{R,II}=\frac{1}{\sqrt{{\left(R_{coupling}\cdot C_{coupling}\right)}^{2}-2{\left[{(R_{coupling}||R}_{IN})(C_{coupling}||C_{IN})\right]}^{2}}}$$

(41) $$=\frac{1}{\sqrt{{\left(\frac{1}{s_{z,1}}\right)}^{2}-2{\left(\frac{1}{s_{p,1}}\right)}^{2}}}.$$

At the high-end side, *ω*_C,II_ in case II can be defined as the angular frequency at which the ascending |*H*_div_(*jω*)|_dB_ increases to a value that is 3 dB below $20{log}_{10}\frac{C_{coupling}}{C_{coupling}+C_{IN}}$ (42,43):

$$CASE\;II:\frac{C_{coupling}}{C_{coupling}+C_{IN}}\cdot \frac{\sqrt{{\omega^{2}}_{C,II}+{\left(\frac{1}{R_{coupling}C_{coupling}}\right)}^{2}}}{\sqrt{{\omega^{2}}_{C,II}+{\left(\frac{1}{{(R_{coupling}||R}_{IN})(C_{coupling}||C_{IN})}\right)}^{2}}}$$

(42) $$\approx \frac{1}{\sqrt{2}}\cdot \frac{C_{coupling}}{C_{coupling}+C_{IN}}\;\;,$$

$$\omega_{C,II}=\sqrt{{\left[\frac{1}{{(R_{coupling}||R}_{IN})(C_{coupling}||C_{IN})}\right]}^{2}-2{\left(\frac{1}{R_{coupling}C_{coupling}}\right)}^{2}\;\;}$$

(43) $$=\sqrt{{s_{p,1}}^{2}-2{s_{z,1}}^{2}}\;\;.$$

This pair of cutoff frequencies *ω*_R,II_ and *ω*_C,II_ for case II exists whenever (44) is satisfied:

(44) $$CASE\;II:\;|s_{z,1}|{\leq \omega }_{R,II},\omega_{C,\mathrm{II}}\leq {|s}_{p,1}|\to \;\frac{\left|s_{p,1}\right|}{\left|s_{z,1}\right|}=\frac{1+\frac{R_{coupling}}{R_{IN}}}{1+\frac{C_{IN}}{C_{coupling}}}\geq \sqrt{3}\;.$$

The resulting calculations are again summarized in Table 2 and visualized in Figure 24 (case II).

**Area of predominantly capacitive coupling with respect to the area of predominant capacitive divider.** Unlike the 10|*s*_z,1_| value, which determines the low-side frequency of the predominantly capacitive coupling area (step 1), conventional angular cutoff frequencies *ω*_C,I_ and *ω*_C,II_ determine the low-side frequency of the predominant capacitive divider area with a 3 dB tolerance. In case I, the condition |*s*_p,1_| ≤ |*s*_z,1_| is valid, hence |*s*_z,1_| ≥ *ω*_C,I_. Also, 10|*s*_z,1_| > *ω*_C,I_. Thus, in case I, the area of predominantly capacitive coupling implies the area of predominant capacitive divider. Conversely, in case II, the condition |*s*_p,1_| > |*s*_z,1_| is valid. Therefore, *ω*_C,II_ might be higher than 10|*s*_z,1_| and, consequently, the area of predominantly capacitive coupling would begin at lower frequencies than the area of predominant capacitive divider. Specifically, for this condition *ω*_C,II_ > 10|*s*_z,1_| to occur, requirement |*s*_p,1_| > $\sqrt{102}$|*s*_z,1_| should be fulfilled (43), which can be achieved only in subcase II.1 (*C*_IN_/*C*_coupling_ < *R*_coupling_/*R*_IN_ and *R*_IN_ < *R*_coupling_) (44). The existence of this special case reveals why it is important to keep track of both |*s*_z,1_| and |*s*_p,1_| values and also track both the area of predominantly capacitive coupling and the area of predominant capacitive divider with respect to the bandwidth of the measured signal. This will be further discussed in step 3.

**Interdependence of the difference between the pole and the zero and the difference between the magnitude levels of the resistive and capacitive voltage dividers.** The use of the derived pairs of angular cutoff frequencies, *ω*_R,I_ and *ω*_C,I_ for case I, and *ω*_R,II_ and *ω*_C,II_ for case II, is three-fold (Figure 24). First, *ω*_R,I_ and *ω*_R,II_ estimate the high-side frequency of the predominant resistive divider area, whereas *ω*_C,I_ and *ω*_C,II_ estimate the low-side frequency of the predominant capacitive divider area, as explained in previous paragraphs. Second, the difference between the cutoff frequencies in a pair indicates the extent of the difference between the magnitude levels 20log_10_$\frac{R_{IN}}{R_{coupling}+R_{IN}}$ and 20log_10_$\frac{C_{\mathrm{coupling}}}{C_{\mathrm{coupling}}+C_{\mathrm{IN}}}$, as well as the extent of their vertical distance in the magnitude response. Third, the difference between the cutoff frequencies in a pair also indicates the extent of the difference between the zero *s*_z,1_ and the pole *s*_p,1_ in the complex *s*-plane, as well as the extent of the difference between their corresponding angular break frequencies |*s*_z,1_| and |*s*_p,1_|. Accordingly, whenever a pair of these angular cutoff frequencies exists, which is determined by (39) and (44) respectively, the difference between the magnitude levels of the resistive and capacitive voltage dividers will be at least 3 dB, and one of the values |*s*_z,1_| and |*s*_p,1_| will be at least $\sqrt{3}$ greater than the other. Naturally, if the difference between the respective magnitude levels is at least 3 dB, but less than 6 dB, cutoff frequencies will overlap (*ω*_R,I_ > *ω*_C,I_ and *ω*_R,II_ > *ω*_C,II_). Once the difference becomes exactly 6 dB, cutoff frequencies in a pair will be equal (*ω*_R,I_ = *ω*_C,I_ and *ω*_R,II_ = *ω*_C,II_), as in dashed red examples in Figure 24. As the difference between the magnitude levels is further increased, conditions *ω*_C,I_ > *ω*_R,I_ and *ω*_C,II_ > *ω*_R,II_ are valid. Moreover, an interdependency exists: the further apart the pole and the zero are set from one another, the greater the difference between the magnitude levels of the dividers. This is in accordance with the Bode approximation of contribution of *s*_z,1_ and *s*_p,1_ to the magnitude response, depicted in Figure A9: above angular break frequency |*s*_z,1_|, the slope of |*H*_div_(*jω*)|_dB_ increases for 20 dB/decade, whereas above |*s*_p,1_|, it decreases for 20 dB/decade. Thus, the greater the difference between *s*_z,1_ and *s*_p,1_, the greater the achieved shift in magnitude between |*s*_z,1_| and |*s*_p,1_|. This can be observed separately for case I, which is based on the |*s*_z,1_|/|*s*_p,1_| ratio (39), and for case II, which is based on the |*s*_p,1_|/|*s*_z,1_| ratio (44). To inspect the interdependence of the increasing difference between angular break frequencies |*s*_z,1_| and |*s*_p,1_|, and the increasing difference between the magnitude levels of the dividers, the magnitude response can be observed when the ratios in (39) and (44) are increased. Specifically, in case I (|*s*_p,1_| ≤ |*s*_z,1_|), the ratio is |*s*_z,1_|/|*s*_p,1_|. To increase it for a fixed value of |*s*_z,1_|, *s*_p,1_ should be moved away from *s*_z,1_ toward the origin of the complex *s*-plane, i.e., |*s*_p,1_| should be reduced. As seen in (39), the ratio |*s*_z,1_|/|*s*_p,1_| can be increased by decreasing *R*_coupling_/*R*_IN_ and by increasing *C*_IN_/*C*_coupling_. For a fixed *R*_coupling_ and *C*_coupling_, this means that both *R*_IN_ and *C*_IN_ should be increased, which is in accordance with the minimization of |*s*_p,1_| = $\frac{1}{\left(R_{coupling}||R_{IN})(C_{coupling}||C_{IN}\right)}$. Graphically, an increase in *R*_IN_ corresponds to an upward shift of the resistive divider level 20log_10_$\frac{R_{IN}}{R_{coupling}+R_{IN}}$, whereas an increase in *C*_IN_ corresponds to a downward shift of the capacitive divider level 20log_10_$\frac{C_{\mathrm{coupling}}}{C_{\mathrm{coupling}}+C_{\mathrm{IN}}}$ (case I in Figure 24). In total, with an increase in *R*_IN_ and *C*_IN_, |*s*_p,1_| decreases, |*s*_p,1_| and |*s*_z,1_| are further separated, and the realized magnitude response will have a more pronounced roll-off (low-pass characteristic). On the other hand, in case II (|*s*_p,1_| > |*s*_z,1_|), the ratio is |*s*_p,1_|/|*s*_z,1_|. To increase it for a fixed value of |*s*_z,1_|, *s*_p,1_ should be once again moved away from *s*_z,1_, but this time away from the origin of the complex *s*-plane, so its value |*s*_p,1_| should now be increased. As seen in (44), the ratio |*s*_p,1_|/|*s*_z,1_| can be increased by increasing *R*_coupling_/*R*_IN_ and by decreasing *C*_IN_/*C*_coupling_. For a fixed *R*_coupling_ and *C*_coupling_, this means that both *R*_IN_ and *C*_IN_ should be decreased, which is in accordance with the maximization of |*s*_p,1_| = $\frac{1}{\left(R_{coupling}||R_{IN})(C_{coupling}||C_{IN}\right)}$. Graphically, a decrease in *R*_IN_ corresponds to a downward shift of the resistive divider level 20log_10_$\frac{C_{\mathrm{coupling}}}{C_{\mathrm{coupling}}+C_{\mathrm{IN}}}$, whereas a decrease in *C*_IN_ corresponds to an upward shift of the capacitive divider level 20log_10_$\frac{C_{\mathrm{coupling}}}{C_{\mathrm{coupling}}+C_{\mathrm{IN}}}$ (case II in Figure 24). In total, with a decrease in *R*_IN_ and *C*_IN_, |*s*_p,1_| increases, |*s*_z,1_| and |*s*_p,1_| are further separated, and the realized magnitude response will have a more pronounced roll-on (high-pass characteristic). These effects will be applied in the following paragraphs to explore the feasibility of practical configurations of the interface between the body and a non-contact and/or insulated biopotential electrode.

**High-pass configuration.** Finally, the previous findings can be used to further inspect the practicality and usability of the specific subcases. Intuitively, the design of non-contact and insulated electrodes and their interface with the body could strive for the concept of a purely capacitive electrode (34), as depicted earlier in Figure 19. In that case, the electrode would be treated as a high-pass, AC-coupling filter with the predominantly resistive coupling area as the stopband and the predominantly capacitive coupling area as the passband. This means that the attenuation of the capacitive voltage divider should be minimized (minimum *C*_IN_/*C*_coupling_), whereas the attenuation of the resistive voltage divider should be maximized (maximum *R*_coupling_/*R*_IN_). For a fixed *R*_coupling_ and *C*_coupling_, this requires a minimization of both *C*_IN_ and *R*_IN_. To allow a roll-on in the magnitude response, case II (|*s*_p,1_| > |*s*_z,1_|, *C*_IN_/*C*_coupling_ < *R*_coupling_/*R*_IN_, Figure 16 and Figure 23) is desirable. Appropriately, *ω*_C,II_ (43) can be used as the low-side cutoff frequency of the system. Accordingly, when *ω*_C,II_ > *ω*_R,II_, i.e., when $20{log}_{10}\frac{C_{\mathrm{coupling}}}{C_{\mathrm{coupling}}+C_{\mathrm{IN}}}-20{log}_{10}\frac{R_{IN}}{R_{coupling}+R_{IN}}$ is greater than 6 dB, the frequency band between *ω*_R,II_ and *ω*_C,II_ can be treated as the transition band. In addition, separating the |*s*_p,1_| value from the |*s*_z,1_| value is desirable to achieve a greater distinction in magnitude levels between the passband and the stopband region. More specifically, since |*s*_p,1_| > |*s*_z,1_| in case II, |*s*_p,1_| = $\frac{1}{\left(R_{coupling}||R_{IN})(C_{coupling}||C_{IN}\right)}$ should be increased, which is in accordance with the reduction in *R*_IN_ and *C*_IN_. For this reason, subcase II.1 would be preferred (*R*_IN_ < *R*_coupling_, Figure 23), since it allows for fulfilling the requirement *C*_coupling_ >> *C*_IN_ and at the same time maximizing the ratio *R*_coupling_/*R*_IN_ without any limitations. An example of a high-pass configuration design can be found in [380]. However, a caveat arising from the discussions in the previous paragraphs should be kept in mind. Namely, for subcase II.1, it can be recalled that if |*s*_p,1_| < $\sqrt{102}$|*s*_z,1_|, the condition 10|*s*_z,1_| > *ω*_C,II_ is valid. Under such circumstances, the area of predominant capacitive divider as the passband is not entirely situated in the predominantly capacitive coupling area, so it would be particularly erroneous to treat the electrode as capacitive. In other words, |*s*_p,1_| in the high-pass configuration for approximating the pure capacitive electrode should be at least $\sqrt{102}$ times higher than |*s*_z,1_|. In Figure 19, this is achieved in the dotted blue example. However, an increase in |*s*_p,1_| increases *ω*_C,II_ (43) and decreases *ω*_R,II_ (41). Thus, the higher the |*s*_p,1_| value with respect to |*s*_z,1_|, the closer the electrode to a pure capacitive electrode, but the wider the transition band in its frequency response. Because of this, such tendencies to create a sharp distinction between magnitude levels in the area of predominantly resistive coupling and the area of predominantly capacitive coupling could limit the use of such pronouncedly capacitive biopotential electrodes in clinical-grade applications, where sensing the sub-Hz frequencies could be important for accurate diagnostics. This will be further discussed in step 3 in terms of phase response.

**All-pass configuration.** Aside from the high-pass configuration, another option is to treat the electrode and the electrode–body interface as an all-pass system, but again with the objective of maximizing the area of predominantly capacitive coupling and the area of predominant capacitive divider. For this purpose, keeping the pole *s*_p,1_ as close as possible to the zero *s*_z,1_ (i.e., bringing the |*s*_p,1_| value closer to the |*s*_z,1_| value) would be desirable to reduce the difference between magnitude levels of the resistive and capacitive voltage dividers. Ideally, if the pole *s*_p,1_ were canceled with the zero *s*_z,1_, the levels of attenuation from the resistive and capacitive voltage dividers would be equal and there would be no cutoff frequencies. The frequency response *H*_div_(*jω*) would come down to a constant level of attenuation as in (45)

(45) $$H_{div}\left(j\omega \right){|}_{s_{p,1}=s_{z,1}}=\frac{R_{IN}}{R_{coupling}+R_{IN}}=\frac{C_{coupling}}{C_{coupling}+C_{IN}}\;.$$

For this condition *s*_z,1_ = *s*_p,1_ to happen, ratios *C*_IN_/*C*_coupling_ and *R*_coupling_/*R*_IN_ should be equal (46). This is not surprising, since this derived relation between |*s*_p,1_| and |*s*_z,1_| (46) has shown that the input voltage divider effect is the reason why a pole exists in the first place. Specifically, if the ratios *C*_IN_/*C*_coupling_ and *R*_coupling_/*R*_IN_ were minimized and equal to zero, the achieved gain of the magnitude response would be raised toward its maximum value of unity (0 dB). This shows that for an all-pass configuration of the interface, attenuation of both the capacitive and the resistive voltage divider should be minimized. For a fixed *R*_coupling_ and *C*_coupling_, this means that minimization of *C*_IN_ and maximization of *R*_IN_ is beneficial. To inspect which subcases can be used for the all-pass configuration of the electrode–body interface, each of the four subcases can be further observed in terms of the preferable pole position and in terms of fulfilling the requirement *C*_coupling_ >> *C*_IN_. For this purpose, the result from (31) can be recalled (46) and rewritten:

(46) $$\left|s_{p,1}\right|=\left|s_{z,1}\right|\frac{1+\frac{R_{coupling}}{R_{IN}}}{1+\frac{C_{IN}}{C_{coupling}}}=\left|s_{z,1}\right|\frac{\frac{C_{coupling}}{C_{coupling}+C_{IN}}}{\frac{R_{IN}}{R_{IN}+R_{coupling}}}=\left|s_{z,1}\right|\frac{R_{IN}+R_{coupling}}{R_{IN}}\frac{C_{coupling}}{C_{coupling}+C_{IN}}\;.$$

**Figure 25:**
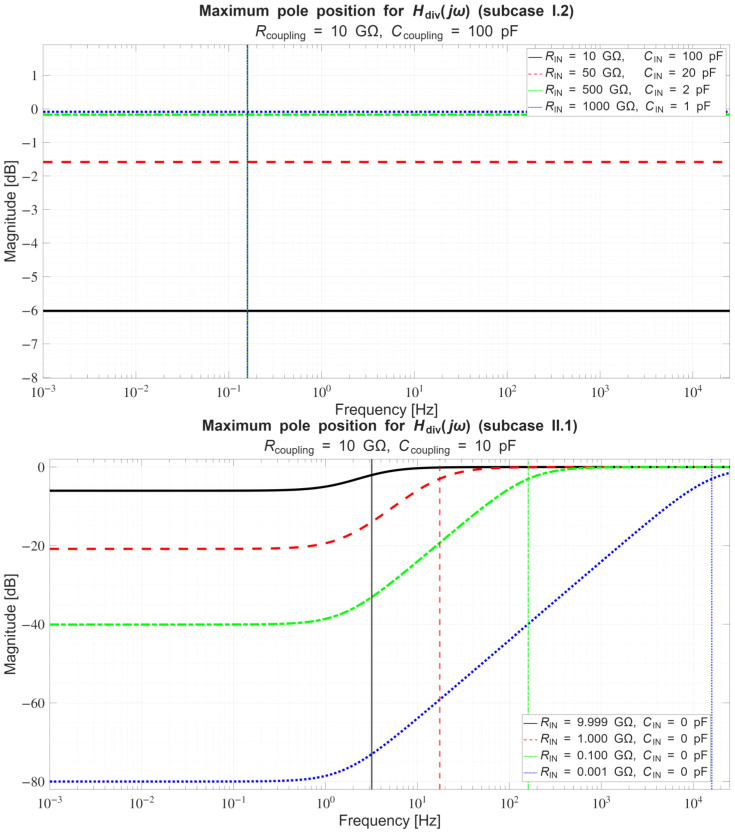

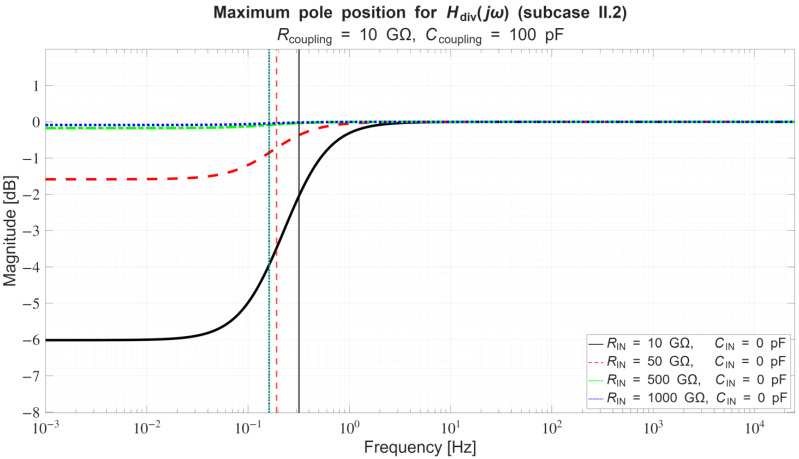
Positions of maximum |*s*_p,1_|, denoted by vertical dashed lines, with respect to the minimum *C*_IN_ and *R*_IN_ achievable in each of the three practical subcases: subcase I.2 (|*s*_p,1_| ≤ |*s*_z,1_|, *C*_IN_/*C*_coupling_ ≥ *R*_coupling_/*R*_IN_, and *R*_IN_ ≥ *R*_coupling_), subcase II.1 (|*s*_p,1_| > |*s*_z,1_|, *C*_IN_/*C*_coupling_ < *R*_coupling_/*R*_IN_, and *R*_IN_ < *R*_coupling_), and subcase II.2 (|*s*_p,1_| > |*s*_z,1_|, *C*_IN_/*C*_coupling_ < *R*_coupling_/*R*_IN_, and *R*_IN_ ≥ *R*_coupling_).

To use case I (|*s*_p,1_| ≤ |*s*_z,1_|, *C*_IN_/*C*_coupling_ ≥ *R*_coupling_/*R*_IN_, Figure 16 and Figure 22) for the all-pass configuration, |*s*_p,1_| value should be maximized. Specifically, only subcase I.2 (*R*_IN_ ≥ *R*_coupling_, Figure 22) enables the desired requirement *C*_coupling_ >> *C*_IN_ to be fulfilled. In fact, this is the only subcase that allows combinations *C*_IN_/*C*_coupling_ = *R*_coupling_/*R*_IN_, which would truly yield |*s*_p,1_| = |*s*_z,1_| and an all-pass configuration with a constant level of attenuation. To further reduce the attenuation and raise the flattened magnitude response, *C*_IN_/*C*_coupling_ and *R*_coupling_/*R*_IN_ can be equally decreased. For a fixed *R*_coupling_ and *C*_coupling_, the requirement *C*_coupling_ = 10*C*_IN_ would call for *R*_IN_ ≥ 10*R*_coupling_. Generally, for a positive real factor *T* ≥ 10, requirement *C*_coupling_ = *T*·*C*_IN_ would call for *R*_IN_ ≥ *T*·*R*_coupling_. In the ideal case of *C*_IN_ = 0 and infinite *R*_IN_, unity gain would be achieved. In other words, although maximizing |*s*_p,1_| = $\frac{1}{\left(R_{coupling}||R_{IN})(C_{coupling}||C_{IN}\right)}$ is desirable in subcase I.2, maximizing *R*_IN_ is still beneficial because it allows for further decrease in *C*_IN_ (subcase I.2 in Figure 25).

On the other hand, case II (|*s*_p,1_| > |*s*_z,1_|, *C*_IN_/*C*_coupling_ < *R*_coupling_/*R*_IN_, Figure 16 and Figure 23) can be considered for the all-pass configuration. The difference with respect to case I is that the |*s*_p,1_| value should now be minimized. Minimizing *C*_IN_ increases |*s*_p,1_| = $\frac{1}{\left(R_{coupling}||R_{IN})(C_{coupling}||C_{IN}\right)}$, but maximizing *R*_IN_ compensates for this increase. Therefore, minimum *C*_IN_ and maximum *R*_IN_ can still be the preferred choice of parameters. Specifically, in subcase II.1 (*R*_IN_ < *R*_coupling_, Figure 23), requirement *C*_coupling_ >> *C*_IN_ can be fulfilled without any limitations. The lowest *C*_IN_ is 0, and the highest achievable *R*_IN_ is slightly lower than *R*_coupling_. In that case, the achieved |*s*_p,1_| is slightly higher than 2|*s*_z,1_| (46), and this is the closest that |*s*_p,1_| can be to |*s*_z,1_| in subcase II.1. For *C*_IN_ = 0, value |*s*_p,1_| would further increase toward $\frac{1}{R_{IN}C_{coupling}}$ as *R*_IN_ is decreased to 0 (46) (subcase II.1 in Figure 25). This shows once again how *s*_p,1_ becomes dominated by *R*_IN_ when *R*_coupling_ >> *R*_IN_ and *C*_coupling_ >> *C*_IN_. Lastly, in subcase II.2 (*R*_IN_ ≥ *R*_coupling_, Figure 23), minimization of *C*_IN_ limits the maximization of *R*_IN_. In particular, requirement *C*_coupling_ = 10*C*_IN_ calls for *R*_IN_ < 10*R*_coupling_. Generally, for a positive real factor *T* ≥ 10, requirement *C*_coupling_ = *T*·*C*_IN_ calls for *R*_IN_ < *T*·*R*_coupling_. However, this does not pose a severe issue on the large scale: the lowest achievable *C*_IN_ is 0, and to achieve it, maximum *R*_IN_ can be any finite number higher than *R*_coupling_. Therefore, |*s*_p,1_| could theoretically be decreased to a value that is infinitesimally larger than |*s*_z,1_|. For *C*_IN_ = 0, as *R*_IN_ is decreased to its minimum (*R*_IN_ = *R*_coupling_), |*s*_p,1_| would increase toward 2|*s*_z,1_| (46) (subcase II.2 in Figure 25).

Now, the three subcases I.2, II.1, and II.2 can be compared to determine which one is preferred for the all-pass configuration. In case II, |*s*_p,1_| > |*s*_z,1_|, so |*s*_p,1_| can never be equal to |*s*_z,1_|, and as a result, a roll-on will always be present in the magnitude response. On the other hand, in subcase I.2, |*s*_p,1_| ≤ |*s*_z,1_|, so |*s*_p,1_| can theoretically be equal to |*s*_z,1_|. However, if it is not, then a roll-off is present in the magnitude response, which would reduce the gain in the area of predominantly capacitive coupling. Also, minimization of *C*_IN_ down to 0 is feasible, but it must be accompanied by maximization of *R*_IN_ toward infinity. In particular, if a positive real factor *T* ≥ 10 is used once again, requirement *C*_coupling_ = *T*·*C*_IN_ calls for *R*_IN_ ≥ *T*·*R*_coupling_, and whenever these two conditions are fulfilled, |*s*_z,1_|/|*s*_p,1_| ratio is lower than $\sqrt{3}$ (39). Consequently, cutoff frequencies do not exist, and the difference between the magnitude levels of the resistive and capacitive voltage dividers is less than 3 dB. On the contrary, in subcase II.1, minimization of *C*_IN_ is unconstrained. However, because *R*_IN_ < *R*_coupling_, attenuation of the resistive voltage divider is at least 50% (roughly −6 dB), so whenever *C*_coupling_ ≥ 10*C*_IN_ is fulfilled, which corresponds to 20log_10_$\frac{C_{coupling}}{C_{coupling}+C_{IN}}$ ≥ −0.8 dB, cutoff frequencies exist. Namely, in accordance with (44), cutoff frequencies in subcase II.1 do not exist when (47) is satisfied:

(47) $$R_{IN}>\frac{R_{coupling}}{\sqrt{3}(1+\frac{C_{IN}}{C_{coupling}})-1}\;.$$

Under requirement *C*_coupling_ ≥ 10*C*_IN_, this would require *R*_IN_ > *R*_coupling_, which is in contradiction with the prerequisite for subcase II.1 (*R*_IN_ < *R*_coupling_). In the ideal case when *C*_IN_ would be minimized to 0, the smallest achievable difference between magnitude levels of the resistive and capacitive voltage dividers would still be roughly 6 dB. Therefore, although minimization of *C*_IN_ in subcase II.1 is unconstrained, it will always yield the existence of cutoff frequencies. Lastly, in subcase II.2, for a positive real factor *T* ≥ 10, requirement *C*_coupling_ = *T*·*C*_IN_ calls for *R*_IN_ < *T*·*R*_coupling_, and whenever these two conditions are fulfilled, the |*s*_p,1_|/|*s*_z,1_| ratio is lower than $\sqrt{3}$, cutoff frequencies do not exist, and the difference between the magnitude levels of the resistive and capacitive voltage dividers is less than 3 dB (44). Compared to subcase I.2, the constraint on minimizing *C*_IN_ and maximizing *R*_IN_ is now relaxed, because minimization of *C*_IN_ down to 0 can be achieved for any finite *R*_IN_. In conclusion, among the three subcases feasible for the all-pass configuration (subcase I.2, II.1, II.2), subcase II.2 is the preferred one, since it allows us to bring the pole in the vicinity of the zero without the possibility for creating a roll-off, whilst relaxing the constraints on the minimization of *C*_IN_ and maximization of *R*_IN_. Once again, since |*s*_p,1_| > |*s*_z,1_| in case II, both the area of predominantly capacitive coupling and the area of predominant capacitive divider should be checked and ensured.

**Low-pass configuration.** Naturally, the third option—a low-pass configuration—can be discussed. This configuration is a conceptual inverse of the high-pass configuration: it would use the area of predominantly resistive coupling as the passband and the area of predominantly capacitive coupling as the stopband. In that sense, maximization of both *R*_IN_ and *C*_IN_ would be preferred for a fixed *R*_coupling_ and *C*_coupling_. Using subcase I.2 (|*s*_p,1_| ≤ |*s*_z,1_| and *R*_IN_ ≥ *R*_coupling_, Figure 22) would be desirable to allow for a roll-off in the magnitude response without imposing an upper limit to the increase in *R*_IN_. Appropriately, *ω*_R,I_ (36) would be used as the high-side cutoff frequency of the system, and the frequency band between *ω*_R,I_ and *ω*_C,I_ would be treated as the transition band whenever *ω*_C,I_ > *ω*_R,I_. However, it can be seen that this configuration would favor the resistive character of the coupling impedance *Z*_coupling_ and that it would require measures opposite to the ones that have been preferred so far. In particular, it requires the extension of the predominantly resistive coupling area toward higher frequencies, the maximization of *R*_IN_ and *C*_IN_, and the minimization of *R*_coupling_ and *C*_coupling_. Therefore, it contradicts the rationale behind using the non-contact and insulated electrodes, and it would be rather the preferred choice for non-insulated surface-contact electrodes. Nevertheless, as discussed in paragraphs “Non-contact on-body electrodes” and “Bias currents and DC biasing” and step 1, the electrode–body interface for non-contact and insulated electrodes could be inadvertently driven into this configuration in the events of decreased *R*_coupling_ or *C*_coupling_.

**STEP 2: SUMMARY.** By choosing *R*_coupling_ and *C*_coupling_ in step 1, the equivalent coupling impedance *Z*_coupling_ was fixed, as well as the zero *s*_z,1_ and its angular break frequency |*s*_z,1_|. Now, in step 2, the other two parameters of the interface were investigated: *C*_IN_ and *R*_IN_, which define the equivalent input impedance *Z*_IN_. The derived relation between |*s*_p,1_| and |*s*_z,1_| (31,46) revealed that the existence of the pole *s*_p,1_ is a direct consequence of the input voltage divider effect. Therefore, parameters *C*_IN_ and *R*_IN_ have been shown to affect not only the attenuation of the resistive and capacitive voltage dividers (20log_10_$\frac{R_{IN}}{R_{coupling}+R_{IN}}$ and 20log_10_$\frac{C_{coupling}}{C_{coupling}+C_{IN}}$), but also the pole *s*_p,1_ and its angular break frequency |*s*_p,1_|. Based on Bode approximation of contribution of *s*_z,1_ and *s*_p,1_ to the magnitude response, an interdependence was discovered: greater difference between |*s*_z,1_| and |*s*_p,1_| draws a greater difference between the magnitude levels of the resistive and capacitive voltage dividers, and vice versa. This was quantified by pairs of cutoff frequencies: *ω*_R,I_ and *ω*_C,I_ for case I (36,38,39), and *ω*_R,II_ and *ω*_C,II_ for case II (41,43,44). In general, while 10|*s*_z,1_| defined the low-side frequency of the predominantly capacitive coupling area (step 1), *ω*_C,I_ and *ω*_C,II_ define the low-side frequency of the predominant capacitive divider area. Non-contact and insulated biopotential electrodes rely on predominantly capacitive coupling, hence they should be used in both specified areas. To determine the frequency range across which these two areas overlap, each of the four established subcases was investigated separately. For this purpose, two essential differences between the subcases were identified and visualized on curves of the magnitude response |*H*_div_(*jω*)|_dB_. The first difference can be used to distinguish case I from case II: in case I, |*H*_div_(*jω*)|_dB_ can contain a roll-off due to |*s*_p,1_| ≤ |*s*_z,1_|, whereas in case II, it contains a roll-on due to |*s*_p,1_| >| *s*_z,1_|. The second difference can be used to distinguish subcases 1 from subcases 2: in subcases 1, the achieved magnitude levels of the resistive voltage divider, 20log_10_$\frac{R_{IN}}{R_{coupling}+R_{IN}}$, are of values below 20log_10_0.5 ≈ −6 dB (50% or more attenuation) due to *R*_IN_ < *R*_coupling_, whereas in subcases 2, they are of values above −6 dB (50% or less attenuation) due to *R*_IN_ ≥ *R*_coupling_. These characteristics were further observed in the context of two specified areas for each of the four subcases. Specifically, subcase I.1 (|*s*_p,1_| ≤ |*s*_z,1_|, *C*_IN_/*C*_coupling_ ≥ *R*_coupling_/*R*_IN_, and *R*_IN_ < *R*_coupling_) is not a practical solution for biopotential measurement, since both the resistive and capacitive voltage dividers attenuate at least 50% of the input signal. Therefore, the analysis continued for subcases I.2, II.1, and II.2. In case I (|*s*_p,1_| ≤ |*s*_z,1_|, *C*_IN_/*C*_coupling_ ≥ *R*_coupling_/*R*_IN_) and subcase II.2 (|*s*_p,1_| > |*s*_z,1_|, *C*_IN_/*C*_coupling_ < *R*_coupling_/*R*_IN_, and *R*_IN_ ≥ *R*_coupling_), the range *ω* ≥ 10|*s*_z,1_| ensures that both the predominantly capacitive coupling area and the predominant capacitive divider area are used. However, in subcase II.1 (|*s*_p,1_| > |*s*_z,1_|, *C*_IN_/*C*_coupling_ < *R*_coupling_/*R*_IN_, and *R*_IN_ < *R*_coupling_), the area of predominant capacitive coupling does not imply the area of predominant capacitive divider if |*s*_p,1_| > $\sqrt{102}$|*s*_z,1_|. In that case, the range *ω* ≥ *ω*_C,II_ ensures that both specified areas are used. This special case shows why it is important to check the values of both |*s*_z,1_| and |*s*_p,1_| and also track both the predominantly capacitive coupling area and the predominant capacitive divider area with respect to the bandwidth of the measured signal. Finally, based on the favorable position of *s*_p,1_ with respect to the fixed *s*_z,1_, possible configurations of the electrode–body interface were investigated. As a tool for relocating the pole *s*_p,1_, values of ratios *R*_coupling_/*R*_IN_ and *C*_IN_/*C*_coupling_ were manipulated. The first analyzed configuration—high-pass configuration, relies on the concept of a purely capacitive electrode (34). This is typical for low-leakage and high-*R*_coupling_ interfaces, such as those achieved with insulated electrodes, which allow very high *R*_coupling_ in the order of 1 TΩ and *C*_coupling_ in the order of 1 nF (refer to paragraph “Insulated electrodes”). Hence, this configuration strives to maximize the resistive divider attenuation (*R*_coupling_/*R*_IN_) and minimize the capacitive divider attenuation (*C*_IN_/*C*_coupling_). For this purpose, achieving a roll-on in the magnitude response and moving *s*_p,1_ away from *s*_z,1_ is beneficial. Thus, subcase II.1 (|*s*_p,1_| > |*s*_z,1_|, *C*_IN_/*C*_coupling_ < *R*_coupling_/*R*_IN_, and *R*_IN_ < *R*_coupling_) is preferred. The value |*s*_p,1_| = $\frac{1}{\left(R_{coupling}||R_{IN})(C_{coupling}||C_{IN}\right)}$ should be increased, and hence, for a fixed *R*_coupling_ and *C*_coupling_, both *R*_IN_ and *C*_IN_ should be minimized. In doing so, whenever *R*_coupling_ >> *R*_IN_ and *C*_coupling_ >> *C*_IN_ is achieved, |*s*_p,1_| simplifies to $\frac{1}{R_{IN}C_{coupling}}$. In general, the further apart |*s*_p,1_| is set from |*s*_z,1_|, the greater the difference between the magnitude levels of the resistive and capacitive voltage dividers, and the closer the electrode to the concept of a pure capacitive electrode (Figure 19). However, this comes at the cost of an expanded transition band (decreased *ω*_R,II_ and increased *ω*_C,II_). As a result, issues of achieving accurate sensing at sub-Hz frequencies arise, which will be further corroborated in step 3. On the other hand, the low-pass configuration poses opposite requirements, and so subcase I.2 (|*s*_p,1_| ≤ |*s*_z,1_|, *C*_IN_/*C*_coupling_ ≥ *R*_coupling_/*R*_IN_, and *R*_IN_ ≥ *R*_coupling_) is preferred. Accordingly, this configuration favors the resistive coupling, so it is desirable for non-insulated surface-contact electrodes. Nevertheless, in the context of non-contact and insulated electrodes, it stresses an important methodological trap of treating non-contact and insulated electrodes as predominantly capacitive, when in fact the realized coupling mechanism at the frequencies of interest is predominantly resistive due to an insufficiently low value of 10|*s*_z,1_| = $\frac{10}{R_{coupling}C_{coupling}}$ (refer to paragraphs “Non-contact on-body electrodes” and “Bias currents and DC biasing”). Finally, the all-pass configuration is an alternative to the high-pass configuration that takes advantage of the finite leakage present at the electrode–body interface. Hence, it strives to flatten and maximize the magnitude response, as illustrated in Figure 25 (subcase I.2). In terms of flattening, bringing *s*_p,1_ close to *s*_z,1_ is beneficial. This comes down to reducing the difference between the ratios *R*_coupling_/*R*_IN_ and *C*_IN_/*C*_coupling_ (46). In terms of maximizing the magnitude response and raising it toward 0 dB, these two ratios should be minimized. In other words, minimization of both the resistive and the capacitive voltage attenuation should be achieved. For a fixed *R*_coupling_ and *C*_coupling_, this means that *R*_IN_ should be maximized and *C*_IN_ minimized. Subcase II.2 (|*s*_p,1_| > |*s*_z,1_|, *C*_IN_/*C*_coupling_ < *R*_coupling_/*R*_IN_, and *R*_IN_ ≥ *R*_coupling_) is preferred, because it allows for relaxed constraints on the simultaneous minimization of *C*_IN_ and maximization of *R*_IN_. Although this configuration strives to maximize *R*_IN_ for a fixed *R*_coupling_, that does not necessarily preclude it from being used with high values of *R*_coupling_ in the order of 100 GΩ and 1 TΩ. Rather, the extent of its utilization for interfaces with high values of *R*_coupling_ is limited by the extent to which *R*_IN_ can be increased with respect to *R*_coupling_. On the other hand, all-pass configuration is particularly interesting in the context of wearable non-contact on-body electrodes, with lower values of *R*_coupling_ in the order of 1 GΩ, 100 MΩ, or even lower, and lower values of *C*_coupling_ in the order of 10 pF and 100 pF. High-pass and all-pass configuration will be further compared considering phase response in step 3.

**STEP 3: Mitigating phase shifts and morphological changes**. In step 1, the area of predominantly capacitive coupling was inspected, and in step 2, it was accompanied by the area of predominant capacitive divider. Finally, aside from ensuring predominantly capacitive coupling in step 1 and improving the magnitude response by manipulating the influence of input voltage divider in step 2, the last important aspect is the phase response ∡*H*_div_(*jω*). Once again, analysis can start from engineering fundamentals. The summary of the analysis is available at the end of step 3.

**Linearity of the phase response.** As is known from the technical literature [399,400,401], to minimize time-domain distortion, the phase response of the system should be linear. Namely, a linear phase response will ensure that individual frequency components are delayed by the same amount of time, and hence that the signal shape and its temporal characteristics are preserved at the output. From the phasor perspective, *ω* is the rate of change in phase Δ*φ* over a time interval Δ*t*. This means that in steady state, phase shift Δ*φ*_k_ between a frequency component *V*_k_cos(*ω*_k_*t* + *φ*_k_) at the input of the subsystem (*v*_surface_) and the corresponding frequency component *V*_k_·|*H*_div_(*jω*_k_)|cos[*ω*_k_*t* + *φ*_k_ + ∡*H*_div_(*jω*_k_)] at the output of the subsystem (*v*_in_) can be translated into time shift Δ*t*_k_ as follows (48):

(48) $$\omega_{k}=-\frac{∆\varphi_{k}}{∆t_{k}}=-\frac{[\varphi_{k}+∡H_{\mathrm{div}}\left(j\omega_{k}\right)]-\varphi_{k}}{∆t_{k}}\;\to ∆t_{k}=-\frac{∡H_{\mathrm{div}}\left(j\omega_{k}\right)}{\omega_{k}}\;.$$

The negative sign indicates that a lagging phase shift, ∡*H*_div_(*jω*_k_) < 0, will result in a time delay, Δ*t*_k_ > 0. Now, it can be shown that if the phase response ∡*H*_div_(*jω*) were linear with a slope *const*, each input frequency component propagating through the subsystem *H*_div_(*jω*) would be delayed at its output for the same value *const* (49):

(49) $$∆t_{k}=-\frac{∡H_{\mathrm{div}}\left(j\omega_{k}\right)}{\omega_{k}}=-\frac{-const\cdot \omega_{k}}{\omega_{k}}=const\;.$$

**Group delay.** The previously analyzed Δ*t*_k_, also called phase delay, represents the time shift of each frequency component separately. Additionally, group delay *gd* can be calculated to analyze the time shift of the signal envelope. Group delay is equal to the negative gradient of phase response. Consequently, a linear phase response would draw a constant group delay, whereas a constant phase response would draw zero group delay. In that sense, frequency bands or areas of constant and zero group delay could be used to investigate at which frequencies a system can reliably transfer, amplify or filter the input signal. Specifically, group delay *gd*_div_ of the observed subsystem *H*_div_(*jω*) can be calculated from the phase response (29) as follows (50):

$${gd}_{div}(\omega )=-\frac{d\left[∡H_{div}\left(j\omega \right)\right]}{d\omega }=$$

$$=\frac{\left(R_{coupling}||R_{IN})(C_{coupling}||C_{IN}\right)}{1+{\left[\omega (R_{coupling}||R_{IN})(C_{coupling}||C_{IN})\right]}^{2}}-\frac{R_{coupling}C_{coupling}}{1+{\left(\omega R_{coupling}C_{coupling}\right)}^{2}}\;$$

(50) $$=\frac{\left|s_{p,1}\right|}{{s_{p,1}}^{2}+\omega^{2}}-\frac{\left|s_{z,1}\right|}{{s_{z,1}}^{2}+\omega^{2}}\;.$$

**Figure 26:**
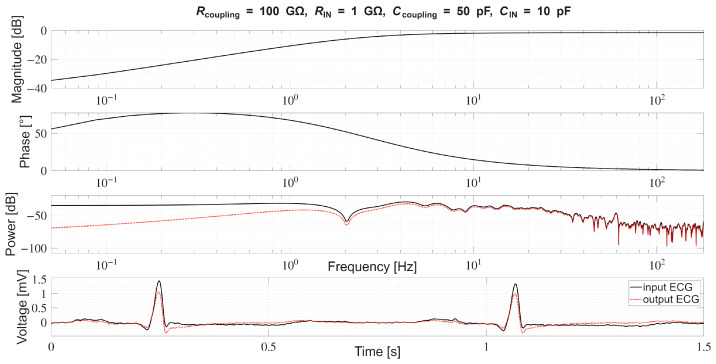

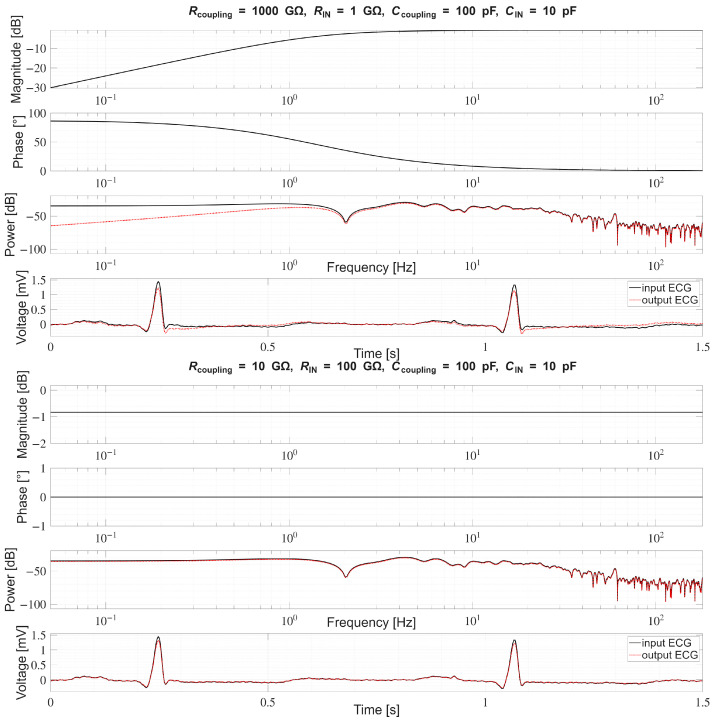
Magnitude response |*H*_div_(*jω*)|_dB_ and phase response ∡*H*_div_(*jω*) of the input voltage divider subsystem, along with an ECG signal at its input and the corresponding response at its output, obtained in MathWorks^®^ MATLAB R2025b environment [146]. Input and output one-channel ECG is given in both the frequency domain (power spectra) and the time domain. Recordings are obtained from the PhysioNet service from the MIT-BIH Arrhythmia database [148]. Signals are recorded at a sampling frequency of 360 Hz and they represent the cardiac disorder of premature ventricular contraction. Results reveal different degrees of changes in signal morphology depending on |*s*_z,1_| and |*s*_p,1_| values defined by the four interface parameters. Three cases are observed: (**upper**) neither *C*_coupling_ >> *C*_IN_ nor *R*_IN_ >> *R*_coupling_ condition is satisfied (high-pass configuration with *R*_coupling_ >> *R*_IN_ and 16%-attenuation of the capacitive voltage divider); (**middle**) high-pass configuration with *R*_coupling_ >> *R*_IN_ and *C*_coupling_ >> *C*_IN_; (**lower**) all-pass configuration with *R*_IN_ >> *R*_coupling_ and *C*_coupling_ >> *C*_IN_.

**Signal morphology issues.** From Section 4.1, it can be recalled that the output of a stable and causal, uniquely solvable LTI system in steady state is a scaled and delayed version of the input. However, phase response ∡*H*_div_(*jω*) (29) is not truly linear, and therefore, group delay *gd*_div_ (50) deviates from a constant value. This indicates that the waveform of the signal *v*_surface_ may not necessarily be preserved after being sensed on the electrode sensing surface and translated to the input of the preamplifier. In other words, the subsystem *H*_div_(*jω*) can indeed only be approximated with an LTI system at narrow bands of frequencies where *gd*_div_ is approximately flat and constant. In fact, the same is true for any analog system, given that the Bode plot is, after all, a linear approximation with a finite error [273,349]. Therefore, the wider the bandwidth of the input signal, the more distortion it could experience. Focusing again on the biopotential measurement, this is especially important to take into account when the feasibility of electrodes in clinical-grade applications is considered. Namely, a non-linear phase response of the measurement system could alter the morphology of the measured biopotential signal and thereby preclude reliable diagnostics. For instance, in the case of clinical ECG measurements, a commonly used low-side frequency limit is 0.05 Hz or even lower, and the influence of the measurement system on near-DC and sub-Hz ECG content, especially ST-segments and T-waves (ST–T changes), but also P-waves, is a well-known issue (refer to [402] and Figure 7). An example can be observed: for this purpose, ECG signals obtained at the input of the simulated subsystem (*v*_surface_ at the surface of the skin) and at the output of the simulated subsystem (*v*_in_ at the preamplifier non-inverting input) can be compared in the time domain. In addition, their power spectra are compared in the frequency domain. Calculations are performed in MathWorks^®^ MATLAB R2025b environment [146] with the use of one-channel ECG signals recorded at a sampling frequency of 360 Hz and obtained from the PhysioNet service from the MIT-BIH Arrhythmia database [148]. Specifically, the results are observed for the case of premature ventricular contraction (Figure 26). Further discussions on this subject in the context of non-contact and insulated electrodes can be found in [403,404,405,406]. More on the morphology of ECG signal and its frequency spectrum can be found in [152,153,154]. Various ECG abnormalities and methods of automatic heart anomaly detection are surveyed in [153,407]. The issue of morphological changes in clinical ECG will be revisited later in paragraph “Practical aspects of DC biasing, feasibility of clinical-grade diagnostics, surface leakage, and dielectric absorption.”

**Figure 27:**
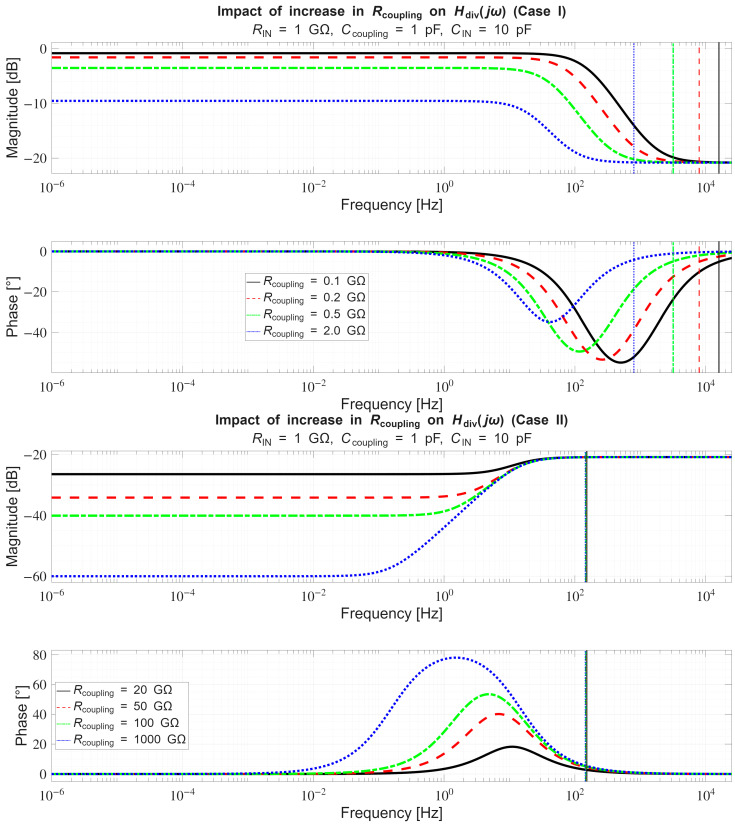
Impact of increased *R*_coupling_ on the magnitude response |*H*_div_(*jω*)|_dB_ and the phase response ∡*H*_div_(*jω*). In the upper figure, vertical lines denote the $10\frac{\left|s_{z,1}\right|}{2\pi }\;$zero frequencies as the low-side limit of the area of minimized phase shifts for case I (|*s*_p,1_| ≤ |*s*_z,1_|, *C*_IN_/*C*_coupling_ ≥ *R*_coupling_/*R*_IN_). In the lower figure, vertical lines denote the $10\frac{\left|s_{p,1}\right|}{2\pi }\;$pole frequencies as the low-side limit of the area of minimized phase shifts for case II (|*s*_p,1_| > |*s*_z,1_|, *C*_IN_/*C*_coupling_ < *R*_coupling_/*R*_IN_).

**Figure 28:**
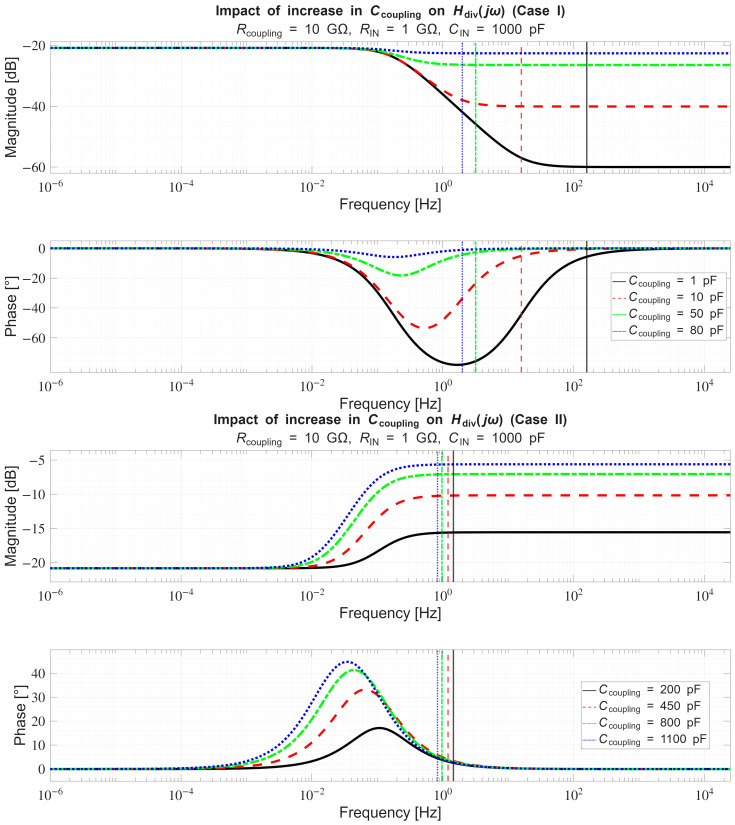
Impact of increased *C*_coupling_ on the magnitude response |*H*_div_(*jω*)|_dB_ and the phase response ∡*H*_div_(*jω*). In the upper figure, vertical lines denote the $10\frac{\left|s_{z,1}\right|}{2\pi }\;$zero frequencies as the low-side limit of the area of minimized phase shifts for case I (|*s*_p,1_| ≤ |*s*_z,1_|, *C*_IN_/*C*_coupling_ ≥ *R*_coupling_/*R*_IN_). In the lower figure, vertical lines denote the $10\frac{\left|s_{p,1}\right|}{2\pi }\;$pole frequencies as the low-side limit of the area of minimized phase shifts for case II (|*s*_p,1_| > |*s*_z,1_|, *C*_IN_/*C*_coupling_ < *R*_coupling_/*R*_IN_).

**Zero and pole phase shifts.** Now that the influence of phase response has been demonstrated, phase response ∡*H*_div_(*jω*) can be inspected more thoroughly. Specifically, entire phase shift created between the skin surface and the input of the preamplifier arises from the pole *s*_p,1_ and the zero *s*_z,1_. As described in Figure A9 with the Bode plot [273,341,348,349,397], the left-half plane pole *s*_p,1_ introduces lagging phase shifts with a negative slope of −45°/decade that begins approximately at angular frequency 0.1|*s*_p,1_| and ends approximately at angular frequency 10|*s*_p,1_|. Similarly, the left-half plane zero *s*_z,1_ introduces leading phase shifts with a positive slope of +45°/decade that begins approximately at angular frequency 0.1|*s*_z,1_| and ends approximately at angular frequency 10|*s*_z,1_|. This means that the phase response ∡*H*_div_(*jω*) will be fairly constant at *ω ≤* min{0.1|*s*_z,1_|, 0.1|*s*_p,1_|} and at *ω* ≥ max{10|*s*_z,1_|, 10|*s*_p,1_|}. However, across the range in between, min{0.1|*s*_z,1_|, 0.1|*s*_p,1_|} < *ω* < max{10|*s*_z,1_|, 10|*s*_p,1_|}, ∡*H*_div_(*jω*) will form a dip if |*s*_p,1_| < |*s*_z,1_| (case I) or a bell-like curve if |*s*_p,1_| > |*s*_z,1_| (case II). This is depicted in Figure 27 and Figure 28, and the same can be further inspected for other types of bilinear transfer functions, as given in [397]. In other words, linearity of ∡*H*_div_(*jω*) cannot be achieved across the entire frequency range of interest (Assumption 1). Rather, it is tied to frequencies that are a decade or less apart from |*s*_z,1_| and |*s*_p,1_| values. Because it is localized, area of linear phase shifts in ∡*H*_div_(*jω*) turns out to be as troublesome as any area of non-linear phase shifts. Therefore, instead of searching for areas of linear phase shifts in ∡*H*_div_(*jω*) and constant group delay *gd*_div_, it is more useful to investigate the areas of approximately constant ∡*H*_div_(*jω*) and near-zero *gd*_div_.

**Figure 29:**
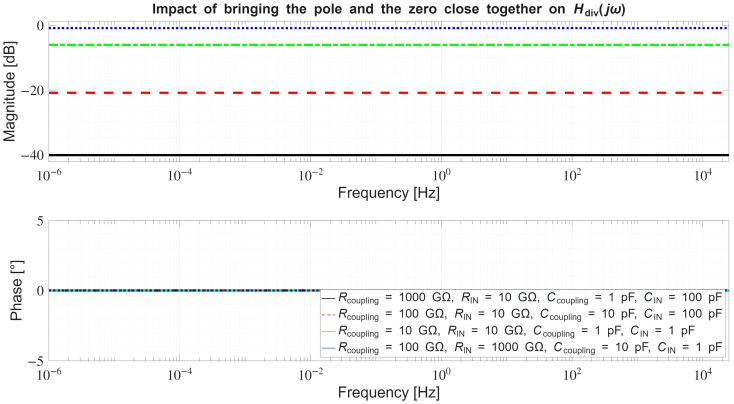
Impact of pole–zero cancelation on the magnitude response |*H*_div_(*jω*)|_dB_ and the phase response ∡*H*_div_(*jω*). When *R*_coupling_/*R*_IN_ = *C*_IN_/*C*_coupling_, |*s*_p,1_| is brought closer to |*s*_z,1_|, and the frequency response flattens out. In addition, as *R*_coupling_/*R*_IN_ and *C*_IN_/*C*_coupling_ ratios decrease to 0, |*H*_div_(*jω*)|_dB_ is raised toward 0 dB.

**Figure 30:**
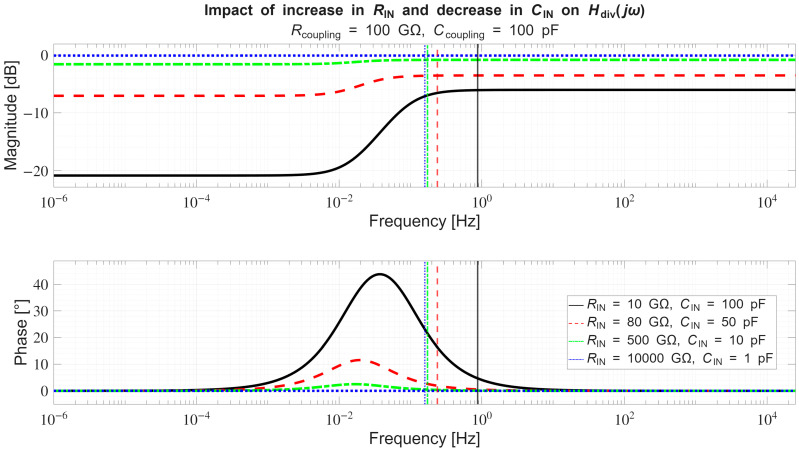
Impact of simultaneous increase in *R*_IN_ and decrease in *C*_IN_ on the magnitude response |*H*_div_(*jω*)|_dB_ and the phase response ∡*H*_div_(*jω*). For *R*_coupling_ and *C*_coupling_ fixed in step 1, decrease in *R*_coupling_/*R*_IN_ and *C*_IN_/*C*_coupling_ ratios can be achieved by minimization of *C*_IN_ and maximization of *R*_IN_. In addition to minimizing phase shifts, this also maximizes the magnitude gain. Examples are tailored in a way to show how a high-pass configuration (solid black example) transforms into a close-to-ideal all-pass configuration (dotted blue example). Vertical lines denote the $10\frac{\left|s_{p,1}\right|}{2\pi }\;$pole frequencies as the low-side limit of the area of minimized phase shifts for the observed case II (|*s*_p,1_| > |*s*_z,1_|, *C*_IN_/*C*_coupling_ < *R*_coupling_/*R*_IN_).

**Influence of equating and decreasing |*s*_z,1_| and |*s*_p,1_|.** The following effect can be observed: as |*s*_z,1_| and |*s*_p,1_| are brought closer to one another, the phase shifts introduced by *s*_z,1_ and *s*_p,1_ (i.e., the dip or the bell) are smaller. In fact, if *s*_z,1_ and *s*_p,1_ canceled out, which would be achieved when *R*_coupling_/*R*_IN_ = *C*_IN_/*C*_coupling_ (46), phase response would flatten out to a value of 0° regardless of the attenuation level in the magnitude response (Figure 29). In other words, bringing *s*_p,1_ closer to *s*_z,1_ flattens not only the magnitude response, but also the phase response, raising it toward 0°. In addition to constant and zero phase response ∡*H*_div_(*jω*), if these two ratios were minimized (*R*_coupling_/*R*_IN_ = *C*_IN_/*C*_coupling_ = 0), a unity gain (0 dB) magnitude response |*H*_div_(*jω*)|_dB_ would be achieved, as described in step 2, and the subsystem *H*_div_(*jω*) would turn into the ideal voltage follower. This speaks in favor of bringing |*s*_p,1_| closer to |*s*_p,1_| and minimizing *R*_coupling_/*R*_IN_ and *C*_IN_/*C*_coupling_ ratios, corroborating once again that the all-pass configuration from step 2 has a greater potential for accurate sensing of sub-Hz frequency components than the high-pass configuration. Namely, in the case of a high-pass configuration, a greater roll-on in magnitude response would cause more devastating phase shifts (Figure 26 and Figure 30). Aside from reducing the difference between |*s*_p,1_| and the fixed |*s*_z,1_| by manipulating the ratios *R*_coupling_/*R*_IN_ and *C*_IN_/*C*_coupling_ for a fixed *R*_coupling_ and *C*_coupling_, both |*s*_z,1_| and |*s*_p,1_| can be further decreased by increasing *R*_coupling_ and *C*_coupling_. This way, the phase shifts in ∡*H*_div_(*jω*) will be moved toward lower frequencies and affect a narrower band of frequencies. Now, if equating and decreasing |*s*_z,1_| and |*s*_p,1_| is observed at the level of individual parameters of the interface, conclusions again show that increase in *R*_coupling_ and *C*_coupling_ from step 1, followed by minimization of *C*_IN_ and maximization of *R*_IN_ from step 2, is beneficial. Accordingly, subcase I.2 (|*s*_p,1_| ≤ |*s*_z,1_|, *C*_IN_/*C*_coupling_ ≥ *R*_coupling_/*R*_IN_) and subcase II.2 (|*s*_p,1_| > |*s*_z,1_|, *C*_IN_/*C*_coupling_ < *R*_coupling_/*R*_IN_) with their prerequisite *R*_IN_ ≥ *R*_coupling_ are desirable, since they allow for the implementation of the least attenuating resistive divider without upper limits on *R*_IN_. Among them, subcase II.2 is preferred, since it allows for relaxed constraints on simultaneous minimization of *C*_IN_ and maximization of *R*_IN_ (step 2).

**Area of minimized phase shifts.** In the last paragraph, we described how to narrow the area of phase shifts of ∡*H*_div_(*jω*) and bring it to lower frequencies. Since these phase shifts are largely concentrated at min{0.1|*s*_z,1_|, 0.1|*s*_p,1_|} < *ω* < max{10|*s*_z,1_|, 10|*s*_p,1_|}, the usable area of minimized phase shifts can be determined accordingly. Interestingly, step 1 has shown that predominantly resistive coupling is achieved below 0.1|*s*_z,1_|, which in fact corresponds to the angular frequency at which the zero *s*_z,1_ introduces its phase shifts in accordance with Bode approximation [273,341,348,349,397]. On the other hand, predominantly capacitive coupling is achieved above 10|*s*_z,1_|, which, in accordance with Bode approximation, corresponds to the angular frequency at which the zero *s*_z,1_ no longer contributes to the phase shifts of the system. In other words, the point of transition into the area of predominantly capacitive coupling, 10|*s*_z,1_| (step 1), corresponds to the frequency of approximate end of phase shifts introduced by *s*_z,1_. Further, in step 2, the area of predominant capacitive divider was presented and distinguished from the area of predominantly capacitive coupling. Therein, it was explained that non-contact and insulated biopotential electrodes, which rely on predominantly capacitive coupling, should be used in both the area of predominantly capacitive coupling and the area of predominant capacitive divider. For this purpose, it was shown that tracking both |*s*_z,1_| and |*s*_p,1_| values is important, since |*s*_p,1_| might be significantly higher than |*s*_z,1_|. Now, in step 3, this can be stressed even further in terms of the phase response: to mitigate the impact of phase shifts present in ∡*H*_div_(*jω*), measurements should be carried out at frequencies at which the phase shifts of both *s*_z,1_ and *s*_p,1_ are minimized. These ranges, *ω* ≤ min{0.1|*s*_z,1_|, 0.1|*s*_p,1_|} and *ω* ≥ max{10|*s*_z,1_|, 10|*s*_p,1_|}, correspond to the desired area within which *gd*_div_ has fallen to values in the vicinity of zero. Given that the designed electrode–body interface is non-contact and/or insulated, only the latter frequency range is of interest. From now on, this area *ω* ≥ max{10|*s*_z,1_|, 10|*s*_p,1_|} will be referred to as the area of minimized phase shifts or area of near-zero group delay. Therein, using the term “minimized” rather than “vanished” and stressing the “near-zero” part is important. Namely, it can be recalled that this entire calculation is based on Bode approximation, which yields a phase error of ±5.7° at 0.1|*s*_z,1_|, 10|*s*_z,1_|, 0.1|*s*_p,1_|, and 10|*s*_p,1_| angular frequencies [273,349]. This means that once the angular frequency max{10|*s*_z,1_|, 10|*s*_p,1_|} is reached and crossed, there is still a small residue of phase shifts, due to which ∡*H*_div_(*jω*) continues to gradually increase (case I) or decline (step II) toward 0° as the frequency is further increased.

**Relation between the three established areas.** Finally, the requirements for measuring in the predominantly capacitive coupling area (step 1) and the predominant capacitive divider area (step 2) can be combined with the requirement for measuring in the area of minimized phase shifts. Accordingly, the target low-side angular frequency limit for measurements should be *ω*_min_ ≥ max{10|*s*_p,1_|, *ω*_C,I_, 10|*s*_z,1_|} = 10|*s*_z,1_| in case I and *ω*_min_ ≥ max{10|*s*_z,1_|, *ω*_C,II_, 10|*s*_p,1_|} = 10|*s*_p,1_| in case II (Table 2). This again shows that aside from tracking and bringing the values |*s*_z,1_| and |*s*_p,1_| closer to one another, decreasing them to lower values is also important to further extend the predominantly capacitive coupling area, predominant capacitive divider area, and area of minimized phase shifts toward lower frequencies. Given the fact that area of minimized phase shifts is only an approximation, ω_min_ should in practice be as high as possible compared to the respective low-side angular frequency.

**STEP 3: SUMMARY.** After considering the magnitude response |*H*_div_(*jω*)|_dB_ and voltage divider attenuation in step 2, the analysis in step 3 is extended to the phase response ∡*H*_div_(*jω*). First, phase delay as the time delay of individual frequency components, Δ*t*_k_, and group delay as the time delay of the signal envelope, *gd*_div_, are derived. Second, the issue behind their non-constant values, arising from non-linearities in the phase response, is observed on clinical-grade ECG signals as a motivational example. Third, based on Bode approximation of phase shifts introduced by the left-half plane zero *s*_z,1_ and the left-half plane pole *s*_p,1_, dips and bell-like shapes are observed in the phase response at min{0.1|*s*_z,1_|, 0.1|*s*_p,1_|} < *ω* < max{10|*s*_z,1_|, 10|*s*_p,1_|}. Suppressing these phase shifts to lower values close to 0° and limiting their influence to a narrower frequency range will draw a smaller deviation between the individual frequency components of the input signal *v*_surface_, hence mitigating signal distortion and morphological changes. For this purpose, three operations prove to be beneficial: first, the values |*s*_z,1_| and |*s*_p,1_| should be brought closer to one another by equating the ratios *R*_coupling_/*R*_IN_ and *C*_IN_/*C*_coupling_ (46). The closer the |*s*_p,1_| value to the |*s*_z,1_| value, the flatter the magnitude response |*H*_div_(*jω*)|_dB_ and the phase response ∡*H*_div_(*jω*), and the closer the phase shifts in ∡*H*_div_(*jω*) to 0°. Second, the smaller the ratios *R*_coupling_/*R*_IN_ and *C*_IN_/*C*_coupling_, the closer the magnitude response |*H*_div_(*jω*)|_dB_ to 0 dB (Figure 29). Third, decreasing |*s*_z,1_| and |*s*_p,1_| will further narrow the area of phase shifts present in ∡*H*_div_(*jω*) and move it toward lower frequencies. Accordingly, an increase in *R*_coupling_ and *C*_coupling_ (step 1), followed by minimization of *C*_IN_ and maximization of *R*_IN_ (step 2), is beneficial. Finally, after introducing the area of predominantly capacitive coupling in step 1 and the area of predominant capacitive divider in step 2, step 3 introduced the area of minimized phase shifts. To ensure that the frequency range of interest is situated in all three areas, the following should be ensured for the lowest angular frequency of interest, ω_min_: *ω*_min_ ≥ 10|*s*_z,1_| in case I (|*s*_p,1_| ≤ |*s*_z,1_|), and *ω*_min_ ≥ 10|*s*_p,1_| in case II (|*s*_p,1_| > |*s*_z,1_|), with a caveat that ω_min_ should be as high as possible compared to these low-side limits to account for the error of Bode approximation. This shows once again that aside from equating|*s*_z,1_| and |*s*_p,1_| values, decreasing them is also important to extend each of the three respective areas toward lower frequencies. All these solutions favor the all-pass configuration from step 2 (Figure 26 and Figure 30). Unlike the high-pass configuration, its focus is not on AC-coupling of the electrode–body interface, but rather on taking the advantage of finite leakage and enhancing the reduction of phase shifts and signal distortion.

**Guidelines for designing non-contact and insulated electrode–body interfaces.** Now, based on the results in Table 2, the laid out analysis can be summarized into four guidelines. To ensure predominantly capacitive coupling in accordance with Test 1, with minimized attenuation and minimized morphological changes at *ω*_min_ ≤ *ω* ≤ *ω*_max_ (refer to Assumption 1), the following steps should be taken:

1. Decrease |*s*_z,1_| = $\frac{1}{R_{coupling}C_{coupling}}\;$ below *ω*_min_/10 by increasing *R*_coupling_ and *C*_coupling_ of the interface.
2. Ensure *C*_coupling_ >> *C*_IN_ to minimize attenuation from the capacitive voltage divider;
3. For a high-pass configuration, ensure *R*_coupling_ >> *R*_IN_ to achieve a roll-on in magnitude response (subcase II.1) and move |*s*_p,1_| ≈ $\frac{1}{R_{IN}C_{coupling}}$ away from |*s*_z,1_| toward higher frequencies. For an all-pass configuration, ensure *R*_IN_ >> *R*_coupling_ and keep |*s*_p,1_| = $\frac{1}{\left(R_{coupling}||R_{IN})(C_{coupling}||C_{IN}\right)}\;$in proximity of |*s*_z,1_| to flatten the frequency response, minimize phase shifts in the phase response, and maximize the magnitude response (subcase I.2 and subcase II.2). These requirements are relaxed in subcase II.2 compared to subcase I.2.
4. Finally, constrain the measurements to *ω* ≥ max{10|*s*_z,1_|, 10|*s*_p,1_|} to mitigate phase shifts and degradation of signal morphology. In addition, bear in mind that *ω*_min_ should be as high as possible compared to this low-side limit to account for the error of Bode approximation.

Guideline number 1 arises from Test 1 applied to the total equivalent *Z*_coupling_. On the other hand, guidelines number 2 and 3 can be understood intuitively as a result of the aim to maximize *Z*_IN_ and thereby minimize the influence of the impedance voltage divider $\frac{Z_{\mathrm{IN}}}{Z_{\mathrm{coupling}}+Z_{\mathrm{IN}}}$, separated into the resistive and the capacitive component. The difference is that for an all-pass configuration, the design strives to maximize *Z*_IN_ and reduce input voltage divider effect across the entire frequency range, whereas the high-pass design focuses on achieving this only in the area of predominantly capacitive coupling and the area of predominantly capacitive divider. Finally, guideline number 4 stresses the importance of satisfying the first three guidelines from the perspective of preserving the morphology of the acquired signal. The positive influence of high *Z*_IN_ on the mitigation of signal distortion has already been highlighted in papers that investigated the input impedance requirements for dry surface-contact ECG recording and its specifications with respect to IEC 60601 performance standard [408]. Overall, in accordance with Assumption 5, the designing principles behind the proposed guidelines are applicable to any given non-invasive active biopotential electrode. They will be further inspected from the perspective of the frequency response of the entire system *H*_el_(*jω*), accounting for the buffer preamplifier, in the next section.

**Practical aspects of achieving a high input impedance of the preamplifier.** Lastly, practical aspects of realizing the expressed guidelines from the perspective of the preamplifier can be discussed. As shown in Figure 10 and in accordance with Assumption 9, *R*_IN_ can be modeled as *R*_bias_||*R*_in_, whereas *C*_IN_ can be modeled as *C*_par_||*C*_in_. This means that achieving as high *Z*_IN_ as possible requires both high *R*_bias_ and *R*_in_, as well as low *C*_par_ and *C*_in_. To accomplish this, various analog techniques can be employed, which will be summarized in the following paragraphs. More details can be found in [86,94,104].

**Practical aspects of DC biasing, feasibility of clinical-grade diagnostics, surface leakage, and dielectric absorption.** As shown in step 2, increasing *R*_IN_ reduces the |*s*_p,1_| value and the attenuation of the resistive voltage divider. From this point of view, *R*_IN_ should be maximized. On the other hand, the paragraph “Bias currents and DC biasing” has shown that high input resistance *R*_in_ makes shunting the bias current *I*_+_ to the circuit common more problematic. Bias currents can be reduced with the use of FET-input stage amplifiers, but this in turn increases *R*_in_ even further (refer to [332,333,383], and subchapters 4x.5 and 4x.10 in [355]). Also, no matter how small they are, they may still limit the long-term performance of the measurement system. Therefore, using active high-input-impedance electrodes necessitates external means of DC biasing, which was most simply modeled as *R*_bias_. To serve its purpose, *R*_bias_ should be at least an order lower than *R*_IN_. Therefore, whenever it is present, it can replace *R*_IN_ in all equations discussed throughout this paper. On the other hand, *R*_bias_ should still be as high as possible in order to avoid degradation of the input impedance. This shows that *R*_bias_ is the limiting factor in the process of increasing *R*_IN_. Furthermore, it leads to the issue of the measured bandwidth and low-side frequency limitations. Capturing the entire bandwidth of ECG signal to allow for clinical diagnostics comparable to hospital-grade systems requires the low-side measurement frequency to be of value 0.05 Hz, or even 0.02 Hz and lower [154] (Assumption 1). According to step 3, this means that |*s*_p,1_| = $\frac{1}{\left(R_{coupling}||R_{IN})(C_{coupling}||C_{IN}\right)}$ ≈ $\frac{1}{R_{IN}(C_{coupling}||C_{IN})}$ (18) should not be higher than 2π·2 mHz. For common *C*_coupling_||*C*_IN_ values of tens of pF, this calls for *R*_bias_ values in the order of 500 GΩ or even higher. Such resistors are expensive, contain a wide tolerance range, and impose a problem of sensitivity to temperature and humidity. This issue of impact on signal morphology at frequencies in the order of 1 Hz and lower is in fact the reason why the majority of predominantly capacitive non-contact and insulated ECG applications have been focused on ambulatory ECG and smart gadget applications such as heart rate measurement, where portability, compactness, and simplicity are of primary concern. Alternatively to discrete resistors with values of 500 GΩ and higher, insulated cable leakage and PCB leakage can be taken advantage of with the use of gimmick resistors from a pair of twisted insulated wires connected to the non-inverting input pin at one end and unconnected on the other end, or with the use of additional PCB pads and a grounded PCB trace placed close to the trace leading to the non-inverting pin. Nevertheless, these are rather prototype solutions, and also, they could increase *C*_IN_ by extra capacitance in the order of 1 pF (see [86] and subchapter 1x.3 in [282]). Even if the design allows for an increase in *R*_bias_, *R*_bias_ cannot be increased indefinitely. Rather, its value is limited by the preamplifier common-mode input voltage range and the output voltage swing with respect to the bias current *I*_+_ [86]. In addition, extremely high values of *R*_bias_ could make the voltage drop introduced by *I*_+_ across *R*_bias_ significant, resulting in an increase in input offset voltage. Also, the higher the *R*_bias_, the more important it becomes to minimize surface leakage between circuit nodes that could occur due to various accumulated contaminants and debris, from flux residue and dust to skin sebum and other products of exocrine glands. For instance, for only 1 V present between two nodes, an additional leakage path between them with resistance as high as 1 TΩ would create a leakage current of 1 V/1 TΩ = 1 pA, which can easily be of the same order as bias currents, or even higher. To avoid such additional spurious bias paths and minimize surface leakage, the guarding technique can be employed, which extends the idea of a grounded PCB trace in close proximity to the non-inverting input. Namely, it surrounds the sensitive, high-input-impedance non-inverting node with a conductor of the same electric potential, thereby effectively minimizing the electric potential difference needed for leakage current flow. Aside from a passive connection of the guard trace to the desired electric potential, this can also be achieved by active driving, similarly to shield drivers [367], to mitigate the influence of additional parasitic capacitance. Aside from externally implemented active guard drivers, active guarding can also be achieved via specialized on-chip guard terminals, as in [249]. Additional leakage might be caused by the dielectric absorption of the dielectric material itself [260,282,305]. Along with intrinsic frequency-dependent variation in dielectric constant, this effect, also called dielectric soakage and dielectric memory, is a second important example of frequency-dependence in the concept of capacitance. It describes a phenomenon of spontaneous recovery of charge and a consequential residual voltage build-up after discharging. This finite error of residual polarization can be accounted for in the model by additional *RC*-series in parallel with the capacitance *C*_coupling_ (i.e., a capacitance in series with the leakage resistance *R*_coupling_). Lastly, finite leakage exists in PCB solder mask as well, hence, solder mask can be removed, and the non-inverting input pin can be bent upwards from the PCB to employ the technique of point-to-point air-wiring or sky-wiring. More on all these techniques can be found in [249,274,336,380,409,410]. Finaly, in any of the aforementioned high-value *R*_bias_ solutions, the final two issues still remain to be solved: first, the issue of long-term stability, and second, the issue of recovery time after a voltage transient. Namely, as seen from the pole angular break frequency |*s*_p,1_| = $\frac{1}{\left(R_{coupling}||R_{IN})(C_{coupling}||C_{IN}\right)}$ (18) and *h*_el_(*t*) (21), the capacitive and resistive elements of the electrode–body interface define the settling time after a transient change, which can reach orders of 10 s when *R*_IN_ in the order of 1 TΩ is achieved [86,99]. This requires additional fast reset circuitries [411] to recover the baseline in reasonable time and prevent high-amplitude artifacts from driving the analog front-end into saturation and obstructing the measurements. For this purpose, voltage-dependent switch-and-transistor-based [411], varistor-based [412], and diode-based circuits [83,104,105,248,411] can be employed. Finally, input impedance can be effectively increased at the frequencies of interest with the use of the bootstrapping method, while at the same time securing a low-impedance bias path for a stable baseline [369,370,410]. Alternative input impedance-boosting methods such as, e.g., conductance neutralization or using a negative impedance converter, can be employed. They are especially suitable for integrated design [140,413].

**Practical aspects of decreasing the input capacitance.** Secondly, reduction in *C*_IN_ can be considered in practice. In the early works on non-contact and insulated electrodes in the 1960s and 1970s [242,243,244,245], *C*_coupling_ in the order of nF was achieved by coating the electrode sensing surface with insulating materials of high dielectric constant. The resulting capacitive voltage divider endured higher input capacitances. With the rapid advancements in electronics that took place in the following decades, *C*_in_ was reduced from the order of 100 pF to the order of 1 pF [414], which, conversely, relaxed the *C*_coupling_ requirement down to the order of 10 pF and 100 pF. This allowed for the sensing surface to be moved away from the immediate vicinity of the body and the utilization of wearable fabrics (which, due to porosity and air cavities, generally achieve lower dielectric constants usually in the range of 1–4 [92,135]). Also, it removed the need for using special coating materials that exhibited unwanted piezoelectric effect under mechanical stress, such as barium-titanate ceramics used in high-*κ* class 2 and class 3 ceramic capacitors (refer to [245], subchapter 1x.3 in [282], and [98]). Nevertheless, as shown in the paragraph “Parasitic input capacitance”, the resulting pin, pad, layout, and cable parasitic capacitance, *C*_par_, is at least in the order of 1 pF, which is still in the order of *C*_in_. Moreover, *C*_in_ could be further increased to the order of 10 pF if large-area input-stage transistors are used [86]. As explained in [97], *C*_IN_ of only 1 pF exhibits impedance of about 16 GΩ at the frequency of 10 Hz (A4), where the maximum power spectral density of ECG is. This way, *Z*_IN_ is degraded even for very low input capacitances. Therefore, unlike *R*_bias_ which is, compared to *R*_in_, a limiting factor in achieving an increase in *R*_IN_, in the case of *C*_IN_, achieving effective decrease in *C*_in_ and *C*_par_ is of equal importance. On the one hand, the aforementioned passive and active guarding schemes [249,274,336,380,409,410] can be employed to effectively reduce spurious conductive paths and minimize surface leakage whilst preserving *R*_bias_. On the other hand, effective reduction of “grounded” capacitances, *C*_par_ and *C*_in_, can be achieved with the use of capacitance neutralization [86,94,104]. In addition, compensating for the internal amplifier capacitance *C*_in_ can also be achieved with power supply bootstrapping [371,372,415].

**Alternative topologies and integrated design.** However, if *C*_coupling_ drops to the order of 1 pF or lower, achieving *C*_IN_ << *C*_coupling_ becomes particularly challenging. In that case, trans-impedance and charge amplifiers can be employed [9,293,294,324,325,326,327,328]. Moreover, various biasing, input impedance boosting, and input capacitance reduction techniques can be employed inside the integrated circuit itself. Such techniques lead to complexified modern designs [104,371,413,416], as well as integrated bioamplifiers [103,109,138,139,140,141,142,176,314,413]. Minimizing the number of external discrete components and using custom integrated solutions instead of off-the-shelf chips allows for greater flexibility in the design: parameters, such as cutoff frequencies, are now designer-defined functions of the designed on-chip elements [417]. This way, the amplifier can be optimized for the specific purpose, and hence, the reduction in both board-level and on-chip parasitic elements can be enhanced. For instance, input capacitances can be further reduced down to the order of 10 fF and 100 fF, as in [109]. Aside from achieving the desired specifications, the choice and design of a biopotential amplifier should also include complying with the requirements of the specific measurement—for instance, performance requirements for ECG measurements can be found in [138,311,408]. Finally, unlike the classic ultra-high-input-impedance active electrodes investigated in this study, two-wired topologies can be used [102]. They can be implemented with the use of a current source bias and either a buffer active electrode, realized with a single transistor or with an operational amplifier [418], or an amplifying active electrode [323]. Also, a combination of both worlds—a high-input-impedance solution and two-wired electrodes—is feasible [415].

### 4.3. System Frequency Response

Appendix D and Section 4.2 brought the analysis of two single-pole subsystems, *H*_preamp_ and *H*_div_, respectively. Now, these two subsystems can be cascaded into the frequency characteristic (frequency response) of the entire system, *H*_el_(*jω*) (25), with magnitude response |*H*_el_(*jω*)|_dB_ and phase response ∡*H*_el_(*jω*) given in (51) [268,269,390,397]. The corresponding transfer function *H*_el_(*s*) was derived in (17), and impulse response *h*_el_(*t*) in (21) in Section 4.1:

$$H_{el}\left(j\omega \right)=H_{div}\left(j\omega \right)\cdot \;H_{preamp}\left(j\omega \right)=\left|H_{\mathrm{el}}\left(j\omega \right)\right|e^{j∡H_{\mathrm{el}}\left(j\omega \right)},$$

$${\left|H_{\mathrm{el}}\left(j\omega \right)\right|}_{\mathrm{dB}}=20\log_{10}\left[\frac{C_{\mathrm{coupling}}}{C_{\mathrm{coupling}}+C_{\mathrm{IN}}}\cdot \frac{\sqrt{\omega^{2}+{\left(\frac{1}{R_{\mathrm{coupling}}C_{\mathrm{coupling}}}\right)}^{2}}}{\sqrt{\omega^{2}+{\left(\frac{1}{{(R_{\mathrm{coupling}}||R}_{\mathrm{IN}})(C_{\mathrm{coupling}}||C_{\mathrm{IN}})}\right)}^{2}}}\cdot \frac{A_{0}\omega_{A}}{\sqrt{\omega^{2}+{\omega_{A}}^{2}}}\right],$$

(51) $$∡H_{\mathrm{el}}\left(j\omega \right)=\mathrm{atan}2\left(\omega R_{\mathrm{coupling}}C_{\mathrm{coupling}}\right)-\mathrm{atan}2\left[\omega (R_{\mathrm{coupling}}||R_{\mathrm{IN}})(C_{\mathrm{coupling}}||C_{\mathrm{IN}})\right]-\mathrm{atan}2\left(\frac{\omega }{\omega_{A}}\right),$$

where atan2 is the four-quadrant arctangent function defined in (30).

**Figure 31:**
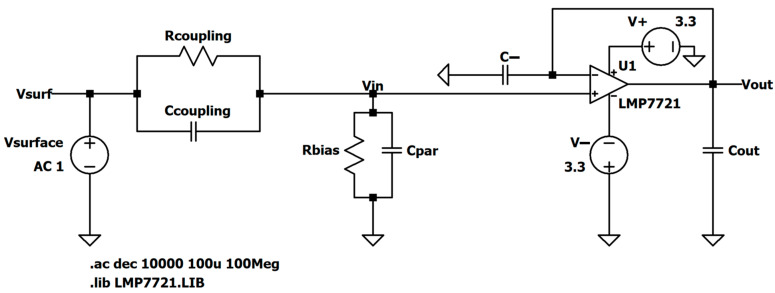
Simulation model of the analyzed system *H*_el_ based on LMP7721 operational amplifier [336,419] and created in LTspice^®^ 24.1.10 simulation software [147] in accordance with Figure 10. Default values of parameters are as follows: *R*_coupling_ = 100 GΩ, *C*_coupling_ = 50 pF, *R*_bias_ = 100 GΩ, *C*_par_ = 10 pF, *C*_−_ = 0 pF, and *C*_out_ = 0 pF.

**Figure 32:**
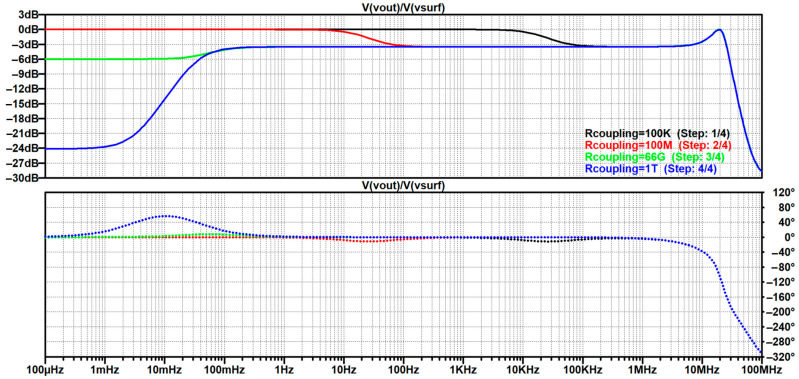
(Solid lines) magnitude response |*H*_el_(*jω*)|_dB_ and (dotted lines) phase response ∡*H*_el_(*jω*) of the entire system based on simulation model in Figure 31 for four values of *R*_coupling_ (100 kΩ, 100 MΩ, 66 GΩ, and 1 TΩ). The rest of the parameters are fixed: *C*_coupling_ = 50 pF, *R*_bias_ = 100 GΩ, *C*_par_ = 10 pF, *C*_−_ = 0 pF, and *C*_out_ = 0 pF.

**Figure 33:**
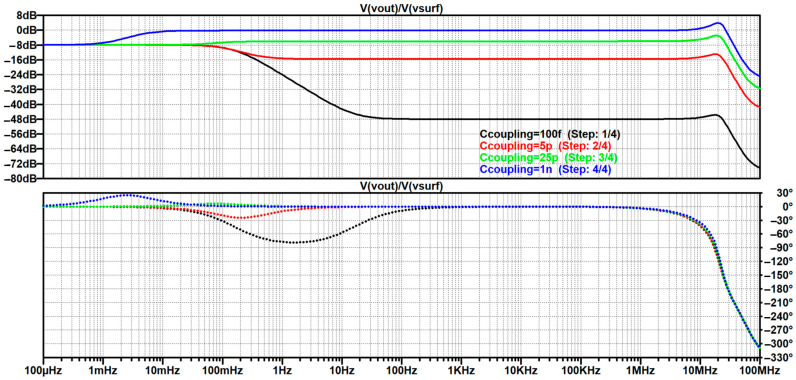
(Solid lines) magnitude response |*H*_el_(*jω*)|_dB_ and (dotted lines) phase response ∡*H*_el_(*jω*) of the entire system based on simulation model in Figure 31 for four values of *C*_coupling_ (100 fF, 5 pF, 25 pF, and 1 nF). The rest of the parameters are fixed: *R*_coupling_ = 100 GΩ, *R*_bias_ = 100 GΩ, *C*_par_ = 10 pF, *C*_−_ = 0 pF, and *C*_out_ = 0 pF.

**Figure 34:**
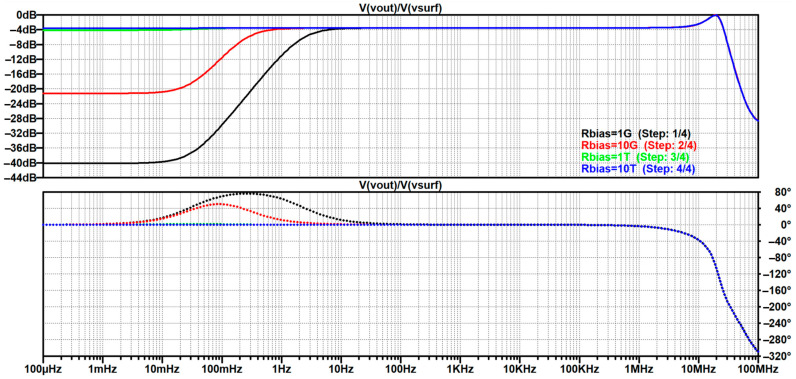
(Solid lines) magnitude response |*H*_el_(*jω*)|_dB_ and (dotted lines) phase response ∡*H*_el_(*jω*) of the entire system based on simulation model in Figure 31 for four values of *R*_bias_ (1 GΩ, 10 GΩ, 1 TΩ, and 10 TΩ). The rest of the parameters are fixed: *R*_coupling_ = 100 GΩ, *C*_coupling_ = 50 pF, *C*_par_ = 10 pF, *C*_−_ = 0 pF, and *C*_out_ = 0 pF.

**Figure 35:**
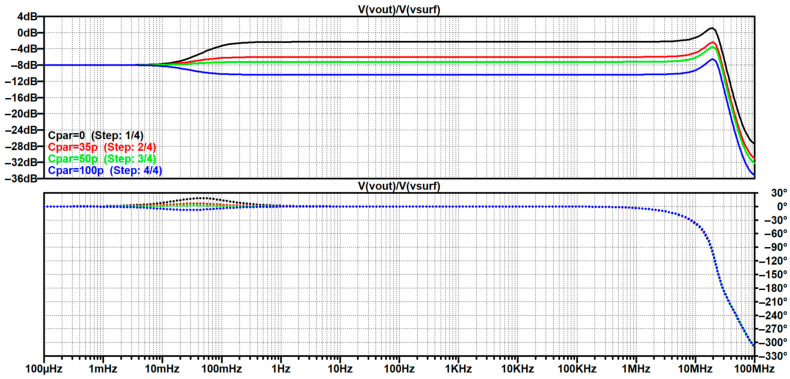
(Solid lines) magnitude response |*H*_el_(*jω*)|_dB_ and (dotted lines) phase response ∡*H*_el_(*jω*) of the entire system based on simulation model in Figure 31 for four values of *C*_par_ (0 pF, 35 pF, 50 pF, and 100 pF). The rest of the parameters are fixed: *R*_coupling_ = 100 GΩ, *C*_coupling_ = 50 pF, *R*_bias_ = 100 GΩ, *C*_−_ = 0 pF, and *C*_out_ = 0 pF.

**Figure 36:**
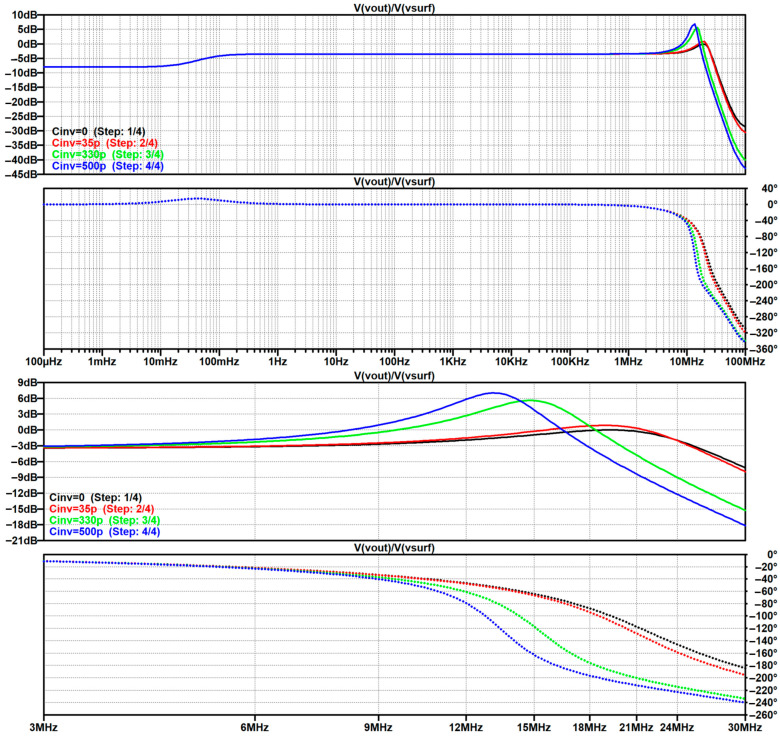
(Solid lines) magnitude response |*H*_el_(*jω*)|_dB_ and (dotted lines) phase response ∡*H*_el_(*jω*) of the entire system based on simulation model in Figure 31 for four values of *C*_−_, denoted by *C*_inv_ (0 pF, 35 pF, 330 pF, and 500 pF). In addition, frequency response is zoomed at frequencies between 3 MHz and 30 MHz. The rest of the parameters are fixed: *R*_coupling_ = 100 GΩ, *C*_coupling_ = 50 pF, *R*_bias_ = 100 GΩ, *C*_par_ = 10 pF, and *C*_out_ = 0 pF.

**Figure 37:**
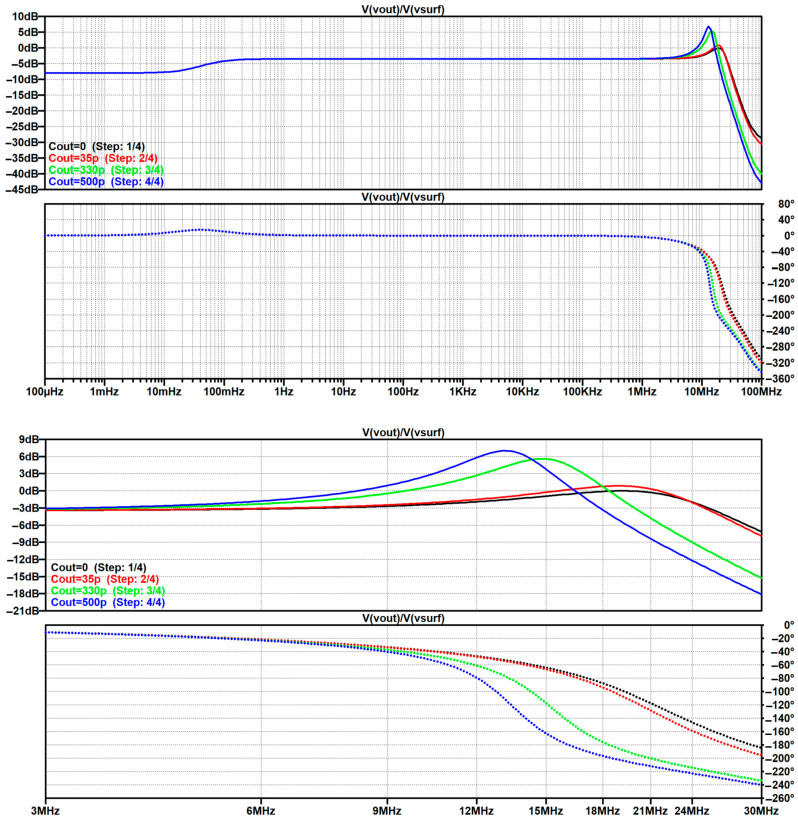
(Solid lines) magnitude response |*H*_el_(*jω*)|_dB_ and (dotted lines) phase response ∡*H*_el_(*jω*) of the entire system based on simulation model in Figure 31 for four values of *C*_out_ (0 pF, 35 pF, 330 pF, and 500 pF). In addition, frequency response is zoomed at frequencies between 3 MHz and 30 MHz. The rest of the parameters are fixed: *R*_coupling_ = 100 GΩ, *C*_coupling_ = 50 pF, *R*_bias_ = 100 GΩ, *C*_par_ = 10 pF, and *C*_−_ = 0 pF.

**Figure 38:**
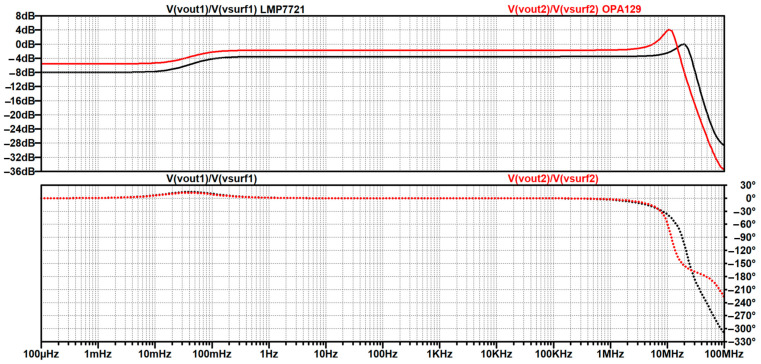
(Solid lines) magnitude response |*H*_el_(*jω*)|_dB_ and (dotted lines) phase response ∡*H*_el_(*jω*) of the entire system based on simulation model in Figure 31 for two operational amplifiers: (black) LMP7721 [336,419] and (red) OPA129 [337,420]. The rest of the parameters are fixed: *R*_coupling_ = 100 GΩ, *C*_coupling_ = 50 pF, *R*_bias_ = 100 GΩ, *C*_par_ = 10 pF, *C*_−_ = 0 pF, and *C*_out_ = 0 pF.

**Figure 39:**
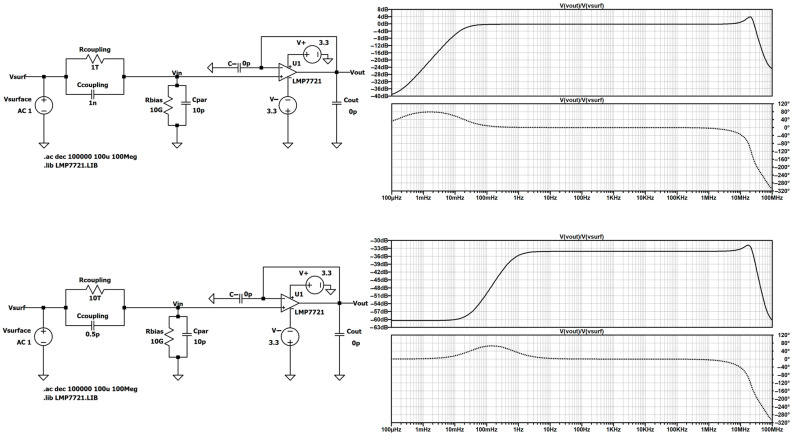

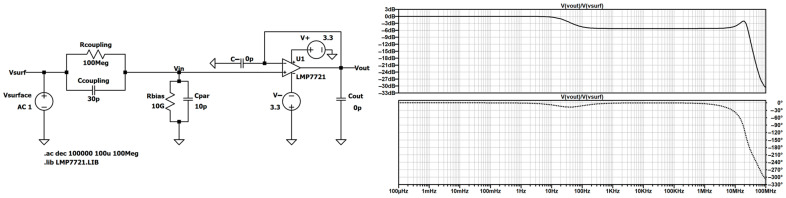
(Solid lines) magnitude response |*H*_el_(*jω*)|_dB_ and (dotted lines) phase response ∡*H*_el_(*jω*) of the entire system based on simulation model in Figure 31 for three different types of electrodes analyzed in Section 3.1: (**upper**) insulated electrode, (**middle**) off-body electrode, and (**lower**) wearable non-contact on-body electrode.

**Summary of upcoming figures.** As inspected in Section 4.1, the entire system *H*_el_ is second-order with two non-trivial, single real poles *s*_p,1_ and *s*_p,2_ (18), and one non-trivial, single real zero *s*_z,1_ (19). To investigate its magnitude response |*H*_el_(*jω*)|_dB_ and phase response ∡*H*_el_(*jω*) (51), simulation is performed in LTspice^®^ 24.1.10 simulation software [147] with the use of SPICE models of two referenced operational amplifiers: LMP7721 [336,419] and OPA129 [337,420]. Firstly, with the use of a model based on LMP7721 (Figure 31), a parametric sweep of four electrode–body interface parameters was performed: *R*_coupling_ (Figure 32) and *C*_coupling_ (Figure 33), which form *Z*_coupling_, and *R*_bias_ (Figure 34) and *C*_par_ (Figure 35) as external parts of *Z*_IN_. These parameters were carefully modeled with values that appropriately reflect the previously investigated effects. Next, the influence of external parasitic capacitance *C*_−_, present at the inverting input terminal and introduced in Appendix D in the discussion titled “Generalized analysis: frequency-dependent feedback network and higher-order models”, is observed in Figure 36. Likewise, the influence of the total effective capacitive load, *C*_out_, representing the parallel combination of *C*_cable_ and *C*_load_ (Figure A10), is investigated in Figure 37. In addition, the influence of the preamplifier and its GBP (A20) is inspected in Figure 38, where frequency response is simulated for both operational amplifiers. Lastly, Figure 39 shows the frequency response for three types of electrodes analyzed in Section 3.1: insulated electrode, off-body electrode, and a wearable non-contact on-body electrode. In this last figure, parameters were modeled with values commonly found in practice to allow for easier comparison with the relevant papers. Results are summarized in the following paragraphs.

**Low-end frequencies and influence of interface parameters.** The cutoff frequencies of the predominant capacitive divider area, *ω*_C,I_ and *ω*_C,II_, derived in step 2 in the previous section (38,43), represent the low-side cutoff frequency of the system *H*_el_. Similarly, high-pass and low-pass configurations, described in step 2 of the previous section, are now observable at the low end of the frequency spectrum. In that area, frequency response *H*_el_(*jω*) is predominantly shaped by the input voltage divider subsystem *H*_div_ and its four interface parameters, explored in the previous section. Resulting curves once again indicate the importance of achieving *C*_coupling_ >> *C*_IN_ (Figure 33 and Figure 35), using on-body electrodes (Figure 39), and taking into account the value of *R*_IN_ with respect to *R*_coupling_ (Figure 32 and Figure 34). In particular, for *R*_coupling_ << *R*_bias_ (black and red curves in Figure 32), resistance *R*_bias_ has no influence on the frequency response, and the area of predominantly resistive coupling stretches up to the frequencies in the order of 1 kHz. This was explained earlier in paragraph “Bias currents and DC biasing” considering the bias current path provided by low *R*_coupling_, and now, it can also be understood in terms of the resistive input voltage divider, as explained in step 2 in the previous section. Furthermore, an increase in *R*_coupling_ from very low values (*R*_coupling_ << *R*_bias_) to very high values (*R*_coupling_ >> *R*_bias_) turns the configuration of the interface at low-end frequencies from all-pass to high-pass, shifting |*s*_z,1_| to lower frequencies. An increase in *R*_coupling_ without a corresponding increase in *R*_bias_ increases the difference between the magnitude levels of the resistive and capacitive voltage dividers, 20log_10_$\frac{R_{IN}}{R_{coupling}+R_{IN}}$ and 20log_10_$\frac{C_{coupling}}{C_{coupling}+C_{IN}}$, but it also increases the phase shifts present in the phase response ∡*H*_el_(*jω*) between angular frequencies 0.1|*s*_z,1_| and 10|*s*_p,1_|, as explained in step 3 in the previous section. Moreover, *R*_bias_ is only an external part of *R*_IN_, which is formed by the parallel combination of *R*_bias_ and *R*_in_ introduced by the preamplifier. Therefore, for *R*_bias_ << *R*_in_, *R*_IN_ comes down to *R*_bias_ (*R*_IN_ ≈ *R*_bias_), whereas for *R*_bias_ >> *R*_in_, *R*_IN_ comes down to *R*_in_ (*R*_IN_ ≈ *R*_in_). In other words, for a specified *R*_bias_, the value of *R*_in_ can be estimated by observing the magnitude of the resistive voltage divider in the magnitude response |*H*_el_(*jω*)|_dB_ (Figure 32 and Figure 34). The resistance *R*_in_ turns out to be about 195 GΩ. This explains why 66 GΩ of *R*_coupling_ gives 6 dB (50%) attenuation of the resistive input voltage divider in Figure 32. Similarly, *C*_par_ is only an external part of *C*_IN_, which is formed by the parallel combination of *C*_par_ and *C*_in_. In other words, for a chosen *C*_par_, the value of *C*_in_ can be estimated by observing the magnitude of the capacitive voltage divider in |*H*_el_(*jω*)|_dB_ (Figure 33 and Figure 35). The capacitance *C*_in_ turns out to be fairly high—15 pF, as estimated in the LMP7721 datasheet [336]. This explains why 35 pF of *C*_par_ for 50 pF of *C*_coupling_ is sufficient to yield 6 dB (50%) attenuation of the capacitive input voltage divider 20log_10_$\frac{C_{coupling}}{C_{coupling}+C_{IN}}$. On the other hand, Figure 38 reveals that the estimated value of *C*_in_ in the case of OPA129 is about 2–3 pF, as stated in the OPA129 datasheet [337].

**Mid-band and high-end frequencies.** At the high-end frequencies, the high-side cutoff frequency of the system *H*_el_ is defined by the closed-loop bandwidth *ω*_A_. For the observed single-pole model of a buffer preamplifier *A*_diff_(*f*) (A17)–(A19) and the ideal unity closed-loop gain *A*_0_ = 1, *ω*_A_ is equal to the open-loop unity-gain crossover angular frequency *ω*_1_, and hence the high-side cutoff frequency is determined by the GBP of the preamplifier (A20) (Figure 38). The area between the low-side and the high-side cutoff frequency corresponds to the mid-band gain, where the magnitude response |*H*_el_(*jω*)|_dB_ is dominated by the magnitude level of the capacitive voltage divider. In accordance with the guidelines given in Section 4.2, the mid-band should be used between angular frequencies max{10|*s*_z,1_|, 10|*s*_p,1_|} and *ω*_1_/10.

**Higher-order models and stability issues revisited.** Finally, effects at high-end frequencies can be observed more carefully. For this purpose, the influence of additional poles and parasitic capacitances *C*_−_ and *C*_out_ that were described in the discussion titled “Generalized analysis: frequency-dependent feedback network and higher-order models” in Appendix D can be recalled. Treating these capacitances as being parallel to one another is now corroborated by their almost identical separate influence depicted in Figure 36 and Figure 37. With respect to the adopted conservative estimation of their influence (A21), the typical GBP of LMP7721 is about ten times higher—15 MHz [336], and its closed-loop output impedance is reduced to very low values in the order of 1 Ω. Additionally, gain peaking is observed at a frequency of about the GBP even when *C*_−_ and *C*_out_ are 0 pF. Adding 35 pF of external *C*_−_||*C*_out_ capacitance reduces the phase margin to 45°, whereas 330 pF reduces it to 0°. Similar simulations for various operational amplifiers and their configurations are available in [355]. As discussed in Appendix D, this shows why modeling at higher frequencies demands higher-order models of operational amplifiers, as well as performing thorough stability analysis with respect to the influence of reactive components in the system, especially the ones in the amplifier feedback, at its inverting input, and at its output. To quantify gain peaking and damping of the response, damping ratio and quality factor [421] can be used as indicators of system behavior under disturbance. More on peaking and phase margin can be found in [273,335,353]. More on damping ratio and quality factor in the context of second-order systems can be found in [341,345,354,390], with details behind various practical engineering examples in [395,422,423]. Examples of second-order and third-order modeling of operational amplifiers [341,346,356] can be found in [367] for the case of shield-driving circuits, in [369,370] for the case of bootstrapping networks, in [371] for a combination of a shield-driving circuit, bootstrapping network, and power supply bootstrapping network, and in [313] for fully differential biopotential amplifiers. Considerations on dry electrode transfer function and amplifier specifications with respect to standards for ECG recording can be found in [311,408]. Additional useful references can be found in discussions titled “Generalized analysis: frequency-dependent feedback network and higher-order models” and “Stability in practical biopotential measurements” in Appendix D.

**Examples of physical measurements and concluding remarks.** To carry out the corresponding physical measurements of electrode–body coupling impedance *Z*_coupling_ and system frequency response *H*_el_(*jω*), it is necessary to come up with solutions that will allow for a reliable operation of non-contact and insulated electrodes in spite of all the adverse phenomena present in real-life experiments. These phenomena are usually neglected in the system analysis due to their high and difficult-to-control dependence on measurement setup, ambient conditions, and environmental noise. Firstly, aside from a complexified design of electrodes and bioamplifiers based on methods described at the end of Section 4.2 [104,371,413,416], such measurement systems also require designing a dedicated DAQ unit, along with a firmware solution for communication protocol, data transmission, and storage, as well as for rejection of power line interference and mitigation of motion artifacts [94,95], which must be implemented in such a way that it does not affect the real-time performance or signal quality. Secondly, such systems call for carefully planned mechanical layouts that will enable stable contact conditions. Lastly, they demand sophisticated methods of monitoring *Z*_coupling_, which would ensure the predominantly contactless coupling principle. This is achieved by implementing strict galvanic isolation or (preferably) battery-powered instrumentation, as well as by managing the transient behavior and the so-called hidden parameters, such as perspiration, temperature, and pressure, which endanger the long-term temporal stability [424]. For these reasons, a considerable number of papers in the field of non-contact and insulated biopotential monitoring have been dedicated solely to a certain aspect of these demanding practical challenges [102,176,177,226,371,380,424,425,426,427,428,429]. However, each of these ingeniously devised measurement protocols and systems is commonly based on a use-case-specific analog front-end and DAQ solution. Also, they are evaluated under significantly different ambient conditions, which are accompanied by sources of electromagnetic interference that are uniquely tied to the measurement environment. When the issue of inherently problematic repeatability of non-contact measurements is added, along with the fact that the dielectric behavior and electrical performance of real-world dielectrics depend on their manufacturing process, structural properties, and ambient conditions, it becomes clear why the obtained measurements are in most cases only qualitatively comparable. Nevertheless, this does not lessen their use in further validation of the model presented in this paper. Namely, in contrast to the aforementioned papers dedicated to solutions for experimental validation, the goal of this paper was to consolidate theoretical foundations with existing practical design implications, and address long-standing terminological inconsistencies in the field of non-contact and insulated biopotential monitoring. To achieve this, an electrical model of the non-contact and insulated electrode–body interface, based on physical data and developed over the last several decades, is generalized and abstracted into a simplified, yet handy engineering tool that allows for the qualitative assessment of systems for non-contact and insulated biopotential measurements. In addition, it also provides a theoretical framework for understanding the phenomena behind real-life experiments. Ultimately, analytical discussions in Section 4.1 and Section 4.2 helped to identify the four interface parameters. Understanding their physical origin is the first step toward embracing the practical limitations of capacitive coupling and using that knowledge to qualitatively anticipate the behavior of real-world non-contact and insulated biopotential monitoring systems. In that sense, the developed model can indeed support examples of physical measurements available in the literature. For instance, in [380], simulation and physical measurements of magnitude response are provided. Simulation is based on OPA124 (Texas Instruments, Inc., Dallas, TX, USA), with parameters *R*_coupling_ = 1 TΩ, *C*_coupling_ = 150 pF, *R*_bias_ in the order of 1 GΩ, *R*_in_ = 100 TΩ, and *C*_IN_ = 3 pF. Since *R*_bias_ << *R*_in_, *R*_IN_ comes down to *R*_bias_. Therefore, *C*_IN_/*C*_coupling_ < *R*_coupling_/*R*_IN_, which belongs to case II (|*s*_p,1_| > |*s*_z,1_|). As a result, low-side frequency of the predominant capacitive divider area, *ω*_C,II_, is defined as in (43). When the effect of an increase in *R*_bias_ is observed for all other parameters fixed (Figure 4 in [380]), since *R*_IN_ ≈ *R*_bias_ for all considered values of *R*_bias_, the corresponding *f*_C,II_ is shifted toward lower frequencies. On the other hand, when the effect of increase in *C*_IN_ is observed for all other parameters fixed and *R*_bias_ = 3 GΩ (Figure 5 in [380]), magnitude level 20log_10_$\frac{C_{coupling}}{C_{coupling}+C_{IN}}$ is decreased due to increased attenuation of the capacitive voltage divider. Furthermore, physical measurements of magnitude response are provided for a system based on AD8642 [338] with active guarding circuitry. |*H*_preamp_(*jω*)|_dB_ is simulated and measured separately in Figure 15 in [380], yielding the high-side cutoff frequency *f*_A_ in accordance with the typical GBP of about 3 MHz, chosen closed-loop gain *A*_0_ = 40 dB = 10^40/20^ = 100 V/V, and the fact that two operational amplifiers were used. Lastly, magnitude response is plotted for three different wearable fabrics: 100% cotton of thickness 0.45 mm, 100% polyester of thickness 0.26 mm, and 100% wool of thickness 0.91 mm. For the predefined coupling (sensing) area *A* = 16 cm^2^, parallel plate capacitor formula (3) could be used to calculate *C*_coupling_ in each of the three cases. However, no data is available for the values of corresponding dielectric constants *ε*_r_ and bulk resistivities *ρ*. As noted earlier in this paragraph, these values depend on the moisture content and structural properties, such as volume density (packing factor) and, consequently, applied pressure [430]. Based on the expected values for *ε*_r_ (1–3 [92,135]), expected *C*_coupling_ is in the range of 30–100 pF (3). Thus, for the simulated *C*_in_ of 150 pF, *C*_coupling_ will be of the same order as *C*_in_, which yields the magnitude level 20log_10_$\frac{C_{coupling}}{C_{coupling}+C_{IN}}\approx$ −8 dB or lower. Similar qualitative comparison is available with other papers: in [384], magnitude response is obtained for three values of sensing (coupling) area *A*; in [296], surface-contact and over-cloth magnitude response are compared; and finally, in [49], magnitude response is obtained for three values of dielectric thickness, *d*. In all three cases, parallel plate capacitor approximation (3) can be used to assess the influence of the varying parameters: larger coupling area *A* increases *C*_coupling_, thereby increasing the corresponding magnitude level 20log_10_$\frac{C_{coupling}}{C_{coupling}+C_{IN}}$. On the contrary, greater thickness *d* decreases *C*_coupling_, thereby decreasing the corresponding magnitude level 20log_10_$\frac{C_{coupling}}{C_{coupling}+C_{IN}}$.

## 5. Conclusions

In Section 1.1, various examples of biomedical and non-biomedical measurements demonstrated versatility of the capacitive sensing strategy, as well as its promising role in electrophysiological and biopotential sensing. Relying on capacitive coupling over insulating layers, the non-contact biopotential measurement principle offers zero preparation and seamless integration of electrodes into everyday garments and wearable body area networks, paving the way for unobtrusive, long-term, remote health monitoring to become standard practice. However, there are challenges left to tackle before unlocking their full potential and widespread use in applications for reliable early diagnostics.

In Section 3.1 and Appendix B, after establishing the frequency range of interest in Assumption 1, motivation for further research on non-contact and capacitive biopotential monitoring was found through a comparison of various types of biopotential electrodes according to their working principle and characteristics: wet and non-insulated dry surface-contact electrodes on the one side (Appendix B) and dry non-contact and capacitive electrodes on the other side (Section 3.1). The analysis of their equivalent electrical models, as well as electrochemical processes at the interface (Figure A2 and Figure A3) and finite leakage resistances of insulating layers (Figure 3), showed that coupling with biopotential electrodes comes down to a combination of resistive and capacitive elements. Discussion in Section 3.1, starting from the coupling over a single insulating layer, resulted in the creation of a generalized model of the interface between the body and a non-contact biopotential electrode (Figure 5, Contribution 2.1). The model is based on parallel plate approximations described with Equations (3), (7), and (9)–(13). These equations revealed that the equivalent electrode–body coupling impedance *Z*_coupling_ can be manipulated with a careful choice of electrode sensing (coupling) area, as well as the coupling material and its thickness. By accounting for a finite leakage resistance of insulating layers, the distinction was emphasized between idealized capacitive electrodes on the one side, and non-contact and insulated electrodes with predominantly capacitive coupling on the other side. In brief, contactless biomonitoring does not guarantee predominantly capacitive coupling, nor does surface-contact biomonitoring guarantee predominantly resistive coupling. At DC (*f* = 0 Hz), equivalent leakage resistance of the electrode–body interface, *R*_coupling_, defines the bulk DC conductivity of the interface, as well as its electrical insulation performance. On the other hand, at a certain frequency *f* > 0 Hz, the value of frequency-dependent *R*_coupling_(*ω*) (9) relative to the capacitive reactance *X*_Ccoupling_(*ω*) (A4) determines the predominant coupling mechanism (resistive or capacitive). Therefore, Test 1 was proposed as a tool for assessing the predominant coupling mechanism achieved over an insulating layer at a certain frequency (Contribution 1). Results are given in Table 1, visualized in Figure 4, and applied to three specific cases of the generalized electrode–body interface model in Figure 5: insulated electrodes, off-body electrodes, and non-contact on-body electrodes. Due to the devastating effect of an air gap (off-body case), on-body electrodes were highlighted as preferred for biopotential measurement. Important findings and examples, summarized in the paragraph titled “Important conclusions and misterming issues as a motivation for the classification of biopotential electrodes”, led to Assumption 4 and replacement of the initially used term “insulating layer” with a more general term “coupling layer”. Likewise, the initially used group term “non-contact and capacitive biopotential electrodes” was replaced with a more appropriate group term “non-contact and insulated biopotential electrodes”. Finally, refined classification of biopotential electrodes was proposed to clearly emphasize that the terms “non-invasive”, “on-body”, “off-body”, “surface-contact”, “non-contact”, “wet”, “dry”, “insulated”, and “capacitive” are not synonyms (Figure 6, Contribution 3).

After explaining the benefits of active electrode design over passive design in Section 3.2, the generalized model of non-contact and insulated electrode–body interfaces was expanded into a model of a single buffer active non-contact and/or insulated biopotential electrode and its interface with the body (Contribution 2.2). The model is based on Assumption 5, introduced in paragraph “Introduction to Section 4 and the first subsystem: operational amplifier”, and depicted in Figure 10. Therein, two equivalent impedance blocks were highlighted for further analysis: the total equivalent electrode–body coupling impedance *Z*_coupling_, defined in Assumption 4, and the total equivalent input impedance *Z*_IN_, defined in Assumption 9. These two equivalent impedances comprise a resistive and a capacitive component, described by four interface parameters: *R*_coupling_, *C*_coupling_, *R*_IN_ = *R*_bias_||*R*_in_, and *C*_IN_ = *C*_par_||*C*_in_. The parameters *R*_in_ and *C*_in_ of the preamplifier common-mode input impedance were introduced in paragraph “Model of a buffer active non-contact and/or insulated electrode and its interface with the body with three equivalent impedances.” On the other hand, the parameter *C*_par_, as the pin, pad, layout, and cable capacitance between the non-inverting input pin (sensing surface) and the preamplifier circuit common, was introduced in paragraph “Parasitic input capacitance”, whereas the parameter *R*_bias_, as the equivalent resistance of the external DC biasing network, was introduced in paragraph “Bias currents and DC biasing.” Influence of the rest of the parameters—additional capacitances *C*_−_*, C*_com−_*, C*_diff_, and *C*_out_ = *C*_cable_||*C*_load_ seen at the preamplifier inverting input and output—was analyzed and neglected in Appendix D in the discussion titled “Generalized analysis: frequency-dependent feedback network and higher-order models”, and revisited in Section 4.3.

The model presented in Figure 10 was further employed throughout Section 4 to carry out the bottom-up analysis of the entire system interface–electrode–preamplifier, which was defined as a cascade of two first-order (single-pole) subsystems: the input voltage divider and the buffer preamplifier. In accordance with Assumption 5, the provided analysis is also applicable to other non-invasive active biopotential electrodes.

The first subsystem—buffer preamplifier—was built in Appendix D. Therein, the analysis started from the working principle of a voltage-feedback operational amplifier, as well as considerations on its stability and voltage follower (buffer) configuration. As a result, the single-pole model of a buffer preamplifier, *A*_diff_(*f*) (A17)–(A19), was established and adopted for the rest of the paper as the subsystem *H*_preamp_. Assumptions on the operational amplifier and the preamplifier analog front-end, as well as on the PCB design considerations, are listed in Assumptions 7 and 8.

In Section 4.1, the transfer function of the entire system interface–electrode–preamplifier, *H*_el_(*s*) (17), and its impulse response, *h*_el_(*t*) (21), were derived. The pole–zero plot was observed (Figure 12), and an example of impulse response and the corresponding transfer function were visualized in Figure 13 and Figure 15, respectively. Specifically, Figure 14 revealed how the location of poles in the complex *s*-plane shapes the impulse response and the time-domain transient response. The transfer function *H*_el_(*s*) confirmed the key influence of the input voltage divider effect: voltage sensing depends on the frequency-dependent voltage divider $\frac{Z_{\mathrm{IN}}}{Z_{\mathrm{coupling}}+Z_{\mathrm{IN}}}$, created by the equivalent electrode–body coupling impedance *Z*_coupling_ and the finite equivalent input impedance *Z*_IN_. Lastly, for the steady-state analysis, Laplace transform and transfer function were reduced to Fourier transform and frequency characteristic (frequency response) *H*_el_(*jω*) (25) with its magnitude and phase response (51). In accordance with Bode approximations presented in Figure A9, an example in Figure 15 showed how poles and zeros of the transfer function were translated into break frequencies of the corresponding magnitude response.

The second subsystem—input voltage divider and its frequency response *H*_div_(*jω*) (26)—were further inspected in Section 4.2. Therein, it has been shown how voltage attenuation and additional phase shifts, introduced by the zero *s*_z,1_ (19) and the pole *s*_p,1_ (18), degrade the frequency response and adversely affect ECG signal morphology. This was demonstrated in Figure 26. Under the assumption of a negligible contribution of the AC conductivity at the frequencies of interest (subcase A.1 in Table 1), two main cases (case I and case II) were identified and illustrated in Figure 16 based on the position of *s*_p,1_ with respect to *s*_z,1_. Additionally, subcases 1 and 2 were observed based on the value of *R*_IN_ with respect to *R*_coupling_. The results are listed in Table 2 for each of the four subcases. Applying Test 1 and Bode approximations to *Z*_coupling_ and subsystem *H*_div_(*jω*) in three steps revealed how the position of the zero *s*_z,1_ and the pole *s*_p,1_ determine each of the three specific areas: area of predominantly capacitive coupling (step 1), area of predominant capacitive divider (step 2), and area of minimized phase shifts and near-zero group delay (step 3). Accordingly, step 2 analyzed the magnitude response |*H*_div_(*jω*)|_dB_ and magnitude levels of the resistive and capacitive voltage dividers, 20log_10_$\frac{R_{IN}}{R_{coupling}+R_{IN}}$ and 20log_10_$\frac{C_{coupling}}{C_{coupling}+C_{IN}}$, respectively. On the other hand, step 3 analyzed the phase response ∡*H*_div_(*jω*) and area of minimized phase shifts. Discussion in each step was corroborated by graphical examples and concluded with a summarizing paragraph.

The derived relation between zero and pole angular break frequencies, |*s*_z,1_| and |*s*_p,1_| (31,46), showed that the existence of the pole is a direct consequence of the input voltage divider effect. Further analysis in step 2 revealed an important interdependence: greater difference between |*s*_z,1_| and |*s*_p,1_| draws a greater difference between the magnitude levels of the resistive and capacitive voltage dividers, and vice versa. In that sense, the shape of the frequency response *H*_div_(*jω*) can be modeled by relocating *s*_p,1_ with respect to *s*_z,1_. This comes down to manipulating the four interface parameters and their ratios *R*_coupling_/*R*_IN_ and *C*_IN_/*C*_coupling_, depending on the target configuration of the interface. On the one hand, high-pass configuration achieves a roll-on in the magnitude response. Hence, moving *s*_p,1_ further away from *s*_z,1_, increasing *R*_coupling_/*R*_IN_, and decreasing *C*_IN_/*C*_coupling_ is beneficial. On the other hand, all-pass configuration achieves a flattened magnitude response. Hence, keeping *s*_p,1_ in the vicinity of *s*_z,1_, as well as equating and decreasing the ratios *R*_coupling_/*R*_IN_ and *C*_IN_/*C*_coupling_, is beneficial. For both configurations, the goal is to minimize the attenuation of the capacitive divider and achieve *C*_coupling_ *>> C*_IN_, i.e., *C*_coupling_ *≥* 10*C*_IN_. However, high-pass configuration strives for the concept of a pure capacitive electrode with a focus on AC-coupling and electrical insulation of the electrode–body interface; thus, it favors increasing *R*_coupling_. On the other hand, all-pass configuration takes advantage of the finite *R*_coupling_ (i.e., finite interface leakage) to focus on extending the measurement range further toward lower frequencies, offering the additional benefit of flattened phase response. As a result, all-pass configuration is less prone to distortion of the signal.

Three-step analysis in Section 4.2 resulted in guidelines for designing non-contact and insulated electrode–body interfaces, proposed in the paragraph “Guidelines for designing non-contact and insulated electrode–body interfaces” (Contribution 4). The aim of these guidelines is to extend the predominantly capacitive coupling area, predominant capacitive divider area, and area of minimized phase shifts toward lower frequencies. Ultimately, flattening the frequency response of the input voltage divider *H*_div_(*jω*) whilst reducing attenuation of its magnitude response and minimizing phase shifts in its phase response is beneficial. This comes down to an increase in *R*_coupling_ and *C*_coupling_, followed by maximization of *R*_IN_ and minimization of *C*_IN_.

These results were further confirmed in Section 4.3 with the use of SPICE simulation of the entire system. Therein, both subsystems, *H*_div_ analyzed in Section 4.2 and *H*_preamp_ analyzed in Appendix D, were combined to observe the total frequency response *H*_el_(*jω*). The analysis has shown that the input voltage divider and electrode–body interface shape the frequency response at low-end frequencies, whereas the preamplifier with its closed-loop bandwidth shapes the frequency response at high-end frequencies.

In practice, maximization of *R*_IN_ is limited by the DC biasing network and its equivalent resistance *R*_bias_. On the other hand, minimization of *C*_IN_ is limited by both the preamplifier common-mode input capacitance *C*_in_ and the pin, pad, layout, and cable parasitic capacitance *C*_par_. The presence of these elements adversely affects the low-side frequency limit of the system mid-band gain, and ignoring them could impede the use of active non-contact and insulated biopotential electrodes in clinical-grade applications. Therefore, their design calls for reconciling the goal of achieving a high input impedance on the one side, which comes with the issue of vulnerability to electric field interference and sensitivity to surface leakage, with the goal of achieving proper DC biasing and fast recovery from large input transients on the other side. For this purpose, various advanced techniques for biasing, input impedance boosting, and input capacitance reduction can be employed, taking into account the balance between cost, size, consumption, and performance. Moreover, specialized integrated bioamplifiers can be employed, as well as solutions other than the analyzed classic high-input-impedance models, such as two-wired topologies, transimpedance amplifiers, and charge amplifiers. These practical aspects of the proposed guidelines were surveyed at the end of Section 4.2.

As discussed in Section 4.3, the goal of this paper was to bring an analytical interpretation of commonly obtained measurement results, as well as explain the caveats behind modeling of biopotential electrodes and their interface with the body. Since the purpose of the laid out system analysis was to provide a deeper insight into the influence of individual parameters that build the equivalent electrical model of non-contact and insulated electrode–body interfaces, simulations were the preferred way of communicating the results of analytical calculation. Accordingly, the intent of this paper was not to introduce novel experiments, but rather to synthesize, reconcile, and systematize the extensive and sometimes inconsistent body of literature in accordance with three objectives and four contributions that were listed and visualized in Section 1.2. Based on other review papers in this research area, categorized in Section 2, this review serves the purpose of enhancing the transparency and unification of earlier findings, translating them from between-the-lines outcomes of a specific research approach into an elaborate list of abstracted conclusions that streamline the access to existing work and allow for easier dissemination of knowledge among research groups, with the hope of preventing the pinpointed conceptual errors and terminological inconsistencies in the future. Accordingly, instead of skipping elementary derivations and common engineering knowledge, fundamentals of the following were thoroughly revisited: biopotential signals and their acquisition (Appendix A), electrochemistry (Appendix B), dielectrics, dielectric loss, capacitors, phasors and impedance (Appendix C and Section 3.1), operational amplifiers, negative feedback, Bode plot and amplifier stability (Appendix D and Section 3.2), transfer function, impulse response, frequency characteristic and system stability (Section 4.1), and phase delay and group delay (Section 4.2). The analysis was built upon each of the nine established assumptions, which offer an abridged version of the content for quick access. The results were additionally corroborated by examples of real-life parameter values, practical aspects of real-world electronic components, and several examples of commonly used operational amplifiers.

Finally, to further place the analysis in the context of real-life measurements available in the existing literature, the last paragraph in Section 4.3 titled “Examples of physical measurements and concluding remarks” expounds on how the existing experimental data relate to the presented theoretical framework. While the developed and generalized model is a cornerstone for modern non-contact and insulated biopotential electrodes, papers dedicated to the challenges of real-world measurements reveal the presence of additional phenomena, which arise from electromagnetic interference with the measurement environment and from variations in interface parameters as a result of motion and non-constant ambient conditions. Nevertheless, the provided model can be used to qualitatively assess non-contact and insulated electrode–body interfaces with respect to the clarified terminology and resolved misconceptions. Further accounting for the adverse effects of environment, ambient conditions, and real-world electronic components would require the addition of interference and noise analysis to the presented system analysis.

## Figures and Tables

**Table 1:** Two cases (A and B) based on the position of *ω*_δ_ = $\frac{F}{R_{coupling,DC}C_{coupling}}$ with respect to angular frequency $\frac{T}{R_{coupling,DC}C_{coupling}}$. Factors *F* and *T* are positive real numbers. For each of the four subcases, the resulting bandwidths, frequency bands, frequency ranges, or areas of predominantly capacitive coupling are given based on the criterion $R_{coupling}(\omega )\geq {T\cdot X}_{Ccoupling}$ for *T* ≥ 10. For *R*_coupling_(*ω*), a first-order model that is monotonically decreasing with frequency as in (9) is assumed.

**Case A**	Condition: $\omega_{\delta }\geq \frac{T}{R_{coupling,DC}C_{coupling}}$ , T = 10 in this paper	
	$\omega_{\delta }=\frac{F}{R_{coupling,DC}C_{coupling}},\;\;\;\;\;\;\;F\geq T\geq 10,\;\;\;\;\;\;\;\frac{1}{10}\geq \frac{1}{T}\geq \frac{1}{F}$	
	Area of predominantly capacitive coupling	**Subcase A.1** $\omega \geq \frac{T}{R_{coupling,DC}C_{coupling}}\;\;\;\;for\;tan\delta \leq \frac{1}{T}$ T = 10 in this paper
		**Subcase A.2** $\frac{T}{R_{coupling,DC}C_{coupling}}\leq \omega \leq \omega_{\delta }\frac{tan\delta -\frac{1}{F}}{tan\delta -\frac{1}{T}}\;\;\;\;for\;tan\delta >\frac{1}{T}$ T = 10 in this paper
**Case B**	Condition: $\omega_{\delta }<\frac{T}{R_{coupling,DC}C_{coupling}}\;$, T = 10 in this paper	
	$\omega_{\delta }=\frac{F}{R_{coupling,DC}C_{coupling}},\;\;\;\;\;\;\;F<T,\;\;\;\;\;\;\;T\geq 10$	
	Area of predominantly capacitive coupling	**Subcase B.1** $\omega \geq \omega_{\delta }\frac{\frac{1}{F}-tan\delta }{\frac{1}{T}-tan\delta }\;\;\;\;for\;tan\delta <\frac{1}{T}$ T = 10 in this paper
		**Subcase B.2** $\omega \in \emptyset \;for\;tan\delta \geq \frac{1}{T}$ T = 10 in this paper

**Table 2:** Four subcases (I.1, I.2, II.1, and II.2) based on the position of the pole *s*_p,1_ (18) with respect to the zero *s*_z,1_ (19) (case I and case II) and based on the *R*_coupling_/*R*_IN_ ratio (subcases 1 and subcases 2). First, bandwidth, frequency band, frequency range, or area of predominantly capacitive coupling is given in accordance with Test 1; it will be further explored in step 1. Second, for cases I and II, cutoff frequencies are given, along with areas of predominant capacitive divider, which will be derived in step 2. Third, area is given in which the influence of phase shifts from the pole and the zero is minimized. This topic will be covered in step 3. Lastly, for all four subcases, the feasibility of achieving minimized *C*_IN_ (*C*_coupling_ >> *C*_IN_, i.e., *C*_coupling_ ≥ 10*C*_IN_) is investigated. To generalize the results, a positive real factor *T* ≥ 10 can be used instead of number 10. This will be further explored in step 2.

**zero:** $s_{z,1}=-\frac{1}{R_{coupling}C_{coupling}}$				$\left|s_{p,1}\right|=\frac{1+\frac{R_{coupling}}{R_{IN}}}{1+\frac{C_{IN}}{C_{coupling}}}\cdot \left|s_{z,1}\right|$	
pole: $s_{p,1}=-\frac{1}{\left(R_{coupling}||R_{IN})(C_{coupling}||C_{IN}\right)}$					
**Area of predominantly capacitive coupling:** $\omega \geq 10\left|s_{z,1}\right|=\frac{10}{R_{coupling}C_{coupling}}$ no high-side frequency limit (only subcase A.1 from Table 1 is considered for each of the coupling layers)					
		**Case I**		**Case II**	
	
**Condition**		$\left|s_{p,1}|\leq |s_{z,1}\right|$ i.e., $\frac{C_{IN}}{C_{coupling}}\geq \frac{R_{coupling}}{R_{IN}}$		$\left|s_{p,1}\right|>|s_{z,1}|\;$ i.e., $\frac{C_{IN}}{C_{coupling}}<\frac{R_{coupling}}{R_{IN}}$	
**Cutoff frequencies**		$\omega_{R,I}=\frac{1}{\sqrt{\;{\left(\frac{1}{s_{p,1}}\right)}^{2}-2{\left(\frac{1}{s_{z,1}}\right)}^{2}}}$ $\omega_{C,I}=\sqrt{{s_{z,1}}^{2}-2{s_{p,1}}^{2}}$		$\omega_{R,II}=\frac{1}{\sqrt{\;{\left(\frac{1}{s_{z,1}}\right)}^{2}-2{\left(\frac{1}{s_{p,1}}\right)}^{2}}}$ $\omega_{C,II}=\sqrt{{s_{p,1}}^{2}-2{s_{z,1}}^{2}}$	
		They exist if $\frac{\left|s_{z,1}\right|}{\left|s_{p,1}\right|}=\frac{1+\frac{C_{IN}}{C_{coupling}}}{1+\frac{R_{coupling}}{R_{IN}}}\geq \sqrt{3}$		They exist if $\frac{\left|s_{p,1}\right|}{\left|s_{z,1}\right|}=\frac{1+\frac{R_{coupling}}{R_{IN}}}{1+\frac{C_{IN}}{C_{coupling}}}\geq \sqrt{3}$	
		**Area of predominant capacitive divider**$:\;\omega \geq \omega_{C,I}$$\;\mathrm{for}\;\mathrm{case}\;I\;\mathrm{and}\;\omega \geq \omega_{C,II}$ for case II			
**Area of minimized phase shifts**		$\omega \geq 10\left|s_{z,1}\right|=\frac{10}{R_{coupling}C_{coupling}}$		$\omega \geq 10|s_{p,1}|=10\left|s_{z,1}\right|\frac{1+\frac{R_{coupling}}{R_{IN}}}{1+\frac{C_{IN}}{C_{coupling}}}$	
**Subcases**		**I.1**	**I.2**	**II.1**	**II.2**
**Condition**		$R_{IN}<R_{coupling}$	$R_{IN}\geq R_{coupling}$	$R_{IN}<R_{coupling}$	$R_{IN}\geq R_{coupling}$
$C_{coupling}\geq 10C_{IN}$ **feasible?**		Never because $C_{IN}>C_{coupling}$	$C_{coupling}=10C_{IN}$ if $R_{IN}\geq {10R}_{coupling}$	Always	$C_{coupling}=10C_{IN}$ if $R_{IN}<{10R}_{coupling}$
**Area of predominantly capacitive coupling + area of predominant capacitive divider + area of minimized phase shifts**: $\omega \geq 10\left|s_{z,1}\right|$$\;\mathrm{for}\;\mathrm{case}\;I\;\mathrm{and}\;\omega \geq 10\left|s_{p,1}\right|$ for case II					

## Data Availability

The original contributions presented in this study are included in the article and its Supplementary Materials. Further inquiries can be directed to the corresponding author.

## Supplementary Materials

The following supporting information can be downloaded at https://www.mdpi.com/article/10.3390/s26041374/s1, Table S1: PRISMA 2020 for Abstracts Checklist [100,101]; Table S2: PRISMA 2020 Checklist [100,101]; Figure S1: Simplified PRISMA 2020 Flow Diagram [100,101] in accordance with the opening remark.

## Appendix A. Origin and Acquisition of Biopotential Signals

**Action potential and biopotential signals.** The principle of biopotential measurements relies on the fact that heartbeats, muscle contractions, and neural activity disturb the distribution of ions in the human body. This results in the process of cell depolarization: the intracellular ions flow outward across the cell membrane, whereas the extracellular ions enter the cells, which in turn raises the difference between the intracellular and extracellular electric potential from about −80 mV in the resting state to +30 mV [127,130,150,151]. The resting cell potential is restored through the reverse process of repolarization. This ion transport under stimulus results in a characteristic biphasic voltage waveform called action potential (Figure A1a), which represents the quintessential element of biopotential signals, giving insight into their origin. The difference in ionic concentration, as a result of the movement of ions during depolarization and repolarization in cells, creates electric dipoles. They source and sink the electric field, which allows ionic currents to spread throughout the fluids and tissue [41]. These ionic currents change in time and space. Hence, every single point on the body represents a different spatial and temporal summation of individual local biopotentials. Simply speaking, the biopotential signal, such as ECG (electrical activity of the heart) [130,152,153,154] (Figure A1b,c) or EEG (electrical activity of the brain) [120], is a result of the electric potential difference between two different points on the body. On the other hand, EMG signals (electrical activity of skeletal muscles) can be explained as a spatial and temporal summation of individual action potentials that are spreading throughout the muscle fibers during muscle contractions [130,156,157].

**Electrodes.** To enable the measurement and computational processing of these biopotential signals, a transducer is needed to convert the ionic currents in the body into electron currents in the electrical circuit. This crucial role is fulfilled by the electrode, which serves as the first component of the signal acquisition chain: the sensed biopotential, captured on the electrode sensing surface, becomes the electrical representation of the measured physiological signal (Figure A1d). For instance, deflections from the baseline as a result of heart activity and constellation of electrodes will compose the shape of an ECG biopotential signal. This way, the recorded biopotential signal can be further processed, filtered, and finally analyzed and classified, enabling health monitoring, clinical diagnostics, and vital functions assessment.

**Surface-contact biopotential electrodes.** Electrodes can approach the source of the biopotential signal very closely when applied under the skin and used within the body, which is achieved either with implantation (implantable, internal or indwelling electrodes) or by piercing the skin in the form of a wire or a needle (percutaneous electrodes) [74,158]. In either case, direct contact with the body implies penetrating the surface of the skin. Therefore, these electrodes are hypodermic-contact (subdermal, subcutaneous) and invasive. On the other hand, electrodes can be placed outside of the body, most commonly on the surface of the skin. Hence, these electrodes can be referred to as surface-contact electrodes. Most of these electrodes achieve minimally invasive contact without skin penetration, which makes them non-invasive. Although invasive electrodes provide better localization, higher accuracy, and complete information by approaching the source of the biopotential signal more closely, they are unsuitable for long-term measurements or monitoring during motion. Therefore, in the context of wearable devices that would allow for unobtrusive day-to-day biomonitoring, surface-contact electrodes, with their easier utilization, improved comfort, and significantly lower health risk, are the first choice. Thus, the rest of the paper will focus solely on non-invasive and surface-contact biopotential electrodes, as well as on their interfaces with the body. More on internal electrodes and microelectrodes can be found in [74,158].

## Appendix B. Surface-Contact Electrodes

To grasp the impact of placing a biopotential electrode on the surface of the skin, the electrical model of the skin and the coupling interface created between the skin and the electrode must be explained first. The following analysis and electrical model of the entire interface between a surface-contact electrode and the skin (Figure A2) are based on discussions provided in [74,111,127,130,158], where more details can be found.

**Biopotential signal source.** The biopotential signal source can be represented by a varying voltage source. This voltage source allows the ionic currents to flow and propagate through subcutaneous fat tissue, dermis, and epidermis, finally reaching the electrode sensing surface on the surface of the skin.

**Subcutaneous layer and dermis.** The skin plays a role of a protective barrier to the transport of chemicals and consists of three layers (strata): the deepest subcutaneous fat layer (hypodermis), the middle layer (dermis), and the upper layer (epidermis). In the dermis, vascular and nervous components, such as blood vessels, nerve endings and sensory receptors, hair roots, and sweat glands, can be found. With the exception of sweat glands, these components generate negligible electric potential. Hence, they do not contribute significantly to the electrical characteristics of the skin [158]. Because these layers can be considered as conductive media, they can be simplified to a pure resistance of inner body fluids and underlying tissues, represented with *R*_inner_ [145]. However, the tissue itself is non-homogeneous and anisotropic, acting at the same time as a low-pass filter [159]: the higher the propagating signal frequency, the greater the attenuation of the signal. A similar effect occurs for the greater the distance that a signal travels (i.e., for a thicker fat layer). The thickness of skin layers varies not only among different individuals but also among various body parts. Common values of *R*_inner_ found in the literature are in the order of 100 Ωcm^2^ and 1 kΩcm^2^ [160,161] (normalized to 1 cm^2^ electrode sensing area) at 10 Hz. This frequency is commonly used for measuring and expressing area-normalized impedance in biopotential sensing applications.

**Epidermis.** As the signal travels towards the surface of the skin, the dermis is followed by the outermost layer—the epidermis. It comprises five sublayers, the deepest being the stratum basale or stratum germinativum, where new cells divide and grow, and the superficial one being the stratum corneum (horny layer), where the old cells degenerate into dead material. This way, continuous regeneration and natural shedding of the skin is achieved. Just like the dermis, the lower four sublayers of epidermis are hydrophilic and present viable epidermis, whereas the superficial layer stratum corneum contains mostly dead material. Hence, the stratum corneum is electrically insulating, i.e., it can be modeled with a capacitor *C*_SC_ of areal capacitance in the order of 30 nFcm^−2^. At the same time, it exhibits a finite conductivity, which means that a resistor *R*_SC_ with an area-normalized value in the order of 100 kΩcm^2^ [124,162] can be placed in parallel with the capacitor *C*_SC_. The impedance value of this *RC*-parallel makes the stratum corneum the major contributor to skin impedance. However, this is true only up to frequencies of about 10 kHz. Namely, presence of the equivalent *RC*-parallel means that the stratum corneum acts as a high-pass signal filter: when a stable contact between the electrode and the skin is achieved, the impedance of the capacitive component starts to decrease as the signal frequency increases, eventually prevailing over the impedance of the resistive component. Thus, the overall impedance of the barrier layer and skin decreases with frequency [162]. Additionally, due to the existence of pores, hair follicles, and ducts, ions from the inner layers can still reach the skin surface. This additional conductive path of skin glands and ducts (skin appendages) can be represented by another *RC*-parallel combination of *R*_glands & ducts_ and *C*_glands & ducts_ [158]. More on human skin morphology, epidermal structure, and chemical, mechanical, and optical properties of the skin, can be found in [124,163].

**Electrode–skin or coupling impedance.** Finally, the signal reaches the non-invasive surface-contact biopotential electrode, most simply represented by a non-insulated metal plate. As explained in the last paragraph, the surface of the skin is, by nature, weakly conducting. Therefore, a conductive path is provided by applying an electrolytic paste or gel, which lowers the overall impedance of the contact with the electrode [120,124] and increases skin conductivity [127], allowing for easier charge transfer via a predominantly resistive (also called ohmic or conductive) coupling. This way, electrodes are immersed in an electrolyte, hence they are specifically called wet or gel surface-contact electrodes. The electrical equivalent of the resulting electrode–electrolyte–skin path can be referred to as the electrode–skin or the coupling impedance (Figure A2). It comprises the electrolyte impedance (*R*_electrolyte_ and *C*_air bubbles_ referred to as contact impedance) and electrode–electrolyte interface impedance (*R*_charge transfer_ and *C*_double-layer_, referred to as electrode impedance). Common area-normalized values of these two impedances are in the order of 1 kΩcm^2^ and 10 kΩcm^2^ at frequencies of interest (Assumption 1) [110]. They will be analyzed in more detail in the following paragraphs.

**Electrolyte impedance.** In general, just like internal body and tissue resistance *R*_inner_, skin conductivity also depends on various parameters, such as age, hydration, and measurement location [124,164]. Additional inhomogeneity and conduction losses in the electrolyte can be accounted for and represented in the model by a series contact resistance *R*_electrolyte_. Also, in the case of incomplete adhesion of the electrode to the skin, air bubbles might be present between the electrode and the skin, forming an equivalent capacitor *C*_air bubbles_ in parallel with electrolyte resistance *R*_electrolyte_ [130]. Further changes in electrolyte impedance would be produced with various cosmetics applied on the skin [165].

**Electrode–electrolyte interface and electrode impedance.** The electrode impedance formed at the electrode–electrolyte interface can now be analyzed in more detail. At this interface, there are two charge transfer mechanisms that allow for a conversion between ionic and electron currents: non-faradaic and faradaic, which are described in detail and compared in [74,127,158,166].

**Non-faradaic process.** In the case of a purely non-faradaic process, charge accumulates across the interface instead of crossing it. This way, a double layer of charge is formed at the electrode–electrolyte interface according to the Helmholtz double-layer effect, thoroughly described in [167]. The electrode–electrolyte interface acts as a capacitor *C*_double-layer_. Hence, there is no direct current (DC current) flow, and the charge transfer is instead based on the displacement current as a result of capacitor charging and discharging, which defines the electric potential difference across the electrode–electrolyte interface [74].

**Faradaic process and half-cell potential.** On the other hand, in a purely faradaic process, charge can flow through the electrode–electrolyte interface. Therefore, DC current is not blocked and the interface between the electrode and the electrolyte can be described as a resistor, called the charge transfer resistor, *R*_charge transfer_ [168]. The charge transfer across the interface is now based on electrochemical reaction of reduction (the process of receiving electrons and creating anions) and its reverse reaction of oxidation (the process of losing electrons and creating cations) [158]. The DC current is permitted to flow, and the amount of charge transferred is proportional to the current flow, which is in accordance with Faraday’s laws of electrolysis and explains why this process is called faradaic [169]. Predominant electrode oxidation will result in electrons flowing from the electrode to the electrolyte, whereas predominant electrode reduction will result in electrons flowing from the electrolyte to the electrode [158]. Therefore, just like in the case of a non-faradaic process, an electric potential difference exists across the electrode–electrolyte interface. With time, the rate of electron loss (oxidation) becomes equal to the rate of electron gain (reduction). Then, the state of dynamic equilibrium is achieved, and the electric potential difference that is established as a result of ionic distribution is called the half-cell potential or contact potential, *E*_electrode–electrolyte_. Namely, each wet surface-contact electrode, being in essence a non-insulated metal plate immersed in an electrolyte, represents one half-cell. Its half-cell potential is measured relatively with respect to the standard hydrogen electrode, which is defined as the reference half-cell system with a zero half-cell potential. The measured difference is known as the standard electrode potential or standard reduction potential. The greater and more positive the standard reduction potential, the higher is the tendency of a metal to be reduced, i.e., to accept electrons and form anions. The Nernst equation [158] gives correction for the half-cell potential, taking into account that the conditions (temperature, pressure, and ionic concentrations) may differ from the standard state (298.15 K temperature, 1 bar of pressure, and 1 M of concentrations) [170]. In the context of biopotential measurements, half-cell potential or contact potential of a wet surface-contact electrode is often used as a synonym for standard electrode potential or standard reduction potential, which will also be the case for the rest of this paper. A step further in the general theory of electrochemistry would consider two electrically connected half-cells, which would form a full galvanic or voltaic electrochemical cell. With regard to electric current and electrons in the wire, the half-cell with predominant oxidation sources the electrons and is called the anode, whereas the other half-cell with predominant reduction attracts the electrons and is called the cathode. Conversely, regarding ions in the electrolyte, the anode attracts anions, whereas the cathode attracts cations. The difference between the cathode and anode half-cell potentials, called the cell voltage, could be used as a supply voltage, which is exactly how batteries work (Figure A3) [170]. More on electrode charge transfer processes from the perspective of energy storage in batteries and supercapacitors can be found in [171,172].

**Polarization and DC electrode offset.** However, in such events of current flow, the previously described half-cell potential as the inherent equilibrium state is not the only electric potential difference that is established within the electrode–electrolyte interface. Namely, when current exists or is externally applied, additional potential difference is created as a result of polarization [166], and the total electric potential difference is now described by the polarization overpotential. In [158], this polarization phenomenon due to current flow is separated into three components: ohmic overpotential as an additional voltage drop created across the electrolyte due to its finite resistance *R*_electrolyte_, concentration overpotential as a result of disturbance in the distribution of ions at the electrode–electrolyte interface, and activation overpotential as a result of unequal energy needed for oxidation and reduction. Together with the half-cell potential, these three overpotentials, present when current flows between the electrode and the electrolyte, add up to the net overpotential of an electrode, called the polarization potential. The polarization potential difference between two electrodes is then called polarization voltage, contact voltage, or DC electrode offset. The choice and design of wet surface-contact electrodes aim to minimize the polarization overpotential. That way, most of the electric potential difference across the electrode–electrolyte interface would originate from the inherent half-cell potential and, consequently, most of the DC electrode offset between two electrodes would originate from the difference between their half-cell potentials.

**Electrode polarizability, Ag/AgCl electrodes, and manufacturing considerations.** The polarization effect is in fact the root cause of *C*_double-layer_. Theoretically speaking, in a perfectly polarizable electrode, only displacement current and no conduction current would be present at the interface. Hence, the electrode–electrolyte impedance model would be simplified to *C*_double-layer_ alone, which is in accordance with the pure non-faradaic process. Conversely, perfectly non-polarizable or reversible electrodes would offer no resistance to DC current flow at all. In that case, only conduction current and no displacement current would be present, meaning that their interface with the electrolyte would be equivalent to an electrical short or, more realistically, a finite resistor *R*_charge transfer_, and behave in accordance with the pure faradaic charge transfer process. Such non-polarizable electrodes do not introduce significant overpotential in addition to the inherent half-cell potential, hence, they are preferred for biopotential signal measurements and whenever DC and low-frequency recordings are considered [127]. In [166], more on comparison between polarizable and non-polarizable electrodes with regard to the faradaic and non-faradaic process can be found. In reality, faradaic charge transfer and non-faradaic charge separation occur simultaneously at the electrode–electrolyte interface. In other words, the double-layer effect and the polarization effect are virtually always present, and the total current flowing through the interface comprises both the non-faradaic displacement current due to ion accumulation at the double layer and the faradaic current as the electron transfer due to reduction–oxidation (redox) reactions. Therefore, just like the equivalent stratum corneum impedance, the impedance of the electrode–electrolyte interface (the electrode impedance) can be modeled as an *RC*-parallel combination of *R*_charge transfer_ and *C*_double-layer_ (Figure A2). Both of these components, along with *R*_electrolyte_, are additionally dependent on frequency, as well as on current density, which is why electrodes for electric stimulation require additional considerations with respect to parameters of the applied stimulus [158]. Since both *R*_charge transfer_ and *C*_double-layer_ exist in real-life applications, most electrodes are polarizable, with a degree of polarity defined by the values of *R*_charge transfer_ and *C*_double-layer_. To determine electrode polarizability for various metals, a polarization test can be used, along with measuring a response to a voltage pulse [127,158]. Among the non-polarizable electrodes preferred for biopotential sensing, the most popular are the Ag/AgCl wet electrodes. They consist of a silver base coated with a thin layer of silver chloride and interfaced with a chloride salt, offering weak polarization, i.e., good DC conductivity and a low value of *R*_charge transfer_ [168]. While this is important in order to reduce the DC electrode offset down to the difference between electrode half-cell potentials, another important property of Ag/AgCl electrodes is a low and stable half-cell potential, demonstrated in [127,158]. This allows for minimization of the half-cell potential difference itself. Namely, even in the complete absence of polarization effect and overpotential components, there still exists a remaining DC electrode offset as a result of half-cell potential difference. It is created as a result of using electrodes that are not identical in design, material, electrochemical properties, and electrolyte concentrations, and this remaining DC electrode offset could still easily reach the order of several 100 mV and drive the amplifier into saturation [139]. This explains the importance of careful electrode construction, which also means ensuring that all parts of the electrode, which are exposed to the electrolyte, are fabricated from the same material. Otherwise, dissimilar metals in contact with the same electrolyte will result in an additional unwanted electrochemical reaction and possible corrosion. These manufacturing considerations also apply to the choice of bonding method and material (soldering, welding, crimping, peening, etc.) and, likewise, to the choice of materials for electric stimulation [158].

**Electrolyte–skin interface.** In addition to the electrode–electrolyte half-cell potential, another half-cell potential is present within the electrolyte–skin interface. Namely, any difference in ionic concentration across the stratum corneum results in another electrochemical reaction, and the corresponding electric potential difference can be modeled as a voltage source *E*_electrolyte–skin_ in series with the stratum corneum impedance (Figure A2) [158].

**Electrolyte–electrolyte interface.** Lastly, the third electric potential difference exists within the electrolyte–electrolyte interface: the liquid-junction potential *E*_electrolyte–electrolyte_, which is a result of the difference in concentration and mobility of ions in sweat ducts and glands on one side and the surrounding dermis and subcutaneous layer on the other side [158].

**Skin potential and electrodermal activity.** These two additional DC voltage sources, *E*_electrolyte–skin_ and *E*_electrolyte–electrolyte_, help to explain the inherent skin conductivity, as well as the skin potential—an inherent electric potential difference between the inside and outside of the stratum corneum. The typical value of the skin potential, mostly corresponding to *E*_electrolyte–skin_, is about 30 mV, but it changes in the order of 1 mV when the skin is stretched or compressed by the electrode [74,128,143,158,160]. Likewise, additional electric potential difference can be measured between the injured skin site and the intact skin around the wound [173]. In general, the skin potential depends on several biological factors such as skin thickness and hydration. Therefore, just like the elasticity and thickness of skin layers, it varies not only with age and location on the human body, but also among individuals. Several theories have been developed to explain its origin [174,175]. For instance, the theory of injury current [102,174,176,177] considers the process of skin shedding, when older skin cells are pushed towards the surface, which creates cracks in the stratum corneum. The sudden flow of ions through the cracks creates the so-called injury current, which results in a voltage drop across the equivalent stratum corneum resistance. The described properties of inherent skin conductivity and skin potential are often termed electrodermal response or galvanic skin reflex. Accordingly, skin electrical properties that originate from the transportation of moisture and ions from sweat glands to the stratum corneum define the electrodermal activity of the skin. More on the topic of skin potential, skin conductivity, electrodermal response, and electrodermal activity monitoring can be found in [164,178,179,180].

**Overview of alternative interface models.** Although the presented model is the most often used model for non-invasive biopotential measurement, other models of skin layers, electrolyte–skin interface, and electrode–electrolyte interface have also been developed. For instance, for the electrode–electrolyte–skin path, alternative two-resistor and even one-resistor models could be used [181,182]. Studies similar to [181] revealed that the resistive component of such models is more dependent on the individual’s skin properties, whereas the capacitive component is more dependent on the properties of the electrode and the electrolyte. Furthermore, [163] accounts for interlayer skin impedances, considering *RC*-layered structures not only vertically (transversally), but also horizontally (longitudinally). Moreover, instead of using capacitor components, constant phase elements (CPEs) [161,163,165,167,168,182,183] can be employed with the use of a fractional-order Laplacian operator [168,184]. Such models focus more on biological properties of the skin rather than its electrical properties and layered structure. In that sense, they adopt a generalized capacitor behavior that deviates from the ideal capacitive impedance behavior, accounting for current density inhomogeneity due to electrode sensing surface irregularities and moisture content. Among these CPEs, diffusion impedance or Warburg impedance can be used to describe the additional diffusion of ions as another example of a faradaic process occurring in the case of skin with a large water or sweat content [163,168]. Finally, to expand the model to higher frequencies in the order of 1 MHz and account for frequency-dependent effects in biological media, various bioimpedance models have been developed [56,75,167,185]. More on human skin morphology and its various properties can be found in [124,163]. More on modeling the electrode–electrolyte–skin path, electrode polarization, and spread impedance that defines the spread of current in tissue under the measurement site, can be found in [168,185]. Useful calculation examples discussing polarization effect and electrode behavior can be found in [158].

**Drawbacks of wet electrodes and alternative mechanical solutions.** Wet surface-contact electrodes, as a widely adopted clinical practice and the gold standard for research applications, inspire confidence due to their well-understood properties and lack of electromagnetic complexity. Even with low-cost, easily disposable, pre-gelled wet electrodes, reliable high-resolution clinical-grade biopotential signals can be recorded. Consequently, research focused on the machine learning and signal processing aspect does not have to be burdened with the possibility of an electrode negatively affecting the measurements. Furthermore, the application of a gel reduces the impedance of the signal path, ensuring good signal quality and excellent adhesion to the skin. However, there are several drawbacks to their utilization regarding convenience and subject’s comfort. Namely, to minimize and stabilize the stratum corneum impedance, as well as minimize the skin potential variation, alongside cleaning, an abrasive process of grease, hair, and particles removal is applied on the surface of the skin, e.g., by rubbing it with a fine sandpaper. This way, the stratum corneum and its equivalent *R*_SC_*C*_SC_-parallel is virtually removed. While this process proves to be the most effective skin preparation procedure [143,160,186], it also leaves the skin unprotected. Thus, it becomes more susceptible to irritation, which requires electrode and electrolyte biocompatibility consideration. Skin redness, swelling, rash, and contact dermatitis may be caused, as well as (although rare) allergic reactions [187], especially in the case of newborn skin [188]. The risk of dermal reaction also depends on the degree of skin sensitivity and dryness, as well as skin response to sunlight, which can be categorized with the Prokerala and Fitzpatrick test, respectively, and that risk increases with larger electrode sensing areas and longer durations of use [189]. The problem is further aggravated when the feasibility of measurement on burned skin [39] or in any other sensitive area is considered, such as the one near the eyes. Extensive preparation is also required for animal monitoring [36]. Additionally, after the measurement, the removal of gel residue must be thoroughly performed, which precludes the usage of wet electrodes without technical assistance or outside of the medical facilities. Furthermore, skin treatment and gel removal can be an unpleasant and stressful process for the subject, as well as a tedious and time-consuming process for the technical staff, especially when a large number of electrodes is used. Moreover, although higher amounts of electrolytes could speed up the process of impedance reduction and achieve higher conductivity of the interface, the risk of cross-coupling via leakage currents and short circuits between neighboring electrodes increases, especially in the case of smaller inter-electrode distances. Finally, the stratum corneum regenerates itself every 1–2 days, which requires iterative skin treatment. To make matters worse, the applied gel eventually dries out due to body heat and airflow. This results in an increase in impedance of the electrode–electrolyte interface, and also, electrodes might start to wrinkle and peel off. Hence, exchange of electrodes and an iterative gel application may be needed even more frequently than skin treatment to prevent impedance variation and signal degradation. All these culprits prevent wet surface-contact electrodes from being straightforwardly used for continuous monitoring, which is essential for the detection of sparse abnormalities and unobtrusive sensing in a home environment. To bypass these disadvantages, alternative mechanical solutions can be employed—suction electrodes that reduce smearing, floating electrodes that reduce the influence of motion during recording [74,158], and kirigami hole patterns for enhanced stretchability and breathability [190].

**Semi-dry electrodes.** Another possibility is to use semi-dry or quasi-dry surface-contact electrodes [122,123], which provide controlled moisturization and even distribution of a small amount of conductive fluid from an internal reservoir. These electrodes maintain a similar interface with the skin as wet electrodes (Figure A2), but with a higher electrolyte resistance due to the inclusion of porous columns in the reservoir [74]. To resolve the issue of signal instability due to inadequate electrolyte release, a foam layer can be used [123]. A comparison of semi-dry and standard wet electrodes can be found in [191], where this novel concept was first proposed in 2013. An alternative way to control the electrolyte flow and enhance the stability of the gel interface for long-term monitoring in hairy areas is to perform reversible electrolyte phase transition with the use of biogels with temperature-controlled gelatinization [192]. Also, ionic liquid gel solutions [193], as well as hydrogel-based sensors [126,194] and similar gel-based patches [195,196], are available as another compromise for achieving an increase in wearing time and alleviating the need for cleaning and wiping the measurement site after use.

**Dry electrodes.** To further enhance ease of use and comfort during prolonged wearing, electrolyte can be entirely removed, leaving the skin dry. In this case, a conductive path and resistive coupling with skin is created without the electrolyte; hence, these electrodes are known as dry contact or dry surface-contact electrodes. As gel-free (also called gel-less and paste-less) sensors, they present a more user-friendly approach to surface biopotential measurements, reducing the time needed for preparation, cleaning, and repositioning and removal of the electrodes, thereby facilitating long-term measurements and application in emergency settings and field hospitals. By being non-disposable, they also allow for multiple and repeated measurements. However, these benefits come with an abundance of new challenges: because there is no explicit wet coupling medium and the dedicated gel layer is removed, conductivity of the interface is significantly lower. The total coupling impedance between a dry surface-contact electrode and skin can easily be 10 times higher than in the case of wet surface-contact electrodes and reach even the order of 1 MΩcm^2^. Particularly challenging are areas rich in hair: if no depilation preceded the recordings, hair would hinder the quality of contact, reducing the effective contact area between the electrode and the skin. The existence of this hair component would be equal to adding another *RC*-parallel to the model in Figure A2 (refer to Figure 2 in [139]). In spite of having a dry electrode–skin interface, a certain degree of moisture is achieved through perspiration. Namely, in a sense, body sweat represents a natural electrolyte (a 0.3% NaCl salt solution [178]). Therefore, it could replace the missing electrolyte component in Figure A2, allowing dry electrodes to achieve greater similarity to the wet electrode–skin interface and improve their performance over time (refer to Figure 3 in [197] for example). Naturally, just like in the case of wet surface-contact electrodes, the chemical response of electrode material to perspiration must be inspected beforehand (e.g., this is the reason why stainless steel sensing surface is preferred to aluminum in [197]). However, sweating is a rather unreliable and not easily controllable moisturization process that is dependent on various factors such as ambient conditions, physical activity, and emotional state. In fact, variations in its quantity translate into variations in half-cell potentials, so the effect of time-variant DC electrode offset might easily be even more pronounced than in the case of wet electrodes [139]. Various aspects of wet, semi-dry, and dry surface-contact electrodes are compared side-by-side in [110,121,122,182,183,198,199].

**Alternative dry solutions: pointed, puncturing, polymer-based, flexible, epidermal, textile, and nanomaterial-based design.** Especially in the case of measurements on scalp and in hair-rich areas, contact can be strengthened with the use of various pointed electrodes [122]: multipins [200] can be employed, as well as flexible prongs [201], sliding and spring-loaded pins [202], claw-shaped electrodes [203], and brush-like bristle electrodes [204]. Such pointed design also comes in handy in animal monitoring applications [205]. However, stability of individual pin contact can be an issue, as well as pressure marks and bruises caused by protruding parts. A step further in decreasing the skin impedance and skin potential variation would be to perform skin abrasion, as demonstrated in [198,199], or even completely bypass the stratum corneum (i.e., short the *R*_SC_*C*_SC_-parallel in Figure A2) with the use of microneedle arrays based on microelectromechanical systems (MEMSs). In that case, dry surface-contact electrodes become invasive. Examples of these electrodes can be found in [122,123,206,207,208]. Specifically, Miura-ori structures with ventilation channels can be used for improved air circulation [209]. Such puncturing spike structures can significantly increase the conductivity of the interface but naturally present a serious health hazard. Fracture strength must be guaranteed to avoid fracture of microneedles in the skin. Also, the possibility of infection is significantly increased, adding pain to the previous issue of discomfort. To overcome these difficulties and yet maintain a non-invasive measurement principle with minimized skin preparation, coating material, geometry, and mechanical properties of dry surface-contact electrodes start to play a vital role in accomplishing a stable contact with reduced coupling impedance. For instance, medical tape, double-sided adhesive tape, and conductive glue adhesive can be replaced with advanced adjustable mechanical solutions [210], but also with hook-and-loop straps, wristbands, belts, and other types of supporting systems and electrode holders, such as headsets, helmets, compression vests, and smart shirts [201,211]. However, although rigid metal electrodes achieve the lowest coupling impedances, as measured in [212], they hinder the goal of achieving lower half-cell potentials and may end up being corroded by sweat. In addition, metallic coating makes electrodes unsuitable for procedures such as an X-ray scan or magnetic resonance imaging (MRI) exam. Instead, electrodes can be fabricated from durable and washable materials, such as conductive rubber [213] or other conductive polymers [214]. Also, dry Ag/AgCl electrodes could be fabricated [215]. Furthermore, special bionic patterns, inspired by physiological microstructures of living organisms, such as octopus suckers and gecko feet, could be fabricated to provide adaptive conformability, enhance mechanical stability and adhesion, and eliminate excessive sweat [114,115,116]. Another way to conform to a curved body surface and reduce skin indentation, as well as possibility of electrode slipping, is to fabricate porous, compressible, sponge-based electrodes [123,216] and flexible hybrid electrodes that combine conventional rigid design with soft flexible substrates, or even fully flexible electrodes [217,218,219]. Printed circuit board (PCB) technologies for the fabrication of electrochemical sensors on rigid and flexible substrates are discussed in [220]. A further reduction in electrode thickness enables the creation of thin sheet electrodes [221] and tattoo electrodes [118]—epidermal electronic systems [222] with kirigami patterns, serpentine-shaped interconnects, and fractal layout for improved stretchability and deformability performance [223]. More on history and recent progress in such materials, as well as their structure and fabrication process, can be found in [112,113,114,115,116,117,118,119,124]. To further enhance wearability and ease of application, instead of embedding rigid electrodes into clothing as in [211], electro-textile or e-textile can be used to manufacture sensing surfaces and create textile electrodes (textrodes). For this purpose, aside from using conductive material fibers, conductivity can be achieved by filling the fibers with conductive particles or coating them with a conductive layer. Strands of conductive fibers—either threads or yarns—can be incorporated into fabric by means of embroidering, weaving, and knitting to achieve mechanical robustness and allow for long-term stretchability and wearability, enabling the concept of fabric circuit boards (FCBs) [85,224,225,226]. Alternatively, fibers can be bonded mechanically, thermally, and chemically into nonwoven fabric [227,228]. Similar material bonding methods can later be used to integrate conductive patterns and electronics into the fabric, along with various printing technologies, such as inkjet printing and screen-printing, or with the use of laser writing. More on various textile fabrication methods and their properties can be found in subchapters 3.3 and 3.6 in [85], as well as in [127,128,130,135,226,229,230]. More specifically, printable technologies are described in subchapter 3.4 in [85] and in [128,130], and in [131] compared to traditional fabrication techniques, such as photolithography and vacuum deposition. The mechanical stability of textile electrodes can be improved further by using, e.g., soft padding support [231] and elastic hook-and-loop structures [232]. Additionally, specialized multi-layered fabric design [233] and thin stretchable textile [234] can be used. A comparison of textile electrodes and traditional Ag/AgCl electrodes is available in [226]. A comparison of various rigid dry surface-contact electrodes with metallic, polymer, and textile sensing surface can be found in [212]. A similar comparison, but between electrodes laminated with textile, is given in [189]. On the other hand, [235] brings a comparison between several fully textile dry surface-contact electrodes. Lastly, [85] expounds on using smart textiles in exoskeleton technology, whereas [225] gives prospects of using garments for functional electrical stimulation as an alternative to standard discrete gel surface-contact electrodes. More on important properties of fabrics, such as surface resistivity, wearability and comfort, tensile strength, elasticity, compression resistance, and tightness, washability and durability, air permeability and breathability (water vapor transmission), biocompatibility and non-cytotoxicity can be found in [123,124,126,129,130], as well as in [134,135,136,229] from the perspective of textile antennae design. Reviews on biocompatibility and biodegradability are given in [132] and [133], respectively. Another way to enhance the mechanical properties and electrical conductivity of the described dry solutions, from stretchable to penetrating, is to combine them with nanomaterials [111,236,237,238]. For instance, polymer nanofibers can be used to create biomimetic fractal-like microstructures for flexible sensors [239]. Especially popular are carbon-based nanomaterials, such as carbon nanotubes (CNTs). However, skin compatibility and safety of nanomaterials is at the moment still an active area of research [238]. In any case, just as compatibility between materials with respect to their electrochemical properties must be checked in the case of wet surface-contact electrodes, long-term characterization must be inspected for dry surface-contact electrodes as well, especially if they are intended for prolonged monitoring [189,198,240].

## Appendix C. Fundamentals of Capacitors and Phasor Algebra

Simply speaking, Equation (2) showed that for a capacitor with a negligible amount of variation in *C*_coupling_, a change in voltage *v*_coupling_(*t*) across it must occur for *i*_dis_(*t*) to flow. Rate of voltage change in time will translate into current value, whereas the sign of that voltage change will translate into current direction (Figure A4). When voltage across the capacitor is increased, the capacitor acts as a load and charges, drawing current from the positive polarity side. As a result, energy is stored in its dielectric material by means of electric field. On the other hand, when voltage across the capacitor is decreased, the capacitor will react as a source and discharge, supplying current to the positive polarity side. Because adding and removing charge takes time, the change in voltage across the capacitor will be zero in the events of transient changes (*dt* infinitesimally small). In other words, as the frequency increases (meaning faster charging–discharging cycles), a capacitor effectively turns into a short. In the steady state, this can also be observed in the frequency domain with the use of rotating phasors [265,266,267,268]. Rotating phasor $\dot{I_{dis}}e^{j\omega t}$, as a complex vector, stems from Euler’s formula [266,269]. Its static part or phasor ${\dot{I}}_{dis}$ contains information on magnitude *I*_dis_ and starting phase *φ*_dis_ (A1):

(A1) $$i_{dis}\left(t\right)=I_{dis}\cos\left(\omega t+\varphi_{dis}\right)=Re\left\{I_{dis}e^{j\left(\omega t+\varphi_{dis}\right)}\right\}=Re\left\{I_{dis}{e^{j\varphi_{dis}}e}^{j\omega t}\right\}=Re\left\{\dot{I_{dis}}e^{j\omega t}\right\}\;,$$

where *ω* is the angular frequency (A2) and *j* is the imaginary unit (*j*^2^ = −1):

(A2) ω=2πf .

Applying phasor calculation to the time derivative in (2) gives the well-known equation for impedance of a capacitor *Z*_Ccoupling_ and its reactance *X*_Ccoupling_ (A3,A4) [267]:

(A3) $$i_{dis}\left(t\right)=C_{coupling}\frac{d}{dt}\left(\dot{V_{\mathrm{coupling}}}e^{j\omega t}\right)=C_{coupling}\cdot \dot{V_{\mathrm{coupling}}}\cdot \frac{d}{dt}\left(e^{j\omega t}\right)=j\omega C_{coupling}\cdot \dot{V_{coupling}}e^{j\omega t}\;,$$

(A4) $${\;Z}_{Ccoupling}\left(j\omega \right)=\frac{\dot{V_{coupling}}e^{j\omega t}}{\dot{I_{dis}}e^{j\omega t}}=\frac{\dot{V_{coupling}}}{\dot{I_{dis}}}=\frac{1}{{j\omega C}_{coupling}}=-j{\;X}_{Ccoupling}\left(\omega \right)\;.$$

The dependence of *X*_Ccoupling_ and *Z*_Ccoupling_ on frequency (A4) corroborates that a capacitor acts like a frequency-dependent resistor. It blocks DC current (0 Hz), operating as an alternating current (AC) coupler and a low-impedance path for higher frequency components. In addition, the (−*j*) term, which originates from the time derivative and multiplies *X*_Ccoupling_, indicates that current *i*_dis_(*t*) leads voltage *v*_coupling_(*t*) by a phase difference of 90° or π/2 rad [265,266]. Consequently, the average value of instantaneous power *v*_coupling_(*t*)*i*_dis_(*t*) over one period *T* is zero (A5) [264,267,268]. This means that an ideal capacitor does not dissipate energy under periodic excitation, but rather stores it in the electric field during charging and releases it during discharging. This represents the so-called lossless property of a capacitor:

(A5) $$\bar{{v_{coupling}\left(t\right)\cdot i}_{dis}\left(t\right)}=\frac{1}{T}\int_{0}^{T}V_{\mathrm{coupling}}\cos\left(\omega t+\varphi_{dis}-\frac{\pi }{2}\right)\cdot I_{dis}\cos\left(\omega t+\varphi_{dis}\right)dt=0\;.$$

## Appendix D. First Subsystem: Operational Amplifier

**Introduction to Appendix D.** Chronologically, the content of this appendix belongs to the beginning of Section 4.1, where it is cross-referenced. Therefore, it is written from the perspective of a content that precedes the content of Section 4. As explained at the beginning of Section 4.1, the purpose of this appendix is to develop and analyze the model of a voltage follower amplifier. In accordance with Assumption 5, that model represents the first out of two subsystems that build the model of a buffer active non-contact and/or insulated biopotential electrode and its interface with the body. In that sense, the analysis provided in this appendix is a prerequisite for Section 4, where a voltage follower amplifier will be employed as the buffer preamplifier subsystem. Throughout the analysis, the fundamentals of operational amplifiers, negative feedback, Bode plot, and amplifier stability are recalled based on the following literature on operational amplifiers: [315,330,331,332,333,334,335]. The analysis is also accompanied by datasheets of several amplifiers commonly employed as preamplifiers for buffer active electrodes: LMP7721 [336] and OPA129 [337] (Texas Instruments, Inc., Dallas, TX, USA), and AD8641 [338] (Analog Devices, Inc., Wilmington, MA, USA).

**Common-mode and differential-mode components.** A standard operational amplifier is essentially a differential amplifier that amplifies the voltage difference between its non-inverting and inverting inputs into a single-ended output voltage. In general, the input voltage can be separated into two components: the normal-mode or differential-mode input voltage *v*_diff_ (the potential difference between the two inputs) and the common-mode input voltage *v*_com_ (average voltage with respect to the circuit common, present at both inputs, with a return current path through the circuit common) (Figure A5). In that sense, *v*_com_ considers the electric potential of the input terminals that are tied together (shorted) with respect to the circuit common. Voltages *v*_diff_ and *v*_com_ can be defined as in (A6) and (A7):

(A6) $$v_{diff}=v_{+}-v_{-}\;\;,$$

(A7) $$v_{com}=\frac{v_{+}+v_{-}}{2},$$

where *v*_+_ and *v*_−_ are single-ended voltages present at the non-inverting and inverting input terminal, respectively. “Plus” and “minus” symbols assigned to the input terminals indicate that the sign of the single-ended output voltage corresponds to the sign of the voltage difference in (A6), whereas the terms “non-inverting” for the “plus” and “inverting” for the “minus” input terminal indicate that the output voltage will be inverted if the input voltage is applied to the inverting terminal.

Equations (A6) and (A7) indicate that the amplifier unloaded open-loop input voltage gain (where the term “open-loop” denotes that there is no output-to-input feedback present), *a*, also consists of the differential-mode component, *a*_diff_, and the common-mode component, *a*_com_. This defines the single-ended output voltage *v*_out,open-loop_ as follows (A8):

(A8) $$v_{out,open\text{-}loop}=a_{diff}v_{diff}+a_{com}v_{com}\;.$$

**Control system representation and negative voltage feedback.** An ideal operational amplifier amplifies only the useful, differential-mode input voltage *v*_diff_, and entirely rejects the undesirable common-mode input voltage *v*_com_ (i.e., *a* ≈ *a*_diff_ and *v*_out,open-loop_ ≈ *a*_diff_*v*_diff_). Ideally, this means that a change in both input terminal voltages *v*_+_ and *v*_−_ for the same small amount with respect to the circuit common will not show up at the output. Therefore, a standard practice for the analysis of an ideal operational amplifier is to consider *a*_diff_ to be infinite, which is justifiable given that *a*_diff_ commonly reaches values of 90 dB and higher at frequencies of interest (Assumption 1) [336,337,338]. Such a high *a*_diff_ means that the slightest difference between *v*_+_ and *v*_−_ or the slightest internal voltage offset is sufficient to drive the amplifier into saturation and clip off its output at the maximum output voltage swing limits. Closing the loop with a negative feedback specifically designed as series-input–parallel-output feedback called series–parallel or series–shunt feedback [334,340,341,342] (Figure A6) will feed a fraction of the output voltage back to the inverting input terminal and subtract that portion from the differential voltage present at the input, hence the term voltage-feedback operational amplifier (for other variants of negative feedback, refer to [334,340], and for differences between voltage-feedback and current-feedback amplifiers specifically, refer to [343]). The amount of output voltage *v*_out,closed-loop_ = *v*_out_ that is fed back to the input is controlled by the feedback factor *β*. Subtracting the attenuated version of the output voltage from the input will act in a way that minimizes the difference between the voltages applied to the input terminals, *v*_+_ and *v*_−_: a greater difference between *v*_+_ and *v*_−_ will produce a greater *v*_out_, which will decrease the difference between *v*_+_ and *v*_−_, and vice versa. Hence, according to the concept of a virtual short, input terminals are forced to the virtually same electric potential with respect to the circuit common, and so *v*_+_ and *v*_−_ reside at the voltage equal to *v*_com_. This leads to the schematic representation of a typical non-inverting and inverting operational amplifier in Figure A7.

**Input and output impedances and voltage-controlled voltage source model**. Similarly to definitions of *v*_com_ and *v*_diff_, as well as *a*_com_ and *a*_diff_, two types of small-signal input impedance can be specified. The first one is the common-mode input impedance *Z*_com_, and the second one is the differential-mode input impedance *Z*_diff_. The common-mode input impedance *Z*_com_ is observed with respect to the circuit common either from the non-inverting input terminal (*Z*_com+_) or from the inverting input terminal (*Z*_com−_). On the other hand, the differential-mode input impedance *Z*_diff_ is the impedance between the two amplifier input terminals. In accordance with the model of a voltage-controlled voltage source [340], to allow for the transfer of the entire source voltage to the input terminals without the loading effect, input impedance should be infinite. Concomitantly, to allow for the transfer of the entire amplifier output voltage to the load or input of the next stage, amplifier intrinsic open-loop output impedance *Z*_out_ should be zero. Modeling of *Z*_com_, *Z*_diff_, and *Z*_out_ will be further explored in the rest of this appendix.

**Non-inverting frequency-independent model.** Specifically, a non-inverting configuration will be in focus of this paper and this appendix. In that case, the input signal is applied to the non-inverting input terminal (*v*_in,non-inv_ in Figure A7). Considering again only the differential-mode open-loop voltage gain *a*_diff_, the closed-loop differential-mode voltage gain *A*_diff_ can be defined in accordance with the non-inverting amplifier block diagram (Figure A6). For simplicity’s sake, *a*_diff_ can be referred to as the open-loop gain, and *A*_diff_ can be referred to as the closed-loop gain. Since the operational amplifier in this configuration effectively amplifies the single-ended input *v*_in_ = *v*_in,non-inv_ into a single-ended output *v*_out_, *A*_diff_ is often also referred to as the signal gain. For the simplest case of ideal frequency-independent frequency response of the amplifier (infinite bandwidth) [334], the resulting relation stems from Figure A6a as follows (A9):

(A9) $$A_{diff}=\frac{v_{out}}{v_{in}}=\frac{1}{\beta }\frac{1}{1+\frac{1}{a_{diff}\beta }}=\;\frac{a_{diff}}{1+a_{diff}\beta }\;\;,$$

where *a*_diff_ is the open-loop gain or forward-path gain (*a* ≈ *a*_diff_), *β* is the feedback factor, *a*_diff_*β* is the loop gain, and 1 + *a*_diff_*β* is the amount of feedback, return difference, desensitivity, or the sacrifice factor. The feedback factor *β* can be expressed with the resistance of the feedback path, *R*_f_, and the resistance present at the inverting input terminal, *R*_−_ (A10):

(A10) $$\beta =\frac{v_{feedback}}{v_{out}}=\frac{v_{-}}{v_{out}}=\frac{R_{-}}{R_{-}+R_{f}}\;\;.$$

As previously discussed, the open-loop gain is so high—i.e., loop gain *a*_diff_*β* >> 1 in (A9)—that the closed-loop gain *A*_diff_ ideally becomes defined by 1/*β*—that is, by the components of the external feedback network. For the basic non-inverting configuration, this yields the following (A11):

(A11) $$A_{ideal}\approx A_{diff,ideal}=\underset{a_{diff}\to \infty }{lim}A_{\mathrm{diff}}=\underset{a_{\mathrm{diff}}\to \infty }{\lim}\frac{1}{\beta }\frac{1}{1+\frac{1}{a_{diff}\beta }}=\frac{1}{\beta }=1+\frac{R_{f}}{R_{-}}\;\;.$$

In other words, *β* sets the attenuation from the output to the input, forcing *v*_out_ to be larger than *v*_in_ by a factor 1/*β*. As long as *a*_diff_*β* >> 1, closed-loop gain is controlled by the feedback network, and 1/*β* represents the gain of the non-inverting voltage-feedback amplifier configuration.

**Buffer amplifier.** As explained earlier, an operational amplifier with a series–parallel negative feedback mimics a voltage-controlled voltage source. Thus, ideally, it would have infinite input impedance and zero output impedance [340]. Conveniently, closing the loop with a series–parallel negative feedback offers an effective increase in the differential-mode input impedance, which turns out to be governed by the desensitivity factor of 1 + *a*_diff_*β* in accordance with the Miller effect. In addition, it also offers an effective decrease in output impedance by the same factor of 1 + *a*_diff_*β*. Finally, by applying a negative voltage feedback, a reduction in amplifier native non-linearities and distortion, such as voltage-dependent gain variations, is also accomplished [331,333,334,340,342]. Since the highest loop gain *a*_diff_*β* for passive feedback networks is achieved when *β* = 1 and, consequently, *a*_diff_*β* = *a*_diff_, ideal voltage source behavior is most closely approximated when *A*_ideal_ is equal to 1 V/V or simply 1 (A11). In modern amplifiers, this is commonly realized with zero feedback resistance *R*_f_ and (virtually) infinite *R*_−_ (Figure A8). The non-inverting unity closed-loop gain *A*_ideal_ = 1 means that the output voltage, *v*_out_, follows the input voltage at the non-inverting input terminal, i.e., *v*_out_ ≈ *v*_+_ = *v*_in_ = *v*_in,non-inv_; hence, this special case of a non-inverting configuration is called the voltage follower (see subchapter 2.6 in [331]). On the other hand, it is also called a voltage buffer due to its isolation or buffering properties: high input impedance and low output impedance. In this paper, the terms “voltage follower” and “buffer” are used interchangeably. With respect to the discussion given in Section 3.2 and Assumption 5, buffer amplifiers with unity closed-loop gain remain to be a paramount special case for the rest of this appendix.

**Frequency-dependent, first-order model.** So far, the analysis (A9)–(A11) has neglected the influence of frequency, assuming that the operational amplifier has an infinite bandwidth. However, due to the presence of reactive elements, reality is frequency-dependent, and so the previous considerations are valid only at 0 Hz. To account for finite bandwidth as in [344], Equation (A9) becomes affected by frequency (A12):

(A12) $$A_{diff}\left(f\right)=\frac{1}{\beta (f)}\frac{1}{1+\frac{1}{a_{diff}(f)\beta (f)}}=\;\frac{a_{diff}(f)}{1+a_{diff}\beta (f)}\;\;,$$

where *β*(*f*) is defined with impedances *Z*_−_ and *Z*_f_ rather than with corresponding resistances *R*_−_ and *R*_f_ (A13):

(A13) $$\beta \left(f\right)=\frac{Z_{-}}{Z_{-}+Z_{f}}\;.$$

Now that frequency-dependence is included in the model, the specific case when negative feedback turns into positive feedback can be observed. This would result in instability and a self-sustaining oscillatory response, as described in [345]. To grasp this, phase shift around the feedback loop can be investigated—specifically, the case when the loop gain *a*_diff_*β*(*f*) becomes real and negative, since the feedback would then turn from negative to positive. In the polar form, the loop gain *a*_diff_*β*(*f*) can be expressed by means of a magnitude |*a*_diff_*β*(*f*)| and a phase (angle, argument) ∡*a*_diff_*β*(*f*) as |*a*_diff_*β*(*f*)|${\cdot e}^{j∡a_{diff}\beta (f)}$ [268,269]. It can be observed that *a*_diff_*β*(*f*) becomes real and negative when the loop phase shift ∡*a*_diff_*β*(*f*) is equal to −180°. Therefore, specific frequency *f*_180_ can be investigated, such that ∡*a*_diff_*β*(*f*_180_) = −180°. Now, if |*a*_diff_*β*(*f*_180_)| = 1 (unity loop gain at *f*_180_), *a*_diff_*β*(*f*_180_) is equal to −1, and so the desensitivity as the denominator in (A12) becomes 0, and the closed-loop gain *A*_diff_(*f*_180_) rises to infinity. Thus, any noise or disturbance that are present in the amplifier and that contain the respective edge-case frequency *f*_180_ would yield an undamped oscillatory response with a constant amplitude, which would remain present at the output indefinitely, even without any signal applied at the input. This condition represents the case of marginal stability. Moreover, if |*a*_diff_*β*(*f*_180_)| > 1, loop gain *a*_diff_*β*(*f*_180_) would be more negative than −1 (e.g., −2). As a result, the feedback network would iteratively add a larger and larger *f*_180_ frequency component to the input. This would yield a growing oscillatory response that would eventually be limited by non-linearities of the amplifier voltage transfer curve or external circuitry, such as a clamping network.

To ensure stability, condition ∡*a*_diff_*β*(*f*) > −180° must be satisfied at the frequencies where |*a*_diff_*β*(*f*)| ≥ 1. In other words, frequency *f*_180_, at which ∡*a*_diff_*β*(*f*) drops to −180°, must be higher than the frequency at which |*a*_diff_*β*(*f*)| drops to 1. This way, condition *a*_diff_*β*(*f*_180_) > −1 will be achieved. To bypass the influence of lowpass filters and lag networks, unintentionally formed by internal amplifier capacitances and finite impedances from the preceding stages, due to which ∡*a*_diff_*β*(*f*) may drop to −180° at a frequency lower than the frequency at which |*a*_diff_*β*(*f*)| drops below unity, real-world multistage amplifiers are often internally compensated with the addition of a capacitor in the second stage (see subchapter 4.9 in [333], and subchapter 7.2 in [346]). In that case, open-loop gain can be modeled as a first-order system *a*_diff_(*f*) with a DC open-loop gain *a*_0_ (A14) [344]:

(A14) $$a_{diff}\left(f\right)=\frac{a_{0}}{1+j\frac{f}{f_{a}}}=\frac{a_{0}}{1+j\frac{\omega }{\omega_{a}}}\;.$$

This model is also referred to as the single-pole model, which will become clear once the relation between the frequency response and poles is established in Section 4.1. This single, dominant compensation pole determines the high-side cutoff, corner, or half-power frequency *f*_a_ that can be estimated as the frequency at which the maximum value *a*_0_ of magnitude |*a*_diff_(*f*)| becomes attenuated by 3 dB (or $\sqrt{2}$ times) [347]. In other words, the bandwidth of the operational amplifier is no longer infinite, but rather defined by *f*_a_. Therefore, *f*_a_ is called the open-loop bandwidth. As a result, *a*_diff_(*f*) frequency characteristic (frequency response) is no longer flat. Rather, its magnitude characteristic (magnitude response) |*a*_diff_(*f*)| starts to slope downward with frequency, falling at *f*_a_ to a value that is 3 dB lower than *a*_0_. This behavior can be approximated graphically with the use of asymptotic Bode plots, as in Figure A9 [273,341,348,349]. In accordance with the Bode linear piecewise approximation of this first-order (single-pole) system, *f*_a_ is equal to the break frequency; once it is reached, the magnitude characteristic (magnitude response) in decibels (dB), |*a*_diff_(*f*)|_dB_ = 20log_10_|*a*_diff_(*f*)|, starts to roll off with a slope of 20 dB/decade or, equivalently, 6 dB/octave [347]. Also, the phase characteristic (phase response) ∡*a*_diff_(*f*) achieves −90° of added lagging (negative, delaying) phase shifts from *f*_a_/10 to 10*f*_a_. In reality, however, one must be aware that |*a*_diff_(*f*)| and ∡*a*_diff_(*f*) are continuous asymptotic. Accordingly, Bode approximations yield a maximum phase error of ±5.7° at 0.1*f*_a_ and 10*f*_a_, and a maximum magnitude error of 3 dB at *f*_a_ [273,349].

Overall, magnitude response |*a*_diff_(*f*)| and phase response ∡*a*_diff_(*f*) decrease monotonically with frequency. In fact, the same behavior of *a*_diff_(*f*) has been assumed throughout the entire stability analysis in the preceding pages. Namely, the described procedure for assessing stability by comparing the frequency of unity loop gain (|*a*_diff_*β*(*f*)| = 1) with frequency *f*_180_ is a simplification of the Nyquist stability criterion that can be applied in the case of monotonic frequency response [350,351]. Since |*a*_diff_(*f*)| and ∡*a*_diff_(*f*) of internally compensated operational amplifiers are largely monotonically decreasing, this simplified procedure, along with Bode plots (Figure A9), will continue to be used in the remaining discussions.

In decibels (dB) [347], magnitude of the loop gain |*a*_diff_*β*(*f*)|_dB_ can be expressed as the difference between |*a*_diff_(*f*)|_dB_ and |(1/*β*)(*f*)|_dB_ (A15):

$$|a_{diff}\beta \left(f\right){|}_{dB}=20\log_{10}\left[a_{diff}\beta \left(f\right)\right]=20\log_{10}\left[\frac{a_{diff}}{\frac{1}{\beta }}\left(f\right)\right]$$

(A15) $$=20\log_{10}\left[a_{diff}(f)\right]-20\log_{10}\left[\frac{1}{\beta }(f)\right]={{|a}_{diff}\left(f\right)|}_{dB}-{\left|\frac{1}{\beta }(f)\right|}_{dB}.$$

Similarly, the relation between the phases can be explored. In the polar form, the ratio of exponential functions turns into a subtraction of exponents [268,348] (A16):

$$a_{diff}\beta \left(f\right)=\frac{a_{diff}\left(f\right)}{\frac{1}{\beta }\left(f\right)}=\frac{\left|a_{diff}\left(f\right)\right|e^{j∡a_{diff}(f)}}{\left|\frac{1}{\beta }\left(f\right)\right|e^{j∡\frac{1}{\beta }(f)}}=\frac{\left|a_{diff}\left(f\right)\right|}{\left|\frac{1}{\beta }\left(f\right)\right|}e^{j(∡a_{diff}-∡\frac{1}{\beta })(f)}$$

(A16) $$\to \;∡a_{diff}\beta \left(f\right)=∡a_{diff}\left(f\right)-∡\frac{1}{\beta }(f)\;.$$

Two important remarks can be highlighted. Firstly, the frequency of unity loop gain (|*a*_diff_*β*(*f*)| = 1 or |*a*_diff_*β*(*f*)|_dB_ = 0 dB) is achieved when |*a*_diff_(*f*)|_dB_ = |(1/*β*)(*f*)|_dB_ (A15). Graphically, this corresponds to the frequency *f*_cross_ at which magnitude responses |*a*_diff_(*f*)|_dB_ and |(1/*β*)(*f*)|_dB_ intersect (Figure A9). This frequency *f*_cross_ is called the crossover frequency.

Secondly, it can be approximated that the magnitude of the closed-loop gain, |*A*_diff_(*f*)|_dB_, tracks |(1/*β*)(*f*)|_dB_. Namely, the DC open-loop gain *a*_0_ is so high compared to 1/*β*(*f*)—i.e., *a*_diff_*β*(*f*) >> 1 in (A12)—that the ideal *A*_diff_(*f*) becomes defined solely by 1/*β*(*f*). This is shown in the following (A17):

$${A\left(f\right)}_{ideal}\approx {A_{diff}\left(f\right)}_{ideal}=\underset{a_{diff}\to \infty }{lim}A_{\mathrm{diff}}\left(f\right)=\underset{a_{\mathrm{diff}}\to \infty }{\lim}\frac{1}{\beta \left(f\right)}\frac{1}{1+\frac{1}{a_{diff}\left(f\right)\beta \left(f\right)}}$$

(A17) $$=\frac{1}{\beta \left(f\right)}=1+\frac{Z_{f}}{Z_{-}}\;.$$

Specifically, at 0 Hz, where only resistive components remain influential, DC closed-loop gain *A*_0_ is obtained (A18):

(A18) $${A\left(0\right)}_{ideal}=A_{0}=\frac{1}{\beta \left(0\right)}=1+\frac{R_{f}}{R_{-}}.$$

This result is in accordance with the earlier frequency-independent model (A9)–(A11) and, specifically, yields a unity closed-loop gain *A*_0_ = *A*_ideal_ = 1 V/V = 1 for a voltage follower configuration from Figure A8. However, this behavior of *A*_diff_(*f*) is true only up to the crossover frequency *f*_cross_. At *f*_cross_, |(1/*β*)(*f*)|_dB_ becomes equal to |*a*_diff_(*f*)|_dB_ that is left available for driving the desired closed-loop response, and so, at frequencies *f* > *f*_cross_, |*A*_diff_(*f*)|_dB_ follows the downward sloping of |*a*_diff_(*f*)|_dB_. In other words, just as *f*_a_ corresponds to the open-loop bandwidth available for the given *a*_0_, *f*_cross_ in this case corresponds to the closed-loop bandwidth available for the realized *A*_0_. This means that, analogously to *f*_a_, *f*_cross_ can also be denoted by *f*_A_. To draw the parallel further, similarly to the expression for the open-loop gain *a*_diff_(*f*) (A14), the closed-loop gain *A*_diff_(*f*) can also be rewritten with respect to its DC closed-loop gain *A*_0_ (A18) and its high-side cutoff frequency *f*_cross_ = *f*_A_ as follows (A19):

(A19) $$A_{diff}\left(f\right)=\frac{A_{0}}{1+j\frac{f}{f_{A}}}=\frac{A_{0}}{1+j\frac{\omega }{\omega_{A}}}\;\;.$$

Overall, the resulting |*A*_diff_(*f*)|_dB_ of the non-inverting configuration follows |(1/*β*)(*f*)|_dB_ up to *f*_cross_ = *f*_A_ and then continues to follow |*a*_diff_(*f*)|_dB_, as indicated by the thick solid line in Figure A9. Incidentally, it can be added that the non-inverting closed-loop gain |*A*_diff_(*f*)|_dB_ also corresponds to the noise gain [334,352]. Various illustrative examples of open-loop and closed-loop frequency response can be found in [344].

**Unity-gain stability of the first-order model, phase margin, and gain margin.** The discovered frequency *f*_cross_ is in fact the aforementioned frequency, at which |*a*_diff_*β*(*f*)| drops to 1 (or 0 dB), and which can be observed with respect to *f*_180_ in order to assess stability. The amount of additional phase shift, left between ∡*a*_diff_*β*(*f*_cross_) and −180°, reveals how close the system is to becoming unstable for the chosen closed-loop gain *A*_0_. Alternatively, the so-called rate-of-closure method could be used, in which the difference in slopes between |*a*_diff_(*f*)|_dB_ and |(1/*β*)(*f*)|_dB_ at their intersection *f*_cross_ is observed, as explained in [345,352]. As seen from (A17,A18), the accomplished closed-loop gain *A*_0_ will depend on the chosen feedback network. Because the feedback network is assumed to be purely resistive (see the dashed line example in Figure A9), it does not introduce additional phase shifts at frequencies of interest (Assumption 1): ∡(1/*β*)(*f*) = 0°. Also, |(1/*β*)(*f*)|_dB_ is flat and constant. Accordingly, a lower value of *β*—i.e., a higher value of 1/*β*—yields not only higher *A*_0_ (A18), but also lower *f*_cross_. As a result, for a lower value of *β*, greater phase shift is left available between ∡*a*_diff_*β*(*f*_cross_) and the critical −180°. Therefore, to obtain the worst-case stability calculation, the highest value of *β* should be considered. Since maximum *β* for commonly used passive feedback networks is 1, this means that stability should in fact be observed for the desired voltage follower configuration. Namely, unity *β* means that there is no attenuation in the feedback path and that the entire output signal, rather than its portion, must be fed back to the input. Also, unity *β* allows for the least attenuation of the oscillation present in the loop. In other words, to allow operational amplifiers to function all the way down to unity closed-loop gain, unity gain stability for *β* = 1 should be observed. In that case, the loop gain also achieves its maximum value *a*_diff_*β*(*f*) = *a*_diff_(*f*). Now, since |(1/*β*)(*f*)|_dB_ = 0 dB, observing the crossover frequency *f*_cross_ means observing the intersection between |*a*_diff_(*f*)|_dB_ and the frequency axis. Consequently, instead of the crossover frequency *f*_cross_ of unity loop gain (|*a*_diff_*β*(*f*_cross_)|_dB_ = 0 dB), frequency *f*_1_ of unity open-loop gain (|*a*_diff_(*f*_1_)|_dB_ = 0 dB) can now be observed. Analogously, this frequency *f*_1_ is called the open-loop unity-gain crossover frequency, or the transition frequency. Just as *f*_a_ corresponds to the open-loop bandwidth and *f*_A_ corresponds to the bandwidth realized with a specific closed-loop gain *A*_0_ > 1, *f*_1_ corresponds to the unity-gain closed-loop bandwidth realized with *A*_0_ = 1. The amount of additional phase shift left available between ∡*a*_diff_(*f*_1_) and −180° is specifically called the phase margin. Aside from ensuring a positive phase margin, it is also important to observe how large it is. Namely, insurance against instability does not imply insurance against oscillatory response. The actual amount of a positive phase margin and its proximity to zero will determine the amount of gain peaking in the closed-loop magnitude response, as well as the amount of overshoot and ringing in the time-domain transient response. Therefore, phase margin should not only be positive, but also commonly at least +45° to insure against significant overshoots and ringing, as well as to save leeway for additional phase shift that could occur due to production tolerances and variations in operating conditions [273,335,341,345,353]. In the case of second-order systems specifically, these issues are usually observed through the damping ratio and the quality factor [341,345,354], which will be revisited later in Section 4.3. Phase margin can also be observed for closed-loop gains higher than unity. In that case, as described earlier, *f*_cross_ = *f*_A_ would be observed for a particular *A*_0_ > 1, along with the phase shift left available between ∡*a*_diff_*β*(*f*_cross_) and −180°. However, as explained in this paragraph, the phase margin for a unity closed-loop gain represents the worst-case stability condition (maximum *β* and *f*_cross_ = *f*_1_). Interesting examples of gain peaking and oscillatory response for different values of *β* can be found in Figures 8.5 and 8.6 in [345]. Alternatively, aside from phase margin, gain margin can be observed as well [345]. While the phase margin for a specific *A*_0_ represents the number of degrees by which ∡*a*_diff_*β*(*f*_cross_) can be lowered before it reaches −180° at *f*_180_, gain margin represents the number of decibels by which |*a*_diff_*β*(*f*_180_)|_dB_ can be increased before it reaches 1 (0 dB) at *f*_cross_ (Figure A9). The most stringent, unity-gain stabilization yields the minimum phase margin and minimum gain margin available.

**Gain-bandwidth product.** Lastly, since a first-order model is considered (A14,A19), *f*_1_ also determines the gain-bandwidth product (GBP or GBW), which establishes the relation between various aforementioned gains and their respective bandwidths (A20):

(A20) $$GBP=a_{0}{\cdot f}_{a}=A_{0}\cdot f_{A}=1\cdot f_{1}\;.$$

For the assumed first-order model and no additional deviation in GBP across the observed frequency range, (A20) shows that the product of achieved gain and bandwidth is constant. It also explains why lower *β* (higher 1/*β* and higher *A*_0_) achieves lower *f*_cross_ = *f*_A_. Although closing the operational amplifier with a series–parallel negative feedback reduced the maximum voltage gain from *a*_0_ to *A*_0_, the gain is now stabilized and also, the bandwidth is increased from *f*_a_ to *f*_A_—with the caveat that there exists a finite phase margin. In addition, as mentioned earlier, the differential-mode input impedance is increased, whereas the output impedance, along with native non-linearities and distortion, are decreased [331,333,334,340,342].

**Generalized analysis: frequency-dependent feedback network and higher-order models.** Having a single pole, the so far analyzed first-order model should guarantee a maximum added lagging phase shift of no greater than 90° and a roll-off in magnitude of no greater than 20 dB/decade (Figure A9). However, even this model is sufficient only for standard, purely resistive feedback networks. In such cases, 1/*β* is constant and flat, and so the only component contributing to the loop phase shift is the amplifier. The corresponding example of assessing its stability was given in dashed lines in Figure A9. On the contrary, when reactive components are present in the feedback path, additional poles, along with their break frequencies, are created, which further reduce the phase margin. Therefore, frequency-dependence of 1/*β* and its phase shifts must also be considered, as in [352] (see the dotted line example in Figure A9). Aside from 1/*β*(*f*), open-loop gain *a*_diff_(*f*) itself could introduce additional poles, depending on whether the amplifier is compensated or not, and on the applied compensation technique. In that case, the open-loop unity-gain crossover frequency (transition frequency) *f*_1_ could end up being lower than the corresponding GBP. When such additional break frequencies are present, a first-order (single-pole) model is replaced with higher-order (multi-pole) models. Because 1/*β*(*f*) in such cases can be an increasing function of frequency (see the dotted line example in Figure A9), to assess stability, Bode plots of *a*_diff_*β*(*f*) and frequency *f*_cross_ are again observed rather than simply *a*_diff_(*f*) and *f*_1_, with a caveat that the Bode approximation yields an absolute maximum phase shift error of 5.7° at 0.1*f*_break_ and 10*f*_break_ of each break frequency *f*_break_ (Figure A9) [273,349,352]. Also, in such complexified cases, it might be erroneous to equate *f*_cross_ with the closed-loop bandwidth *f*_A_, since |*A*_diff_(*f*)|_dB_ might be increasing or decreasing with frequency at *f* < *f*_cross_.

Aside from the dotted line example in Figure A9, various examples can be found in [333,345,346,352,355,356] and chapter IV in [335]. For instance, capacitive load creates an additional break frequency with the amplifier closed-loop output impedance, causing additional lagging phase shifts and, possibly, a non-linear response as a result of output voltage slew rate limitation due to increased demand for current from the amplifier output [357,358,359]. Moreover, closed-loop output impedance may exhibit significant inductive-like behavior and increase with frequency (refer to [358] and to Figure 4.53 in [333]). This effective inductor would create a series *LC*-resonant circuit with the capacitive load. On the other side, another break frequency would be introduced by the parallel *Z*_f_||*Z*_−_, in combination with the capacitance seen at the inverting input [360].

The influence of these reactive components can be observed in more detail for the case of the desired voltage follower configuration from Figure A8, as depicted in Figure A10. As a conservative estimate with respect to the phase shifts introduced by a single pole (Figure A9), in order for these additional poles not to affect the stability and phase margin of the devised first-order (single-pole) model (A14,A19), their respective break frequencies should be at least a decade higher than the unity-gain closed-loop bandwidth *f*_1_. For a voltage follower amplifier, *Z*_f_ is close to 0 Ω. Therefore, for the worst case of the lowest break frequency, both the total capacitance at the inverting input and the total capacitance at the output of the amplifier can be considered in parallel. On the one hand, at the inverting input, aside from external pin, pad, and layout capacitance *C*_−_ and the internal common-mode input capacitance *C*_com−_ that are seen between the inverting input and circuit common, internal *C*_diff_ of the differential-mode input impedance *Z*_diff_ must be accounted for. Namely, just like *Z*_coupling_ in Section 3.1, *Z*_diff_ can also be modeled as an *RC*-parallel [330]: the differential-mode input resistance, *R*_diff_, shunted by the differential-mode input capacitance, *C*_diff_. This capacitance describes the effective frequency-dependent decrease in *R*_diff_, arising due to a decrease in the open-loop gain and an increase in input differential voltage *v_+_* − *v*_−_ with increasing frequency. For the measured signal looking into the non-inverting input, *C*_diff_ is also referenced to the circuit common [345]. On the other hand, the capacitance at the output of the amplifier comprises the cable capacitance *C*_cable_. In addition, for negligibly low cable resistance, *C*_cable_ is in parallel with the input capacitance of the next stage, *C*_load_. These two capacitances form *C*_out_ = *C*_cable_||*C*_load_, which describes the total effective capacitive load. Overall, the total equivalent worst-case capacitance *C*_total_ is then seen at the input as a parallel combination of *C*_−_, *C*_com−_, *C*_diff_, *C*_cable_, and *C*_load_. It creates a break frequency with the closed-loop output impedance [360]. Taking a fairly conservative estimate of 100 Ω for the closed-loop output impedance to account for its inductive behavior (refer to Figure 4.53 in [333]) and assuming a typical GBP = *f*_1_ of about 1 MHz based on [336,337,338], total capacitance *C*_total_ added to amplifier inverting input terminal and output should not be higher than about 159 pF (A21):

(A21) $$\frac{1}{2\pi \cdot 100\;Ω\cdot C_{total}}>10\;MHz\to C_{total}<\frac{1}{2\pi \cdot 100\;Ω\cdot 10\;MHz}\approx 159\;pF.$$

A frequency of 10 MHz is used as a limit to account for the fact that the phase shifts introduced by the pole, in accordance with the earlier first-order (single-pole) Bode approximation (Figure A9), start a decade below the corresponding break frequency of the pole. In the context of active biopotential electrodes, introduced in Section 3.2 and further investigated in Section 4, the voltage follower model in Figure A8 and Figure A10 corresponds to the buffer preamplifier mounted on a buffer active electrode. From that point of view, if data acquisition (DAQ) systems are used as the next stage, comprising modern instrumentation amplifiers such as AD620 [361] and AD8221 [362] (Analog Devices, Inc., Wilmington, MA, USA), INA333 [363] (Texas Instruments, Inc., Dallas, TX, USA), and ISL28x3x [364] (Renesas Electronics, Tokyo, Japan), or integrated biopotential analog front-ends such as ADS1292 [365] (Texas Instruments, Inc., Dallas, TX, USA) and MAX30001 [366] (Analog Devices, Inc., Wilmington, MA, USA), the presented *C*_load_ is about several tens of pF in the worst case. On the other hand, the amplifiers, considered as buffer preamplifiers [336,337,338], add several pF of *C*_com−_ + *C*_diff_, whereas *C*_−_ should be minimized to the order of 1 pF with a proper PCB design. Therefore, the described capacitances, that are present at the inverting input and the output of a buffer preamplifier, will be neglected in Section 4.1 and Section 4.2, and recalled later in Section 4.3.

**Stability in practical biopotential measurements.** Despite encouraging results, there are cases when values of these additional capacitances require external means of restoring stability and the use of higher-order models instead of the first-order model. For instance, from the aspect of active biopotential electrodes with mounted preamplifiers (Section 3.2 and Section 4), long cable connections from electrodes to the offboard remote DAQ unit could significantly increase *C*_out_ = *C*_cable_||*C*_load_ due to increased cable capacitance *C*_cable_. Namely, even low-capacitance cables exhibit about 1 pF/cm of *C*_cable_ [259,272,356], and as seen from the graphs in preamplifier datasheets such as [336], *C*_out_ values of only several tens of pF could already start to significantly affect the phase margin of the preamplifier. A similar stability issue could arise in the case of shield drivers and driven right leg (DRL) circuits (see [367,368] and Appendix 2 in [143]). In addition, parasitic inductance of the cable can be accounted for [367]. Similarly, just like the inductive component of the output impedance ([358] and Figure 4.53 in [333]), the inductive component of amplifier input impedance could also be accounted for [352]. In general, such inductive components form tank *LC*-circuits with analyzed parallel capacitances and produce high-frequency ringing at the amplifier output. Thus, aside from the goal of reducing bias and quiescent current, the goal of mitigating potential oscillations at higher frequencies is another reason for avoiding the use of high-bandwidth (high-speed) operational amplifiers [333,355,367]. Further, more complex circuit techniques applied on the preamplifier, such as bootstrapping and power supply bootstrapping, which will be mentioned at the end of Section 4.2, also require validation of stability [369,370,371,372]. Stability analysis plays an important role in a transimpedance amplifier configuration as well [293,294,327,328]. Another example of parasitic effects can be found in bioimpedance measurement systems [167]. Common solutions to these issues include external out-of-loop and in-loop compensation techniques with the use of additional feedback capacitance, output *RC* components, and the feedforward approach [333,345,346,355,356,373]. Application notes on these techniques can be found in [336,357,358,359,360].

The influence of *C*_total_ will be revisited in Section 4.3 in the context of the frequency response of the entire system. The issue of cable and layout capacitance will be discussed in greater detail in the context of the capacitance present at the non-inverting input terminal of the buffer preamplifier, which will be introduced in paragraph “Parasitic input capacitance” in Section 4.1. Also, in Section 4.1, more on stability at the level of the entire system can be found.
