# Supplementary material for: A Systematic Review of Design of Electrodes and Interfaces for Non-Contact and Capacitive Biomedical Measurements: Terminology, Electrical Model, and System Analysis
Source: Sensors (Basel). 2026 Feb 22;26(4):1374. doi: 10.3390/s26041374 (PMC12944335; doi:10.3390/s26041374)
Supplement: Supplementary file 1 [file sensors-26-01374-s001.zip › sensors-3934601-supplementary.pdf]

A Systematic Review of Design of Electrodes and Interfaces for Non-Contact and Capacitive Biomedical Measurements: Terminology, Electrical Model, and System Analysis: **PRISMA 2020 Statement**

Supplementary Material to:

Klaić, L.; Cindrić, D.; Stanešić, A.; Cifrek, M. A Systematic Review of Design of Electrodes and Interfaces for Non-Contact and Capacitive Biomedical Measurements: Terminology, Electrical Model, and System Analysis.

Correspondence to: Luka Klaić, University of Zagreb Faculty of Electrical Engineering and Computing, Unska ulica 3, 10000 Zagreb, Croatia. Email address: luka.klaic@fer.unizg.hr

The following supplementary material provides PRISMA 2020 for Abstracts Checklist (Table S1), PRISMA 2020 Checklist (Table S2), and PRISMA 2020 Flow Diagram (Figure S1) for the corresponding study. The structure and content of the supplementary material is based on guidelines provided in [100,101]. A detailed report is available in the paper itself in accordance with the following remark.

Remark:
The study is only partially a meta-analysis. Namely, as discussed in Abstract, Introduction (Section 1), Materials and Methods (Section 2), and Conclusions (Section 5), the purpose of this study is to analytically demonstrate and evaluate the common practice, as well as to remedy occasional inconsistencies and inaccuracies in terminology and research methodology. Ultimately, the study has three objectives and leads to four contributions, as stated in Introduction (Section 1.2). Results are corroborated with examples from existing literature with a scope extending from early works (1960s) to the newest research papers in 2025. With this perspective in mind, Abstract is tailored in a way to highlight the objectives and contributions of the entire research. Accordingly, the study does not include data aggregation or groups of participants, nor did it investigate such studies or their reports. Therefore, some of the items in PRISMA checklists are not reported, and some of the items in PRISMA checklists are only partially applicable with respect to the context and focus of the study. In the following pages, these items are denoted by an asterisk (*) in the rightmost columns.

Table S1. PRISMA 2020 for Abstracts Checklist [100,101].

Section and Topic	Item #	Checklist item	Reported (Yes/No)
**TITLE**			
Title	1	Identify the report as a systematic review.	Yes
**BACKGROUND**			
Objectives	2	Provide an explicit statement of the main objective(s) or question(s) the review addresses.	Yes
**METHODS**			
Eligibility criteria	3	Specify the inclusion and exclusion criteria for the review.	*No (reported in PRISMA 2020 Checklist)
Information sources	4	Specify the information sources (e.g. databases, registers) used to identify studies and the date when each was last searched.	*No (reported in PRISMA 2020 Checklist)
Risk of bias	5	Specify the methods used to assess risk of bias in the included studies.	*No (reported in PRISMA 2020 Checklist)
Synthesis of results	6	Specify the methods used to present and synthesise results.	Yes
**RESULTS**			
Included studies	7	Give the total number of included studies and participants and summarise relevant characteristics of studies.	*No (reported in PRISMA 2020 Checklist)
Synthesis of results	8	Present results for main outcomes, preferably indicating the number of included studies and participants for each. If meta-analysis was done, report the summary estimate and confidence/credible interval. If comparing groups, indicate the direction of the effect (i.e. which group is favoured).	*No (reported in PRISMA 2020 Checklist)
**DISCUSSION**			
Limitations of evidence	9	Provide a brief summary of the limitations of the evidence included in the review (e.g. study risk of bias, inconsistency and imprecision).	*No (reported in PRISMA 2020 Checklist)
Interpretation	10	Provide a general interpretation of the results and important implications.	*No (reported in PRISMA 2020 Checklist)
**OTHER**			
Funding	11	Specify the primary source of funding for the review.	*No (reported in PRISMA 2020 Checklist)
Registration	12	Provide the register name and registration number.	*Not applicable

Table S2. PRISMA 2020 Checklist [100,101].

Section and Topic	Item #	Checklist item	Location where item is reported
**TITLE**			
Title	1	Identify the report as a systematic review.	Title
**ABSTRACT**			
Abstract	2	See the PRISMA 2020 for Abstracts checklist.	Abstract<br>1. Introduction<br>2. Materials and Methods
**INTRODUCTION**			
Rationale	3	Describe the rationale for the review in the context of existing knowledge.	Objective 1 in 1.2. Objectives and contributions
Objectives	4	Provide an explicit statement of the objective(s) or question(s) the review addresses.	List of 3 objectives and 4 contributions in 1.2. Objectives and contributions
**METHODS**			
Eligibility criteria	5	Specify the inclusion and exclusion criteria for the review and how studies were grouped for the syntheses.	2. Materials and Methods
Information sources	6	Specify all databases, registers, websites, organisations, reference lists and other sources searched or consulted to identify studies. Specify the date when each source was last searched or consulted.	2. Materials and Methods
Search strategy	7	Present the full search strategies for all databases, registers and websites, including any filters and limits used.	2. Materials and Methods
Selection process	8	Specify the methods used to decide whether a study met the inclusion criteria of the review, including how many reviewers screened each record and each report retrieved, whether they worked independently, and if applicable, details of automation tools used in the process.	2. Materials and Methods
Data collection process	9	Specify the methods used to collect data from reports, including how many reviewers collected data from each report, whether they worked independently, any processes for obtaining or confirming data from study investigators, and if applicable, details of automation tools used in the process.	2. Materials and Methods
Data items	10a	List and define all outcomes for which data were sought. Specify whether all results that were compatible with each outcome domain in each study were sought (e.g. for all measures, time points, analyses), and if not, the methods used to decide which results to collect.	2. Materials and Methods
	10b	List and define all other variables for which data were sought (e.g. participant and intervention characteristics, funding sources). Describe any assumptions made about any missing or unclear information.	2. Materials and Methods
Study risk of bias assessment	11	Specify the methods used to assess risk of bias in the included studies, including details of the tool(s) used, how many reviewers assessed each study and whether they worked independently, and if applicable, details of automation tools used in the process.	2. Materials and Methods
Effect measures	12	Specify for each outcome the effect measure(s) (e.g. risk ratio, mean difference) used in the synthesis or presentation of results.	*Not applicable
Synthesis methods	13a	Describe the processes used to decide which studies were eligible for each synthesis (e.g. tabulating the study intervention characteristics and comparing against the planned groups for each synthesis (item #5)).	*Not applicable
	13b	Describe any methods required to prepare the data for presentation or synthesis, such as handling of missing summary statistics,	*2. Materials and Methods

Section and Topic	Item #	Checklist item	Location where item is reported
		or data conversions.	
	13c	Describe any methods used to tabulate or visually display results of individual studies and syntheses.	*Not applicable
	13d	Describe any methods used to synthesize results and provide a rationale for the choice(s). If meta-analysis was performed, describe the model(s), method(s) to identify the presence and extent of statistical heterogeneity, and software package(s) used.	*2. Materials and Methods
	13e	Describe any methods used to explore possible causes of heterogeneity among study results (e.g. subgroup analysis, meta-regression).	*Not applicable
	13f	Describe any sensitivity analyses conducted to assess robustness of the synthesized results.	*2. Materials and Methods
Reporting bias assessment	14	Describe any methods used to assess risk of bias due to missing results in a synthesis (arising from reporting biases).	*2. Materials and Methods
Certainty assessment	15	Describe any methods used to assess certainty (or confidence) in the body of evidence for an outcome.	*2. Materials and Methods
**RESULTS**			
Study selection	16a	Describe the results of the search and selection process, from the number of records identified in the search to the number of studies included in the review, ideally using a flow diagram.	PRISMA 2020 Flow Diagram
	16b	Cite studies that might appear to meet the inclusion criteria, but which were excluded, and explain why they were excluded.	2. Materials and Methods
Study characteristics	17	Cite each included study and present its characteristics.	*3. Electrodes<br>4. System Analysis
Risk of bias in studies	18	Present assessments of risk of bias for each included study.	*Not applicable
Results of individual studies	19	For all outcomes, present, for each study: (a) summary statistics for each group (where appropriate) and (b) an effect estimate and its precision (e.g. confidence/credible interval), ideally using structured tables or plots.	*Not applicable
Results of syntheses	20a	For each synthesis, briefly summarise the characteristics and risk of bias among contributing studies.	*Not applicable
	20b	Present results of all statistical syntheses conducted. If meta-analysis was done, present for each the summary estimate and its precision (e.g. confidence/credible interval) and measures of statistical heterogeneity. If comparing groups, describe the direction of the effect.	*Not applicable
	20c	Present results of all investigations of possible causes of heterogeneity among study results.	*Not applicable
	20d	Present results of all sensitivity analyses conducted to assess the robustness of the synthesized results.	*Not applicable
Reporting biases	21	Present assessments of risk of bias due to missing results (arising from reporting biases) for each synthesis assessed.	*Not applicable
Certainty of evidence	22	Present assessments of certainty (or confidence) in the body of evidence for each outcome assessed.	*3. Electrodes<br>4. System Analysis
**DISCUSSION**			
Discussion	23a	Provide a general interpretation of the results in the context of other evidence.	4. System Analysis<br>5. Conclusions
	23b	Discuss any limitations of the evidence included in the review.	4. System Analysis<br>5. Conclusions

Section and Topic	Item #	Checklist item	Location where item is reported
	23c	Discuss any limitations of the review processes used.	2. Materials and Methods<br>4. System Analysis<br>5. Conclusions
	23d	Discuss implications of the results for practice, policy, and future research.	4. System Analysis<br>5. Conclusions
**OTHER INFORMATION**			
Registration and protocol	24a	Provide registration information for the review, including register name and registration number, or state that the review was not registered.	*Not applicable
	24b	Indicate where the review protocol can be accessed, or state that a protocol was not prepared.	*Not applicable
	24c	Describe and explain any amendments to information provided at registration or in the protocol.	*Not applicable
Support	25	Describe sources of financial or non-financial support for the review, and the role of the funders or sponsors in the review.	Funding
Competing interests	26	Declare any competing interests of review authors.	Conflicts of Interest
Availability of data, code and other materials	27	Report which of the following are publicly available and where they can be found: template data collection forms; data extracted from included studies; data used for all analyses; analytic code; any other materials used in the review.	Data Availability Statement

A Systematic Review of Design of Electrodes and Interfaces for Non-Contact and Capacitive Biomedical Measurements: Terminology, Electrical Model, and System Analysis:
PRISMA 2020 Statement

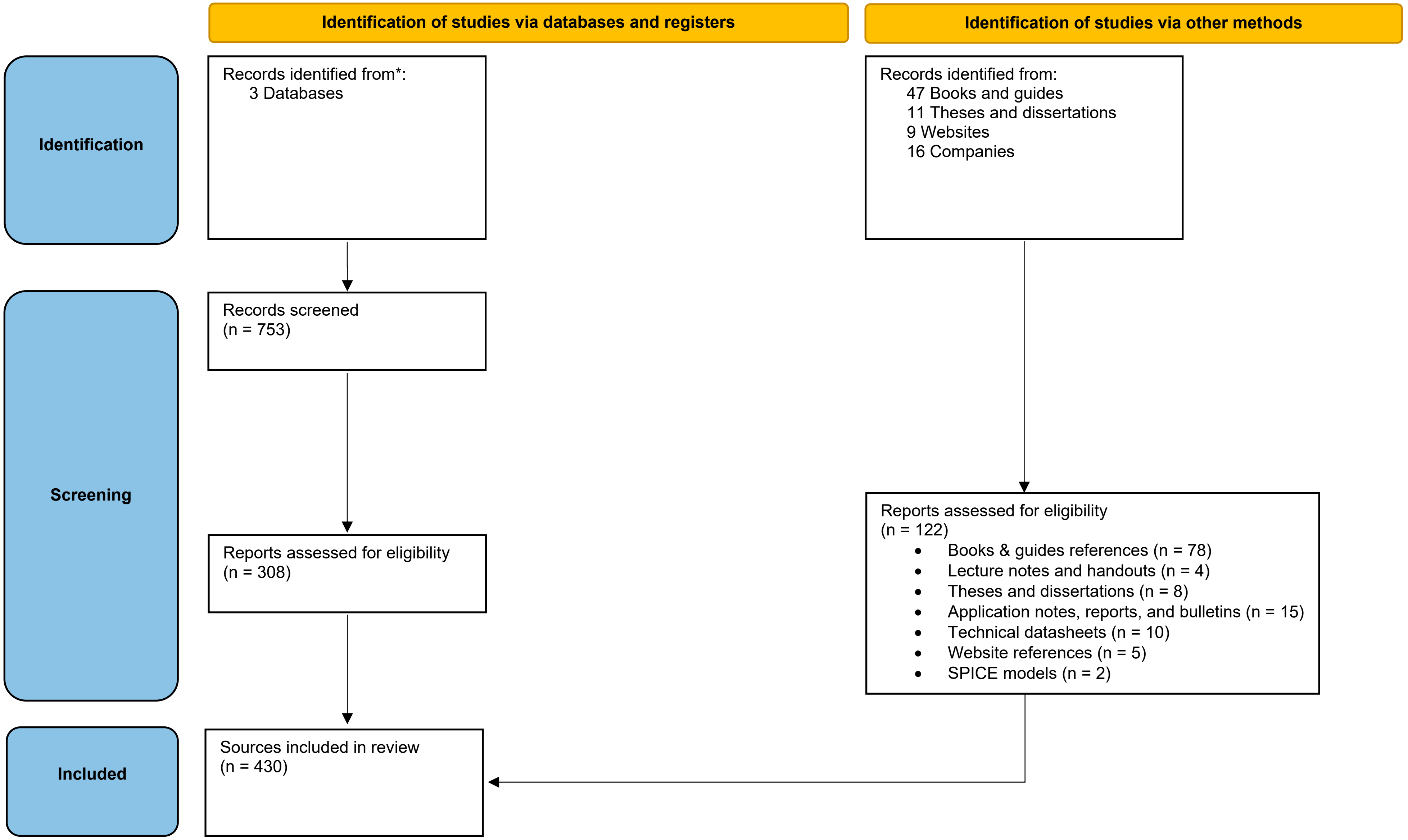

Figure S1. Simplified PRISMA 2020 Flow Diagram [100,101] in accordance with the opening remark.
